# Base‐Catalyzed Remote Hydrogermylation of Olefins

**DOI:** 10.1002/anie.202503573

**Published:** 2025-03-26

**Authors:** Eric Ahrweiler, Aymane Selmani, Franziska Schoenebeck

**Affiliations:** ^1^ Institute of Organic Chemistry RWTH Aachen University Landoltweg 1 52074 Aachen Germany

**Keywords:** Alkyl germane, Base‐catalyzed, Chain‐walk, Remote functionalization

## Abstract

Although remote functionalization has emerged as a powerful strategy for modifying unactivated sites that are traditionally challenging to functionalize, there has been no remote hydrogermylation known to date. This work reports the first remote hydrogermylation of alkenes, achieved through a rare base‐catalyzed approach‐completely free of added transition metal catalysts. The methodology is operationally simple, versatile, and capable of achieving up to 8‐carbon chain walks, overcoming the previous two‐carbon limit of base‐mediated processes.

## Graphical abstract

This study presents the first remote hydrogermylation of alkenes, utilizing a rare base‐catalyzed strategy in the absence of transition metal catalysts. The methodology is operationally simple and highly versatile, enabling up to 8‐carbon chain walks. This advance expands the scope of remote functionalization for modifying traditionally unactivated sites.

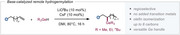

Remote functionalization is a strategic synthetic approach wherein an initial transformation occurs at an activated site, ultimately leading to the selective functionalization of a distal, typically unreactive position.^[^ ^1^, ^2^, ^3^, ^4^, ^5^, ^6^, ^7^ ^]^ This concept has attracted substantial interest in recent years due to its capacity to enable the modification of otherwise challenging, unactivated sites.^[^ ^8^, ^9^, ^10^, ^11^, ^12^ ^]^ By leveraging alternative disconnections and minimizing the number of synthetic steps, remote functionalization offers a more efficient pathway to complex molecular architectures. Additionally, it facilitates the use of readily accessible starting materials, further enhancing the practicality and sustainability of modern synthetic methodologies. It frequently proceeds via olefin migration in a so‐called “chain‐walk”, followed by a functionalization of the olefin in the remote position as the termination step. In this context, C─C,^[^ ^13^, ^14^ ^]^ C─Si,^[^ ^15^, ^16^ ^]^ and C─B^[^ ^8^, ^17^, ^18^, ^19^ ^]^ bond formations have been realized. The vast majority of chain‐walking processes are catalyzed by a transition metal. Indeed, a SciFinder analysis reveals over 1000 references related to metal‐catalyzed olefin migrations and more than 50 reports on metal‐catalyzed remote hydroboration or hydrosilylation.^[^ ^20^ ^]^ In stark contrast, olefin migrations that proceed in the absence of an added transition metal catalyst remain exceptionally rare. Only a handful of studies have demonstrated such transformations,^[^ ^21^ ^]^ typically relying on either superbase‐^[^ ^22^ ^]^ or Lewis acid catalysis^[^ ^23^, ^24^ ^]^ (employing B(C_6_F_5_)_3_ or HB(C_6_F_5_)_2_) under high‐temperature conditions).

Despite these advances, a remote germylation—specifically, the selective installation of a C(sp^3^)‐GeR_3_ moiety—is yet to be achieved. Due to their robustness, nontoxicity, and orthogonal reactivity, alkyl germanes hold significant potential as versatile functional handles for the construction of C(sp^3^)‐rich molecular scaffolds, particularly within modular functionalization strategies.^[^ ^25^, ^26^, ^27^, ^28^, ^29^, ^30^, ^31^, ^32^, ^33^, ^34^, ^35^, ^36^, ^37^, ^38^ ^]^ Recent advances have demonstrated their efficient transformation through photoredox catalysis,^[^ ^34^, ^35^, ^36^, ^37^ ^]^ electrochemical methods,^[^ ^38^ ^]^ and metal‐catalyzed approaches.^[^ ^39^ ^]^ The synthetic access to these motifs has also progressed in recent years,^[^ ^25^, ^40^ ^]^ including via hydrogermylations of olefins, which is typically catalyzed by a metal or Lewis acid.^[^ ^41^, ^42^, ^43^, ^44^, ^45^, ^46^, ^47^ ^]^ However, the concept of remote functionalization has not been realized for germylations (Figure 1).

**Figure 1:**
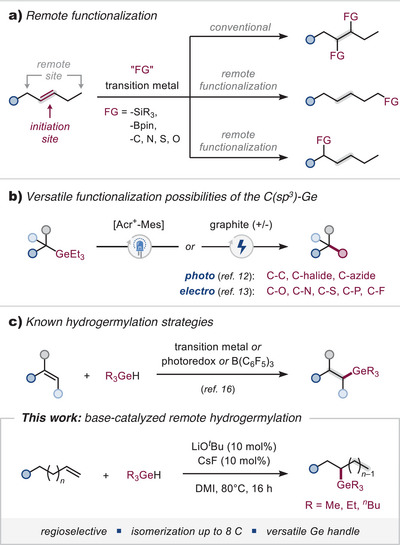
a) Remote functionalization of alkenes. b) Functionalization of C(sp^3^)‐Ge. c) State‐of‐the‐art for hydrogermylation of alkenes. d) This work: base catalyzed remote site‐selective hydrogermylation.

This report discloses the first remote germylation, which proceeds via a rare, base‐catalyzed olefin migration‐free of any added transition metal catalyst. The terminating germylation is triggered by an attack of a germylanion.

Given the exceptional stability of germanes under basic conditions,^[^ ^25^, ^26^, ^34^, ^35^, ^36^, ^37^, ^38^, ^48^ ^]^ we sought to determine whether remote germylation of olefins could be achieved under similar conditions. To investigate this, we selected 4‐phenyl‐1‐butene as a model substrate and systematically examined different bases in combination with various germylating reagents (Figure 2).

**Figure 2:**
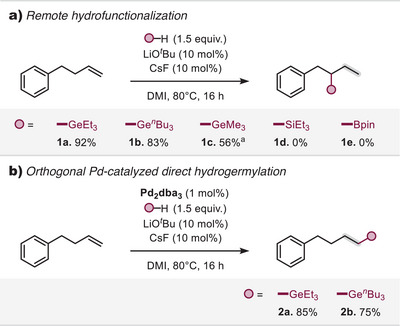
a) Remote functionalization of alkenes using different hyrdofunctionalization reagents. b) Orthogonal direct hydrogermylation under Pd‐catalysis. ^a)^Ge_2_Me_6_ was used as germylation reagent.

Through these studies, we discovered that employing 2.5 equivalents of a *tert*‐butoxide base alongside Et_3_Ge‐SiMe_2_Ph^[^ ^49^ ^]^ or Me_3_Ge‐GeMe_3_ effectively yielded the desired germylated product (**1a**). Notably, replacing the digermane or [Ge]–[Si] reagents with Et_3_GeH resulted in the same product, making this approach particularly attractive as it ensures complete incorporation of the germanium reagent into the final product. Traditional hydrogermylations of olefins have historically required either transition metal catalysis or Lewis acid activation. In contrast, the transformation described here proceeds exclusively under base‐mediated conditions.^[^ ^50^ ^]^

Encouraged by these results, we set out to explore the limits of this transformation and systematically varied the amount of added base. Notably, we discovered that a stoichiometric quantity of base was not required (see Supporting Information for details) and that the transformation was most efficient when the counterion of the *tert*‐butoxide base was modified to CsO*^t^* Bu (in DMI at 80 °C). This enhanced reactivity was achieved through an in situ salt metathesis reaction between LiO*^t^* Bu and CsF.^[^ ^51^, ^52^ ^]^ As such, 4‐phenyl‐1‐butene was successfully transformed to alkyl germane **1a** in an overnight reaction at 80 °C using 10 mol% of in situ generated CsO*^t^* Bu in DMI. Formally, a double‐bond migration had taken place in this substrate, followed by hydrogermylation.

Given that well‐defined transition metal catalysts are known to facilitate double‐bond isomerization,^[^ ^21^, ^53^, ^54^, ^55^, ^56^, ^57^ ^]^ we conducted a trace‐metal (ICP‐MS) analysis on the reagents used in our study to rigorously assess the presence of trace metal contaminants (for detailed results, see Supporting Information). ICP‐MS analysis revealed that the commercially available Et_3_GeH contained approximately 0.1 ppm of palladium, whereas *^n^* Bu_3_GeH was essentially free of Pd, i.e., below the limit of detection (<20 ppb). Despite this difference, *^n^* Bu₃GeH proved equally effective in the transformation, affording the desired germylated product **1b** in a high yield of 83%. In contrast, well‐established hydrofunctionalization reagents such as Et_3_SiH and HBpin showed no reactivity under these conditions, highlighting the distinct reactivity of alkyl germane reagents in this transformation.^[^ ^58^ ^]^ To further explore the potential involvement of palladium, we examined the effect of an added Pd(0) catalyst (Pd_2_dba_3_, 1.0 mol%) on our reaction. Under these conditions, the reaction proceeded via a conventional hydrogermylation pathway, selectively yielding the terminal hydrogermylated anti‐Markovnikov products (**2a**, **2b**) in high yield instead of the remote functionalization product. This finding underscores the fundamentally different reaction mechanisms operating in the base‐catalyzed versus Pd‐catalyzed process.

We next tested the generality and scope of the base‐catalyzed remote germylation of alkenes (Scheme 1). Expanding the explorations with allylbenzene derivatives enabled the selective germylation of a wide range of functionalized substrates, including natural products such as estragole, methyl eugenol, and safrole (**3**–**5**). This transformation exhibited broad functional group tolerance, successfully accommodating electron‐donating substituents at the ortho, meta, and para positions (**8**–**10**), as well as aryl halides (Ar─F and ─Cl; **11**–**12**) and tertiary amines (**14**), all yielding high conversion to product. Notably, when a substrate containing both an allyl group and an allyl ether was subjected to the reaction, exclusive hydrogermylation of the allyl group was observed, whereas the allyl ether underwent selective isomerization to the *cis*‐allyl ether configuration (**15**).^[^ ^59^ ^]^ Furthermore, allyl‐functionalized heterocycles, including benzothiophene (**16**) and thiophene (**17**), proved to be compatible, affording high yields of the desired products.

**Scheme 1:**
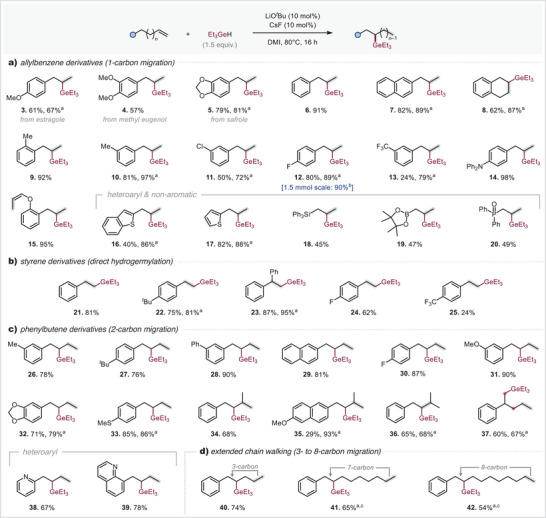
Scope of the remote hydrogermylation reaction. Reaction conditions: LiO*^t^* Bu (10 mol%), CsF (10 mol%), anhydrous DMI (0.1 M), alkene (1.0 equiv), Et_3_GeH (1.5 equiv), 80 °C, 16 h. The reaction was set up in a glovebox on 0.3 mmol scale. Isolated yields. ^a)^2.0 equiv of LiO*^t^* Bu and CsF were used. ^b)^Yield was determined by ^19^F NMR (using 1,4‐difluorobenzene as an internal standard). ^c)^Reaction was run at 120 °C for 72 h.

Importantly, akin to transition metal–catalyzed remote functionalization of alkenes,^[^ ^8^, ^9^, ^10^, ^11^, ^12^ ^]^ we successfully extended this methodology to nonaromatic allyl substrates, demonstrating that they serve as viable candidates for remote hydrogermylation. This approach facilitated the efficient incorporation of diverse functional groups, yielding triphenylsilane (**18**), alkyl‐Bpin (**19**), and diphenylphosphine oxide (**20**) derivatives in good yields, further underscoring the versatility and synthetic potential of this transformation.

Simple styrenes and their derivatives were also successfully hydrogermylated under these conditions on the β position, tolerating substituents like *tert*‐butyl, fluorine, and trifluoromethyl groups (**21**–**25**).

Next, we investigated the limits of possible distance for the remote functionalization and systematically varied the chain length. Phenylbutene derivatives, which necessitate a two‐carbon chain walk, were found to be equally effective under these conditions. A diverse array of aromatic substituents was well tolerated, affording high yields of the corresponding products (**26**–**33**). When branched olefins were employed, the reaction outcome was unaffected by the position of the double bond, proceeding efficiently regardless of whether the olefin was terminal (**34**–**35**) or internal (**36**), yielding the desired products in good yields. For substrates featuring two potential β positions for hydrogermylation, regioselectivity favored the sterically less hindered site, with the alternative position undergoing only trace‐level conversion (**37**). Moreover, pharmaceutically relevant heterocyclic scaffolds, including pyridine^[^ ^60^ ^]^ (**38**) and quinoline (**39**) derivatives, were successfully hydrogermylated at the remote position using catalytic amounts of base, delivering the target products in high yields. This highlights the broad applicability of this methodology in the synthesis of complex bioactive molecules.

Given that base‐catalyzed isomerization reported in the literature is largely restricted to migrations over two carbon bonds,^[^ ^22^, ^61^ ^]^ we sought to evaluate the feasibility of extending this transformation to longer alkyl chains under our catalytic protocol. We found that the remote hydrogermylation of 5‐phenyl‐1‐pentene proceeded efficiently under catalytic conditions, affording the desired product (**40**) in high yield. Extending the alkyl chain length further required elevated temperatures (120 °C) and stoichiometric base, however, to ensure selective chain‐walking toward the remote functionalization site. This strategy enabled the successful isolation of remote hydrogermylated products following isomerization across seven (**41**) and eight (**42**) double bonds, delivering satisfying yields of 65% and 54%, respectively.

The versatility of this protocol is further exemplified by the site‐selective formation of a single high‐yielding product (**1a**) from 1:1:1 mixture of different olefin isomers (Scheme 2a). These constitutional and stereoisomers were efficiently funneled into the same desired product. Given that alkenes are widely available as abundant feedstock materials, this approach eliminates the need for prior separation of isomeric mixtures, streamlining synthetic workflows and enhancing the practicality of remote hydrogermylation in complex molecular settings.

**Scheme 2:**
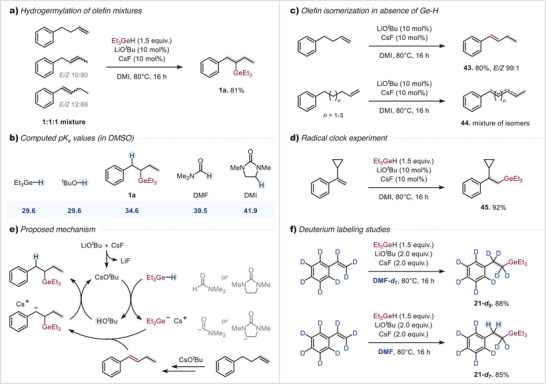
Mechanistic studies. Reaction conditions: LiO*^t^* Bu (10 mol%), CsF (10 mol%), anhydrous DMI (0.1 M), alkene mixture (0.3 mmol, 1.0 equiv), Et_3_GeH (1.5 equiv), 80 °C, 16 h. Computed p*K_a_* values (in DMSO) were obtained using CosmothermX based on DFT‐optimized minimum energy conformers (for details see the Supporting Information).

Mechanistically, as the same product is formed regardless if styrene or substrate with more distal olefin was employed, there is likely an initial olefin migration, followed by germylation. Our calculations of p*K_a_* values (Scheme 2b) indicated that Et_3_GeH and *^t^* BuOH have very similar acidities, which implies that the in situ formed CsO*^t^* Bu should be—at minimum to some extent—capable of Et_3_GeH deprotonation to the corresponding Et_3_Ge‐anion. Both, *tert*‐butoxide and the germylanion could therefore be involved in the olefin migration from the terminal olefin to the thermodynamically more stable internal olefin.^[^ ^62^ ^]^ Although our tests indicated that the olefin migration was complete and selective to deliver substrate **43** (Scheme 2c), the longer chain examples, i.e., migration over 3‐ to 5‐carbon atoms delivered a mixture of positional alkene isomers (**44**) even with stoichiometric amounts of base and elevated temperature. As such, the germylation is critical in these cases to deliver the single product (e.g., **40**) selectively.^[^ ^63^ ^]^ As germylation was also observed in the absence of cyclopropane opening to give **45** (Scheme 2d) and radical intermediates are therefore unlikely,^[^ ^64^ ^]^ a germyl anion likely attacks the in situ formed styrenyl derivative as to give a benzylic anion (see Scheme 2e for the proposed mechanism).^[^ ^65^, ^66^ ^]^ The, thereby, formed benzyl anion is calculated to be slightly more basic than the employed base (calculated p*K_a_* of 34.6) and could hence either deprotonate *^t^* BuOH or Et_3_GeH to regenerate the required base‐catalyst for olefin migration. On the other hand, Taillefer et al. suggest that KO*^t^* Bu can also deprotonate DMF at elevated temperatures,^[^ ^67^ ^]^ and the proton exchanges therefore likely extend to the solvent also. In line with this, our deuterium labelling studies (Scheme 2f) clearly indicate that under the employed reaction conditions, the benzylic protons/deuterons are readily exchanged with the solvent.

In summary, this work discloses the first remote hydrogermylation of alkenes, achieved through a rare base‐catalyzed strategy. The methodology stands out for its operational simplicity and broad scope, enabling seamless functionalization irrespective of initial olefin positioning and achieving up to 8‐carbon chain walks, far surpassing the previous two‐carbon limit of base‐mediated processes. Beyond Et_3_Ge, both Me_3_Ge and *^n^* Bu_3_Ge were successfully incorporated at remote positions. The key lies in the meticulously balanced p*K_a_* values between the employed base CsO*^t^* Bu and R_3_GeH, driving the reaction through a strategically controlled Ge‐anion attack.

## Conflict of Interests

The authors declare no conflict of interest.

## Supporting information



Supporting Information

## Data Availability

The data that support the findings of this study are available in the Supporting Information of this article.
