# Supplementary material for: Base‐Catalyzed Remote Hydrogermylation of Olefins
Source: Angew Chem Int Ed Engl. 2025 Mar 26;64(22):e202503573. doi: 10.1002/anie.202503573 (PMC12105699; doi:10.1002/anie.202503573)
Supplement: Supplementary file 1 — Supporting Information [file ANIE-64-e202503573-s001.pdf]

Contents

1. General Experimental Details S3

2. Initial Results and Optimization of Reaction Conditions S5

2.1 Initial Results Using $Et_3GeSiMe_2Ph$ S5

2.2 Initial Results Using Different Germylation Reagents S6

2.3 Optimization of Reaction Conditions S7

3. Base-Catayzed Remote Site-Selective Hydrogermylation of alkenes S10

3.1 General Procedure A S10

3.2 General Procedure B S10

3.3 Characterization Data of the Products S10

3.4 Different Hydrofunctionalization Reagents S24

4. Starting Material Synthesis S26

4.1 Synthesis of Olefins S26

4.2 General Procedure C S26

4.3 Characterization Data of Alkene Starting Materials S26

5. Mechanistic Studies S29

5.1 Trace Metal Analysis (ICP-MS) S29

5.2 Control Experiments S30

5.2.1 Presence of metals S30

5.2.2 Absence of germanium hydride S30

5.2.3 Orthogonal terminal hydrogermylation under Palladium catalysis S31

5.3 Radical Clock Experiment S32

5.4 Deuterium Labeling Studies S33

6. Computed pK_a Values S34

6.1 XYZ Coordinates and Energies of Optimized Structures S34

7. NMR Spectra S38

7.1 Products S38

7.2 Starting materials S90

8. References S96

1. General Experimental Details

Techniques

All reactions were performed utilizing standard Schlenk techniques under an Argon atmosphere or inside a glovebox unless otherwise stated. Glassware and magnetic stir bars were dried in an oven (110 °C) for at least 24 hours prior to use. Unless otherwise stated, experiments were carried out at room temperature (25 ± 2 °C). The removal of solvents in vacuo was achieved using a rotary evaporator (bath temperatures up to 40 °C) at a pressure of 20 mmHg (diaphragm pump), or at 0.1 mmHg (oil pump) on a vacuum line at room temperature.

Solvents, reagents and starting materials

Unless otherwise stated, all anhydrous solvents were purchased from Sigma Aldrich. Anhydrous 1,3-dimethyl-2-imidazolidinone (DMI, absolute, over molecular sieve (H_2O ≤0.04%), ≥99.5% (GC)) was purchased from Sigma Aldrich. *Note: DMI with lower purity than 90% and higher contamination of H_2O leads to low conversion.* LiO^{t}Bu (99.99% trace metal basis) was purchased from Alfa Aesar and CsF (99.9%) was purchased from chemPUR. THF and Et_2O were dried using a PS-MD-5 solvent purification system from Innovative Technology. Technical grade solvents were distilled prior to use for chromatography and extraction.

Unless otherwise stated, all reagents and starting materials were commercially available and used as received.

Purification

Thin layer chromatography (TLC) was performed on Macherey Nagel ALUGRAM Xtra SIL G UV254 aluminium plates with unmodified silica and visualized either under UV light or stained with iodine or $KMnO_4$. Flash silica gel column chromatography was performed with silica gel (0.04 – 0.063 mm particle size) purchased from Macherey Nagel. Preparative HPLC was performed on a Knauer Azura HPLC (employing UV detector 2600, at 254 and 230 nm) using a LiChrosorb Si60 column (Merck, 250 x 25 mm, 7 µm silica poriosity).

Characterization

All ^{1}H, ^{13}C and ^{19}F NMR spectra were recorded on Bruker Avance Neo 600, Varian VNMRS 600 or Varian VNMRS 400 or Bruker Avance Neo 400 spectrometers at ambient temperature (unless otherwise specified). Chemical shifts (δ) are reported in parts per million (ppm) and were referenced either to residual solvent peak (for ^{1}H and ^{13}C spectra) or internally by the instrument after locking and shimming to the deuterated solvent (for ^{19}F). Coupling constants (J) are given in Hertz (Hz). Multiplicities of signals in ^{1}H, ^{19}F, and ^{13}C NMR were designated as s (singlet), d (doublet), dd (doublet of doublets), dt (doublet of triplets), ddd (doublet of doublets of doublets), t (triplet), td (triplet of doublets), q (quartet), p (quintet), h (sextet), hept (septet), and m (multiplet).

Gas chromatography coupled with mass spectrometry (GC-MS) was performed on an Agilent Technologies 5975 series MSD mass spectrometer under electrospray ionization (EI) mode coupled with an Agilent Technologies 7820A gas chromatograph employing an Agilent 19091s-433 HP-5MS column (30 m x 0.250 μm x 0.250 μm). High-resolution mass spectrometry (HRMS) was performed using a Thermo Scientific LTQ Orbitrap XL spectrometer (ESI), Finnigan MAT 95 (EI) or Bruker Maxis II LC-MS-System (APCI). Low-resolution masses of known compounds were extracted from their GC-MS chromatograms. IR spectra were recorded on a Spectrum 100 spectrometer with a UATR Diamond/KRS-5 crystal with attenuated total reflectance (ATR).

ICP-MS analysis was conducted on an Agilent 8900 Triple Quadrupole (ICP QQQ) at the RWTH Uniklinik Aachen.

2. Initial Results and Optimization of Reaction Conditions

2.1 Initial Results Using $Et_3GeSiMe_2Ph$

In an argon-filled glovebox, a 4 mL screw-capped glass vial equipped with a magnetic stir bar was charged with a base (3.0 equiv.) and dissolved in anhydrous DMI (1.0 mL, 0.1 M). To the mixture were added 1-allyl-3-methylbenzene (13.2 mg, 0.1 mmol, 1.0 equiv.) and $Et_3GeSiMe_2Ph$ (73.8 mg, 2.5 equiv.). The vial was sealed and brought out of glovebox and stirred in a heating block at 80°C over night. After cooling down to room temperature a small aliquot was taken and analyzed by GC-MS.

Table S1. Initial results using different bases.

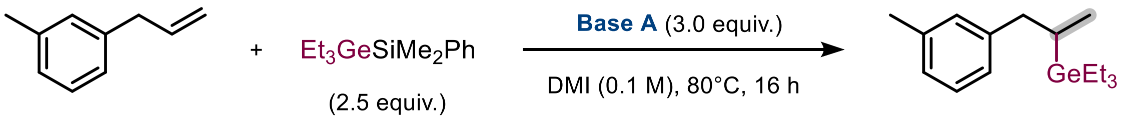

Entry	Base A	Yield[a] [%]
1	LiO^{t}Bu	95
2	NaO^{t}Bu	15
3	KO^{t}Bu	23
4	NaOMe	15
5	LiO^{i}Pr	41

[a] based on conversion determined by qualitative GC-MS analysis.

Table S2. Initial results using different solvents.

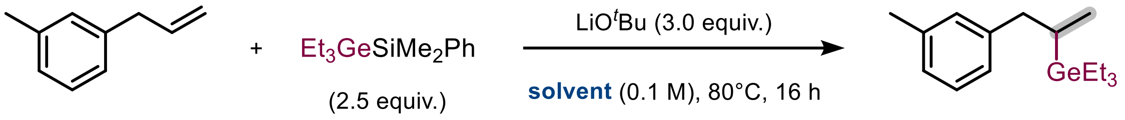

Entry	Solvent	Yield[a] [%]
1	DMF	14
2	DMSO	50
3	THF	0
4	Acetone	0
5	HFIP	0

[a] based on conversion determined by qualitative GC-MS analysis.

2.2 Initial Results Using Different Germylation Reagents

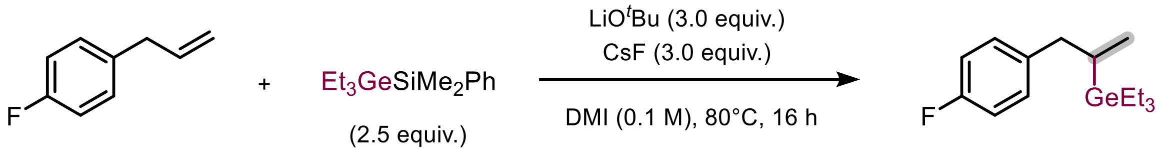

12. 93%

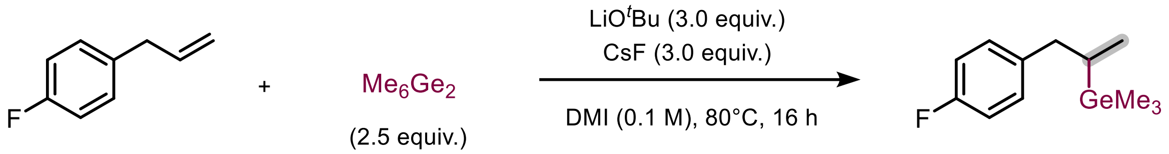

12. 71%

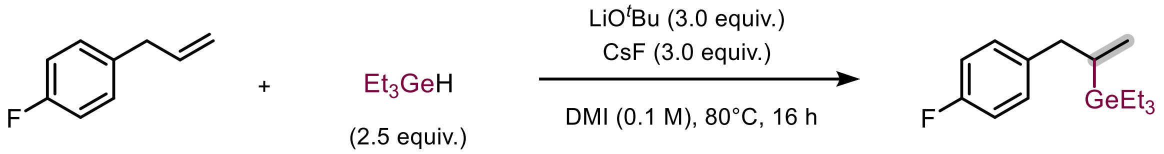

12. 97%

In an argon-filled glovebox, an 8 mL screw-capped glass vial equipped with a magnetic stir bar was charged with LiO^{t}Bu (3.0 equiv.) and CsF (3.0 equiv.) and dissolved in anhydrous DMI (3.0 mL, 0.1 M). To the mixture were added 1-allyl-4-fluorobenzene (13.6 mg, 0.30 mmol, 1.0 equiv.) and the germanium reagent (0.75 mmol, 2.5 equiv.). The vial was sealed and brought out of glovebox and stirred in a heating block at 80°C over night. After cooling down to room temperature the crude reaction mixture was directly subjected to flash column chromatography on silica gel to obtain the desired product. Characterization data match with compound **12**, obtained using the standard conditions (general procedure A, see chapter 3.1).

2.3 Optimization of Reaction Conditions

All reaction optimization and sreening reactions were carried out using the following general procedure:

In an argon-filled glovebox, a 4 mL screw-capped glass vial equipped with a magnetic stir bar was charged with LiO^{t}Bu (0.1-2.0 equiv.) and CsF (0.1-2.0 equiv.) and dissolved in anhydrous DMI (1.0 mL, 0.1 M). To the mixture were added 1-(but-3-en-1-yl)-4-fluorobenzene (15.0 mg, 0.1 mmol, 1.0 equiv.) and Et_3GeH (24.3 µL, 1.5 equiv.). The vial was sealed and brought out of glovebox and stirred in a heating block at 80°C over night. After cooling down to room temperature 1,4-difluorobenzene was added as internal standard for ^{19}F NMR yield determination. Additionally, a small aliquot was taken and analyzed by GC-MS.

Table S3. Screening of bases.

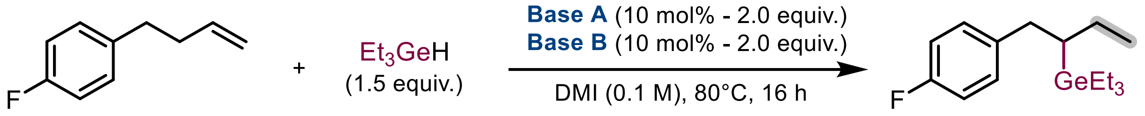

Entry	Base A	Base B	Base loading	^{19}F NMR Yield [%]
1	LiO^{t}Bu	CsF	2.0 equiv.	91
2	NaO^{t}Bu	CsF	2.0 equiv.	18
3	KO^{t}Bu	CsF	2.0 equiv.	56
4	LiOMe	CsF	2.0 equiv.	2
5	LiO^{t}Bu	LiF	2.0 equiv.	0
6	LiO^{t}Bu	NaF	2.0 equiv.	0
7	LiO^{t}Bu	KF	2.0 equiv.	14
8	LiO^{t}Bu	RbF	2.0 equiv.	12
9	KO^{t}Bu	KF	2.0 equiv.	81
10	LiO^{t}Bu	CsF	10 mol%	91
11	LiO^{t}Bu	-	10 mol%	0
12	NaO^{t}Bu	-	10 mol%	0
13	KO^{t}Bu	-	10 mol%	87

Table S4. Solvent screening.

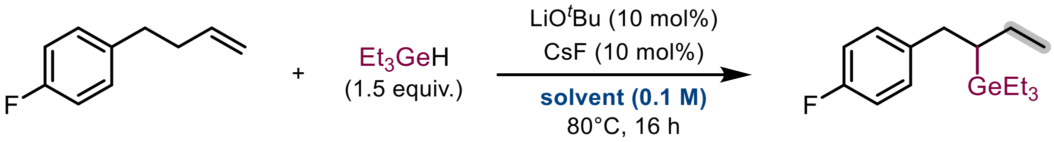

Entry	Solvent	^{19}F NMR Yield [%]
1	DMI	91
2	NMP	0
3	DMF	45
4	DMAc	13
5	THF	0
6	ACN	0
7	DMSO	66
8	Benzene	0
9	Cyclohexane	0

Table S5. Solvent concentration optimization.

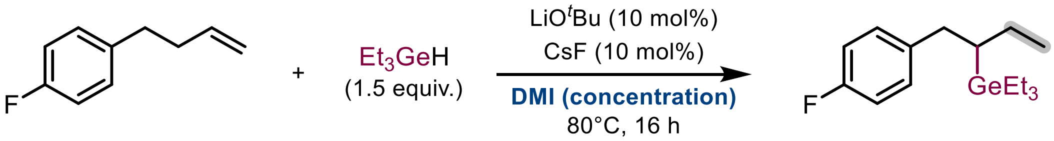

Entry	Concentration [M]	^{19}F NMR Yield [%]
1	0.4	67
2	0.2	84
3	0.1	91
4	0.05	80

Table S4. Reaction temperature optimization.

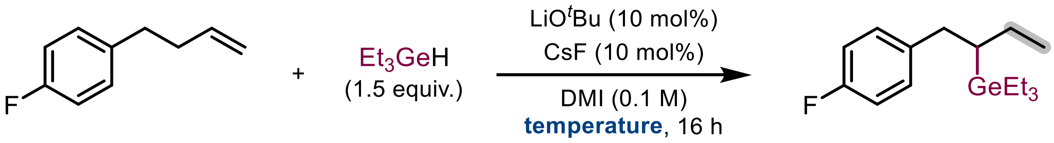

Entry	Temperature (°C)	^{19}F NMR Yield (%)
1	Room temperature	0
2	40	0
3	60	6
4	80	91
5	100	89
6	120	86

Table S5. Reaction time optimization.

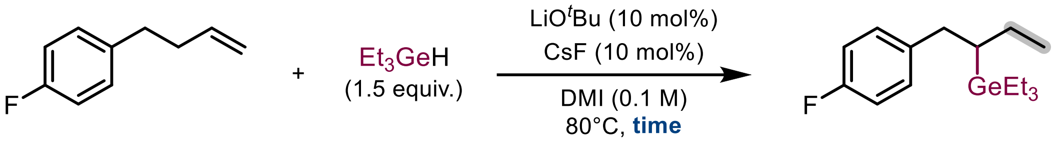

Entry	Time (h)	^{19}F NMR Yield (%)
1	2	0
2	4	19
3	6	29
4	8	56
5	16	91

3. Base-Catayzed Remote Site-Selective Hydrogermylation of alkenes

3.1 General Procedure A

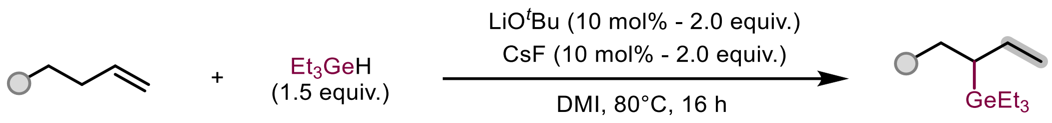

In an argon-filled glovebox, an 8 mL screw-capped glass vial equipped with a magnetic stir bar was charged with LiO*t*Bu (10 mol% – 2.0 equiv.) and CsF (10 mol% – 2.0 equiv.) and dissolved in anhydrous DMI (1.0 mL, 0.1 M). To the mixture were added the alkene (0.10 – 0.30 mmol, 1.0 equiv.) and Et_3GeH (0.15 – 0.45 mmol, 1.5 equiv.). The vial was sealed and brought out of glovebox and stirred in a heating block at 80°C over night. After cooling down to room temperature the crude reaction mixture was directly subjected to flash column chromatography on silica gel to obtain the desired product.

3.2 General Procedure B

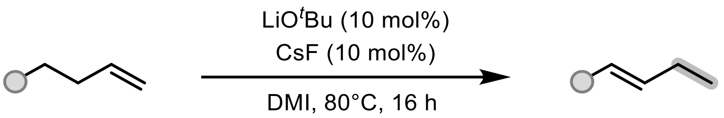

In an argon-filled glovebox, an 8 mL screw-capped glass vial equipped with a magnetic stir bar was charged with LiO*t*Bu (10 mol%) and CsF (10 mol%) and dissolved in anhydrous DMI (1.0 mL, 0.1 M) and to the mixture was added the alkene (0.10 - 0.30 mmol, 1.0 equiv.). The vial was sealed and brought out of glovebox and stirred in a heating block at 80°C over night. After cooling down to room temperature the crude reaction mixture was directly subjected to flash column chromatography on silica gel to obtain the desired product.

3.3 Characterization Data of the Products

Triethyl(1-phenylbutan-2-yl)germane (1a)

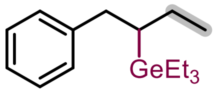

Prepared, following the general procedure A from but-3-en-1-ylbenzene (39.7 mg, 0.30 mmol) with LiO*t*Bu (2.4 mg, 0.03 mmol, 0.1 equiv.) and CsF (4.6 mg, 0.03 mmol, 0.1 equiv.). The title product was obtained after purification by flash chromatography on silica gel (*n*-pentane) as colorless oil (80.0 mg, 0.27 mmol, 92%). Using stoichiometric amounts of bases, LiO*t*Bu (48.0 mg, 0.60 mmol, 2.0 equiv.) and CsF (91.1 mg, 0.60 mmol, 2.0 equiv.), resulted in a similar product yield (81.1 mg, 0.28 mmol, 95%).

Prepared, following general procedure A from a mixture of olefins in a 1:1:1 ratio (39.7 mg, 0.30 mmol) with LiO*t*Bu (2.4 mg, 0.03 mmol, 0.1 equiv.) and CsF (4.6 mg, 0.03 mmol, 0.1 equiv.). The title product

was obtained after purification by flash chromatography on silica gel (*n*-pentane) as colorless oil (71.3 mg, 0.24 mmol, 81%).

R_f = 0. (*n*-pentane). **^{1}H NMR** (600 MHz, $CDCl_3$) δ/ppm = 7.30 – 7.25 (m, 2H), 7.21 – 7.15 (m, 3H), 2.80 (dd, *J* = 13.9, 4.9 Hz, 1H), 2.63 (dd, *J* = 13.6, 10.3 Hz, 1H), 1.51 – 1.38 (m, 3H), 1.05 (t, *J* = 7.9 Hz, 9H), 0.88 (t, *J* = 7.3 Hz, 3H), 0.76 (q, *J* = 7.8 Hz, 6H). **^{13}C NMR** (151 MHz, $CDCl_3$) δ/ppm = 143.2, 129.0, 128.2, 125.7, 37.1, 29.3, 23.3, 13.7, 9.4, 3.9. **HRMS** (ESI) calculated for $C_{14}H_{23}Ge$: 265.1006 $[M-CH_2CH_3]^+$, found: 265.0999. **IR** (neat): ν/ cm^{-1} = 3026, 2951, 2871, 1456, 1428.

Triethyl(1-(4-methoxyphenyl)propan-2-yl)germane (3)

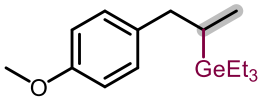

Prepared, following the general procedure A from 4-allylanisole (44.5 mg, 0.30 mmol) with LiO*t*Bu (2.4 mg, 0.03 mmol, 0.1 equiv.) and CsF (4.6 mg, 0.03 mmol, 0.1 equiv.). The title product was obtained after purification by flash chromatography on silica gel (*n*-pentane/Et_2O 98:2) as a colorless oil (56.5 mg, 0.18 mmol, 61%). Using stoichiometric amounts of bases, LiO*t*Bu (48.0 mg, 0.60 mmol, 2.0 equiv.) and CsF (91.1 mg, 0.60 mmol, 2.0 equiv.), resulted in similar product yield (62.0 mg, 0.20 mmol, 67%).

R_f = 0.68 (*n*-pentane). **^{1}H NMR** (600 MHz, $CDCl_3$) δ/ppm = 7.07 (d, *J* = 8.5 Hz, 2H), 6.82 (d, *J* = 8.6 Hz, 2H), 3.79 (s, 3H), 2.84 (dd, *J* = 13.8, 4.3 Hz, 1H), 2.36 – 2.30 (m, 1H), 1.38 – 1.30 (m, 1H), 1.05 (t, *J* = 7.9 Hz, 9H), 0.93 (d, *J* = 7.4 Hz, 3H), 0.76 (q, *J* = 8.0 Hz, 6H). **^{13}C NMR** (151 MHz, $CDCl_3$) δ/ppm = 157.8, 134.9, 129.8, 113.7, 55.4, 38.9, 21.5, 15.5, 9.4, 2.9. **HRMS** (APCI) calculated for $C_{14}H_{23}GeO$: 281.0955 $[M-CH_2CH_3]^+$, found: 281.0951. **IR** (neat): ν/ cm^{-1} = 2946, 2906, 2870, 1610, 1509, 1245.

(1-(3,4-Dimethoxyphenyl)propan-2-yl)triethylgermane (4)

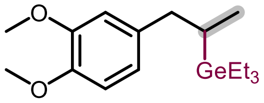

Prepared, following the general procedure A from methyl eugenol (53.5 mg, 0.30 mmol) with LiO*t*Bu (2.4 mg, 0.03 mmol, 0.1 equiv.) and CsF (4.6 mg, 0.03 mmol, 0.1 equiv.). The title product was obtained after purification by flash chromatography on silica gel (*n*-pentane/Et_2O 94:6) as a colorless oil (57.6 mg, 0.17 mmol, 57%).

R_f = 0.22 (*n*-pentane). **^{1}H NMR** (600 MHz, $CDCl_3$) δ/ppm = 6.79 (d, *J* = 8.0 Hz, 1H), 6.71 – 6.66 (m, 2H), 3.88 (s, 3H), 3.86 (s, 3H), 2.84 (dd, *J* = 13.8, 4.5 Hz, 1H), 2.38 – 2.32 (m, 1H), 1.40 – 1.32 (m, 1H), 1.05 (t, *J* = 7.9 Hz, 9H), 0.95 (d, *J* = 7.4 Hz, 3H), 0.76 (q, *J* = 7.6 Hz, 6H). **^{13}C NMR** (151 MHz, $CDCl_3$) δ/ppm = 148.8, 147.2, 135.5, 120.8, 112.2, 111.2, 56.1, 56.0, 39.5, 21.5, 15.6, 9.4, 2.9. **HRMS** (ESI) calculated for $C_{17}H_{30}O_2GeNa$: 363.1350 $[M+Na]^+$, found: 363.1346. **IR** (neat): ν/ cm^{-1} = 2945, 2870, 1590, 1458, 1261, 1026.

(1-(Benzo[*d*][1,3]dioxol-5-yl)propan-2-yl)triethylgermane (5)

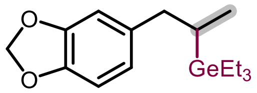

Prepared, following the general procedure A from safrole (48.6 mg, 0.30 mmol) with LiO*t*Bu (2.4 mg, 0.03 mmol, 0.1 equiv.) and CsF (4.6 mg, 0.03 mmol, 0.1 equiv.). The title product was obtained after purification by flash chromatography on silica gel (*n*-pentane/Et_2O 94:6) as a colorless oil (76.1 mg, 0.24 mmol, 79%). Using stoichiometric amounts of bases,

LiO^{t}Bu (48.0 mg, 0.60 mmol, 2.0 equiv.) and CsF (91.1 mg, 0.60 mmol, 2.0 equiv.), resulted in the same product yield (78.5 mg, 0.24 mmol, 81%).

R$_f$ = 0.34 (*n*-pentane). **^{1}H NMR** (600 MHz, $CDCl_3$) δ/ppm = 6.72 (d, *J* = 7.9 Hz, 1H), 6.65 (s, 1H), 6.60 (d, *J* = 7.8 Hz, 1H), 5.92 (s, 2H), 2.82 (dd, *J* = 13.7, 4.3 Hz, 1H), 2.34 – 2.28 (m, 1H), 1.37 – 1.29 (m, 1H), 1.05 (t, *J* = 7.9 Hz, 9H), 0.93 (d, *J* = 7.4 Hz, 3H), 0.76 (q, *J* = 7.9 Hz, 6H). **^{13}C NMR** (151 MHz, $CDCl_3$) δ/ppm = 147.6, 145.6, 136.8, 121.7, 109.2, 108.0, 100.8, 39.6, 21.6, 15.4, 9.3, 2.9. **HRMS** (APCI) calculated for $C_{14}H_{21}GeO_2$: 295.0748 $[M\text{-}CH_2CH_3]^+$, found: 295.0744. **IR** (neat): ν/ cm^{-1} = 2947, 2871, 1608, 1244, 1039.

Triethyl(1-phenylpropan-2-yl)germane (6)

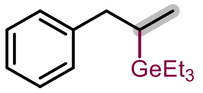

Prepared, following the general procedure A from allylbenzene (35.5 mg, 0.30 mmol) with LiO^{t}Bu (2.4 mg, 0.03 mmol, 0.1 equiv.) and CsF (4.6 mg, 0.03 mmol, 0.1 equiv.). The title product was obtained after purification by flash chromatography on silica gel (*n*-pentane) as a colorless oil (75.9 mg, 0.27 mmol, 91%).

R$_f$ = 0.87 (*n*-pentane). **^{1}H NMR** (600 MHz, $CDCl_3$) δ/ppm = 7.29 – 7.26 (m, 2H), 7.19 – 7.14 (m, 3H), 2.90 (dd, *J* = 13.7, 4.2 Hz, 1H), 2.43 – 2.36 (m, 1H), 1.44 – 1.36 (m, 1H), 1.06 (t, *J* = 7.9 Hz, 9H), 0.94 (d, *J* = 7.4 Hz, 3H), 0.77 (q, *J* = 8.1 Hz, 6H). **^{13}C NMR** (151 MHz, $CDCl_3$) δ/ppm = 142.8, 129.0, 128.2, 125.7, 39.8, 21.4, 15.5, 9.3, 2.9. **HRMS** (ESI) calculated for $C_{13}H_{21}Ge$: 251.0850 $[M\text{-}CH_2CH_3]^+$, found: 251.0852. **IR** (neat): ν/ cm^{-1} =2947, 2870, 1456, 1015.

Triethyl(1-(naphthalen-2-yl)propan-2-yl)germane (7)

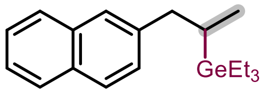

Prepared, following the general procedure A from 2-allylnaphthalene (50.5 mg, 0.30 mmol) with LiO^{t}Bu (2.4 mg, 0.03 mmol, 0.1 equiv.) and CsF (4.6 mg, 0.03 mmol, 0.1 equiv.). The title product was obtained after purification by flash chromatography on silica gel (*n*-pentane) as a colorless oil (80.8 mg, 0.25 mmol, 82%). Using stoichiometric amounts of bases, LiO^{t}Bu (48.0 mg, 0.60 mmol, 2.0 equiv.) and CsF (91.1 mg, 0.60 mmol, 2.0 equiv.), resulted in a similar product yield (87.6 mg, 0.27 mmol, 89%).

R$_f$ = 0.89 (*n*-pentane). **^{1}H NMR** (600 MHz, $CDCl_3$) δ/ppm = 7.82 – 7.74 (m, 3H), 7.58 (d, *J* = 1.6 Hz, 1H), 7.44 (ddd, *J* = 8.2, 6.8, 1.5 Hz, 1H), 7.41 (ddd, *J* = 8.1, 6.8, 1.4 Hz, 1H), 7.32 (dd, *J* = 8.3, 1.8 Hz, 1H), 3.07 (dd, *J* = 13.7, 4.1 Hz, 1H), 2.58 – 2.50 (m, 1H), 1.54 – 1.46 (m, 1H), 1.08 (t, *J* = 7.9 Hz, 9H), 0.96 (d, *J* = 7.4 Hz, 3H), 0.80 (q, *J* = 7.9 Hz, 6H). **^{13}C NMR** (151 MHz, $CDCl_3$) δ/ppm = 140.3, 133.7, 132.1, 127.8, 127.8, 127.7, 127.6, 127.0, 125.9, 125.1, 39.9, 21.2, 15.4, 9.4, 2.9. **HRMS** (APCI) calculated for $C_{17}H_{23}Ge$: 301.1006 $[M\text{-}CH_2CH_3]^+$, found: 301.1002. **IR** (neat): ν/ cm^{-1} = 3052, 2947, 2869, 1906, 1599, 1458.

Triethyl(1,2,3,4-tetrahydronaphthalen-2-yl)germane (8)

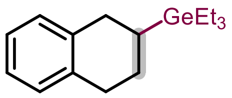

Prepared, following the general procedure A from 1,4-dihydronaphthalene (39.1 mg, 0.30 mmol) with LiO^{t}Bu (2.4 mg, 0.03 mmol, 0.1 equiv.) and CsF (4.6 mg, 0.03 mmol, 0.1 equiv.). The title product was obtained after purification by flash chromatography on silica gel (*n*-pentane) as a colorless oil (54.1 mg, 0.19 mmol, 62%). Using stoichiometric amounts of

bases, LiO^{t}Bu (48.0 mg, 0.60 mmol, 2.0 equiv.) and CsF (91.1 mg, 0.60 mmol, 2.0 equiv.), resulted in higher product yield (76.0 mg, 0.26 mmol, 87%).

R$_f$ = 0.72 (*n*-pentane). **^{1}H NMR** (600 MHz, $CDCl_3$) δ/ppm = 7.10 – 7.02 (m, 4H), 2.85 – 2.72 (m, 4H), 2.03 – 1.96 (m, 1H), 1.70 – 1.61 (m, 1H), 1.43 (tdd, *J* = 12.5, 5.6, 2.7 Hz, 1H), 1.06 (t, *J* = 7.9 Hz, 9H), 0.80 (q, *J* = 7.9 Hz, 6H). **^{13}C NMR** (151 MHz, $CDCl_3$) δ/ppm = 138.1, 137.3, 129.4, 128.9, 125.5, 125.5, 32.2, 30.6, 26.0, 21.6, 9.4, 2.7. **HRMS** (APCI) calculated for $C_{14}H_{21}Ge$: 263.0850 $[M-CH_2CH_3]^+$, found: 263.0849. **IR** (neat): ν/ cm^{-1} = 2910, 2872, 2324, 1796, 1454, 1014.

Triethyl(1-(2-tolyl)propan-2-yl)germane (9)

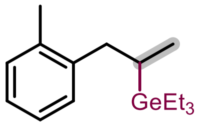

Prepared, following the general procedure A from 1-allyl-2-methylbenzene (39.7 mg, 0.30 mmol) with LiO^{t}Bu (2.4 mg, 0.03 mmol, 0.1 equiv.) and CsF (4.6 mg, 0.03 mmol, 0.1 equiv.). The title product was obtained after purification by flash chromatography on silica gel (*n*-pentane) as a colorless oil (80.9 mg, 0.28 mmol, 92%).

R$_f$ = 0.91 (*n*-pentane). **^{1}H NMR** (600 MHz, $CDCl_3$) δ/ppm = 7.14 – 7.07 (m, 4H), 2.90 (dd, *J* = 13.8, 3.7 Hz, 1H), 2.44 – 2.38 (m, 1H), 2.30 (s, 3H), 1.38 (dqd, *J* = 11.3, 7.3, 3.8 Hz, 1H), 1.07 (t, *J* = 7.9 Hz, 9H), 0.94 (d, *J* = 7.3 Hz, 3H), 0.80 (q, *J* = 8.0 Hz, 6H). **^{13}C NMR** (151 MHz, $CDCl_3$) δ/ppm = 140.7, 136.1, 130.4, 129.9, 125.8, 125.6, 36.7, 19.9, 19.6, 15.4, 9.4, 2.9. **HRMS** (APCI) calculated for $C_{14}H_{23}Ge$: 265.1006 $[M-CH_2CH_3]^+$, found: 265.1005. **IR** (neat): ν/ cm^{-1} =3016, 2947, 1458, 1015.

Triethyl(1-(3-tolyl)propan-2-yl)germane (10)

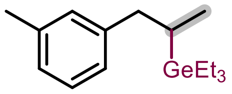

Prepared, following the general procedure A from 1-allyl-3-methylbenzene (39.7 mg, 0.30 mmol) with LiO^{t}Bu (2.4 mg, 0.03 mmol, 0.1 equiv.) and CsF (4.6 mg, 0.03 mmol, 0.1 equiv.). The title product was obtained after purification by flash chromatography on silica gel (*n*-pentane) as a colorless oil (70.3 mg, 0.24 mmol, 81%). Using stoichiometric amounts of bases, LiO^{t}Bu (48.0 mg, 0.60 mmol, 2.0 equiv.) and CsF (91.1 mg, 0.60 mmol, 2.0 equiv.), resulted in higher product yield (85.2 mg, 0.29 mmol, 97%).

R$_f$ = 0.90 (*n*-pentane). **^{1}H NMR** (600 MHz, $CDCl_3$) δ/ppm = 7.18 – 7.13 (m, 1H), 7.00 – 6.94 (m, 3H), 2.86 (dd, *J* = 13.6, 4.1 Hz, 1H), 2.39 – 2.32 (m, 4H), 1.42 – 1.34 (m, 1H), 1.08 – 1.04 (m, 9H), 0.94 (d, *J* = 7.4 Hz, 3H), 0.77 (q, *J* = 8.1 Hz, 6H). **^{13}C NMR** (151 MHz, $CDCl_3$) δ/ppm = 142.8, 137.7, 129.7, 128.1, 126.4, 126.0, 39.7, 21.6, 21.3, 15.5, 9.4, 2.9. **HRMS** (APCI) calculated for $C_{14}H_{23}Ge$: 265.1006 $[M-CH_2CH_3]^+$, found: 265.1005. **IR** (neat): ν/ cm^{-1} = 3052, 2947, 2870, 1601, 1458, 1232.

(1-(3-Chlorophenyl)propan-2-yl)triethylgermane (11)

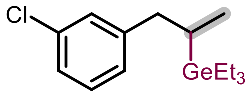

Prepared, following the general procedure A from 1-allyl-3-chlorobenzene (45.8 mg, 0.30 mmol) with LiO^{t}Bu (2.4 mg, 0.03 mmol, 0.1 equiv.) and CsF (4.6 mg, 0.03 mmol, 0.1 equiv.). The title product was obtained after purification by flash chromatography on silica gel (*n*-pentane) as a colorless oil (46.6 mg, 0.15 mmol, 50%). Using stoichiometric amounts of bases, LiO^{t}Bu (48.0 mg, 0.60 mmol, 2.0 equiv.) and CsF (91.1 mg, 0.60 mmol, 2.0 equiv.), resulted in higher product yield (67.7 mg, 0.22 mmol, 72%).

R_f = 0.90 (*n*-pentane). **^{1}H NMR** (600 MHz, $CDCl_3$) δ/ppm = 7.21 – 7.17 (m, 1H), 7.17 – 7.13 (m, 2H), 7.03 (d, *J* = 7.4 Hz, 1H), 2.87 (dd, *J* = 13.9, 4.2 Hz, 1H), 2.36 (ddd, *J* = 13.6, 11.5, 1.7 Hz, 1H), 1.41 – 1.32 (m, 1H), 1.06 (t, *J* = 7.9 Hz, 9H), 0.93 (d, *J* = 7.3 Hz, 3H), 0.77 (q, *J* = 7.7 Hz, 6H). **^{13}C NMR** (151 MHz, $CDCl_3$) δ/ppm = 145.0, 134.1, 129.5, 129.0, 127.2, 125.9, 39.5, 21.2, 15.4, 9.3, 2.9. **HRMS** (APCI) calculated for $C_{13}H_{20}GeCl$: 285.0460 $[M-CH_2CH_3]^+$, found: 285.0457. **IR** (neat): ν/ cm^{-1} = 2947, 2870, 2325, 2090, 1864, 1595, 1461.

Triethyl(1-(4-fluorophenyl)propan-2-yl)germane (12)

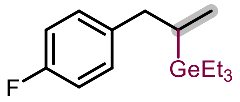

Prepared, following the general procedure A from 1-allyl-4-fluorobenzene (40.9 mg, 0.30 mmol) with LiO*t*Bu (2.4 mg, 0.03 mmol, 0.1 equiv.) and CsF (4.6 mg, 0.03 mmol, 0.1 equiv.). The title product was obtained after purification by flash chromatography on silica gel (*n*-pentane) as a colorless oil (71.3 mg, 0.24 mmol, 80%). Using stoichiometric amounts of bases, LiO*t*Bu (48.0 mg, 0.60 mmol, 2.0 equiv.) and CsF (91.1 mg, 0.60 mmol, 2.0 equiv.), resulted in slightly higher product yield (79.4 mg, 0.27 mmol, 89%).

The reaction was also performed on *larger scale*, following general procedure A from 1-allyl-4-fluorobenzene (204.3 mg, 1.50 mmol) with LiO*t*Bu (12.0 mg, 0.15 mmol, 0.1 equiv.) and CsF (22.8 mg, 0.15 mmol, 0.1 equiv.). The yield was determined by ^{19}F NMR using 1,4-difluorobenzene as an internal standard showing product formation in 90%.

R_f = 0.91 (*n*-pentane). **^{1}H NMR** (600 MHz, $CDCl_3$) δ/ppm = 7.12 – 7.07 (m, 2H), 6.98 – 6.92 (m, 2H), 2.86 (dd, *J* = 13.7, 4.1 Hz, 1H), 2.40 – 2.33 (m, 1H), 1.38 – 1.30 (m, 1H), 1.05 (t, *J* = 7.9 Hz, 9H), 0.92 (d, *J* = 7.3 Hz, 3H), 0.76 (q, *J* = 7.8 Hz, 6H). **^{13}C NMR** (151 MHz, $CDCl_3$) δ/ppm = 161.34 (d, *J* = 242.9 Hz), 138.38 (d, *J* = 3.3 Hz), 130.16 (d, *J* = 7.8 Hz), 114.9 (d, *J* = 21.1 Hz) 39.0, 21.5, 15.4, 9.3, 2.9. **^{19}F NMR** (564 MHz, $CDCl_3$) δ/ppm = -118.23 (s, 1F). **HRMS** (APCI) calculated for $C_{13}H_{20}GeF$: 269.0755 $[M-CH_2CH_3]^+$, found: 269.0755. **IR** (neat): ν/ cm^{-1} = 2947, 2870, 2323, 1881, 1507, 1222.

Triethyl(1-(3-(trifluoromethyl)phenyl)propan-2-yl)germane (13)

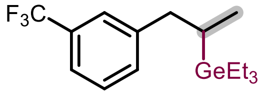

Prepared, following the general procedure A from 1-allyl-3-(trifluoromethyl)benzene (55.9 mg, 0.30 mmol) with LiO*t*Bu (2.4 mg, 0.03 mmol, 0.1 equiv.) and CsF (4.6 mg, 0.03 mmol, 0.1 equiv.). The title product was obtained after purification by flash chromatography on silica gel (*n*-pentane) as a colorless oil (25.1 mg, 0.07 mmol, 24%). Using stoichiometric amounts of bases, LiO*t*Bu (48.0 mg, 0.60 mmol, 2.0 equiv.) and CsF (91.1 mg, 0.60 mmol, 2.0 equiv.), resulted in higher product yield (82.2 mg, 0.24 mmol, 79%).

R_f = 0.91 (*n*-pentane). **^{1}H NMR** (600 MHz, $CDCl_3$) δ/ppm = 7.44 (d, *J* = 7.7 Hz, 1H), 7.41 – 7.36 (m, 2H), 7.33 (d, *J* = 7.7 Hz, 1H), 2.94 (dd, *J* = 13.8, 4.4 Hz, 1H), 2.46 (dd, *J* = 13.7, 11.7 Hz, 1H), 1.43 – 1.35 (m, 1H), 1.06 (t, *J* = 7.9 Hz, 9H), 0.93 (d, *J* = 7.3 Hz, 3H), 0.78 (q, *J* = 7.9 Hz, 6H). **^{13}C NMR** (151 MHz, $CDCl_3$) δ/ppm = 143.7, 132.3, 130.6 (q, *J* = 31.9 Hz), 128.6, 125.5 (q, *J* = 3.8 Hz), 124.5 (q, *J* = 272.3 Hz), 122.7 (q, *J* = 3.8 Hz), 39.7, 21.2, 15.4, 9.3, 2.9. **^{19}F NMR** (564 MHz, $CDCl_3$) δ/ppm = 62.52 (s, 3F). **HRMS** (APCI) calculated

for $C_{14}H_{20}GeF_3$: 319.0723 $[M-CH_2CH_3]^+$, found: 319.0720. **IR** (neat): ν/ cm^{-1} = 2950, 2872, 1452, 1327, 1125, 699.

N,*N*-Diphenyl-4-(2-(triethylgermyl)propyl)aniline (14)

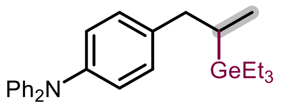

Prepared, following the general procedure A from 4-allyl-*N*,*N*-diphenylaniline (85.6 mg, 0.30 mmol) with LiO*t*Bu (2.4 mg, 0.03 mmol, 0.1 equiv.) and CsF (4.6 mg, 0.03 mmol, 0.1 equiv.). The title product was obtained after purification by flash chromatography on silica gel (*n*-pentane/Et_2O 98:2) as a clear yellowish oil (130.6 mg, 0.29 mmol, 98%).

R$_f$ = 0.80 (*n*-pentane). **^{1}H NMR** (600 MHz, $CDCl_3$) δ/ppm = 7.24 – 7.20 (m, 4H), 7.08 – 7.04 (m, 6H), 7.02 – 7.00 (m, 2H), 6.99 – 6.94 (m, 2H), 2.82 (dd, *J* = 13.7, 5.0 Hz, 1H), 2.44 – 2.38 (m, 1H), 1.39 (ddd, *J* = 11.8, 7.7, 5.0 Hz, 1H), 1.04 (t, *J* = 7.9 Hz, 9H), 0.99 (d, *J* = 7.3 Hz, 3H), 0.75 (q, *J* = 7.9 Hz, 6H). **^{13}C NMR** (151 MHz, $CDCl_3$) δ/ppm = 148.2, 145.6, 137.8, 129.8, 129.2, 124.9, 123.8, 122.3, 39.4, 21.6, 15.9, 9.4, 3.0. **HRMS** (ESI) calculated for $C_{25}H_{30}GeN$: 418.1585 $[M-CH_2CH_3]^+$, found: 418.1581. **IR** (neat): ν/ cm^{-1} = 2939, 2866, 1689, 1589, 1501, 1281.

(*Z*)-triethyl(1-(2-(prop-1-en-1-yloxy)phenyl)propan-2-yl)germane (15)

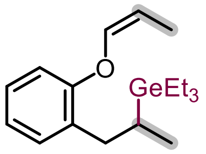

Prepared, following the general procedure A from 1-allyl-2-(allyloxy)benzene (52.3 mg, 0.30 mmol) with LiO*t*Bu (2.4 mg, 0.03 mmol, 0.1 equiv.) and CsF (4.6 mg, 0.03 mmol, 0.1 equiv.). The title product was obtained after purification by flash chromatography on silica gel (*n*-pentane/Et_2O 98:2) as a colorless oil (95.4 mg, 0.28 mmol, 95%).

R$_f$ = 0.84 (*n*-pentane). **^{1}H NMR** (600 MHz, $CDCl_3$) δ/ppm = 7.18 – 7.10 (m, 2H), 6.99 – 6.94 (m, 1H), 6.90 (d, *J* = 8.0 Hz, 1H), 6.34 (d, *J* = 5.8 Hz, 1H), 4.86 – 4.80 (m, 1H), 2.99 (dd, *J* = 13.3, 3.5 Hz, 1H), 2.50 – 2.41 (m, 1H), 1.72 (d, *J* = 6.7 Hz, 3H), 1.50 – 1.41 (m, 1H), 1.07 (t, *J* = 7.9 Hz, 9H), 0.94 (d, *J* = 7.4 Hz, 3H), 0.79 (q, *J* = 7.9 Hz, 6H). **^{13}C NMR** (151 MHz, $CDCl_3$) δ/ppm = 155.83, 141.66, 132.03, 131.08, 126.98, 122.13, 114.95, 106.45, 33.90, 20.09, 15.46, 9.57, 9.33, 2.87. **HRMS** (APCI) calculated for $C_{16}H_{25}GeO$: 307.1112 $[M-CH_2CH_3]^+$, found: 307.1110. **IR** (neat): ν/ cm^{-1} = 3044, 2946, 2870, 1668, 1487, 1247.

(1-(Benzo[*b*]thiophen-2-yl)propan-2-yl)triethylgermane (16)

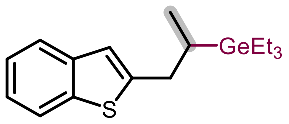

Prepared, following the general procedure A from 2-allylbenzo[*b*]thiophene (52.3 mg, 0.30 mmol) with LiO*t*Bu (2.4 mg, 0.03 mmol, 0.1 equiv.) and CsF (4.6 mg, 0.03 mmol, 0.1 equiv.). The title product was obtained after purification by flash chromatography on silica gel (*n*-pentane) as colorless oil (40.2 mg, 0.12 mmol, 40%). Using stoichiometric amounts of bases, LiO*t*Bu (48.0 mg, 0.60 mmol, 2.0 equiv.) and CsF (91.1 mg, 0.60 mmol, 2.0 equiv.), resulted in higher product yield (86.4 mg, 0.26 mmol, 86%).

R$_f$ = 0.81 (*n*-pentane/Et_2O 94:6). **^{1}H NMR** (600 MHz, $CDCl_3$) δ/ppm = 7.76 (d, *J* = 7.9 Hz, 1H), 7.66 (d, *J* = 7.8 Hz, 1H), 7.32 – 7.28 (m, 1H), 7.26 – 7.22 (m, 1H), 6.99 (s, 1H), 3.14 (dd, *J* = 14.8, 3.9 Hz, 1H), 2.77 – 2.71 (m, 1H), 1.52 – 1.44 (m, 1H), 1.11 – 1.04 (m, 12H), 0.80 (q, *J* = 7.9 Hz, 6H). **^{13}C NMR** (151 MHz, $CDCl_3$) δ/ppm = 147.4, 140.4, 139.6, 124.1, 123.4, 122.8, 122.3, 121.1, 34.9, 21.7, 15.7, 9.3, 2.9. **HRMS** (ESI)

calculated for $C_{15}H_{21}GeS$: 307.0570 $[M-CH_2CH_3]^+$, found: 307.0567. **IR** (neat): ν/ cm^{-1} = 2947, 2870, 1677, 1457, 1014.

Triethyl(1-(thiophen-2-yl)propan-2-yl)germane (17)

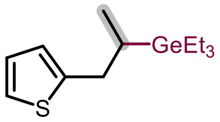

Prepared, following the general procedure A from 2-allylthiophene (37.3 mg, 0.30 mmol) with LiO*t*Bu (2.4 mg, 0.03 mmol, 0.1 equiv.) and CsF (4.6 mg, 0.03 mmol, 0.1 equiv.). The title product was obtained after purification by flash chromatography on silica gel (*n*-pentane) as colorless oil (69.9 mg, 0.25 mmol, 82%). Using stoichiometric amounts of bases, LiO*t*Bu (48.0 mg, 0.60 mmol, 2.0 equiv.) and CsF (91.1 mg, 0.60 mmol, 2.0 equiv.), resulted in a similar product yield (75.2 mg, 0.26 mmol, 88%).

R_f = 0.80 (*n*-pentane). **^{1}H NMR** (600 MHz, $CDCl_3$) δ/ppm = 7.11 (dd, *J* = 5.1, 1.2 Hz, 1H), 6.91 (dd, *J* = 5.1, 3.4 Hz, 1H), 6.78 – 6.75 (m, 1H), 3.06 (dd, *J* = 14.8, 5.0 Hz, 1H), 2.68 (dd, *J* = 14.8, 11.2 Hz, 1H), 1.43 – 1.35 (m, 1H), 1.06 – 1.02 (m, 12H), 0.76 (q, *J* = 8.0 Hz, 6H). **^{13}C NMR** (151 MHz, $CDCl_3$) δ/ppm = 146.2, 126.7, 124.6, 123.0, 34.0, 22.4, 15.8, 9.3, 2.9. **HRMS** (ESI) calculated for $C_{11}H_{19}GeS$: 257.0414 $[M-CH_2CH_3]^+$, found: 257.0410. **IR** (neat): ν/ cm^{-1} = 2947, 2906, 2870, 1458, 1015.

Triphenyl(2-(triethylgermyl)propyl)silane (18)

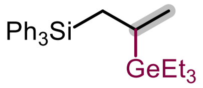

Prepared, following the general procedure A from allyltriphenylsilane (90.1 mg, 0.30 mmol) with LiO*t*Bu (2.4 mg, 0.03 mmol, 0.1 equiv.) and CsF (4.6 mg, 0.03 mmol, 0.1 equiv.). The title product was obtained after purification by flash chromatography on silica gel (*n*-pentane) as a milky viscous oil (62.3 mg, 0.14 mmol, 45%).

R_f = 0.38 (*n*-pentane). **^{1}H NMR** (600 MHz, $CDCl_3$) δ/ppm = 7.57 – 7.54 (m, 6H), 7.42 – 7.34 (m, 9H), 1.65 (d, *J* = 13.9 Hz, 1H), 1.44 – 1.33 (m, 2H), 1.01 (t, *J* = 7.9 Hz, 9H), 0.89 (d, *J* = 6.9 Hz, 3H), 0.74 (q, *J* = 7.9 Hz, 6H). **^{13}C NMR** (151 MHz, $CDCl_3$) δ/ppm = 136.1, 135.9, 129.4, 127.9, 19.1, 16.7, 14.2, 9.4, 2.6. **HRMS** (ESI) calculated for $C_{25}H_{31}GeSi$: 433.1401 $[M-CH_2CH_3]^+$, found: 433.1408. **IR** (neat): ν/ cm^{-1} = 3066, 2926, 2326, 1763, 1426, 1107.

Triethyl(1-(4,4,5,5-tetramethyl-1,3,2-dioxaborolan-2-yl)propan-2-yl)germane (19)

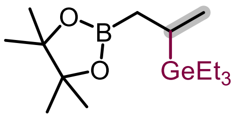

Prepared, following the general procedure A from 2-allyl-4,4,5,5-tetramethyl-1,3,2-dioxaborolane (50.4 mg, 0.30 mmol) with LiO*t*Bu (2.4 mg, 0.03 mmol, 0.1 equiv.) and CsF (4.6 mg, 0.03 mmol, 0.1 equiv.). The title product was obtained after purification by flash chromatography on silica gel (*n*-pentane/Et_2O 94:6) as a colorless oil (46.4 mg, 0.14 mmol, 47%).

R_f = 0.90 (*n*-pentane/Et_2O 94:6). **^{1}H NMR** (600 MHz, $CDCl_3$) δ/ppm = 1.35 – 1.27 (m, 1H), 1.26 (s, 6H), 1.25 (s, 6H), 1.07 (d, *J* = 7.4 Hz, 3H), 1.02 (t, *J* = 8.0 Hz, 9H), 1.05 – 0.96 (m, 1H), 0.80 – 0.74 (m, 1H), 0.72 (q, *J* = 7.7 Hz, 6H). **^{13}C NMR** (151 MHz, $CDCl_3$) δ/ppm = 83.1, 25.2, 24.9, 19.0, 14.8, 9.4, 2.7. **HRMS** (ESI) calculated for $C_{13}H_{28}GeBO_2$: 301.1389 $[M-CH_2CH_3]^+$, found: 301.1382. **IR** (neat): ν/ cm^{-1} = 2946, 2871, 1800, 1355, 1233.

Diphenyl(2-(triethylgermyl)propyl)phosphine oxide (20)

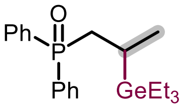

Prepared, following the general procedure A from allyldiphenylphosphine (67.9 mg, 0.30 mmol) with LiO^{t}Bu (2.4 mg, 0.03 mmol, 0.1 equiv.) and CsF (4.6 mg, 0.03 mmol, 0.1 equiv.). The title product was obtained after purification by preparative HPLC (ethylacetate, 20 mL/min) as white solid (59.4 mg, 0.15 mmol, 49%).

R$_f$ = 0. (*n*-pentane). **^{1}H NMR** (600 MHz, CDCl$_3$) δ/ppm = 7.79 – 7.71 (m, 4H), 7.52 – 7.42 (m, 6H), 2.42 – 2.32 (m, 1H), 2.28 – 2.18 (m, 1H), 1.67 – 1.56 (m, 1H), 1.14 (d, *J* = 7.3 Hz, 3H), 0.99 (t, *J* = 7.9 Hz, 9H), 0.78 – 0.71 (m, 6H). **^{13}C NMR** (151 MHz, CDCl$_3$) δ/ppm = 131.6 (d, *J* = 7.1 Hz), 131.0 (d, *J* = 8.1 Hz), 130.8 (d, *J* = 8.8 Hz), 128.7 (d, *J* = 10.6 Hz), 33.1 (d, *J* = 66.6 Hz), 17.4, 12.9 (d, *J* = 7.0 Hz), 9.2, 2.6. **^{31}P NMR** (243 MHz, CDCl$_3$) δ/ppm = 32.18 (s, 1P). **HRMS** (ESI) calculated for $C_{21}H_{31}GePO$: 427.1217 [M+Na]$^+$, found: 427.1234. **IR** (neat): ν/ cm^{-1} = 2950, 2873, 1459, 1435, 1176.

Note: The phosphine oxidizes quickly to the phosphine oxide. Only the oxide could be isolated.

Triethyl(phenethyl)germane (21)

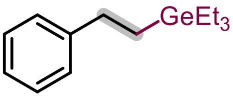

Prepared, following the general procedure A from styrene (31.2 mg, 0.30 mmol) with LiO^{t}Bu (2.4 mg, 0.03 mmol, 0.1 equiv.) and CsF (4.6 mg, 0.03 mmol, 0.1 equiv.). The title product was obtained after purification by flash chromatography on silica gel (*n*-pentane) as a colorless oil (60.2 mg, 0.24 mmol, 81%).

R$_f$ = 0.70 (*n*-pentane). **^{1}H NMR** (600 MHz, CDCl$_3$) δ/ppm = 7.30 – 7.26 (m, 2H), 7.21 (d, *J* = 7.5 Hz, 2H), 7.18 – 7.15 (m, 1H), 2.69 – 2.65 (m, 2H), 1.09 – 0.99 (m, 11H), 0.74 (q, *J* = 7.9 Hz, 6H). **^{13}C NMR** (151 MHz, CDCl$_3$) δ/ppm = 145.6, 128.4, 127.9, 125.7, 31.5, 13.7, 9.1, 4.0. **MS** (EI) m/z (%): 237 (100) [M-CH$_2$CH$_3$]$^+$, 133 (86), 105 (80). These data are in agreement with those reported previously in the literature.[1]

(4-(*tert*-Butyl)phenethyl)triethylgermane (22)

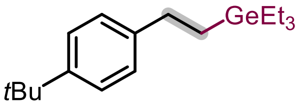

Prepared, following the general procedure A from 4-*tert*-butylstyrene (48.1 mg, 0.30 mmol) with LiO^{t}Bu (2.4 mg, 0.03 mmol, 0.1 equiv.) and CsF (4.6 mg, 0.03 mmol, 0.1 equiv.). The title product was obtained after purification by flash chromatography on silica gel (*n*-pentane) as a colorless oil (72.0 mg, 0.22 mmol, 75%). Using stoichiometric amounts of bases, LiO^{t}Bu (48.0 mg, 0.60 mmol, 2.0 equiv.) and CsF (91.1 mg, 0.60 mmol, 2.0 equiv.), resulted in higher product yield (78.0 mg, 0.24 mmol, 81%).

R$_f$ = 0.76 (*n*-pentane). **^{1}H NMR** (600 MHz, CDCl$_3$) δ/ppm = 7.31 (d, *J* = 8.3 Hz, 2H), 7.15 (d, *J* = 8.3 Hz, 2H), 2.66 – 2.62 (m, 2H), 1.31 (s, 9H), 1.08 – 1.04 (m, 2H), 1.03 (t, *J* = 7.9 Hz, 9H), 0.74 (q, *J* = 7.9 Hz, 6H). **^{13}C NMR** (151 MHz, CDCl$_3$) δ/ppm = 148.5, 142.6, 127.5, 125.3, 34.5, 31.6, 30.8, 13.6, 9.1, 4.0. **HRMS** (ESI) calculated for $C_{13}H_{21}Ge$: 293.1319 [M-CH$_2$CH$_3$]$^+$, found: 293.1320. **IR** (neat): ν/ cm^{-1} =2955, 2873, 1683, 1300, 1016.

(2,2-Diphenylethyl)triethylgermane (23)

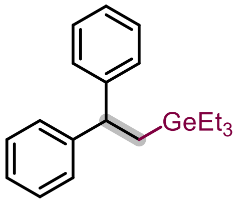

Prepared, following the general procedure A from 1,1-diphenylethylene (54.1 mg, 0.30 mmol) with LiO^{t}Bu (2.4 mg, 0.03 mmol, 0.1 equiv.) and CsF (4.6 mg, 0.03 mmol, 0.1 equiv.). The title product was obtained after purification by flash chromatography on silica gel (*n*-pentane) as a colorless oil (88.7 mg, 0.26 mmol, 87%). Using stoichiometric amounts of bases, LiO^{t}Bu (48.0 mg, 0.60 mmol, 2.0 equiv.) and CsF (91.1 mg, 0.60 mmol, 2.0 equiv.), resulted in higher product yield (97.3 mg, 0.29 mmol, 95%).

R_f = 0.82 (*n*-pentane). **^{1}H NMR** (600 MHz, $CDCl_3$) δ/ppm = 7.29 (dd, *J* = 8.1, 1.5 Hz, 4H), 7.27 – 7.23 (m, 4H), 7.17 – 7.12 (m, 2H), 4.09 (t, *J* = 8.1 Hz, 1H), 1.56 (d, *J* = 8.1 Hz, 2H), 0.91 (t, *J* = 7.9 Hz, 9H), 0.53 (q, *J* = 7.9 Hz, 6H). **^{13}C NMR** (151 MHz, $CDCl_3$) δ/ppm = 147.4, 128.5, 127.6, 126.1, 48.2, 19.6, 9.0, 4.3. **MS** (EI) m/z (%): 313 (100) $[M\text{-}CH_2CH_3]^+$, 209 (49), 133 (40). These data are in agreement with those reported previously in the literature.[1]

Triethyl(4-fluorophenethyl)germane (24)

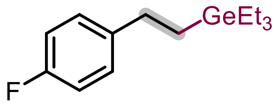

Prepared, following the general procedure A from 4-fluorostyrene (36.6 mg, 0.30 mmol) with LiO^{t}Bu (2.4 mg, 0.03 mmol, 0.1 equiv.) and CsF (4.6 mg, 0.03 mmol, 0.1 equiv.). The title product was obtained after purification by flash chromatography on silica gel (*n*-pentane) as a colorless oil (52.6 mg, 0.19 mmol, 62%).

R_f = 0.83 (*n*-pentane). **^{1}H NMR** (600 MHz, $CDCl_3$) δ/ppm = 7.17 – 7.12 (m, 2H), 6.98 – 6.92 (m, 2H), 2.67 – 2.61 (m, 2H), 1.06 – 0.99 (m, 11H), 0.73 (q, *J* = 7.9 Hz, 6H). **^{13}C NMR** (151 MHz, $CDCl_3$) δ/ppm = 161.2 (d, *J* = 242.8 Hz), 141.2 (d, *J* = 3.2 Hz), 129.2 (d, *J* = 7.7 Hz), 115.1 (d, *J* = 21.0 Hz), 30.7, 13.8, 9.1, 4.0. **^{19}F NMR** (564 MHz, $CDCl_3$) δ/ppm = -118.4 (s, 1F). **HRMS** (ESI) calculated for $C_{12}H_{18}FGe$: 255.0599 $[M\text{-}CH_2CH_3]^+$, found: 255.0598. **IR** (neat): ν/ cm^{-1} = 2948, 2872, 1507, 1223, 1015.

Triethyl(4-(trifluoromethyl)phenethyl)germane (25)

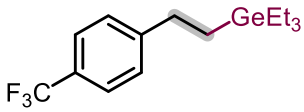

Prepared, following the general procedure A from 4-(trifluoromethyl)styrene (51.6 mg, 0.30 mmol) with LiO^{t}Bu (2.4 mg, 0.03 mmol, 0.1 equiv.) and CsF (4.6 mg, 0.03 mmol, 0.1 equiv.). The title product was obtained after purification by flash chromatography on silica gel (*n*-pentane) as a colorless oil (24.3 mg, 0.07 mmol, 24%).

R_f = 0.74 (*n*-pentane). **^{1}H NMR** (600 MHz, $CDCl_3$) δ/ppm = 7.52 (d, *J* = 8.1 Hz, 2H), 7.31 (d, *J* = 8.0 Hz, 2H), 2.74 – 2.68 (m, 2H), 1.08 – 1.00 (m, 11H), 0.75 (q, *J* = 7.9 Hz, 6H). **^{13}C NMR** (151 MHz, $CDCl_3$) δ/ppm = 149.7, 128.2, 128.0 (q, *J* = 32.3 Hz), 125.4 (q, *J* = 3.9 Hz), 124.6 (q, *J* = 272.0 Hz), 31.4, 13.5, 9.1, 4.0. **^{19}F NMR** (564 MHz, $CDCl_3$) δ/ppm = -62.2. **MS** (EI) m/z (%): 305 (21) $[M\text{- }CH_2CH_3]^+$, 153 (100), 133 (10). These data are in agreement with those reported previously in the literature.[2]

Triethyl(1-(3-tolyl)butan-2-yl)germane (26)

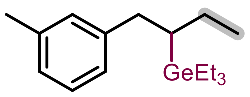

Prepared, following the general procedure A from 1-(but-3-en-1-yl)-3-methylbenzene (43.9 mg, 0.30 mmol) with LiO^{t}Bu (2.4 mg, 0.03 mmol, 0.1 equiv.) and CsF (4.6 mg, 0.03 mmol, 0.1 equiv.). The title product was obtained after purification by flash chromatography on silica gel (*n*-pentane) as colorless oil (72.3 mg, 0.24 mmol, 78%).

R$_f$ = 0.86 (*n*-pentane). **^{1}H NMR** (600 MHz, $CDCl_3$) δ/ppm = 7.17 – 7.12 (m, 1H), 7.01 – 6.94 (m, 3H), 2.75 (dd, *J* = 13.9, 5.1 Hz, 1H), 2.57 (dd, *J* = 13.9, 10.3 Hz, 1H), 2.33 (s, 3H), 1.49 – 1.42 (m, 2H), 1.42 – 1.37 (m, 1H), 1.03 (t, *J* = 7.9 Hz, 9H), 0.86 (t, *J* = 7.3 Hz, 3H), 0.75 (q, *J* = 8.1 Hz, 6H). **^{13}C NMR** (151 MHz, $CDCl_3$) δ/ppm = 143.1, 137.7, 129.7, 128.1, 126.4, 126.0, 37.0, 29.3, 23.3, 21.6, 13.7, 9.4, 3.9. **HRMS** (ESI) calculated for $C_{15}H_{25}Ge$: 279.1163 $[M\text{-}CH_2CH_3]^+$, found: 279.1164. **IR** (neat): ν/ cm^{-1} = 3016, 2950, 1457, 1014.

(1-(4-(*tert*-Butyl)phenyl)butan-2-yl)triethylgermane (27)

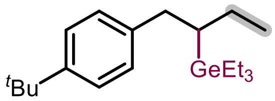

Prepared, following the general procedure A from 1-(but-3-en-1-yl)-4-(tert-butyl)benzene (56.5 mg, 0.30 mmol) with LiO^{t}Bu (2.4 mg, 0.03 mmol, 0.1 equiv.) and CsF (4.6 mg, 0.03 mmol, 0.1 equiv.). The title product was obtained after purification by flash chromatography on silica gel (*n*-pentane) as a colorless oil (79.6 mg, 0.23 mmol, 76%).

R$_f$ = 0.83 (*n*-pentane). **^{1}H NMR** (600 MHz, $CDCl_3$) δ/ppm = 7.28 (d, *J* = 8.1 Hz, 2H), 7.10 (d, *J* = 8.1 Hz, 2H), 2.75 (dd, *J* = 14.0, 5.1 Hz, 1H), 2.59 (dd, *J* = 14.0, 10.4 Hz, 1H), 1.49 – 1.43 (m, 2H), 1.43 – 1.36 (m, 1H), 1.31 (s, 9H), 1.03 (t, *J* = 7.9 Hz, 9H), 0.87 (t, *J* = 7.4 Hz, 3H), 0.74 (q, *J* = 8.0 Hz, 6H). **^{13}C NMR** (151 MHz, $CDCl_3$) δ/ppm = 148.5, 134.0, 128.5, 125.1, 36.4, 34.5, 31.6, 29.3, 23.3, 13.6, 9.4, 3.9. **HRMS** (APCI) calculated for $C_{18}H_{31}Ge$: 321.1632 $[M\text{-}CH_2CH_3]^+$, found: 321.1634. **IR** (neat): ν/ cm^{-1} = 2955, 2870, 2090, 1897, 1459, 1016.

(1-([1,1'-Biphenyl]-3-yl)butan-2-yl)triethylgermane (28)

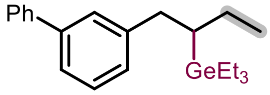

Prepared, following the general procedure A from 3-(but-3-en-1-yl)-1,1'-biphenyl (62.4 mg, 0.30 mmol) with LiO^{t}Bu (2.4 mg, 0.03 mmol, 0.1 equiv.) and CsF (4.6 mg, 0.03 mmol, 0.1 equiv.). The title product was obtained after purification by flash chromatography on silica gel (*n*-pentane) as a colorless oil (100.0 mg, 0.27 mmol, 90%).

R$_f$ = 0.55 (*n*-pentane). **^{1}H NMR** (600 MHz, $CDCl_3$) δ/ppm = 7.61 – 7.57 (m, 2H), 7.46 – 7.42 (m, 2H), 7.40 (d, *J* = 8.9 Hz, 2H), 7.36 – 7.32 (m, 2H), 7.17 (d, *J* = 7.5 Hz, 1H), 2.86 (dd, *J* = 13.9, 4.9 Hz, 1H), 2.69 (dd, *J* = 13.8, 10.1 Hz, 1H), 1.54 – 1.42 (m, 3H), 1.05 (t, *J* = 7.9 Hz, 9H), 0.89 (t, *J* = 7.2 Hz, 3H), 0.77 (q, *J* = 8.0 Hz, 6H). **^{13}C NMR** (151 MHz, $CDCl_3$) δ/ppm = 143.6, 141.7, 141.2, 128.8, 128.6, 127.9, 127.8, 127.3, 127.3, 124.7, 37.2, 29.4, 23.4, 13.7, 9.4, 4.0. **HRMS** (APCI) calculated for $C_{20}H_{27}Ge$: 341.1319 $[M\text{-}CH_2CH_3]^+$, found: 341.1315. **IR** (neat): ν/ cm^{-1} = 3059, 2950, 2870, 2324, 2111, 1598, 1457.

Triethyl(1-(naphthalen-2-yl)butan-2-yl)germane (29)

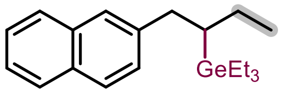

Prepared, following the general procedure A from 2-(but-3-en-1-yl)naphthalene (54.7 mg, 0.30 mmol) with LiO*t*Bu (2.4 mg, 0.03 mmol, 0.1 equiv.) and CsF (4.6 mg, 0.03 mmol, 0.1 equiv.). The title product was obtained after purification by flash chromatography on silica gel (*n*-pentane) as colorless oil (83.0 mg, 0.24 mmol, 81%).

R_f = 0.79 (*n*-pentane). **^{1}H NMR** (600 MHz, $CDCl_3$) δ/ppm = 7.82 – 7.74 (m, 3H), 7.60 (s, 1H), 7.47 – 7.39 (m, 2H), 7.34 (dd, *J* = 8.3, 1.8 Hz, 1H), 2.97 (dd, *J* = 14.0, 4.7 Hz, 1H), 2.77 (dd, *J* = 13.8, 10.5 Hz, 1H), 1.55 – 1.45 (m, 3H), 1.06 (t, *J* = 7.9 Hz, 9H), 0.87 (t, *J* = 7.3 Hz, 3H), 0.79 (q, *J* = 8.0 Hz, 6H). **^{13}C NMR** (151 MHz, $CDCl_3$) δ/ppm = 140.7, 133.7, 132.1, 127.8, 127.7 (2C), 127.5, 127.0, 125.9, 125.1, 37.3, 29.1, 23.3, 13.7, 9.4, 4.0. **HRMS** (APCI) calculated for $C_{18}H_{25}Ge$: 315.1163 $[M\text{-}CH_2CH_3]^+$, found: 315.1169. **IR** (neat): ν/ cm^{-1} = 3052, 2950, 2327, 1796, 1457.

Triethyl(1-(4-fluorophenyl)butan-2-yl)germane (30)

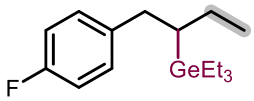

Prepared, following the general procedure A from 1-(but-3-en-1-yl)-4-fluorobenzene (45.1 mg, 0.30 mmol) with LiO*t*Bu (2.4 mg, 0.03 mmol, 0.1 equiv.) and CsF (4.6 mg, 0.03 mmol, 0.1 equiv.). The title product was obtained after purification by flash chromatography on silica gel (*n*-pentane) as colorless oil (81.2 mg, 0.26 mmol, 87%).

R_f = 0.84 (*n*-pentane). **^{1}H NMR** (600 MHz, $CDCl_3$) δ/ppm = 7.14 – 7.09 (m, 2H), 6.97 – 6.92 (m, 2H), 2.75 (dd, *J* = 14.0, 5.4 Hz, 1H), 2.59 (dd, *J* = 14.1, 10.3 Hz, 1H), 1.49 – 1.38 (m, 2H), 1.37 – 1.32 (m, 1H), 1.03 (t, *J* = 7.9 Hz, 9H), 0.86 (t, *J* = 7.4 Hz, 3H), 0.77 – 0.71 (m, 6H). **^{13}C NMR** (151 MHz, $CDCl_3$) δ/ppm = 161.3 (d, *J* = 242.9 Hz), 138.7 (d, *J* = 3.1 Hz), 130.2 (d, *J* = 7.5 Hz), 114.9 (d, *J* = 21.1 Hz) 36.3, 29.5, 23.2, 13.7, 9.3, 3.9. **^{19}F NMR** (564 MHz, $CDCl_3$) δ/ppm = -118.18 (s, 1F). **HRMS** (APCI) calculated for $C_{14}H_{22}GeF$: 283.0912 $[M\text{-}CH_2CH_3]^+$, found: 283.0904. **IR** (neat): ν/ cm^{-1} = 2951, 2871, 1878, 1601, 1507.

Triethyl(1-(3-methoxyphenyl)butan-2-yl)germane (31)

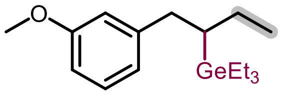

Prepared, following the general procedure A from 1-(but-3-en-1-yl)-3-methoxybenzene (48.7 mg, 0.30 mmol) with LiO*t*Bu (2.4 mg, 0.03 mmol, 0.1 equiv.) and CsF (4.6 mg, 0.03 mmol, 0.1 equiv.). The title product was obtained after purification by flash chromatography on silica gel (*n*-pentane/Et_2O 98:2) as colorless oil (87.2 mg, 0.27 mmol, 90%).

R_f = 0.85 (*n*-pentane/Et_2O 98:2). **^{1}H NMR** (600 MHz, $CDCl_3$) δ/ppm = 7.21 – 7.14 (m, 1H), 6.77 (d, *J* = 7.6 Hz, 1H), 6.73 – 6.70 (m, 2H), 3.80 (s, 3H), 2.76 (dd, *J* = 13.9, 5.1 Hz, 1H), 2.59 (dd, *J* = 13.9, 10.2 Hz, 1H), 1.50 – 1.37 (m, 3H), 1.04 (t, *J* = 7.9 Hz, 9H), 0.86 (t, *J* = 7.3 Hz, 3H), 0.75 (q, *J* = 8.0 Hz, 6H). **^{13}C NMR** (151 MHz, $CDCl_3$) δ/ppm = 159.6, 144.9, 129.1, 121.5, 114.8, 110.9, 55.3, 37.1, 29.2, 23.4, 13.7, 9.4, 3.9. **HRMS** (ESI) calculated for $C_{15}H_{25}GeO$: 295.1112 $[M\text{-}CH_2CH_3]^+$, found: 295.1122. **IR** (neat): ν/ cm^{-1} = 2949, 2871, 2090, 1763, 1596, 1259, 1153.

(1-(Benzo[*d*][1,3]dioxol-5-yl)butan-2-yl)triethylgermane (32)

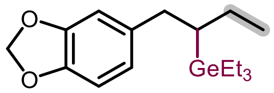

Prepared, following the general procedure A from 5-(but-3-en-1-yl)benzo[*d*][1,3]dioxole (52.9 mg, 0.30 mmol) with LiO^{t}Bu (2.4 mg, 0.03 mmol, 0.1 equiv.) and CsF (4.6 mg, 0.03 mmol, 0.1 equiv.). The title product was obtained after purification by flash chromatography on silica gel (*n*-pentane/Et_2O 98:2) as colorless oil (71.7 mg, 0.21 mmol, 71%). Using stoichiometric amounts of bases, LiO^{t}Bu (48.0 mg, 0.60 mmol, 2.0 equiv.) and CsF (91.1 mg, 0.60 mmol, 2.0 equiv.), resulted in a similar product yield (79.9 mg, 0.24 mmol, 79%).

R_f = 0.85 (*n*-pentane/Et_2O 98:2). **^{1}H NMR** (600 MHz, $CDCl_3$) δ/ppm = 6.71 (d, *J* = 7.8 Hz, 1H), 6.69 – 6.65 (m, 1H), 6.63 – 6.60 (m, 1H), 5.92 (s, 2H), 2.71 (dd, *J* = 14.0, 5.2 Hz, 1H), 2.53 (dd, *J* = 14.0, 10.5 Hz, 1H), 1.49 – 1.41 (m, 2H), 1.36 – 1.30 (m, 1H), 1.04 (t, *J* = 7.9 Hz, 9H), 0.86 (t, *J* = 7.4 Hz, 3H), 0.75 (q, *J* = 7.9 Hz, 6H). **^{13}C NMR** (151 MHz, $CDCl_3$) δ/ppm = 147.6, 145.5, 137.1, 121.6, 109.2, 108.0, 100.8, 36.8, 29.5, 23.2, 13.7, 9.4, 3.9. **HRMS** (ESI) calculated for $C_{15}H_{23}GeO_2$: 309.0904 $[M-CH_2CH_3]^+$, found: 309.0901. **IR** (neat): ν/ cm^{-1} = 2954, 2872, 1442,1243.

Triethyl(1-(4-(methylthio)phenyl)butan-2-yl)germane (33)

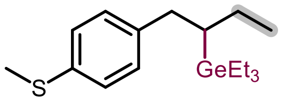

Prepared, following the general procedure A from (4-(but-3-en-1-yl)phenyl)(methyl)sulfane (53.5 mg, 0.30 mmol) with LiO^{t}Bu (2.4 mg, 0.03 mmol, 0.1 equiv.) and CsF (4.6 mg, 0.03 mmol, 0.1 equiv.). The title product was obtained after purification by flash chromatography on silica gel (*n*-pentane) as colorless oil (86.6 mg, 0.26 mmol, 85%). Using stoichiometric amounts of bases, LiO^{t}Bu (48.0 mg, 0.60 mmol, 2.0 equiv.) and CsF (91.1 mg, 0.60 mmol, 2.0 equiv.), resulted in the same product yield (87.5 mg, 0.26 mmol, 86%).

R_f = 0.68 (*n*-pentane). **^{1}H NMR** (600 MHz, $CDCl_3$) δ/ppm = 7.19 (d, *J* = 8.2 Hz, 2H), 7.10 (d, *J* = 8.2 Hz, 2H), 2.75 (dd, *J* = 14.0, 5.2 Hz, 1H), 2.57 (dd, *J* = 14.0, 10.4 Hz, 1H), 2.47 (s, 3H), 1.48 – 1.40 (m, 2H), 1.40 – 1.34 (m, 1H), 1.04 (t, *J* = 7.9 Hz, 9H), 0.86 (t, *J* = 7.4 Hz, 3H), 0.75 (q, *J* = 7.8 Hz, 6H). **^{13}C NMR** (151 MHz, $CDCl_3$) δ/ppm = 140.4, 135.0, 129.5, 127.2, 36.5, 29.3, 23.2, 16.6, 13.7, 9.4, 3.9. **HRMS** (APCI) calculated for $C_{15}H_{25}GeS$: 311.0883 $[M-CH_2CH_3]^+$, found: 311.0886. **IR** (neat): ν/ cm^{-1} = 2937, 2864, 1687, 1503, 1397, 1284.

Triethyl(3-methyl-1-phenylbutan-2-yl)germane (34)

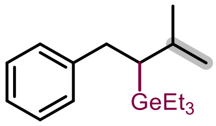

Prepared, following the general procedure A from (3-methylbut-3-en-1-yl)benzene (43.9 mg, 0.30 mmol) with LiO^{t}Bu (2.4 mg, 0.03 mmol, 0.1 equiv.) and CsF (4.6 mg, 0.03 mmol, 0.1 equiv.). The title product was obtained after purification by flash chromatography on silica gel (*n*-pentane) as a colorless oil (59.9 mg, 0.20 mmol, 65%). Using stoichiometric amounts of bases, LiO^{t}Bu (48.0 mg, 0.60 mmol, 2.0 equiv.) and CsF (91.1 mg, 0.60 mmol, 2.0 equiv.), resulted in the same product yield (62.6 mg, 0.20 mmol, 68%).

R_f = 0.68 (*n*-pentane). **^{1}H NMR** (600 MHz, $CDCl_3$) δ/ppm = 7.30 – 7.25 (m, 2H), 7.22 – 7.17 (m, 3H), 2.81 – 2.70 (m, 2H), 1.95 – 1.87 (m, 1H), 1.56 – 1.51 (m, 1H), 1.04 (t, *J* = 7.9 Hz, 9H), 0.97 (d, *J* = 6.8 Hz, 3H), 0.91 (d, *J* = 7.0 Hz, 3H), 0.82 – 0.70 (m, 6H). **^{13}C NMR** (151 MHz, $CDCl_3$) δ/ppm = 143.5, 129.0, 128.2,

125.7, 36.1, 34.2, 28.8, 22.1, 21.8, 9.4, 4.9. **HRMS** (APCI) calculated for $C_{15}H_{25}Ge$: 279.1163 $[M\text{-}CH_2CH_3]^+$, found: 279.1157. **IR** (neat): ν/ cm^{-1} = 2944, 2868, 2325, 1696, 1502, 1397.

Triethyl(1-(6-methoxynaphthalen-2-yl)-3-methylbutan-2-yl)germane (35)

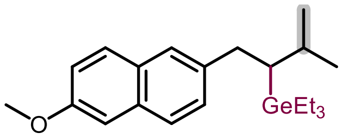

Prepared, following the general procedure A from 2-methoxy-6-(3-methylbut-3-en-1-yl)naphthalene (67.9 mg, 0.30 mmol) with LiO*t*Bu (2.4 mg, 0.03 mmol, 0.1 equiv.) and CsF (4.6 mg, 0.03 mmol, 0.1 equiv.). The title product was obtained after purification by flash chromatography on silica gel (*n*-pentane/Et_2O 98:2) as colorless oil (33.7 mg, 0.09 mmol, 29%). Using stoichiometric amounts of bases, LiO*t*Bu (48.0 mg, 0.60 mmol, 2.0 equiv.) and CsF (91.1 mg, 0.60 mmol, 2.0 equiv.), resulted in higher product yield (108.0 mg, 0.28 mmol, 93%).

$\mathbf{R_f}$ = 0.34 (*n*-pentane). **^{1}H NMR** (600 MHz, $CDCl_3$) δ/ppm = 7.69 – 7.63 (m, 2H), 7.52 (s, 1H), 7.32 – 7.28 (m, 1H), 7.14 – 7.08 (m, 2H), 3.91 (s, 3H), 2.90 – 2.81 (m, 2H), 1.95 – 1.85 (m, 1H), 1.63 – 1.57 (m, 1H), 1.02 (t, *J* = 7.9 Hz, 9H), 0.97 (d, *J* = 6.8 Hz, 3H), 0.90 (d, *J* = 7.0 Hz, 3H), 0.81 – 0.71 (m, 6H). **^{13}C NMR** (151 MHz, $CDCl_3$) δ/ppm = 157.2, 138.7, 133.0, 129.1, 129.0, 128.2, 126.9, 126.7, 118.7, 105.8, 55.4, 36.0, 34.3, 28.8, 22.2, 21.8, 9.4, 5.0. **HRMS** (ESI) calculated for $C_{20}H_{29}GeO$: 359.1425 $[M\text{-}CH_2CH_3]^+$, found: 359.1423. **IR** (neat): ν/ cm^{-1} = 2951, 2870, 1903, 1604, 1459, 1264.

Triethyl(3-methyl-1-phenylbutan-2-yl)germane (36)

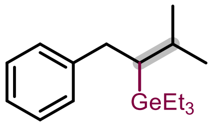

Prepared, following the general procedure A from (3-methylbut-2-en-1-yl)benzene (43.9 mg, 0.30 mmol) with LiO*t*Bu (2.4 mg, 0.03 mmol, 0.1 equiv.) and CsF (4.6 mg, 0.03 mmol, 0.1 equiv.). The title product was obtained after purification by flash chromatography on silica gel (*n*-pentane) as a colorless oil (62.1 mg, 0.20 mmol, 68%).

Analytical data are in agreement with compound **34** (vide supra).

Triethyl(2-phenylpentyl)germane (37)

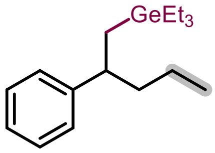

Prepared, following the general procedure A from pent-4-en-2-ylbenzene (43.9 mg, 0.30 mmol) with LiO*t*Bu (2.4 mg, 0.03 mmol, 0.1 equiv.) and CsF (4.6 mg, 0.03 mmol, 0.1 equiv.). The title product was obtained after purification by flash chromatography on silica gel (*n*-pentane) as a colorless oil (55.3 mg, 0.18 mmol, 60%). Using stoichiometric amounts of bases, LiO*t*Bu (48.0 mg, 0.60 mmol, 2.0 equiv.) and CsF (91.1 mg, 0.60 mmol, 2.0 equiv.), resulted in a similar product yield (61.6 mg, 0.20 mmol, 67%).

$\mathbf{R_f}$ = 0.75 (*n*-pentane). **^{1}H NMR** (600 MHz, $CDCl_3$) δ/ppm = 7.27 – 7.23 (m, 2H), 7.18 – 7.13 (m, 3H), 2.69 – 2.61 (m, 1H), 1.59 – 1.51 (m, 2H), 1.19 – 1.05 (m, 4H), 0.91 (t, *J* = 7.9 Hz, 9H), 0.83 (t, *J* = 7.3 Hz, 3H), 0.60 – 0.46 (m, 6H). **^{13}C NMR** (151 MHz, $CDCl_3$) δ/ppm = 148.1, 128.3, 127.5, 125.9, 43.1, 42.6, 21.0, 20.4, 14.2, 9.0, 4.5. **HRMS** (APCI) calculated for $C_{15}H_{25}Ge$: 279.1163 $[M\text{-}CH_2CH_3]^+$, found: 279.1158. **IR** (neat): ν/ cm^{-1} = 3062, 3026, 2951, 2871, 1800, 1455, 1014.

2-(2-(Triethylgermyl)butyl)pyridine (38)

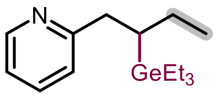

Prepared, following the general procedure A from 2-(but-3-en-1-yl)pyridine (40.0 mg, 0.30 mmol) with LiO^{t}Bu (2.4 mg, 0.03 mmol, 0.1 equiv.) and CsF (4.6 mg, 0.03 mmol, 0.1 equiv.). The title product was obtained after purification by flash chromatography on silica gel (*n*-pentane/Et_2O 9:1) as colorless oil (58.9 mg, 0.20 mmol, 67%).

R$_f$ = 0.23 (*n*-pentane). **^{1}H NMR** (600 MHz, $CDCl_3$) δ/ppm = 8.54 – 8.50 (m, 1H), 7.58 – 7.53 (m, 1H), 7.14 (d, *J* = 7.8 Hz, 1H), 7.08 (dd, *J* = 7.5, 4.9 Hz, 1H), 2.93 (dd, *J* = 13.9, 5.8 Hz, 1H), 2.84 (dd, *J* = 13.9, 10.1 Hz, 1H), 1.68 – 1.62 (m, 1H), 1.54 – 1.40 (m, 2H), 1.03 (t, *J* = 7.9 Hz, 9H), 0.85 (t, *J* = 7.4 Hz, 3H), 0.74 (q, *J* = 8.2 Hz, 6H). **^{13}C NMR** (151 MHz, $CDCl_3$) δ/ppm = 163.1, 149.3, 136.1, 123.3, 121.0, 39.8, 28.0, 23.8, 13.8, 9.4, 3.8. **HRMS** (ESI) calculated for $C_{15}H_{28}GeN$: 296.1428 [M-CH_2CH_3]$^+$, found: 296.1423. **IR** (neat): ν/ cm^{-1} = 3068, 3006, 2950, 2871, 2321, 1900, 1588, 1464.

8-(2-(Triethylgermyl)butyl)quinoline (39)

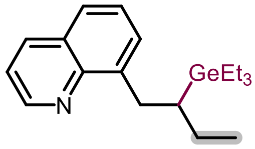

Prepared, following the general procedure A from 8-(but-3-en-1-yl)quinoline (55.0 mg, 0.30 mmol) with (2.4 mg, 0.03 mmol, 0.1 equiv.) and CsF (4.6 mg, 0.03 mmol, 0.1 equiv.). The title product was obtained after purification by flash chromatography on silica gel (*n*-pentane /Et_2O 98:2) as a clear yellow oil (80.5 mg, 0.23 mmol, 78%).

R$_f$ = 0.39 (*n*-pentane/Et_2O 98:2). **^{1}H NMR** (600 MHz, $CDCl_3$) δ/ppm = 8.91 (dd, *J* = 4.1, 1.9 Hz, 1H), 8.11 (d, *J* = 8.2 Hz, 1H), 7.65 (dd, *J* = 8.1, 1.5 Hz, 1H), 7.56 (d, *J* = 7.0 Hz, 1H), 7.49 – 7.42 (m, 1H), 7.36 (dd, *J* = 8.2, 4.1 Hz, 1H), 3.52 (dd, *J* = 13.7, 5.2 Hz, 1H), 3.29 (dd, *J* = 13.7, 10.4 Hz, 1H), 1.78 – 1.70 (m, 1H), 1.54 – 1.40 (m, 2H), 1.05 (t, *J* = 7.9 Hz, 9H), 0.86 (t, *J* = 7.4 Hz, 3H), 0.79 (q, *J* = 7.8 Hz, 6H). **^{13}C NMR** (151 MHz, $CDCl_3$) δ/ppm = 149.2, 147.4, 142.1, 136.2, 129.2, 128.5, 126.1, 125.8, 120.8, 32.1, 28.6, 23.8, 13.8, 9.4, 4.0. **HRMS** (ESI) calculated for $C_{19}H_{30}GeN$: 346.1585 [M+H]$^+$, found: 346.1581. **IR** (neat): ν/ cm^{-1} = 3042, 2949, 2870, 2326, 1718, 1459.

Triethyl(1-phenylpentan-2-yl)germane (40)

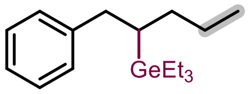

Prepared, following the general procedure A from pent-4-en-1-ylbenzene (43.9 mg, 0.30 mmol) with LiO^{t}Bu (2.4 mg, 0.03 mmol, 0.1 equiv.) and CsF (4.6 mg, 0.03 mmol, 0.1 equiv.). The title product was obtained after purification by flash chromatography on silica gel (*n*-pentane) as a colorless oil (68.2 mg, 0.22 mmol, 74%). Using stoichiometric amounts of bases, LiO^{t}Bu (48.0 mg, 0.60 mmol, 2.0 equiv.) and CsF (91.1 mg, 0.60 mmol, 2.0 equiv.), resulted in a similar product yield (74.5 mg, 0.24 mmol, 81%).

R$_f$ = 0.86 (*n*-pentane). **^{1}H NMR** (600 MHz, $CDCl_3$) δ/ppm = 7.28 – 7.24 (m, 2H), 7.19 – 7.15 (m, 3H), 2.79 (dd, *J* = 14.0, 5.5 Hz, 1H), 2.61 (dd, *J* = 13.9, 9.9 Hz, 1H), 1.48 – 1.41 (m, 1H), 1.42 – 1.26 (m, 3H), 1.25 – 1.17 (m, 1H), 1.03 (t, *J* = 7.9 Hz, 9H), 0.81 (t, *J* = 7.1 Hz, 3H), 0.73 (q, *J* = 7.8 Hz, 6H). **^{13}C NMR** (151 MHz, $CDCl_3$) δ/ppm = 143.2, 128.9, 128.2, 125.7, 37.6, 33.3, 27.4, 22.3, 14.6, 9.4, 3.8. **HRMS** (ESI) calculated for $C_{15}H_{25}Ge$: 279.1163 [M-CH_2CH_3]$^+$, found: 279.1164. **IR** (neat): ν/ cm^{-1} = 3063, 3025, 2950, 2109, 1602, 1454, 1014.

Triethyl(1-phenylnonan-2-yl)germane (41)

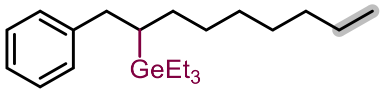

Prepared, following the general procedure A, but stirring for 3 days and heating at 120°C, from non-8-en-1-ylbenzene (60.7 mg, 0.30 mmol) with LiO^{t}Bu (48.0 mg, 0.60 mmol, 2.0 equiv.) and CsF (91.1 mg, 0.60 mmol, 2.0 equiv.). The title product was obtained after purification by flash chromatography on silica gel (*n*-pentane) as a colorless oil (70.8 mg, 0.20 mmol, 65%).

R$_f$ = 0.81 (*n*-pentane). **^{1}H NMR** (600 MHz, $CDCl_3$) δ/ppm = 7.28 – 7.23 (m, 2H), 7.19 – 7.14 (m, 3H), 2.78 (dd, *J* = 13.9, 5.1 Hz, 1H), 2.61 (dd, *J* = 14.0, 9.6 Hz, 1H), 1.46 – 1.35 (m, 3H), 1.29 – 1.24 (m, 3H), 1.22 – 1.14 (m, 7H), 1.02 (t, *J* = 7.9 Hz, 9H), 0.87 (t, *J* = 7.2 Hz, 3H), 0.73 (q, *J* = 7.9 Hz, 6H). **^{13}C NMR** (151 MHz, $CDCl_3$) δ/ppm = 143.2, 128.9, 128.2, 125.7, 37.6, 32.0, 30.8, 30.1, 29.4, 29.1, 27.6, 22.8, 14.2, 9.4, 3.8. **HRMS** (ESI) calculated for $C_{19}H_{33}Ge$: 335.1789 $[M-CH_2CH_3]^+$, found: 335.1788. **IR** (neat): ν/ cm^{-1} = 2923, 2865, 1457, 1257, 1020.

Triethyl(1-phenyldecan-2-yl)germane (42)

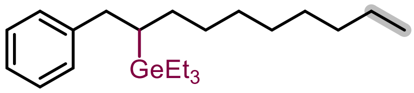

Prepared, following the general procedure A, but stirring for 3 days and heating at 120°C, from dec-9-en-1-ylbenzene (64.9 mg, 0.30 mmol) with LiO^{t}Bu (48.0 mg, 0.60 mmol, 2.0 equiv.) and CsF (91.1 mg, 0.60 mmol, 2.0 equiv.). The title product was obtained after purification by flash chromatography on silica gel (*n*-pentane) as a colorless oil (61.1 mg, 0.16 mmol, 54%).

R$_f$ = 0.78 (*n*-pentane). **^{1}H NMR** (600 MHz, $CDCl_3$) δ/ppm = 7.29 – 7.25 (m, 2H), 7.20 – 7.16 (m, 3H), 2.80 (dd, *J* = 13.9, 5.1 Hz, 1H), 2.63 (dd, *J* = 13.9, 9.6 Hz, 1H), 1.48 – 1.35 (m, 3H), 1.32 – 1.27 (m, 3H), 1.25 – 1.16 (m, 9H), 1.04 (t, *J* = 7.9 Hz, 9H), 0.89 (t, *J* = 7.1 Hz, 3H), 0.75 (q, *J* = 7.9 Hz, 6H).**^{13}C NMR** (151 MHz, $CDCl_3$) δ/ppm = 143.2, 128.9, 128.2, 125.7, 37.6, 32.0, 30.8, 30.1, 29.7, 29.4, 29.1, 27.6, 22.8, 14.3, 9.4, 3.8. **HRMS** (ESI) calculated for $C_{20}H_{35}Ge$: 349.1945 $[M-CH_2CH_3]^+$, found: 349.1943. **IR** (neat): ν/ cm^{-1} = 2923, 2858, 1494, 1457, 1014.

3.4 Different Hydrofunctionalization Reagents

Tributyl(1-phenylbutan-2-yl)germane (1b)

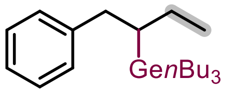

Prepared, following the general procedure A from but-3-en-1-ylbenzene (39.7 mg, 0.30 mmol) with LiO^{t}Bu (2.4 mg, 0.03 mmol, 0.1 equiv.), CsF (4.6 mg, 0.03 mmol, 0.1 equiv.) and tributylgermanium hydride (110.2 mg, 0.45 mmol, 1.5 equiv.). The title product was obtained after purification by flash chromatography on silica gel (*n*-pentane) as a colorless oil (94.1 mg, 0.25 mmol, 83%).

R$_f$ = 0.81 (*n*-pentane). **^{1}H NMR** (600 MHz, $CDCl_3$) δ/ppm = 7.29 – 7.24 (m, 2H), 7.20 – 7.15 (m, 3H), 2.79 (dd, *J* = 13.9, 5.3 Hz, 1H), 2.62 (dd, *J* = 13.9, 10.2 Hz, 1H), 1.51 – 1.40 (m, 2H), 1.40 – 1.28 (m, 13H), 0.90 (t, *J* = 7.0 Hz, 9H), 0.87 (t, *J* = 7.4 Hz, 3H), 0.76 – 0.69 (m, 6H). **^{13}C NMR** (151 MHz, $CDCl_3$) δ/ppm = 143.2, 129.0, 128.2, 125.7, 37.1, 29.9, 27.8, 27.0, 23.4, 13.9, 13.7, 12.4. **HRMS** (EI) calculated for $C_{18}H_{31}Ge$: 321.1632 $[M-(CH_2)_3CH_3]^+$, found: 321.1631. **IR** (neat): ν/ cm^{-1} = 2956, 2858, 1457, 1079.

Trimethyl(1-phenylbutan-2-yl)germane (1c)

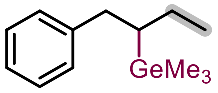

Prepared, following the general procedure A from but-3-en-1-ylbenzene (39.7 mg, 0.30 mmol) with LiO^{t}Bu (2.4 mg, 0.03 mmol, 0.1 equiv.), CsF (4.6 mg, 0.03 mmol, 0.1 equiv.) and hexamethyldigermane (106.0 mg, 0.45 mmol, 1.5 equiv.). The title product was obtained after purification by flash chromatography on silica gel (*n*-pentane) as a colorless oil (42.3 mg, 0.17 mmol, 56%).

R$_f$ = 0.71 (*n*-pentane). **^{1}H NMR** (600 MHz, $CDCl_3$) δ/ppm = 7.28 – 7.24 (m, 2H), 7.19 – 7.14 (m, 3H), 2.75 (dd, *J* = 13.9, 6.2 Hz, 1H), 2.62 (dd, *J* = 13.9, 9.7 Hz, 1H), 1.48 – 1.42 (m, 2H), 1.28 – 1.22 (m, 1H), 0.88 (t, *J* = 7.4 Hz, 3H), 0.08 (s, 9H). **^{13}C NMR** (151 MHz, $CDCl_3$) δ/ppm = 142.9, 129.0, 128.3, 125.7, 36.8, 31.9, 23.3, 13.3, -2.5. **HRMS** (ESI) calculated for $C_{12}H_{19}FGe$: 237.0693 $[M-CH_3]^+$, found:237.0691. **IR** (neat): ν/ cm^{-1} = 2963, 2859, 1454, 1234, 819.

Attempted Hydrosilylation and Hydroboration (1d, 1e)

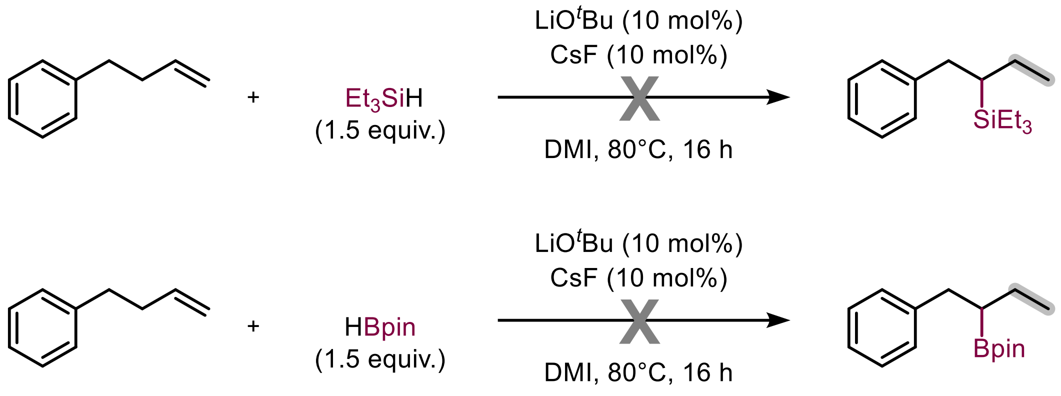

In an argon-filled glovebox, an 8 mL screw-capped glass vial equipped with a magnetic stir bar was charged with LiO^{t}Bu (10 mol%) and CsF (10 mol%) and dissolved in anhydrous DMI (3.0 mL, 0.1 M). To the mixture were added but-3-en-1-ylbenzene (39.7 mg, 0.30 mmol) and the hydrofunctionalization reagent Et_3SiH or HBpin (0.45 mmol, 1.5 equiv.). The vial was sealed and brought out of glovebox and stirred in a heating block at 80°C over night. After cooling down to room temperature the crude reaction mixture was analyzed by GC-MS and no desired product formation was observed.

4. Starting Material Synthesis

4.1 Synthesis of Olefins

Note: The following compounds were synthesized as previously reported:

1-allyl-2-(allyloxy)benzene[3], 1-(but-3-en-1-yl)-3-methylbenzene[4], 1-(but-3-en-1-yl)-4-(tert-butyl)benzene[4], 2-(but-3-en-1-yl)naphthalene[5], 1-(but-3-en-1-yl)-4-fluorobenzene[4], 1-(but-3-en-1-yl)-3-methoxybenzene[6], 2-methoxy-6-(3-methylbut-3-en-1-yl)naphthalene[7], (3-methylbut-2-en-1-yl)benzene[8], pent-4-en-2-ylbenzene[9], 2-(but-3-en-1-yl)pyridine[10], pent-4-en-1-ylbenzene[5], hex-5-en-1-ylbenzene[5], hept-6-en-1-ylbenzene[11], oct-7-en-1-ylbenzene[11], non-8-en-1-ylbenzene[12], dec-9-en-1-ylbenzene[11], (1-cyclopropylvinyl)benzene[13].

4.2 General Procedure C

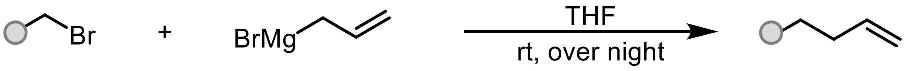

Benzylbromide (2.0-5.0 mmol, 1.0 equiv.) and THF (0.5 M) were added to an oven dried flask under argon atmosphere. Then, allylmagnesiumbromide (1.0 M in 2-Me-THF or Et_2O, 7.5-10.0 mmol, 1.5-2.0 equiv.) was added slowly to the stirring mixture at ambient temperature and left stirring over night. The reaction mixture was quenched by the addition of aqueous NH_4Cl, the organic layer was separated and the aqueous layer extracted three times with Et_2O. The combined organic layers were washed with brine, dried over $MgSO_4$, filtered and concentrated under reduced pressure. The product was obtained after column chromathrophy on silica gel.

4.3 Characterization Data of Alkene Starting Materials

2-allylbenzo[*b*]thiophene (S16)

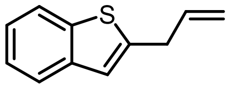

Prepared, following the general procedure C from 2-bromobenzo[*b*]thiophene (1.00 g, 4.70 mmol, 1.0 equiv.) and allylmagnesiumbromide (7.05 mL, 7.05 mmol, 1.5 equiv.). The title compound was obtained after purification by preparative HPLC (*n*-hexane, 20 mL/min) as colorless oil (696.0 mg, 4.00 mmol, 85%).

R_f = 0.78 (*n*-pentane). **^{1}H NMR** (600 MHz, $CDCl_3$) δ/ppm = 7.77 (d, *J* = 8.0 Hz, 1H), 7.68 (d, *J* = 7.9 Hz, 1H), 7.33 – 7.29 (m, 1H), 7.28 – 7.24 (m, 1H), 7.04 (s, 1H), 6.05 (ddtd, *J* = 16.8, 10.0, 6.7, 1.3 Hz, 1H), 5.23 (dt, *J* = 17.0, 1.5 Hz, 1H), 5.17 (dt, *J* = 10.0, 1.4 Hz, 1H), 3.66 (dt, *J* = 6.6, 1.3 Hz, 2H). **^{13}C NMR** (151 MHz, $CDCl_3$) δ/ppm = 144.1, 140.3, 139.8, 135.9, 124.3, 123.7, 123.0, 122.3, 121.2, 117.0, 35.2. **MS** (EI) m/z (%): 174 (100), 147 (66), 129 (14). These data are in agreement with those reported previously in the literature.[14]

3-(But-3-en-1-yl)-1,1'-biphenyl (S28)

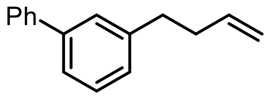

Prepared, following the general procedure C from 3-(bromomethyl)-1,1'-biphenyl (1.235 g, 5.0 mmol, 1.0 equiv.) and allylmagnesiumbromide (7.5 mL, 7.5 mmol, 1.5 equiv.). The title compound was obtained after purification by flash chromatography on silica gel (*n*-pentane) as colorless oil (344.2 mg, 1.65 mmol, 33%).

$\mathbf{R_f}$ = 0.75 (*n*-pentane). **^{1}H NMR** (600 MHz, $CDCl_3$) δ/ppm = 7.61 – 7.58 (m, 2H), 7.46 – 7.41 (m, 4H), 7.38 – 7.32 (m, 2H), 7.19 (d, *J* = 7.5 Hz, 1H), 5.94 – 5.85 (m, 1H), 5.08 (dd, *J* = 17.1, 1.7 Hz, 1H), 5.00 (dd, *J* = 10.2, 1.7 Hz, 1H), 2.79 (t, *J* = 7.9 Hz, 2H), 2.47 – 2.40 (m, 2H). **^{13}C NMR** (151 MHz, $CDCl_3$) δ/ppm = 142.5, 141.5, 141.4, 138.2, 128.8, 127.5, 127.5, 127.3, 127.3, 127.3, 124.9, 115.2, 35.7, 35.6. **MS** (EI) m/z (%): 208 (86) $[M]^+$, 178 (10), 167 (100), 152 (45). These data are in agreement with those reported previously in the literature.[15]

5-(But-3-en-1-yl)benzo[*d*][1,3]dioxole (S32)

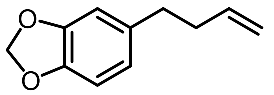

Prepared, following the general procedure C from 5-(bromomethyl) benzo[*d*][1,3]dioxole (430.1 mg, 2.0 mmol, 1.0 equiv.) and allylmagnesium bromide (4.0 mL, 4.0 mmol, 2.0 equiv.). The title compound was obtained after purification by flash chromatography on silica gel (*n*-pentane/Et_2O 97:3) as colorless oil (304.0 mg, 1.73 mmol, 86%).

$\mathbf{R_f}$ = 0.63 (*n*-pentane). **^{1}H NMR** (600 MHz, $CDCl_3$) δ/ppm = 6.73 (d, *J* = 7.9 Hz, 1H), 6.70 – 6.67 (m, 1H), 6.66 – 6.62 (m, 1H), 5.92 (s, 2H), 5.84 (ddt, *J* = 16.9, 10.2, 6.6 Hz, 1H), 5.04 (dd, *J* = 17.1, 1.7 Hz, 1H), 5.00 – 4.95 (m, 1H), 2.63 (dd, *J* = 8.8, 6.7 Hz, 2H), 2.37 – 2.30 (m, 2H). **^{13}C NMR** (151 MHz, $CDCl_3$) δ/ppm = 147.6, 145.7, 138.1, 135.9, 121.3, 115.1, 109.0, 108.2, 100.9, 35.9, 35.3. **MS** (EI) m/z (%): 176 (82), 135 (100), 77 (40). These data are in agreement with those reported previously in the literature.[16]

(4-(But-3-en-1-yl)phenyl)(methyl)sulfane (S33)

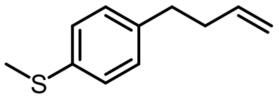

Prepared, following the general procedure C from 4-(methylthio)benzyl bromide (1.08 g, 5.0 mmol, 1.0 equiv.) and allylmagnesiumbromide (10.0 mL, 10.0 mmol, 2.0 equiv.). The title compound was obtained after purification by flash chromatography on silica gel (*n*-pentane/Et_2O 98:2) as colorless oil (879.1 mg, 4.93 mmol, 99%).

$\mathbf{R_f}$ = 0.74 (*n*-pentane). **^{1}H NMR** (600 MHz, $CDCl_3$) δ/ppm = 7.20 (dd, *J* = 8.2, 1.7 Hz, 2H), 7.12 (d, *J* = 7.8 Hz, 2H), 5.84 (ddd, *J* = 16.8, 10.7, 5.6 Hz, 1H), 5.04 (d, *J* = 17.1 Hz, 1H), 4.98 (d, *J* = 10.2 Hz, 1H), 2.67 (t, *J* = 7.8 Hz, 2H), 2.47 (s, 3H), 2.39 – 2.32 (m, 2H). **^{13}C NMR** (151 MHz, $CDCl_3$) δ/ppm = 139.2, 138.1, 135.4, 129.1, 127.3, 115.2, 35.6, 35.0, 16.5. **MS** (EI) m/z (%): 178 (78), 137 (100), 122 (61). These data are in agreement with those reported previously in the literature.[17]

(3-Methylbut-3-en-1-yl)benzene (S34)

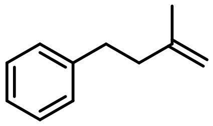

Prepared, following the general procedure C from (bromomethyl)benzene (855.2 mg, 5.0 mmol, 1.0 equiv.) and 2-methylallylmagnesium bromide (0.5 M in THF, 15.0 mL, 7.5 mmol, 1.5 equiv.). The title compound was obtained after

purification by flash chromatography on silica gel (*n*-pentane) as colorless oil (662.7 mg, 4.53 mmol, 91%).

R$_f$ = 0.50 (*n*-pentane). **^{1}H NMR** (600 MHz, $CDCl_3$) δ/ppm = 7.31 – 7.26 (m, 2H), 7.22 – 7.16 (m, 3H), 4.74 (s, 1H), 4.72 (s, 1H), 2.78 – 2.73 (m, 2H), 2.33 (dd, *J* = 9.5, 6.9 Hz, 2H), 1.78 (s, 3H). **^{13}C NMR** (151 MHz, $CDCl_3$) δ/ppm = 145.6, 142.4, 128.5, 128.4, 125.9, 110.3, 39.8, 34.4, 22.8. **HRMS** (APCI) calculated for $C_{11}H_{15}$: 147.1168 $[M+H]^+$, found: 147.1176. These data are in agreement with those reported previously in the literature.[18]

8-(But-3-en-1-yl)quinoline (S39)

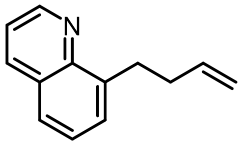

Prepared, following the general procedure C from 8-(bromomethyl)quinoline (1.0 g, 4.5 mmol, 1.0 equiv.) and allylmagnesiumbromide (6.75 mL, 6.75 mmol, 1.5 equiv.). The title compound was obtained after purification by preparative HPLC (*n*-hexane/EtOAc 7:3, 18 mL/min) as colorless oil (305.3 mg, 1.67 mmol, 37%).

R$_f$ = 0.52 (*n*-pentane/Et_2O 9:1). **^{1}H NMR** (600 MHz, $CDCl_3$) δ/ppm = 8.95 (dd, *J* = 4.2, 1.8 Hz, 1H), 8.13 (dd, *J* = 8.2, 1.8 Hz, 1H), 7.67 (dd, *J* = 8.2, 1.5 Hz, 1H), 7.56 (dd, *J* = 7.0, 1.5 Hz, 1H), 7.47 (dd, *J* = 8.1, 7.0 Hz, 1H), 7.39 (dd, *J* = 8.2, 4.1 Hz, 1H), 5.97 (ddt, *J* = 16.9, 10.2, 6.6 Hz, 1H), 5.07 (dd, *J* = 17.1, 1.8 Hz, 1H), 4.99 (dd, *J* = 10.2, 1.7 Hz, 1H), 3.41 – 3.37 (m, 2H), 2.61 – 2.56 (m, 2H). **^{13}C NMR** (151 MHz, $CDCl_3$) δ/ppm = 149.4, 147.0, 140.7, 138.8, 136.5, 129.0, 128.6, 126.4, 126.2, 120.9, 114.8, 34.8, 31.1. **HRMS** (ESI) calculated for $C_{13}H_{14}N$: 184.1121 $[M+H]^+$, found: 184.1119. **IR** (neat): ν/ cm^{-1} = 3070, 2975, 2920, 1639, 1595, 1497.

5. Mechanistic Studies

5.1 Trace Metal Analysis (ICP-MS)

To prove the absence of undesired trace metals in our reagents, which might promote chain walking and hydrogermylation, we analyzed samples of LiO^{t}Bu, CsF, DMI, alkene starting material and trialkyl germanium hydrides using inductively coupled plasma mass spectroscopy (ICP-MS). The results received from the ICP-MS analysis show that the majority of trace metal contaminations lie below the machine's lowest detection limit (values given in nanogram per gram which equals parts per billion (ppb)).

Table S6. ICP-MS trace metal analysis (quantities in ppb).

Element	LiO^{t}Bu (Alfa Aesar)	CsF (chemPUR)	DMI (Sigma)	4-phenyl-1-butene (Sigma)	Et_3GeH (Alfa Aesar)	*n*Bu_3GeH (Sigma)
Silver	< 2	< 2	< 2	< 2	< 20	< 20
Cobalt	< 2	< 2	< 2	< 2	< 20	< 20
Copper	< 2	< 2	< 2	< 2	< 20	< 20
Iron	< 8	< 8	< 8	< 8	< 80	< 80
Iridium	< 2	< 2	< 2	< 2	< 20	< 20
Manganese	< 2	< 2	< 2	< 2	< 20	< 20
Molybdenum	< 2	203	< 2	< 2	< 20	< 20
Nickel	< 2	360	< 2	< 2	< 20	< 20
Palladium	< 2	< 2	< 2	< 2	147	< 20
Platinum	< 2	< 2	< 2	< 2	< 20	< 20
Ruthenium	< 2	< 2	< 2	< 2	< 20	< 20
Titanium	< 2	< 2	< 2	9890	< 20	< 20
Zirconium	1360	39300	< 5	< 5	< 20	< 20

5.2 Control Experiments

5.2.1 Presence of metals

Table S7. Control reactions with metals found in trace analysis.

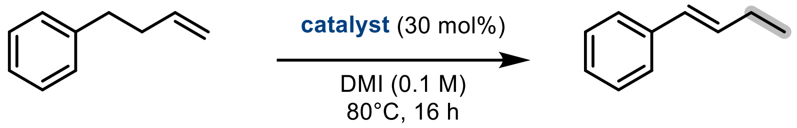

Entry	Catalyst	GC-MS analysis
1	NiF_2	only starting material
2	NiI_2	only starting material
3	$Ti(O^iPr)_4$	only starting material
4	$ZrCl_4$	only starting material
5	$Zr(O^tBu)_4$	only starting material

In an argon-filled glovebox, a 4 mL screw-capped glass vial equipped with a magnetic stir bar was charged with the catalyst (30 mol%) and dissolved in anhydrous DMI (1.0 mL, 0.1 M). To the mixture was added but-3-en-1-ylbenzene (13.2 mg, 0.1 mmol, 1.0 equiv.) and the vial was sealed and brought out of glovebox and stirred in a heating block at 80°C over night. After cooling down to room temperature the reaction mixture was analyzed by GC-MS. All tested catalysts showed no isomerization of the starting material.

5.2.2 Absence of germanium hydride

(*E*)-but-1-en-1-ylbenzene (43)

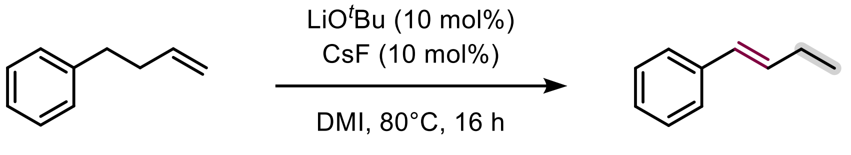

43. 80%, *E/Z* 99:1

Prepared, following the general procedure B from but-3-en-1-ylbenzene (39.7 mg, 0.30 mmol) with LiO^{t}Bu (2.4 mg, 0.03 mmol, 0.1 equiv.), CsF (4.6 mg, 0.03 mmol, 0.1 equiv.). The title product was obtained after purification by flash chromatography on silica gel (*n*-pentane) as a colorless oil (31.7 mg, 0.27 mmol, 80%).

R$_f$ = 0.82 (*n*-pentane). **^{1}H NMR** (600 MHz, $CDCl_3$) δ/ppm = 7.35 (d, *J* = 7.5 Hz, 2H), 7.29 (dd, *J* = 7.6 Hz, 2H), 7.19 (dd, *J* = 7.3 Hz, 1H), 6.38 (d, *J* = 15.8 Hz, 1H), 6.27 (dt, *J* = 15.8, 6.4 Hz, 1H), 2.28 – 2.20 (m, 2H), 1.10 (t, *J* = 7.5 Hz, 3H). **^{13}C NMR** (151 MHz, $CDCl_3$) δ/ppm = 138.1, 132.8, 128.9, 128.6, 126.9, 126.0, 26.2, 13.8. **MS** (EI) m/z (%): 132 (55) $[M]^+$, 117 (100), 91 (20). These data are in agreement with those reported previously in the literature.[19]

Longer chains (44)

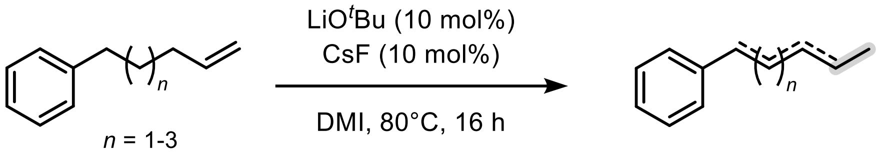

44. mixture of isomers

Reactions were conducted following the general procedure B from either pent-4-en-1-ylbenzene, hex-5-en-1-ylbenzene or hept-6-en-1-ylbenzene (0.30 mmol) with LiO^{t}Bu (2.4 mg, 0.03 mmol, 0.1 equiv.), CsF (4.6 mg, 0.03 mmol, 0.1 equiv.). The crude reaction mixtures were analyzed by GC-MS. In all cases product mixtures were observed and isolation of the products was hence not attempted.

5.2.3 Orthogonal terminal hydrogermylation under Palladium catalysis

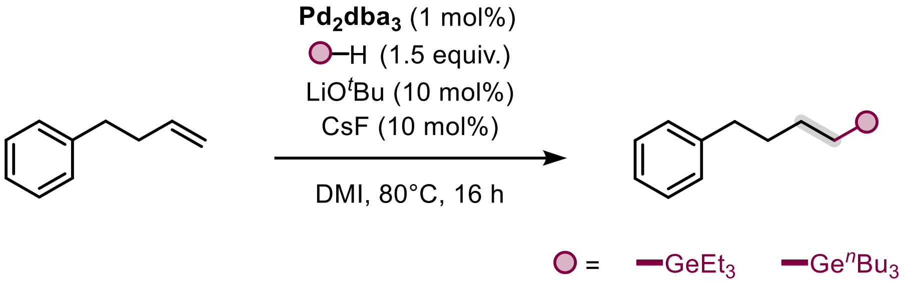

2a. 85% **2b.** 75%

Triethyl(4-phenylbutyl)germane (2a)

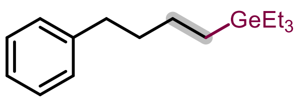

In an argon-filled glovebox, an 8 mL screw-capped glass vial equipped with a magnetic stir bar was charged with LiO^{t}Bu (2.4 mg, 0.03 mmol, 0.1 equiv.) and CsF (4.6 mg, 0.03 mmol, 0.1 equiv.) and Pd_2dba_3 (6.9 mg, 0.008 mmol, 0.025 equiv.) and dissolved in anhydrous DMI (3.0 mL, 0.1 M). To the mixture were added but-3-en-1-ylbenzene (39.7 mg, 0.30 mmol) and Et_3GeH (72.8 µL, 0.45 mmol, 1.5 equiv.). The vial was sealed and brought out of glovebox and stirred in a heating block at 80°C over night. After cooling down to room temperature the crude reaction mixture was directly subjected to flash column chromatography on silica gel (*n*-pentane) to obtain the desired product as a colorless oil (74.3 mg, 0.25 mmol, 85%).

R_f = 0.91 (*n*-pentane). **^{1}H NMR** (600 MHz, $CDCl_3$) δ/ppm = 7.30 – 7.26 (m, 2H), 7.20 – 7.14 (m, 3H), 2.61 (t, *J* = 7.8 Hz, 2H), 1.63 (p, *J* = 7.6 Hz, 2H), 1.42 (p, *J* = 7.7 Hz, 2H), 1.00 (t, *J* = 7.9 Hz, 9H), 0.77 – 0.66 (m, 8H). **^{13}C NMR** (151 MHz, $CDCl_3$) δ/ppm = 143.1, 128.5, 128.4, 125.7, 35.8, 35.6, 25.1, 11.5, 9.1, 4.1. **HRMS** (EI) calculated for $C_{14}H_{23}Ge$: 265.1006 $[M-CH_2CH_3]^+$, found: 265.1007. **IR** (neat): ν/ cm^{-1} = 2925, 1495, 1456, 1015, 696. These data are in agreement with those reported previously in the literature.[20]

Tributyl(4-phenylbutyl)germane (2b)

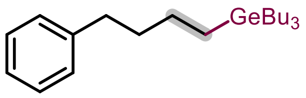

In an argon-filled glovebox, an 8 mL screw-capped glass vial equipped with a magnetic stir bar was charged with LiO^{t}Bu (0.8 mg, 0.01 mmol, 0.1 equiv.) and CsF (1.5 mg, 0.01 mmol, 0.1 equiv.) and Pd_2dba_3 (2.3 mg, 0.003 mmol, 0.025 equiv.) and dissolved in anhydrous DMI (1.0 mL, 0.1 M). To the mixture were added but-3-en-1-ylbenzene (13.2 mg, 0.10 mmol) and $^nBu_3GeH$ (116.2 µL, 0.45 mmol, 1.5 equiv.). The vial was sealed and brought out of glovebox and stirred in a heating block at 80°C over night. After cooling down to room temperature the crude reaction mixture was directly subjected to flash column chromatography on silica gel (*n*-pentane) to obtain the desired product as a colorless oil (28.2 mg, 0.07 mmol, 75%).
R$_f$ = 0.89 (*n*-pentane). **^{1}H NMR** (600 MHz, $CDCl_3$) δ/ppm = 7.29 – 7.26 (m, 2H), 7.19 – 7.15 (m, 3H), 2.62 (t, *J* = 7.8 Hz, 2H), 1.63 (p, *J* = 7.4 Hz, 2H), 1.43 – 1.36 (m, 2H), 1.32 – 1.29 (m, 12H), 0.88 (t, *J* = 7.0 Hz, 9H), 0.75 – 0.65 (m, 8H). **^{13}C NMR** (151 MHz, $CDCl_3$) δ/ppm = 143.1, 128.6, 128.4, 125.7, 35.7, 35.6, 27.7, 26.8, 25.1, 13.9, 12.7, 12.6. **HRMS** (EI) calculated for $C_{18}H_{31}Ge$: 321.1632 $[M-CH_2CH_3]^+$, found: 321.1631. **IR** (neat): ν/ cm^{-1} = 3027, 2870, 1456, 1015, 743.

5.3 Radical Clock Experiment

(2-Cyclopropyl-2-phenylethyl)triethylgermane (45)

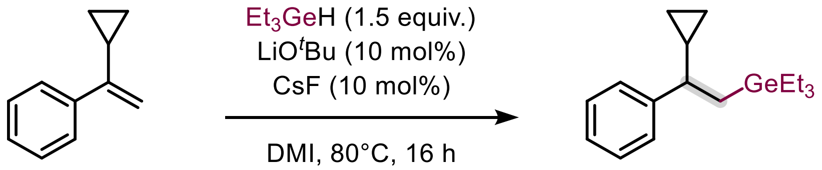

In an argon-filled glovebox, a 4 mL screw-capped glass vial equipped with a magnetic stir bar was charged with LiO^{t}Bu (0.8 mg, 0.01 mmol, 0.1 equiv.) and CsF (1.5 mg, 0.01 mmol, 0.1 equiv.) and dissolved in anhydrous DMI (1.0 mL, 0.1 M). To the mixture were added (1-cyclopropylvinyl)benzene (14.4 mg, 0.10 mmol) and Et_3GeH (24.3 µL, 0.15 mmol, 1.5 equiv.). The vial was sealed and brought out of glovebox and stirred in a heating block at 80°C over night. After cooling down to room temperature the crude reaction mixture was directly subjected to flash column chromatography on silica gel (*n*-pentane) to obtain the desired product as a colorless oil (28.0 mg, 0.09 mmol, 92%).
R$_f$ = 0.90 (*n*-pentane). **^{1}H NMR** (600 MHz, $CDCl_3$) δ/ppm = 7.28 – 7.25 (m, 2H), 7.20 – 7.16 (m, 3H), 1.93 (td, *J* = 9.4, 5.4 Hz, 1H), 1.30 – 1.21 (m, 2H), 1.01 – 0.95 (m, 1H), 0.89 (t, *J* = 7.9 Hz, 9H), 0.58 – 0.45 (m, 7H), 0.37 – 0.31 (m, 1H), 0.22 – 0.15 (m, 1H), 0.11 – 0.05 (m, 1H). **^{13}C NMR** (151 MHz, $CDCl_3$) δ/ppm = 147.7, 128.3, 127.5, 126.1, 48.0, 21.3, 19.4, 9.0, 5.9, 4.5, 4.5. **HRMS** (EI) calculated for $C_{15}H_{23}Ge$: 277.1006 $[M-CH_2CH_3]^+$, found: 277.1004. **IR** (neat): ν/ cm^{-1} = 2947, 2871, 2326, 1455, 1014.

5.4 Deuterium Labeling Studies

Triethyl(2-(phenyl-d_5)ethyl-1,1,2,2-d_4)germane (21-d_9)

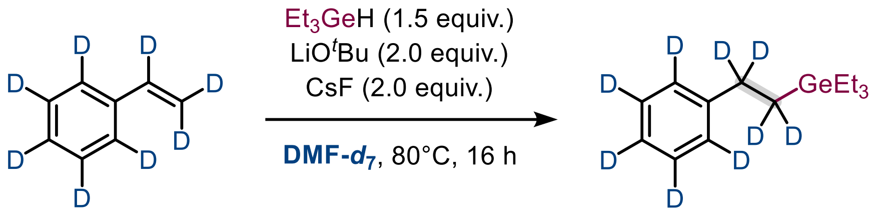

21-*d*₉. 88%

Prepared, following the general procedure A from styrene-d_8 (33.7 mg, 0.30 mmol) with LiO^{t}Bu (48.0 mg, 0.60 mmol, 2.0 equiv.) and CsF (91.1 mg, 0.60 mmol, 2.0 equiv.) in DMF-d_7. The title product was obtained after purification by flash chromatography on silica gel (*n*-pentane) as a colorless oil (72.4 mg, 0.27 mmol, 88%).

R$_f$ = 0.85 (*n*-pentane). **^{1}H NMR** (600 MHz, $CDCl_3$) δ/ppm = 1.03 (t, *J* = 7.9 Hz, 9H), 0.74 (q, *J* = 7.9 Hz, 6H). **^{13}C NMR** (151 MHz, $CDCl_3$) δ/ppm = 145.4, 127.9 (t, *J* = 24.2 Hz), 127.5 (t, *J* = 23.7 Hz), 125.1 (t, *J* = 24.2 Hz), 30.5 (p, *J* = 19.7, 19.1, 15.9 Hz), 12.7 (p, *J* = 19.2, 18.8 Hz), 9.1, 4.0. **HRMS** (APCI) calculated for $C_{12}H_{10}{}^2H_9Ge$: 246.1258 $[M\text{-}CH_2CH_3]^+$, found: 246.1258. **IR** (neat): ν/ cm^{-1} = 2948, 2906, 1458, 1379, 1015.

Triethyl(2-(phenyl-d_5)ethyl-1,1-d_2)germane (21-d_7)

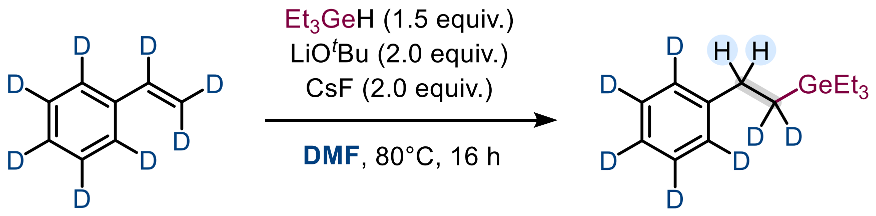

21-*d*₇. 85%

Prepared, following the general procedure A from styrene-d_8 (33.7 mg, 0.30 mmol) with LiO^{t}Bu (48.0 mg, 0.60 mmol, 2.0 equiv.) and CsF (91.1 mg, 0.60 mmol, 2.0 equiv.) in DMF. The title product was obtained after purification by flash chromatography on silica gel (*n*-pentane) as a colorless oil (71.8 mg, 0.26 mmol, 85%).

R$_f$ = 0.87 (*n*-pentane). **^{1}H NMR** (600 MHz, $CDCl_3$) δ/ppm = 2.67 (s, 2H), 1.04 (t, *J* = 7.9 Hz, 9H), 0.75 (q, *J* = 7.9 Hz, 6H). **^{13}C NMR** (151 MHz, $CDCl_3$) δ/ppm = 145.4, 127.9 (t, *J* = 23.6 Hz), 127.5 (t, *J* = 23.6 Hz), 125.1 (t, *J* = 24.3, 23.5 Hz), 31.2, 12.9 (p, *J* = 20.0, 19.3, 11.2 Hz), 9.1, 4.0. **HRMS** (EI) calculated for $C_{12}H_{12}{}^2H_7Ge$: 244.1132 $[M\text{-}CH_2CH_3]^+$, found: 244.1137. **IR** (neat): ν/ cm^{-1} = 2948, 2870, 1457, 1379, 1014.

6. Computed pK_a Values

For calculation of the pK_a, first the CREST package[21] in xTB[22] was used to explore conformational space for both the neutral molecules as well as their anions (after deprotonation). If more than 10 conformers were obtained the clustering option of CREST was utilized to select the most representative conformers. The number of representatives (i.e. clusters) was initially not specified, but set to 10 in those cases where more than 10 clusters/representatives were formed.

The selected conformers were then further optimized at the DFT level using the Gaussian 16 program package (revision A.03).[23] Geometry optimizations were carried out using M06-2X and def2-TZVPP basis set on all atoms and accounting for solvation using the SMD model for DMSO. The minimum energy conformer of each structure was then used to calculate the pK_a using COSMOTherm.[24] Input structures for COSMOtherm were generated from single-point energy calculations at the BP86/def2-TZVPD level of theory using Turbomole 7.5.1.[25] Figures were created using CYLView.[26]

6.1 XYZ Coordinates and Energies of Optimized Structures

DMI neutral

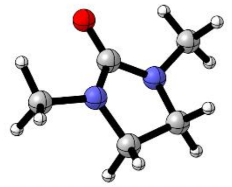

C	0.75668	1.48722	-0.09968
C	-0.00000	-0.70039	-0.00001
C	-0.75668	1.48722	0.09968
H	1.27224	2.17914	0.56323
H	1.02121	1.72287	-1.13656
H	-1.27223	2.17915	-0.56323
H	-1.02120	1.72287	1.13656
N	1.09193	0.10699	0.20731
N	-1.09193	0.10699	-0.20730
C	2.43048	-0.37802	-0.01922
H	3.13200	0.17576	0.60395
H	2.47563	-1.43117	0.24700
H	2.72718	-0.26193	-1.06730
C	-2.43048	-0.37802	0.01922
H	-3.13201	0.17586	-0.60385
H	-2.47567	-1.43113	-0.24713
H	-2.72714	-0.26206	1.06733
O	-0.00001	-1.92192	0.00000

Zero-point correction = 0.156473 (Hartree/Particle)
Thermal correction to Energy = 0.164804
Thermal correction to Enthalpy = 0.165748
Thermal correction to Gibbs Free Energy = 0.123708
Sum of electronic and zero-point Energies = -381.142907
Sum of electronic and thermal Energies = -381.134576
Sum of electronic and thermal Enthalpies = -381.133631
Sum of electronic and thermal Free Energies = -381.175672

DMI anion

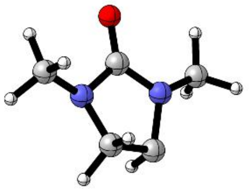

C	-0.73832	1.60627	-0.07962
C	-0.04982	-0.65695	-0.05086
C	0.76233	1.48962	0.15002
H	-0.85821	1.75061	-1.17467
H	1.04314	1.59688	1.21182
H	1.34230	2.21299	-0.42535
N	-1.09253	0.18466	0.15990
N	1.08338	0.11439	-0.25995
C	-2.44814	-0.27250	0.06846
H	-2.49227	-1.34130	0.27086
H	-3.06890	0.25733	0.79332
H	-2.86827	-0.09046	-0.92944
C	2.37430	-0.43621	0.06583
H	2.58373	-0.37103	1.14194
H	2.40990	-1.48221	-0.23188
H	3.15467	0.10850	-0.46638
O	-0.07302	-1.88949	-0.05160

Zero-point correction = 0.141552 (Hartree/Particle)
Thermal correction to Energy = 0.149690
Thermal correction to Enthalpy = 0.150635
Thermal correction to Gibbs Free Energy = 0.109129
Sum of electronic and zero-point Energies = -380.597378
Sum of electronic and thermal Energies = -380.589240
Sum of electronic and thermal Enthalpies = -380.588296
Sum of electronic and thermal Free Energies = -380.629801

DMF neutral

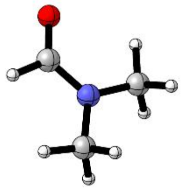

O	1.94811	-0.08192	-0.00003
N	-0.33320	-0.02324	0.00016
C	-1.57146	-0.76964	-0.00005
H	-1.35816	-1.83657	0.00046
H	-2.16123	-0.52526	0.88559
H	-2.16053	-0.52581	-0.88628
C	-0.44188	1.41899	-0.00002
H	0.55383	1.85179	-0.00005
H	-0.98489	1.75341	-0.88611
H	-0.98490	1.75366	0.88597
C	0.85939	-0.63622	-0.00000
H	0.76711	-1.73201	-0.00005

Zero-point correction = 0.103250 (Hartree/Particle)
Thermal correction to Energy = 0.109323
Thermal correction to Enthalpy = 0.110267
Thermal correction to Gibbs Free Energy = 0.074095
Sum of electronic and zero-point Energies = -248.406191
Sum of electronic and thermal Energies = -248.400119
Sum of electronic and thermal Enthalpies = -248.399174
Sum of electronic and thermal Free Energies = -248.435346

DMF anion

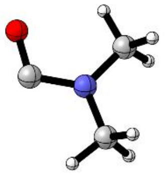

O	1.92747	-0.16129	-0.00001
N	-0.31271	-0.06504	0.00006
C	-0.33531	1.37866	-0.00001
H	-0.85055	1.77308	0.88333
H	0.69409	1.73171	0.00004
H	-0.85042	1.77301	-0.88346
C	-1.59882	-0.70304	-0.00001
H	-2.18998	-0.43075	-0.88305
H	-1.45611	-1.78395	0.00008
H	-2.19013	-0.43062	0.88287
C	0.86952	-0.82342	-0.00001

Zero-point correction = 0.088450 (Hartree/Particle)
Thermal correction to Energy = 0.094513
Thermal correction to Enthalpy = 0.095457
Thermal correction to Gibbs Free Energy = 0.059308
Sum of electronic and zero-point Energies = -247.870571
Sum of electronic and thermal Energies = -247.864508
Sum of electronic and thermal Enthalpies = -247.863564
Sum of electronic and thermal Free Energies = -247.899712

Et_3GeH neutral

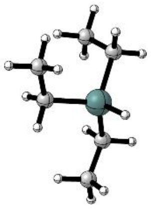

Ge	-0.15435	0.01785	-0.47342
C	0.47289	-1.52908	0.57819
H	0.50159	-1.23464	1.63045
H	-0.26557	-2.32938	0.49812
C	1.84571	-2.02094	0.12210
H	2.60309	-1.24163	0.23047
H	2.18045	-2.88503	0.69988
H	1.83184	-2.31571	-0.92954
C	-1.82261	0.73822	0.29040
H	-1.58924	1.16865	1.26721
H	-2.16405	1.56098	-0.34141
C	-2.91264	-0.32392	0.42584
H	-3.83659	0.09299	0.83204
H	-3.15268	-0.77275	-0.54041
H	-2.59834	-1.13092	1.09052
C	1.26475	1.38692	-0.50251
H	0.89165	2.26339	-1.03657
H	2.10748	1.00303	-1.08138
C	1.71492	1.76829	0.90734
H	0.88646	2.16852	1.49546
H	2.10791	0.90331	1.44626
H	2.50006	2.52702	0.89040
H	-0.44297	-0.46617	-1.93018

Zero-point correction = 0.203756 (Hartree/Particle)
Thermal correction to Energy = 0.215678
Thermal correction to Enthalpy = 0.216622
Thermal correction to Gibbs Free Energy = 0.165277
Sum of electronic and zero-point Energies = -2315.047985
Sum of electronic and thermal Energies = -2315.036064
Sum of electronic and thermal Enthalpies = -2315.035120
Sum of electronic and thermal Free Energies = -2315.086464

Et_3GeH anion

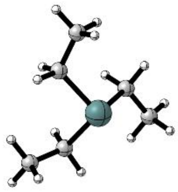

Ge	-0.00013	-0.00091	-0.59230
C	-1.75198	-0.25512	0.43170
H	-1.57163	-0.06000	1.49645
H	-2.48403	0.48817	0.10104
C	-2.33451	-1.65630	0.25483
H	-1.65220	-2.41945	0.63797
H	-3.29064	-1.79451	0.76917
H	-2.50150	-1.88372	-0.80228
C	0.65579	1.64555	0.42836
H	0.73957	1.39211	1.49283
H	1.66352	1.91057	0.09390
C	-0.26967	2.84884	0.25580
H	-0.38817	3.10712	-0.80077
H	-1.26956	2.63734	0.64341
H	0.08948	3.74647	0.76877
C	1.09817	-1.38890	0.43182
H	0.82260	-2.39545	0.10197
H	0.83911	-1.33035	1.49656
C	2.60260	-1.19082	0.25535
H	3.20068	-1.94994	0.76907
H	2.88338	-1.22034	-0.80175
H	2.92118	-0.21854	0.63987

Zero-point correction = 0.193518 (Hartree/Particle)
Thermal correction to Energy = 0.205255
Thermal correction to Enthalpy = 0.206200
Thermal correction to Gibbs Free Energy = 0.155627
Sum of electronic and zero-point Energies = -2314.535289
Sum of electronic and thermal Energies = -2314.523552
Sum of electronic and thermal Enthalpies = -2314.522607
Sum of electronic and thermal Free Energies = -2314.573180

***tert*-Butanol neutral**

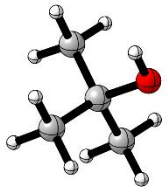

C	0.00780	-0.00000	0.01668
C	1.49372	-0.00059	-0.29502
H	1.96953	-0.88676	0.12758
H	1.65580	-0.00057	-1.37333
H	1.97023	0.88515	0.12770
C	-0.65969	1.25279	-0.53574
H	-0.57991	1.28845	-1.62330
H	-1.72020	1.26737	-0.27269
H	-0.18839	2.14578	-0.12263
C	-0.66072	-1.25218	-0.53588
H	-1.72125	-1.26591	-0.27282
H	-0.58098	-1.28779	-1.62344
H	-0.19016	-2.14561	-0.12288
O	-0.08520	-0.00005	1.44681
H	-1.01971	0.00020	1.68101

Zero-point correction = 0.135980 (Hartree/Particle)
Thermal correction to Energy = 0.142652
Thermal correction to Enthalpy = 0.143596
Thermal correction to Gibbs Free Energy = 0.107057
Sum of electronic and zero-point Energies = -233.527544
Sum of electronic and thermal Energies = -233.520872
Sum of electronic and thermal Enthalpies = -233.519928
Sum of electronic and thermal Free Energies = -233.556467

***tert*-Butanol anion**

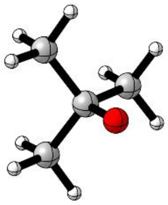

C	0.00025	-0.00026	0.14088
C	-1.14243	-0.87640	-0.43075
H	-1.02609	-1.90190	-0.06892
H	-2.10175	-0.50033	-0.06416
H	-1.17739	-0.89976	-1.52581
C	1.32947	-0.54947	-0.43457
H	1.36670	-0.56305	-1.52969
H	2.15896	0.06301	-0.06991
H	1.48387	-1.56986	-0.07253
C	-0.18975	1.42711	-0.43061
H	0.61590	2.07081	-0.06627
H	-0.19562	1.46904	-1.52560
H	-1.13545	1.83808	-0.06586
O	0.00320	-0.00149	1.48988

Zero-point correction = 0.121510 (Hartree/Particle)
Thermal correction to Energy = 0.127779
Thermal correction to Enthalpy = 0.128724
Thermal correction to Gibbs Free Energy = 0.092926
Sum of electronic and zero-point Energies = -233.009417
Sum of electronic and thermal Energies = -233.003148
Sum of electronic and thermal Enthalpies = -233.002204
Sum of electronic and thermal Free Energies = -233.038001

Triethyl(1-phenylbutan-2-yl)germane neutral

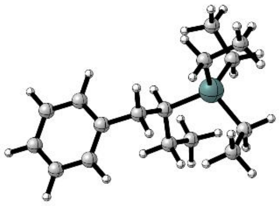

C	-4.47067	-1.47937	-0.37467
C	-3.11199	-1.27629	-0.57239
C	-2.57798	0.01202	-0.62083
C	-3.44344	1.09220	-0.46480
C	-4.80551	0.89515	-0.26591
C	-5.32377	-0.39205	-0.21960
H	-4.86636	-2.48673	-0.34494
H	-2.45223	-2.12811	-0.69549
H	-3.04405	2.09949	-0.49939
H	-5.46128	1.74838	-0.14705
H	-6.38363	-0.54849	-0.06634
C	-1.09428	0.21141	-0.80279
H	-0.75877	-0.41076	-1.63563
H	-0.90500	1.25086	-1.08566
C	-0.27358	-0.13748	0.45431
H	-0.47023	-1.18774	0.70165
C	-0.67616	0.71930	1.65722
H	-0.46526	1.77239	1.43944
H	-1.75846	0.65213	1.80606
C	0.01593	0.31653	2.95370
H	-0.36965	0.88867	3.79875
H	-0.14709	-0.74297	3.16510
H	1.09369	0.48307	2.91049
C	2.33553	1.81870	0.46428
H	2.08801	2.06381	1.49973
H	3.42693	1.80138	0.40490
C	1.77189	2.87875	-0.48196
H	2.15864	3.87451	-0.25360
H	2.02685	2.66050	-1.52136
H	0.68268	2.92969	-0.41941
C	1.99875	-0.35505	-1.86131
H	1.57412	-1.32928	-2.11694
H	1.45410	0.38436	-2.45304
C	3.48824	-0.31574	-2.20154
H	4.04591	-1.06534	-1.63518
H	3.67134	-0.50554	-3.26149
H	3.92337	0.65819	-1.96482
C	2.71162	-1.35614	1.07271
H	2.48216	-1.26178	2.13580
H	3.77389	-1.12277	0.96216
C	2.43043	-2.77899	0.59093
H	1.37017	-3.02754	0.67877
H	2.70675	-2.90434	-0.45826
H	2.98764	-3.52182	1.16597
Ge	1.68018	-0.00444	0.06017

Zero-point correction = 0.400931 (Hartree/Particle)
Thermal correction to Energy = 0.422943
Thermal correction to Enthalpy = 0.423887
Thermal correction to Gibbs Free Energy = 0.348378
Sum of electronic and zero-point Energies = -2703.138742
Sum of electronic and thermal Energies = -2703.116731
Sum of electronic and thermal Enthalpies = -2703.115787
Sum of electronic and thermal Free Energies = -2703.191296

Triethyl(1-phenylbutan-2-yl)germane anion

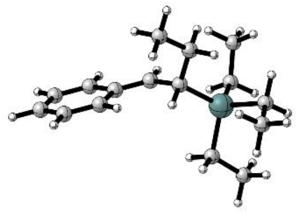

C	1.13849	0.10511	0.69757
H	0.87008	-0.24630	1.69267
C	0.06423	0.79097	-0.10458
H	0.30717	0.72212	-1.17322
C	-0.14859	2.28710	0.20524
H	-1.02287	2.66038	-0.34003
H	-0.37706	2.40547	1.26913
C	1.05711	3.15077	-0.14922
H	0.89776	4.19647	0.12209
H	1.95205	2.80186	0.37021
H	1.25705	3.11125	-1.22269
C	-1.99352	-0.74254	1.94731
H	-2.98106	-1.21174	1.96944
H	-1.28071	-1.51068	2.25747
C	-1.95304	0.43928	2.91605
H	-2.63080	1.23716	2.60317
H	-2.24059	0.14647	3.92874
H	-0.95122	0.86905	2.97268
C	-1.46068	-1.91517	-0.99787
H	-1.11699	-1.65504	-2.00251
H	-0.67534	-2.53148	-0.55276
C	-2.77238	-2.69571	-1.07181
H	-3.55819	-2.10593	-1.54951
H	-2.66838	-3.62165	-1.64231
H	-3.13129	-2.96619	-0.07610
C	-3.16658	0.76536	-0.64492
H	-3.27896	1.69089	-0.07474
H	-4.07493	0.18230	-0.47041
C	-3.00996	1.07272	-2.13388
H	-2.93624	0.15413	-2.72103
H	-3.85188	1.64563	-2.52954
H	-2.10285	1.65115	-2.32696
Ge	-1.61734	-0.25129	0.06523
C	2.43364	-0.15298	0.27427
C	3.38187	-0.86359	1.10715
C	2.97577	0.23830	-1.00802
C	4.66728	-1.13899	0.70783
H	3.05060	-1.19023	2.08837
C	4.27172	-0.05802	-1.38462
H	2.35024	0.79010	-1.69885
C	5.15642	-0.75004	-0.55186
H	5.31945	-1.67568	1.39019
H	4.60984	0.26734	-2.36408
H	6.16935	-0.97105	-0.85861

Zero-point correction = 0.384841 (Hartree/Particle)
Thermal correction to Energy = 0.407222
Thermal correction to Enthalpy = 0.408166
Thermal correction to Gibbs Free Energy = 0.331706
Sum of electronic and zero-point Energies = -2702.613415
Sum of electronic and thermal Energies = -2702.591035
Sum of electronic and thermal Enthalpies = -2702.590090
Sum of electronic and thermal Free Energies = -2702.666550

7. NMR Spectra

7.1 Products

Triethyl(1-phenylbutan-2-yl)germane (1a)

1H NMR
(600.44 MHz, CDCl3)

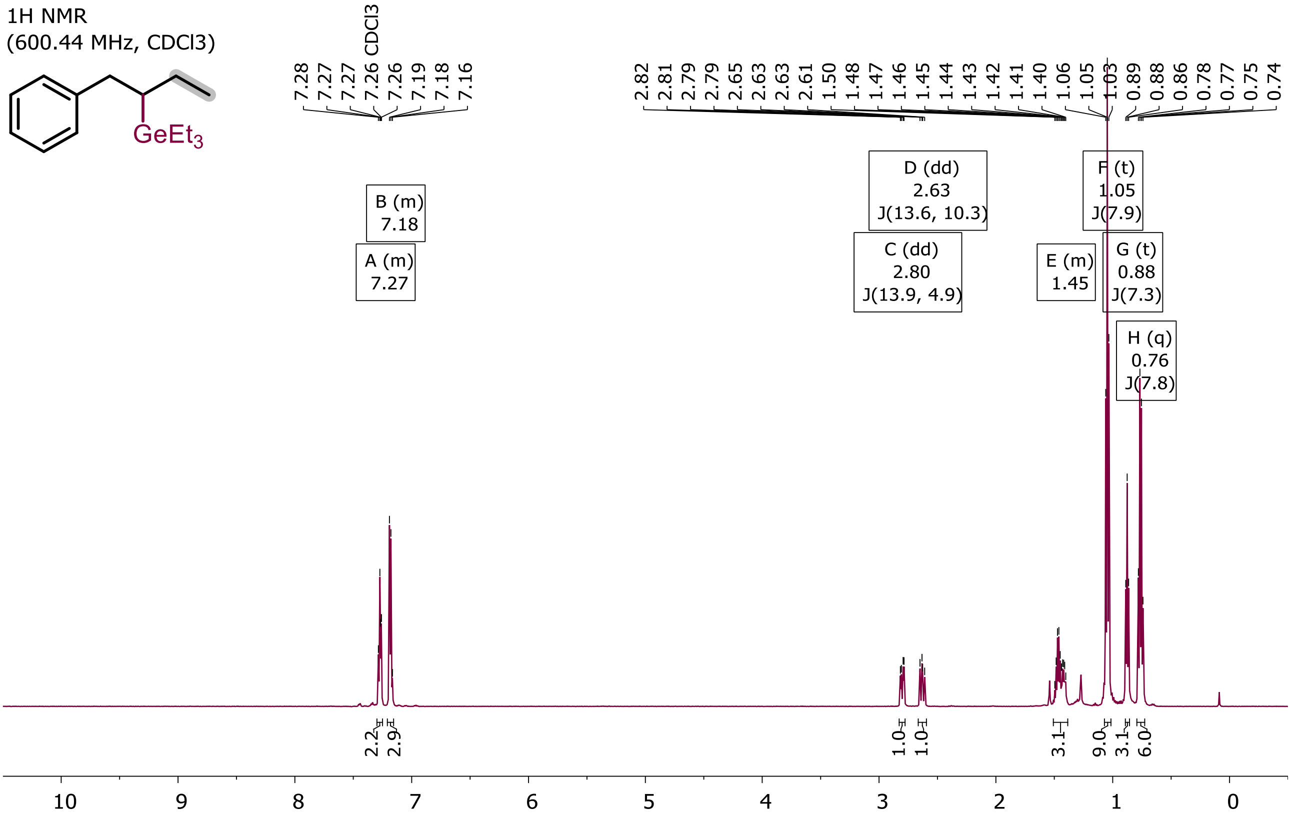

13C NMR
(151.00 MHz, CDCl3)

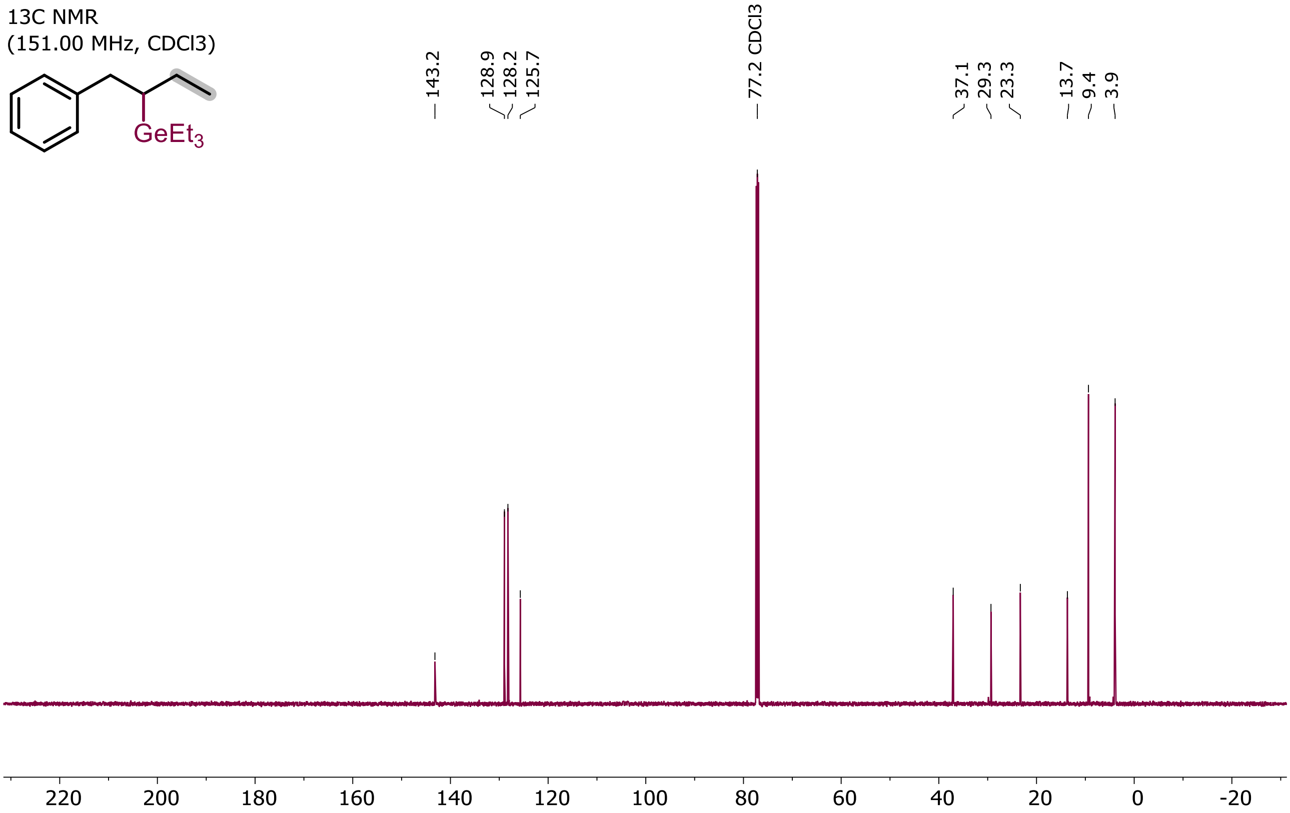

Tributyl(1-phenylbutan-2-yl)germane (1b)

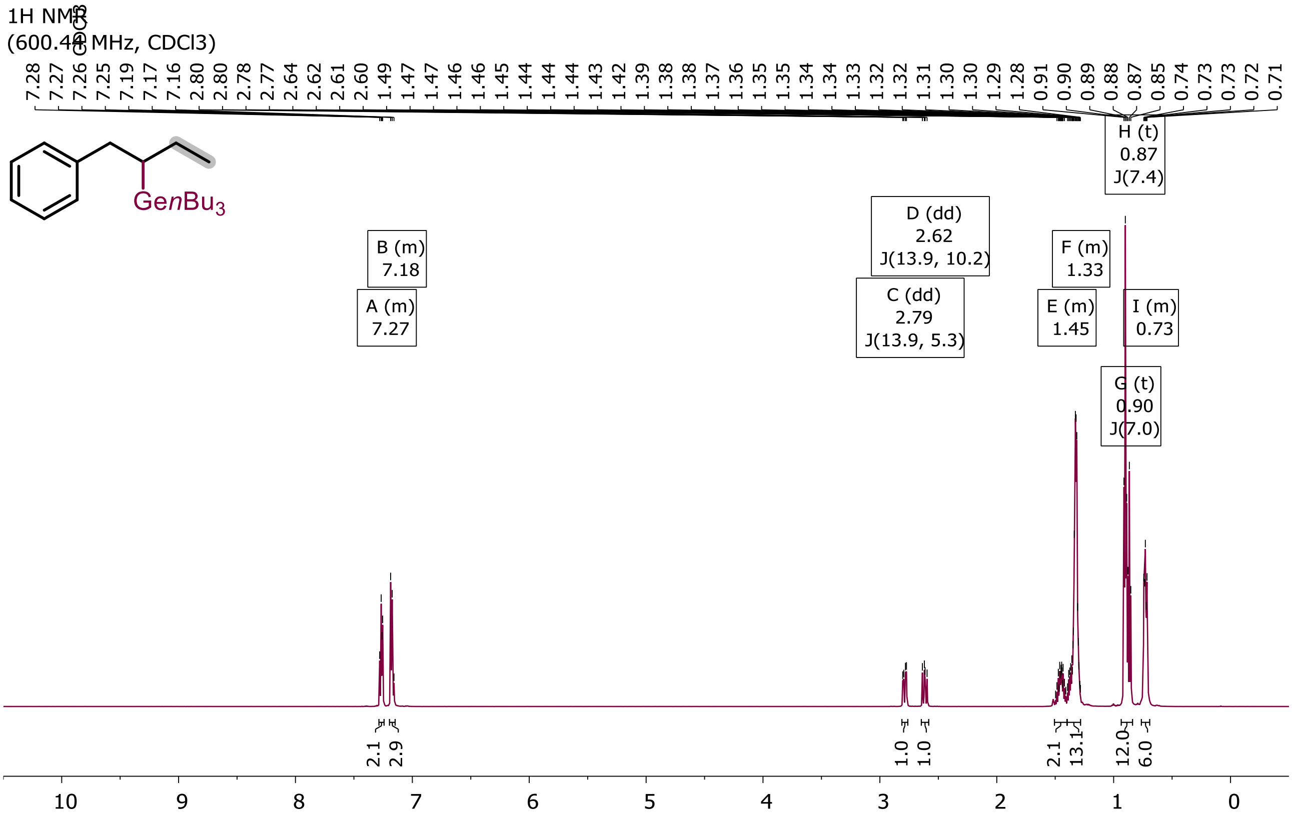

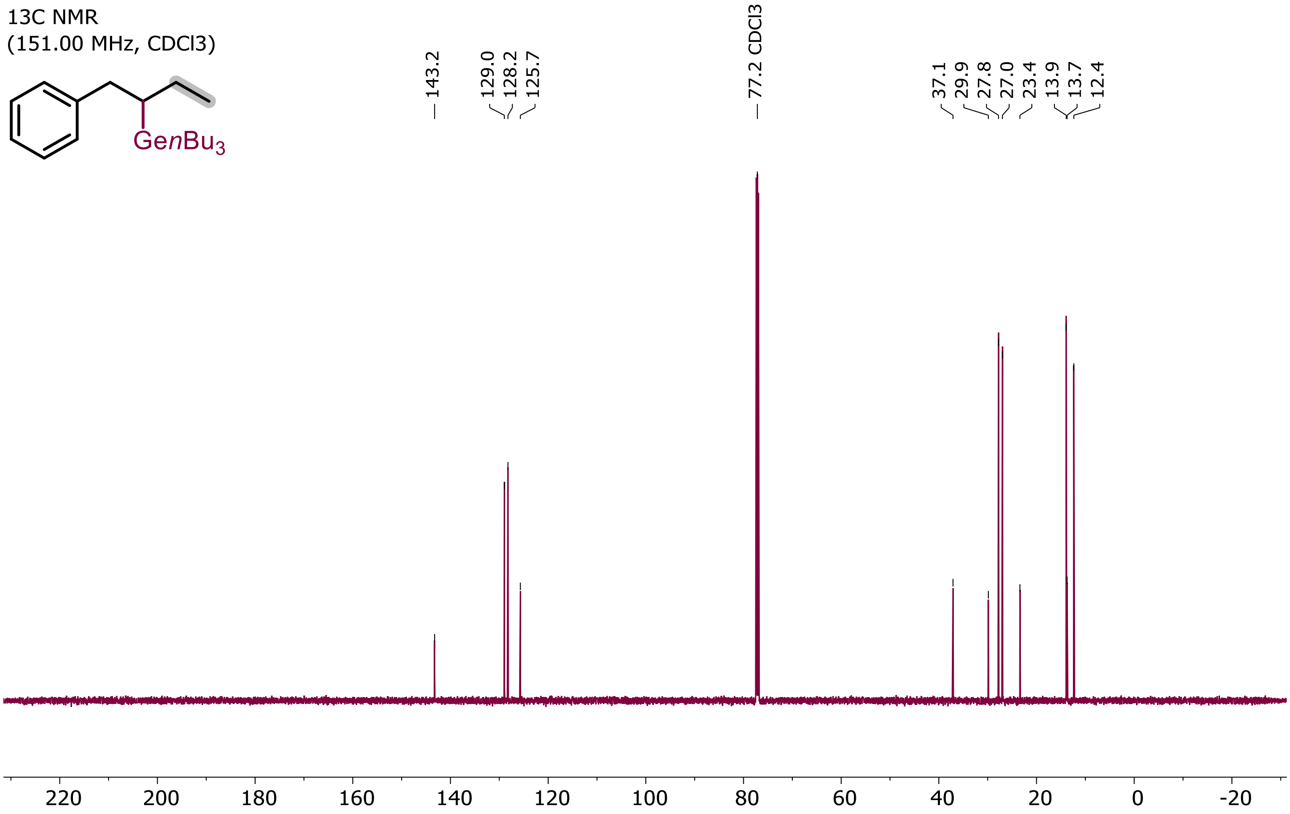

Trimethyl(1-phenylbutan-2-yl)germane (1c)

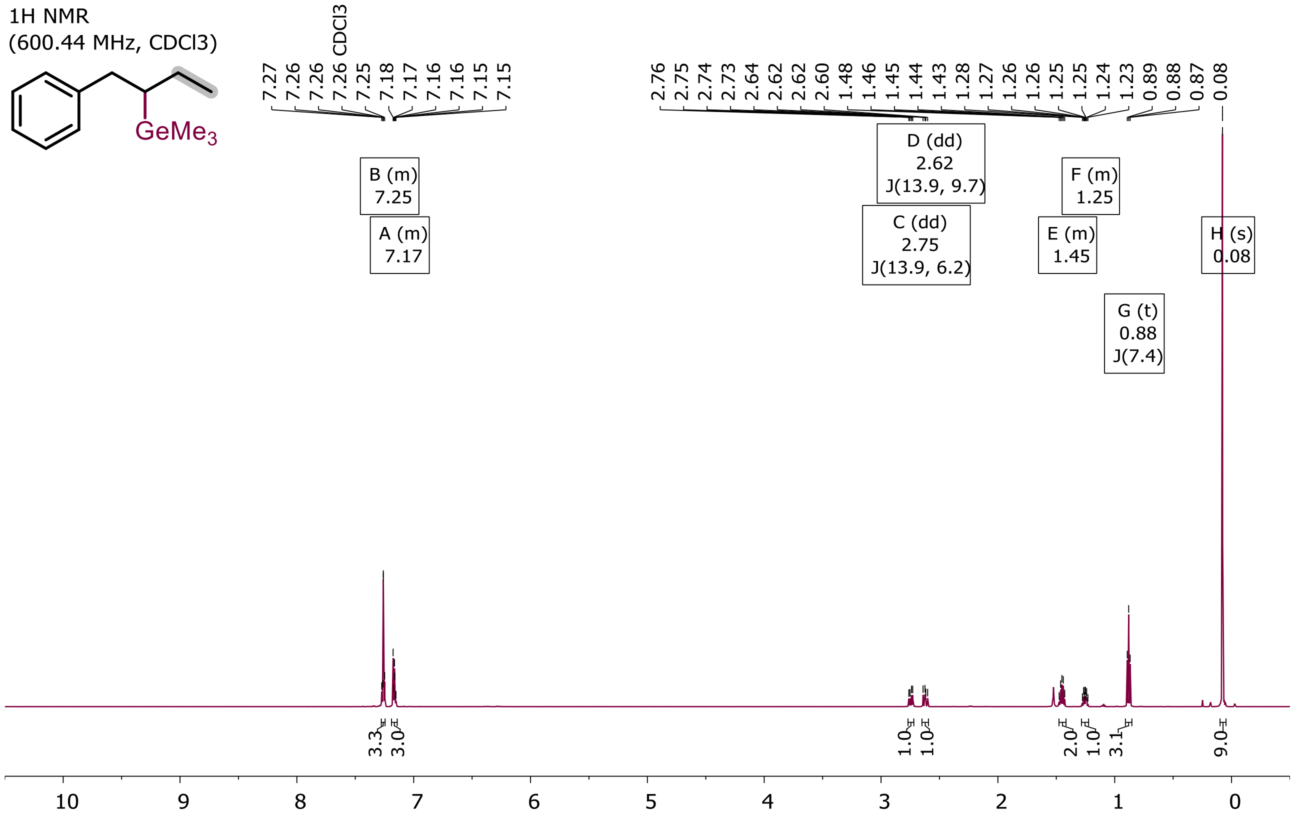

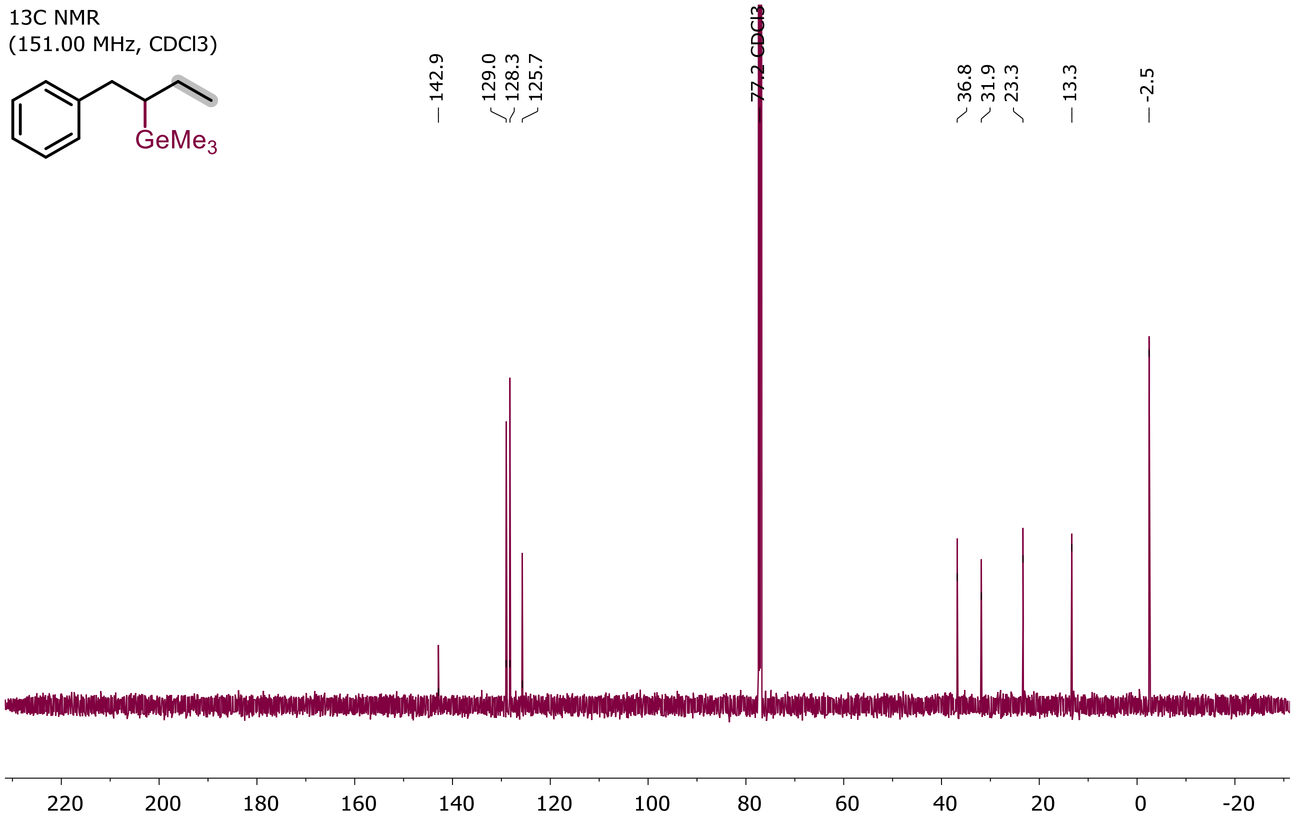

Triethyl(4-phenylbutyl)germane (2a)

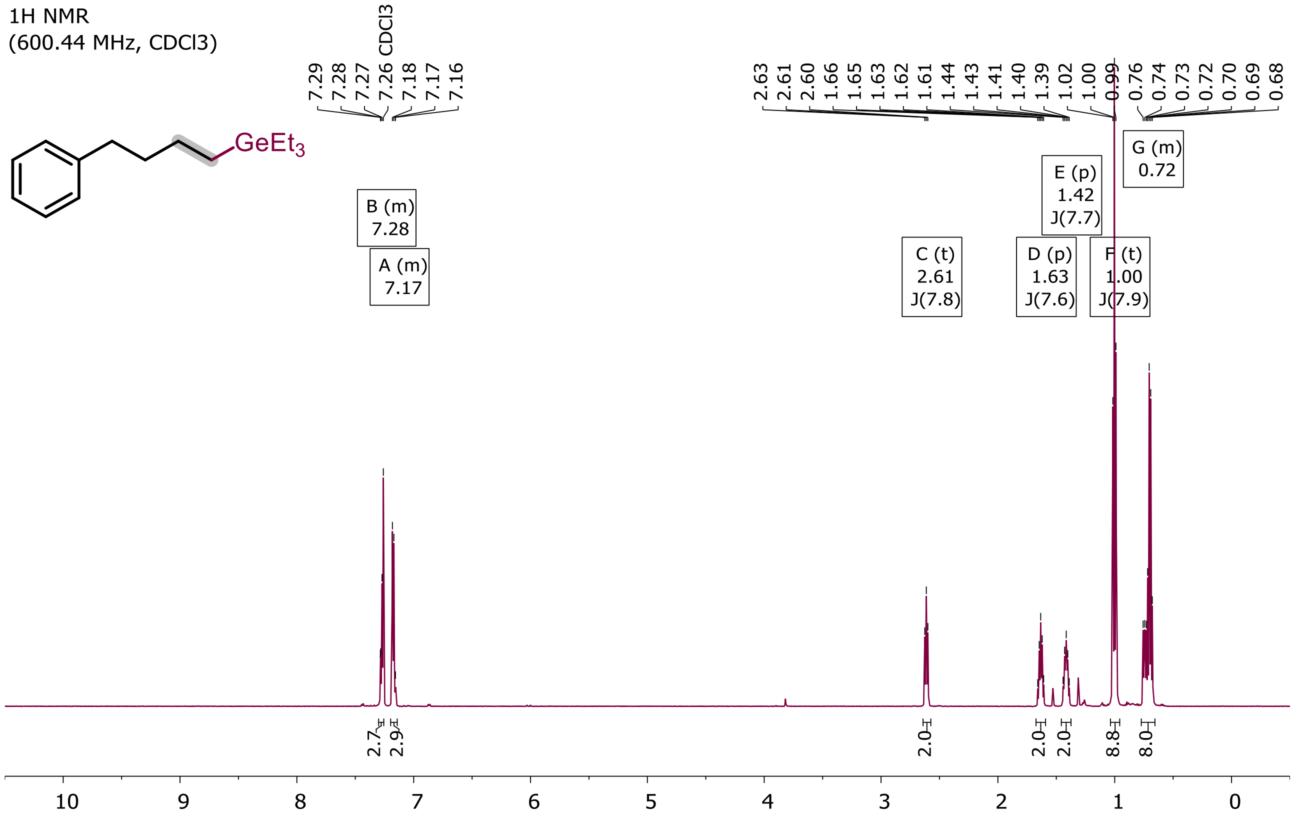

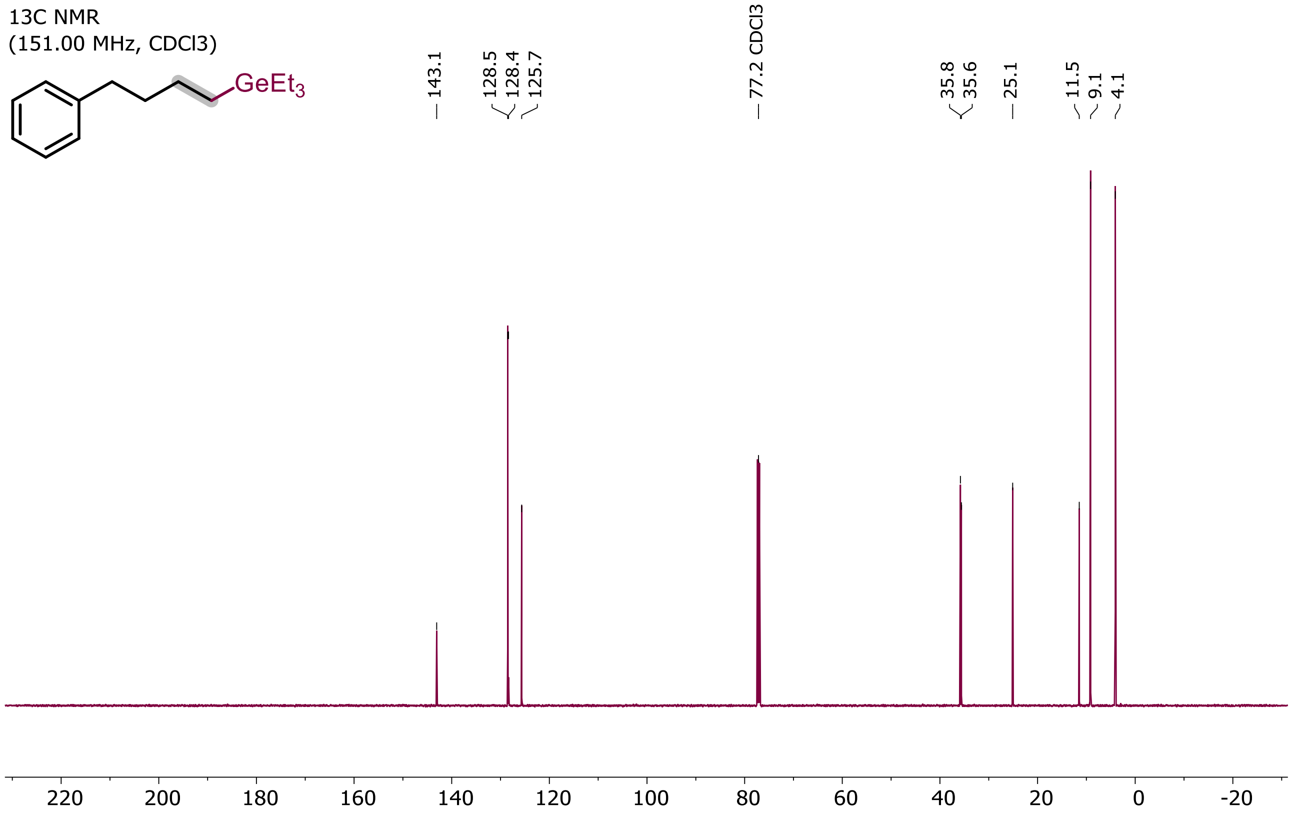

Tributyl(4-phenylbutyl)germane (2b)

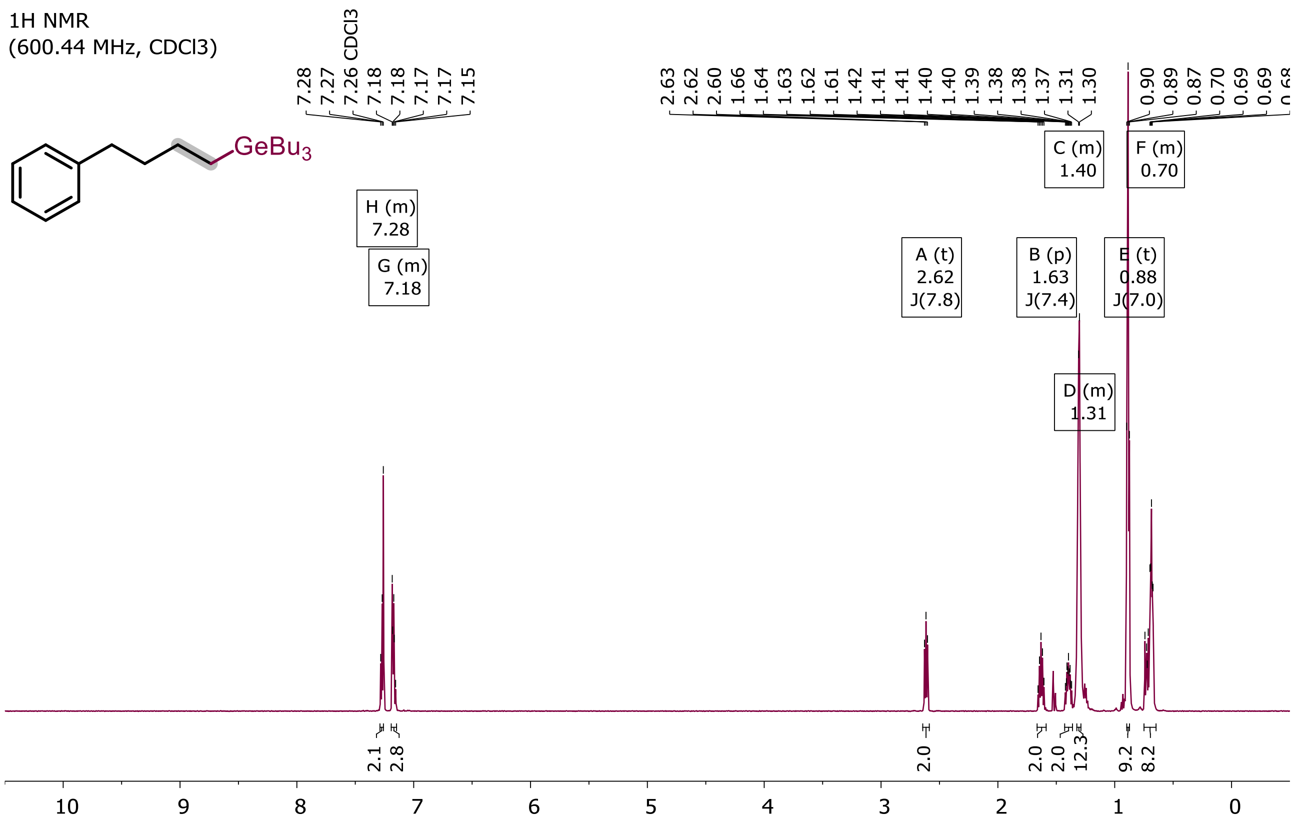

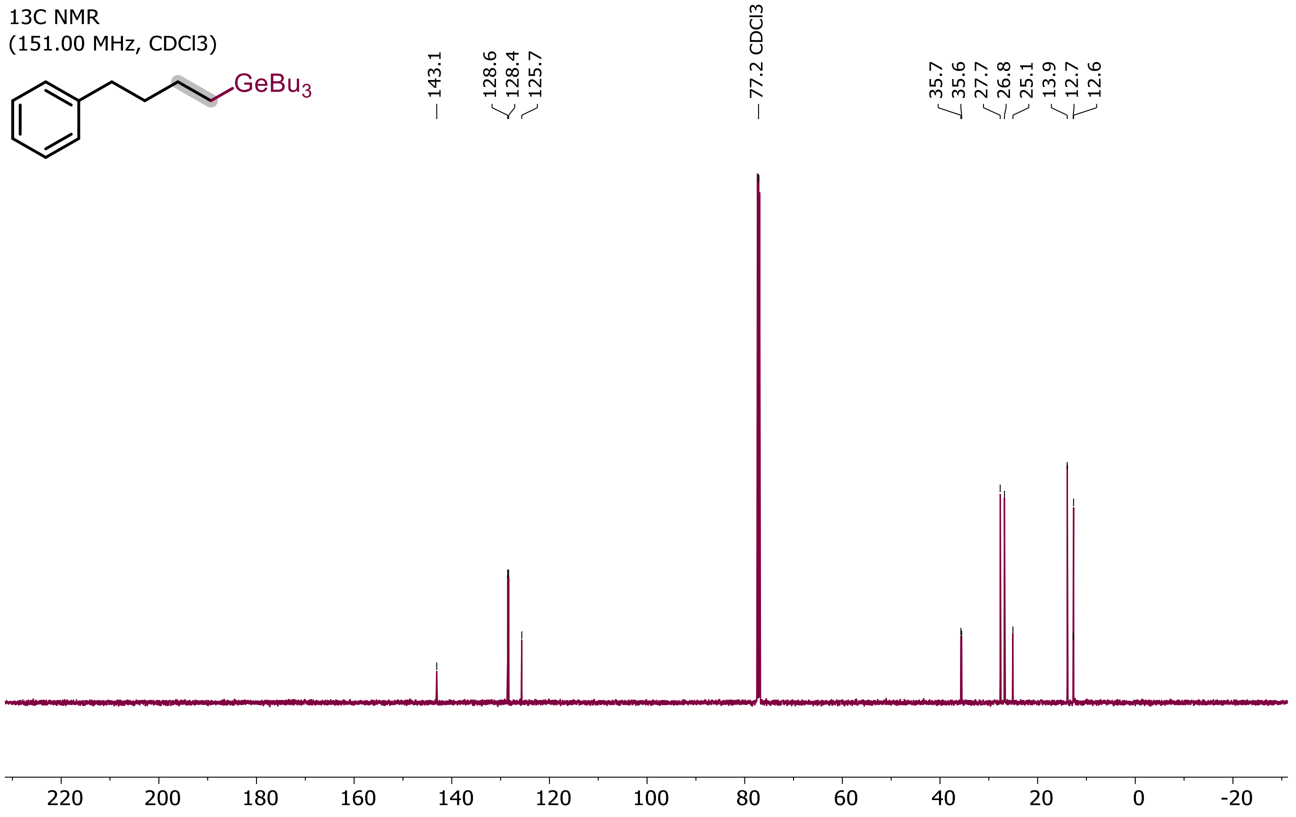

Triethyl(1-(4-methoxyphenyl)propan-2-yl)germane (3)

1H NMR
(600.44 MHz, CDCl3)

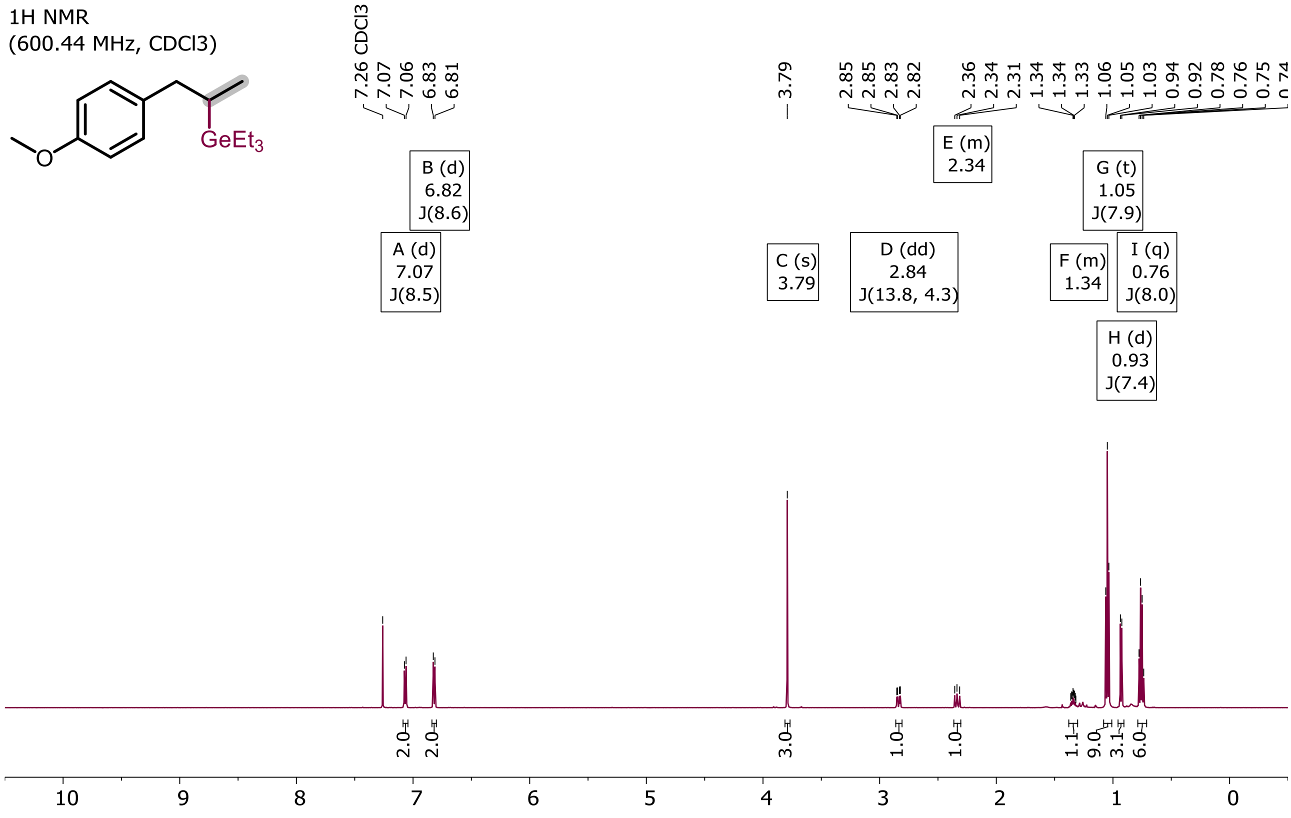

13C NMR
(151.00 MHz, CDCl3)

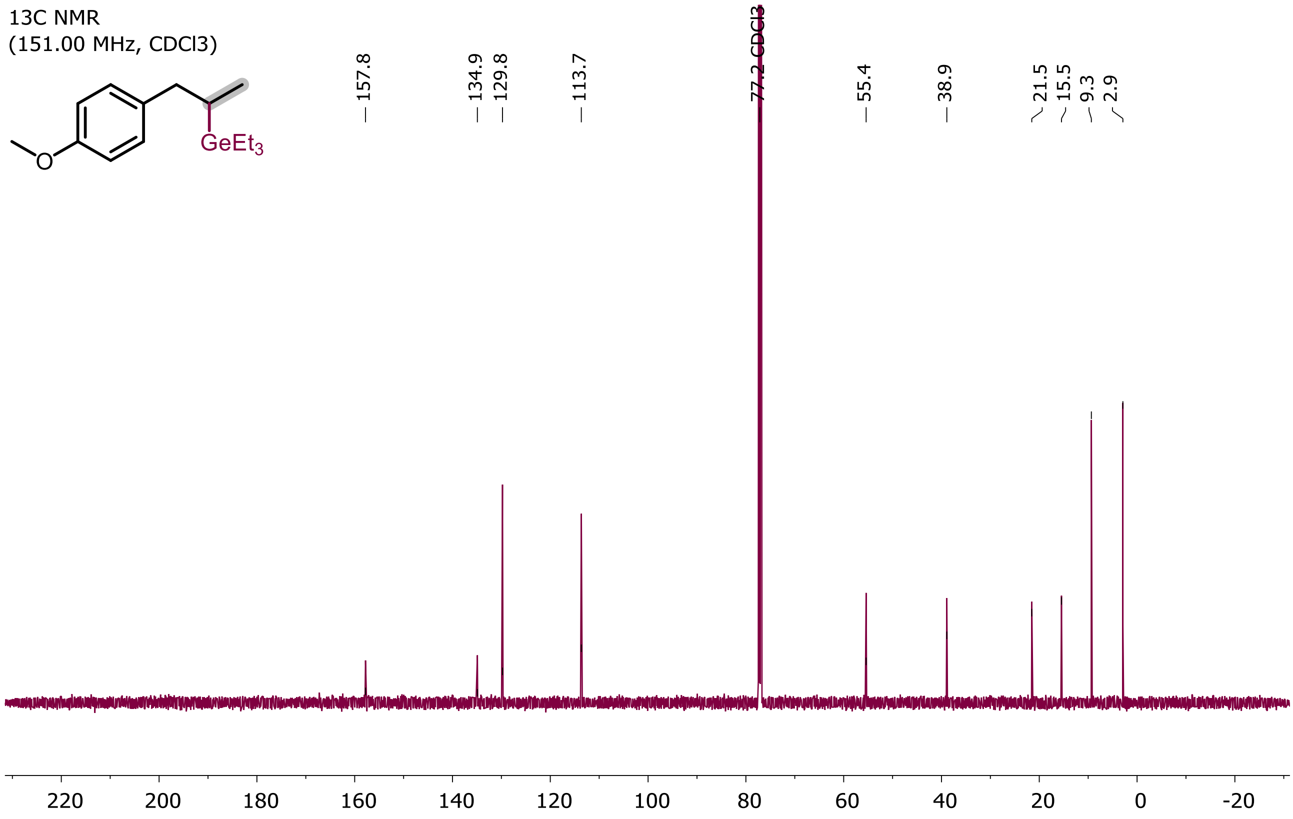

(1-(3,4-Dimethoxyphenyl)propan-2-yl)triethylgermane (4)

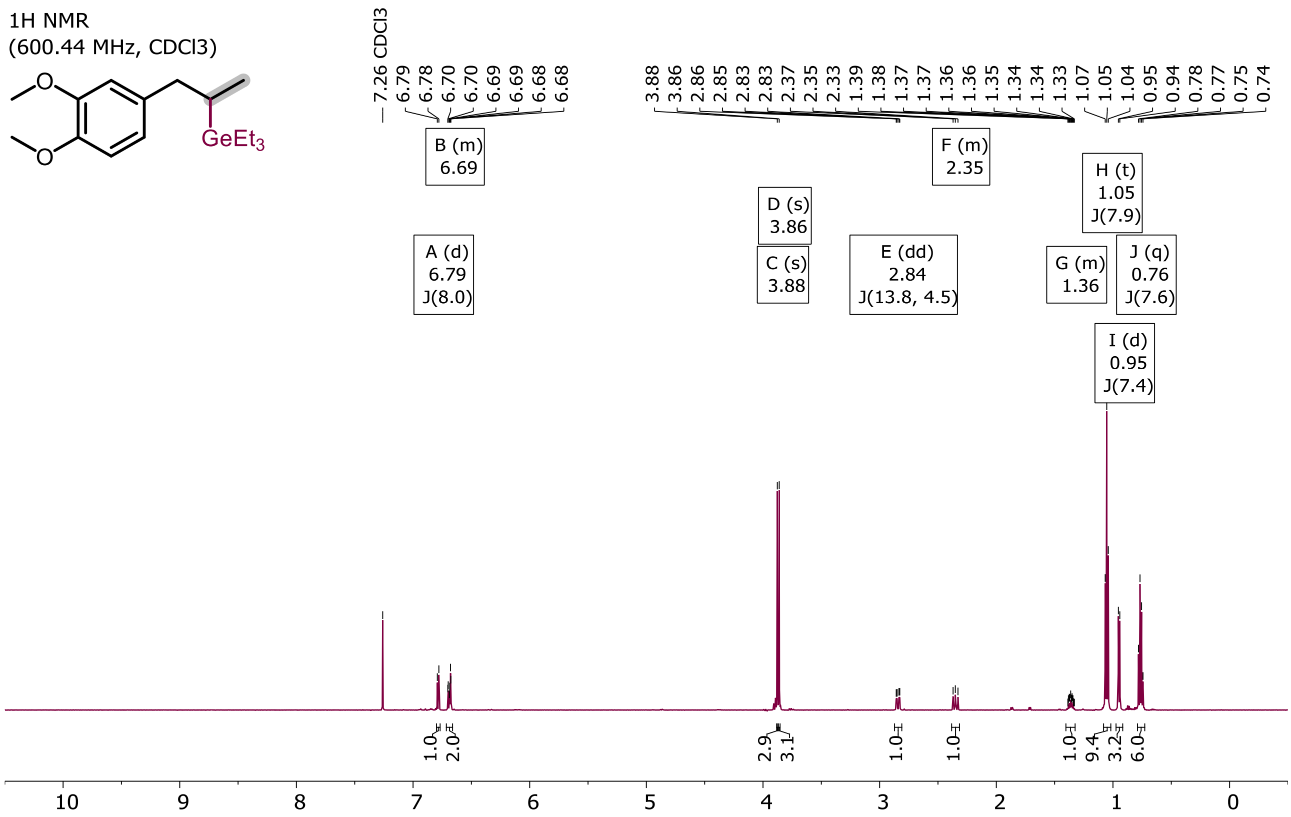

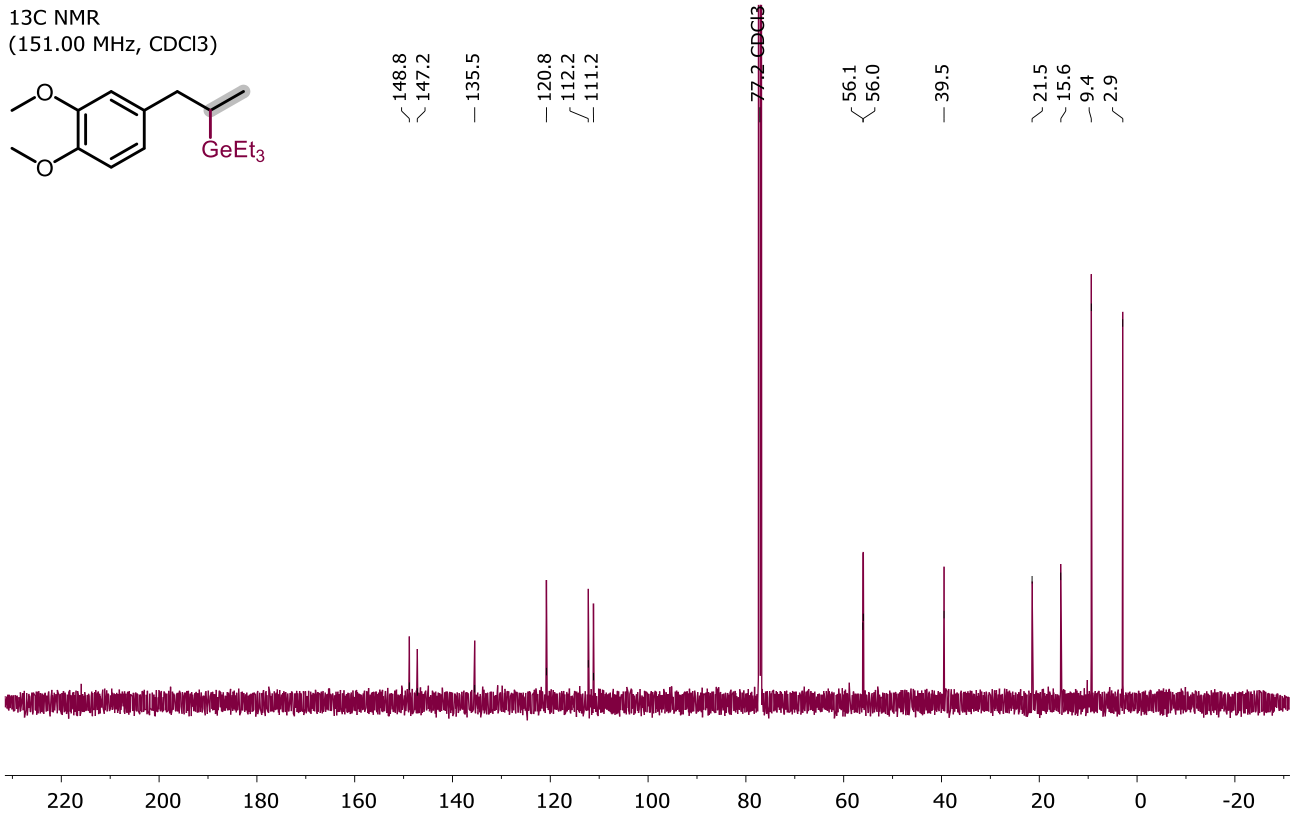

(1-(Benzo[*d*][1,3]dioxol-5-yl)propan-2-yl)triethylgermane (5)

1H NMR
(600.44 MHz, CDCl3)

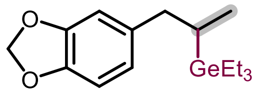

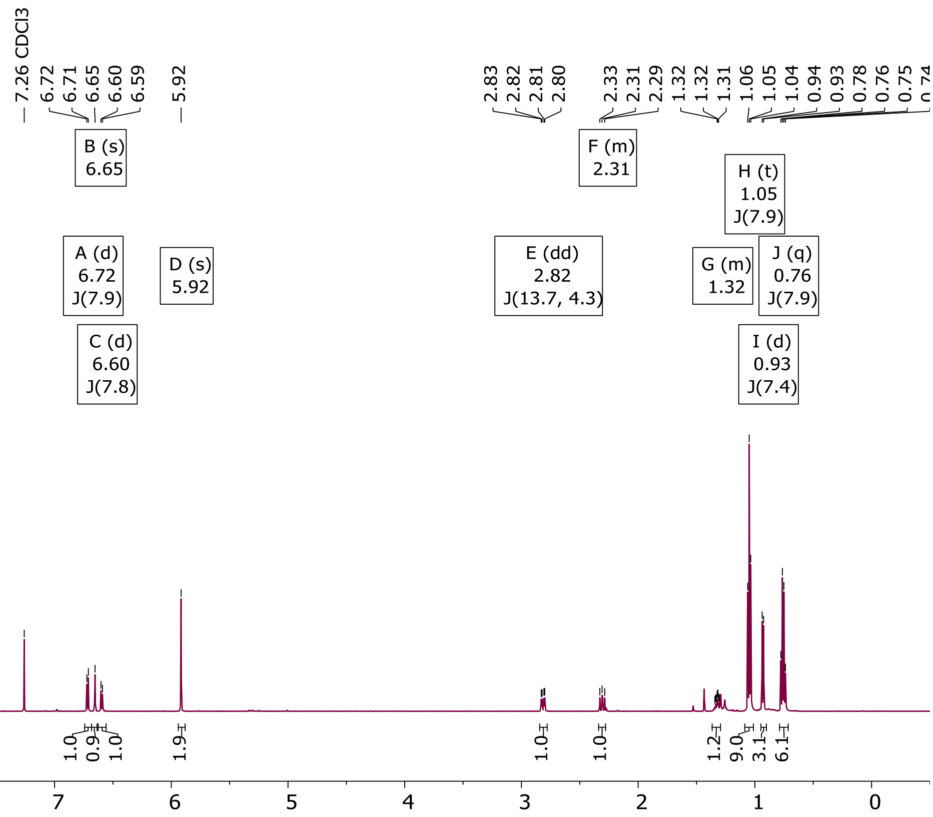

13C NMR
(151.00 MHz, CDCl3)

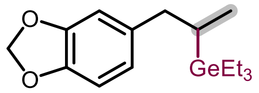

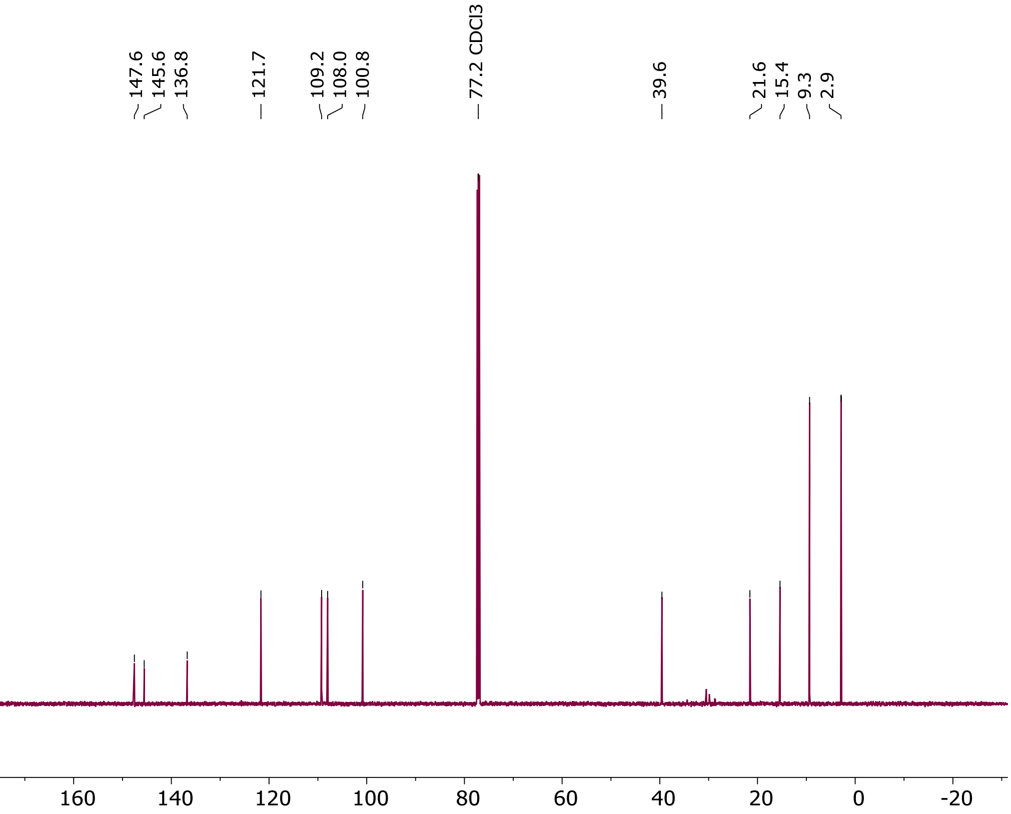

Triethyl(1-phenylpropan-2-yl)germane (6)

1H NMR
(600.44 MHz, CDCl3)

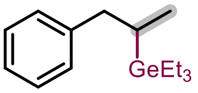

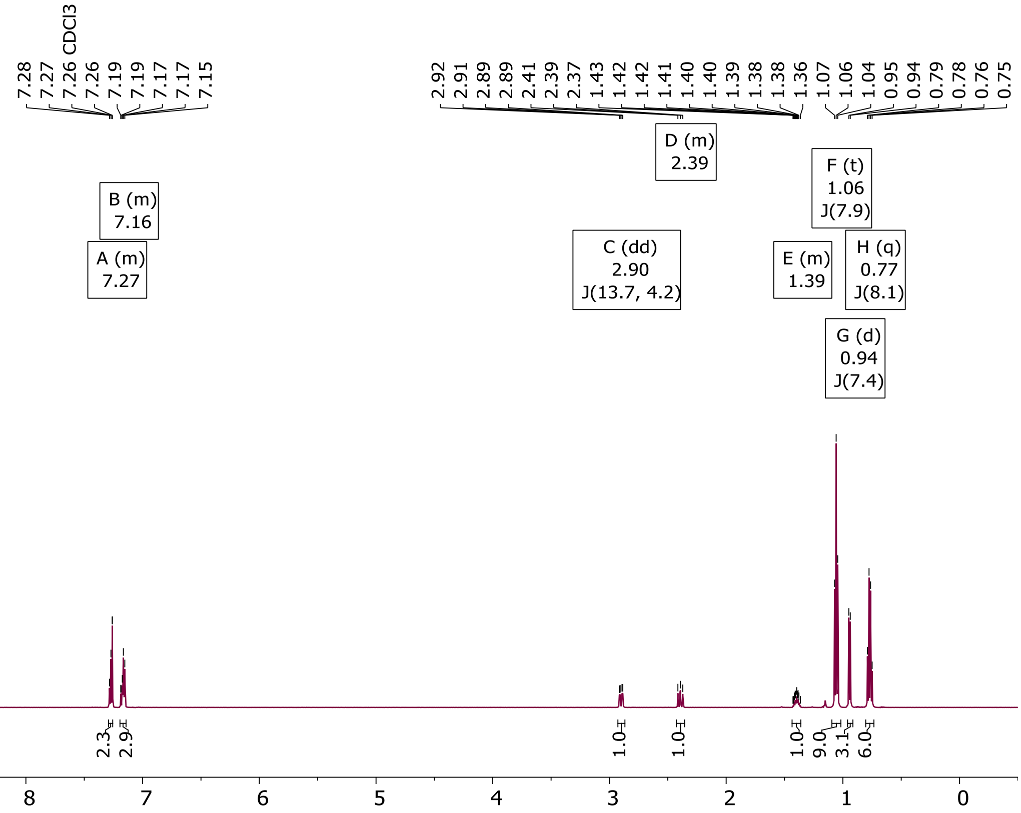

13C NMR
(151.00 MHz, CDCl3)

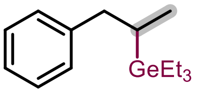

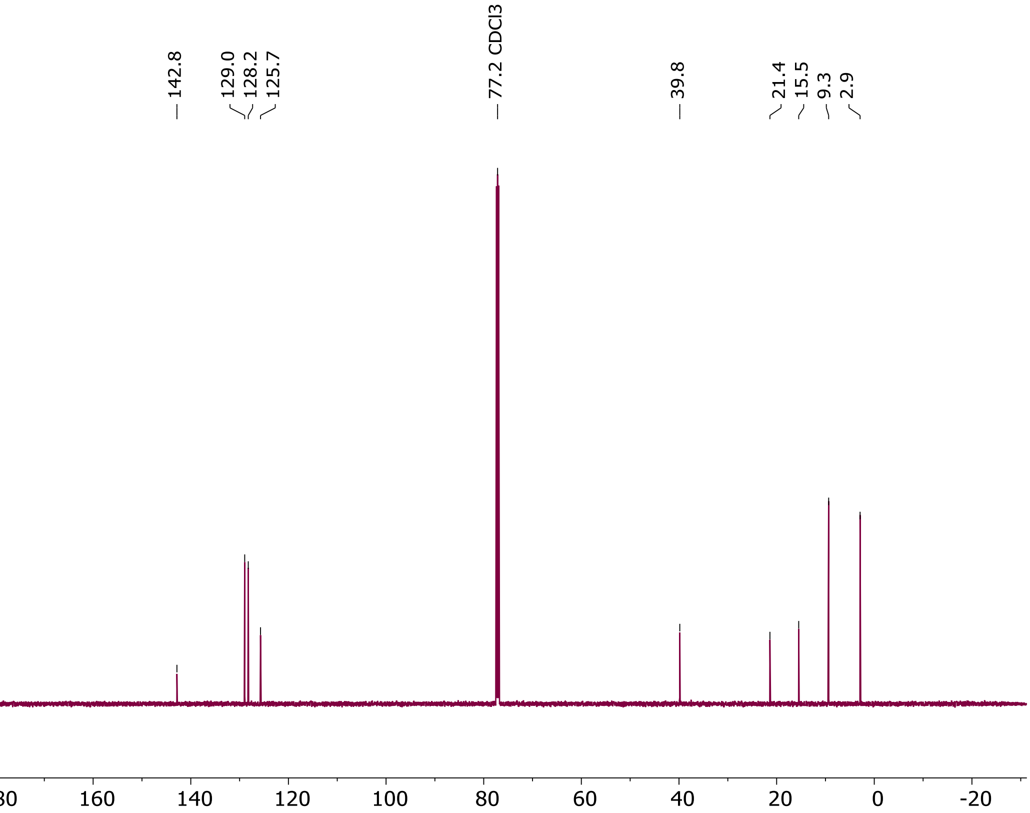

Triethyl(1-(naphthalen-2-yl)propan-2-yl)germane (7)

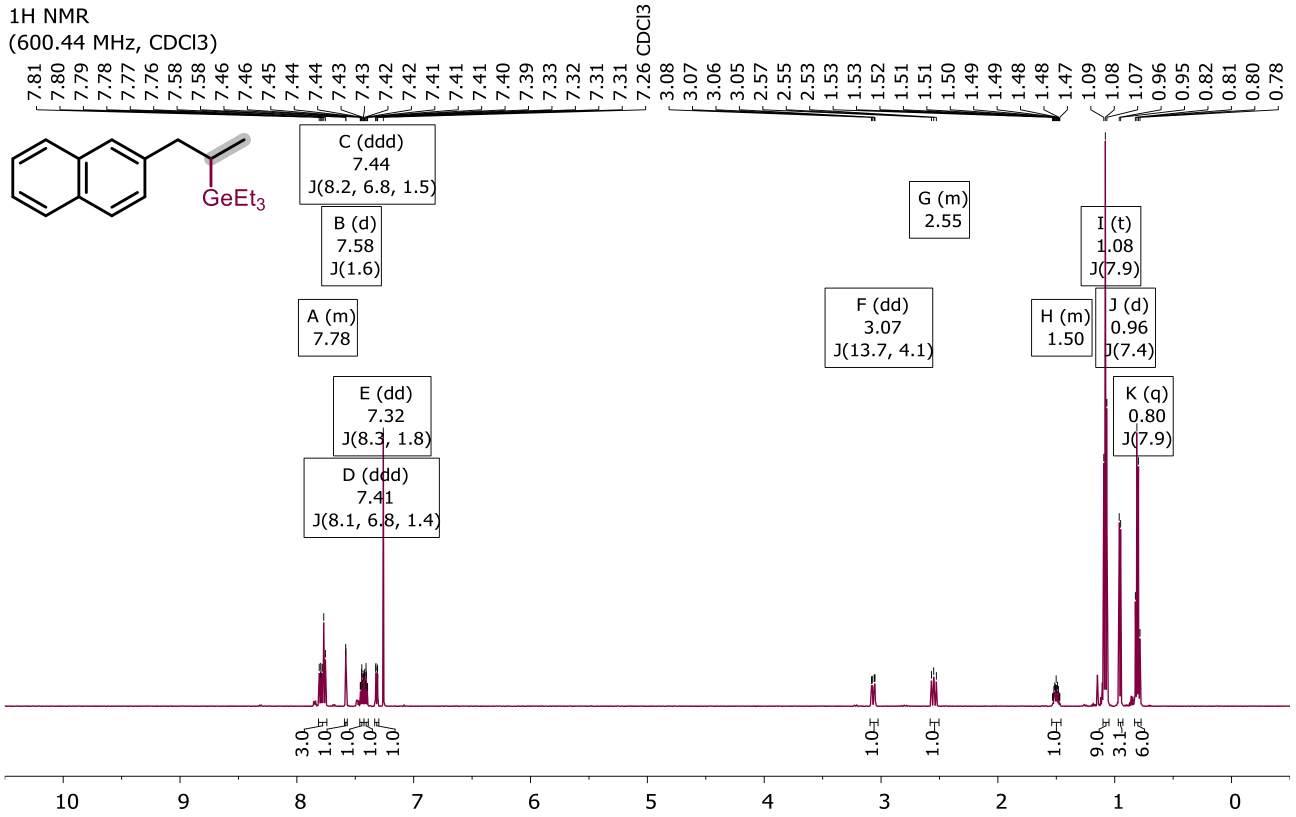

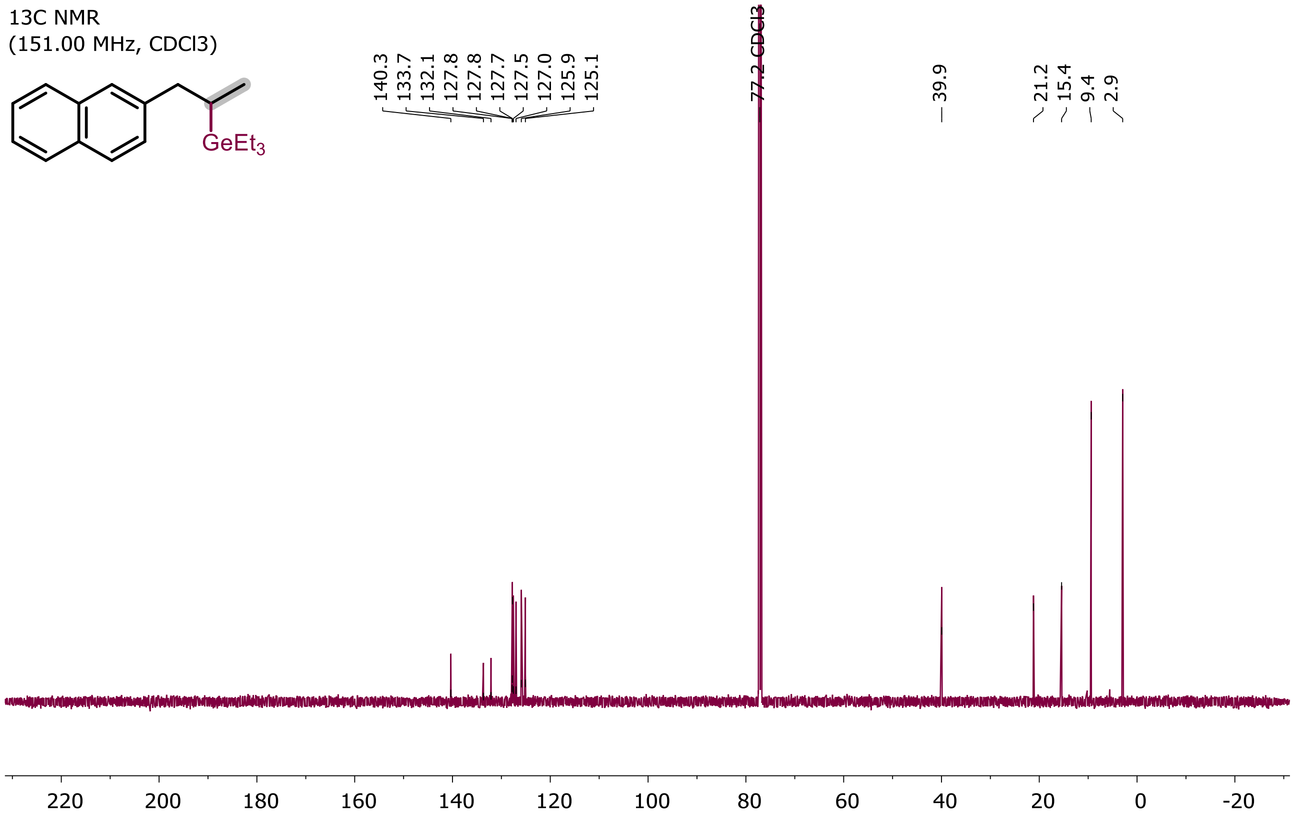

Triethyl(1,2,3,4-tetrahydronaphthalen-2-yl)germane (8)

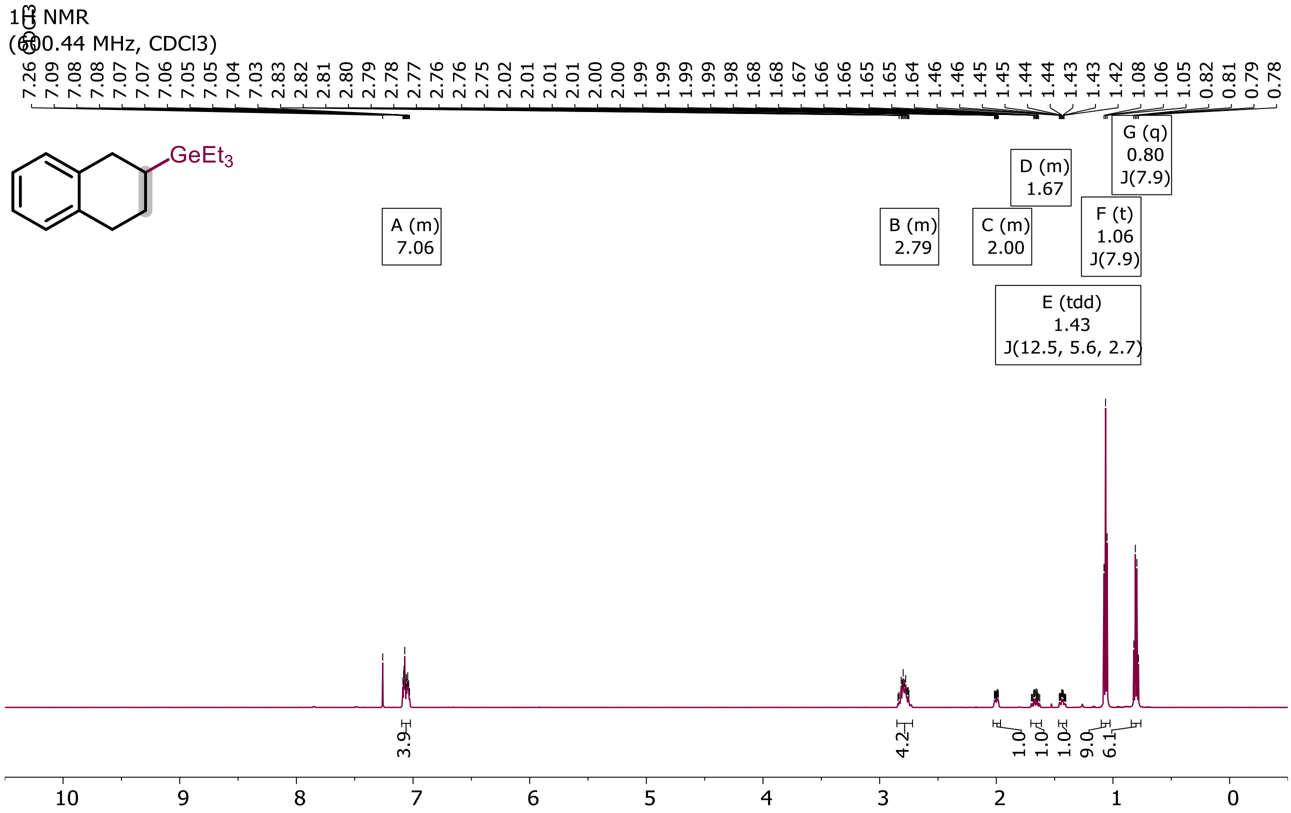

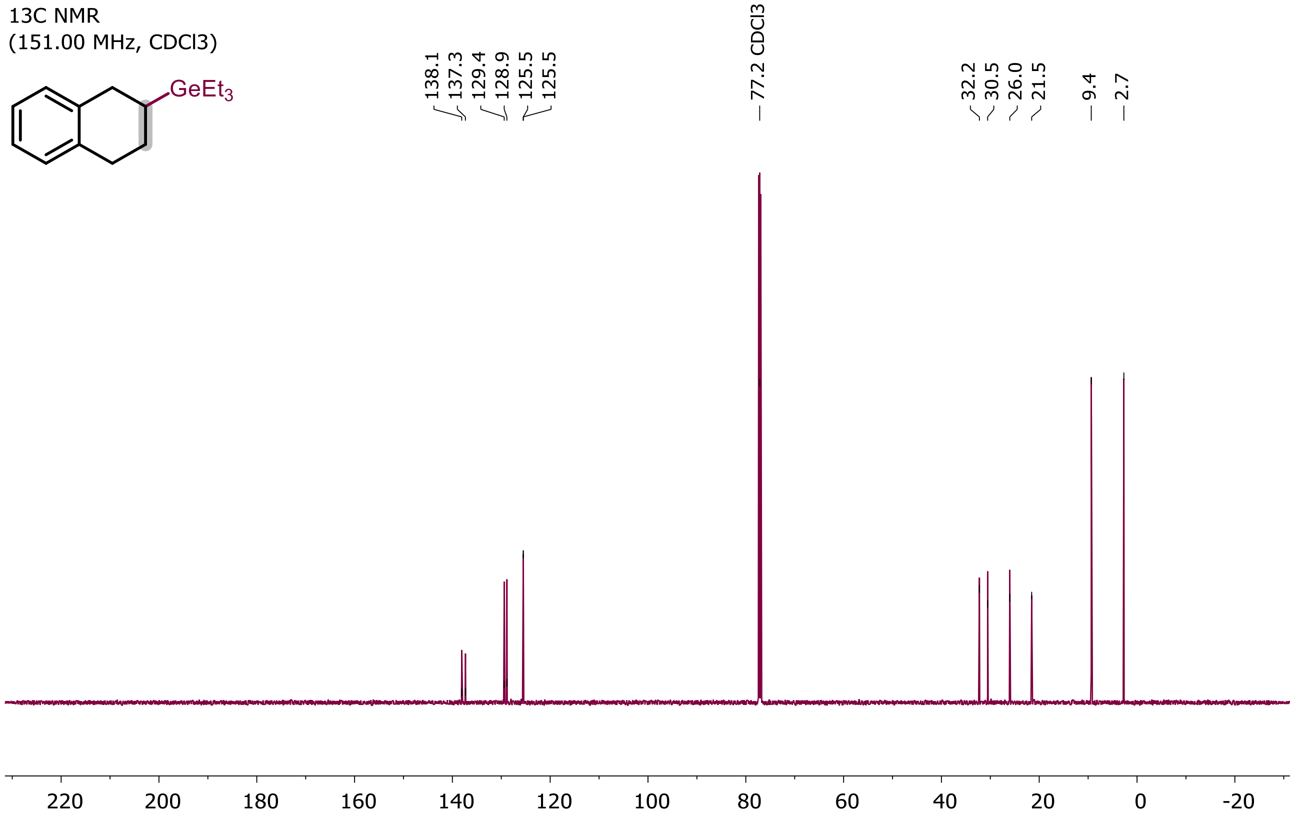

Triethyl(1-(2-tolyl)propan-2-yl)germane (9)

1H NMR
(600.44 MHz, CDCl3)

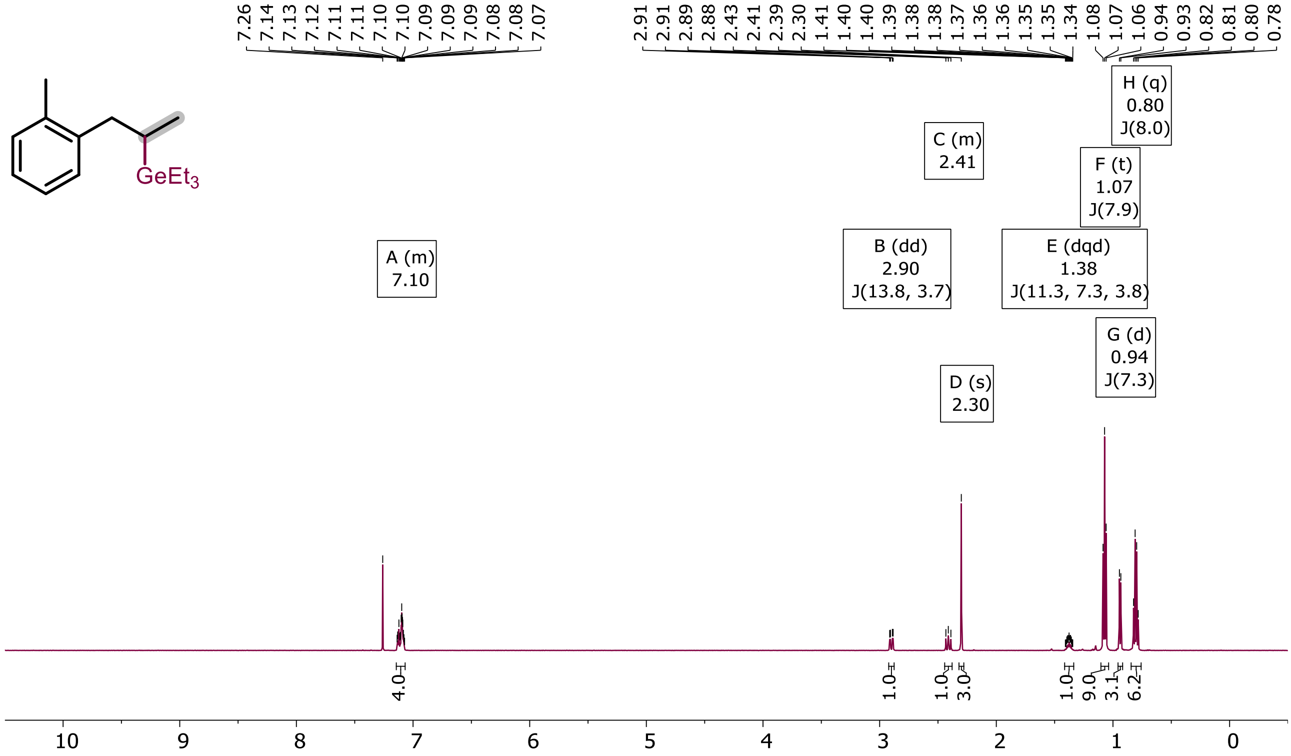

13C NMR
(151.00 MHz, CDCl3)

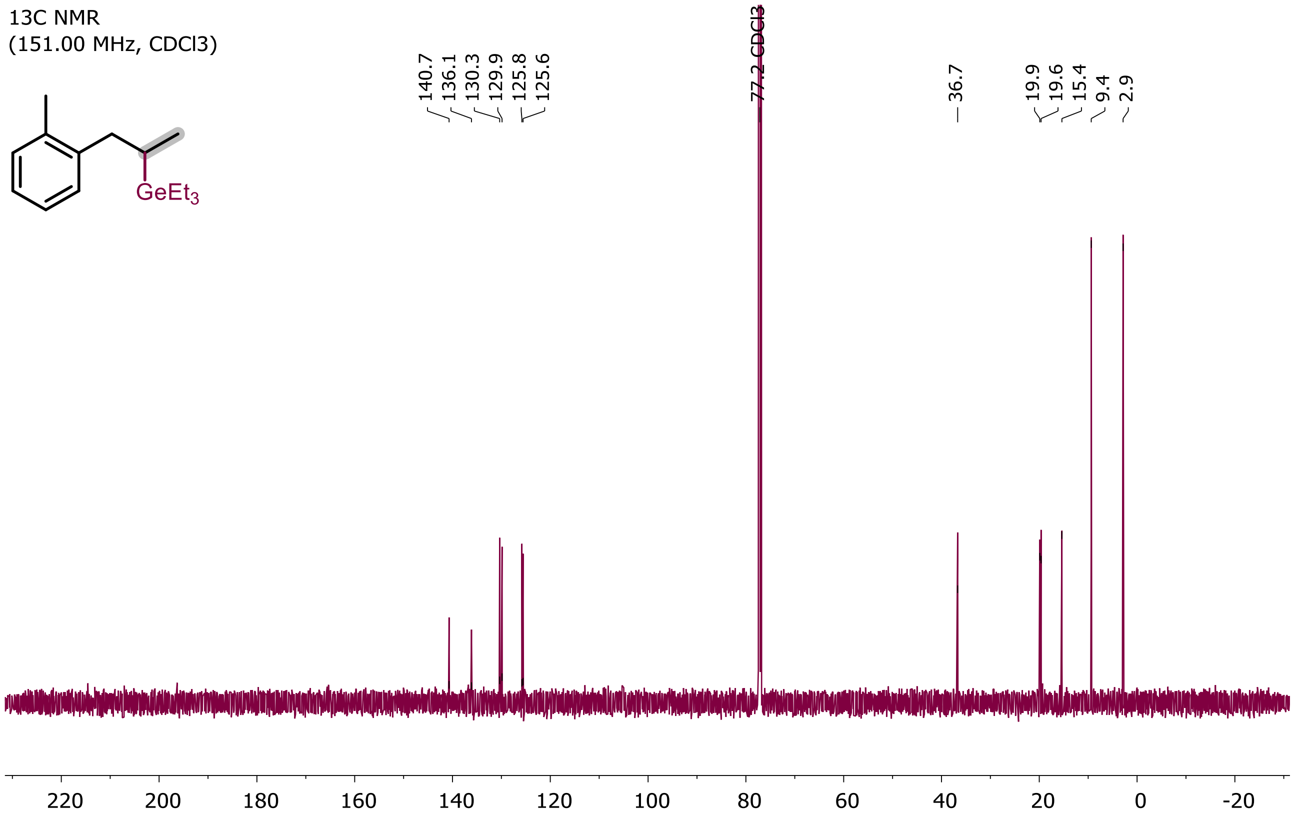

Triethyl(1-(3-tolyl)propan-2-yl)germane (10)

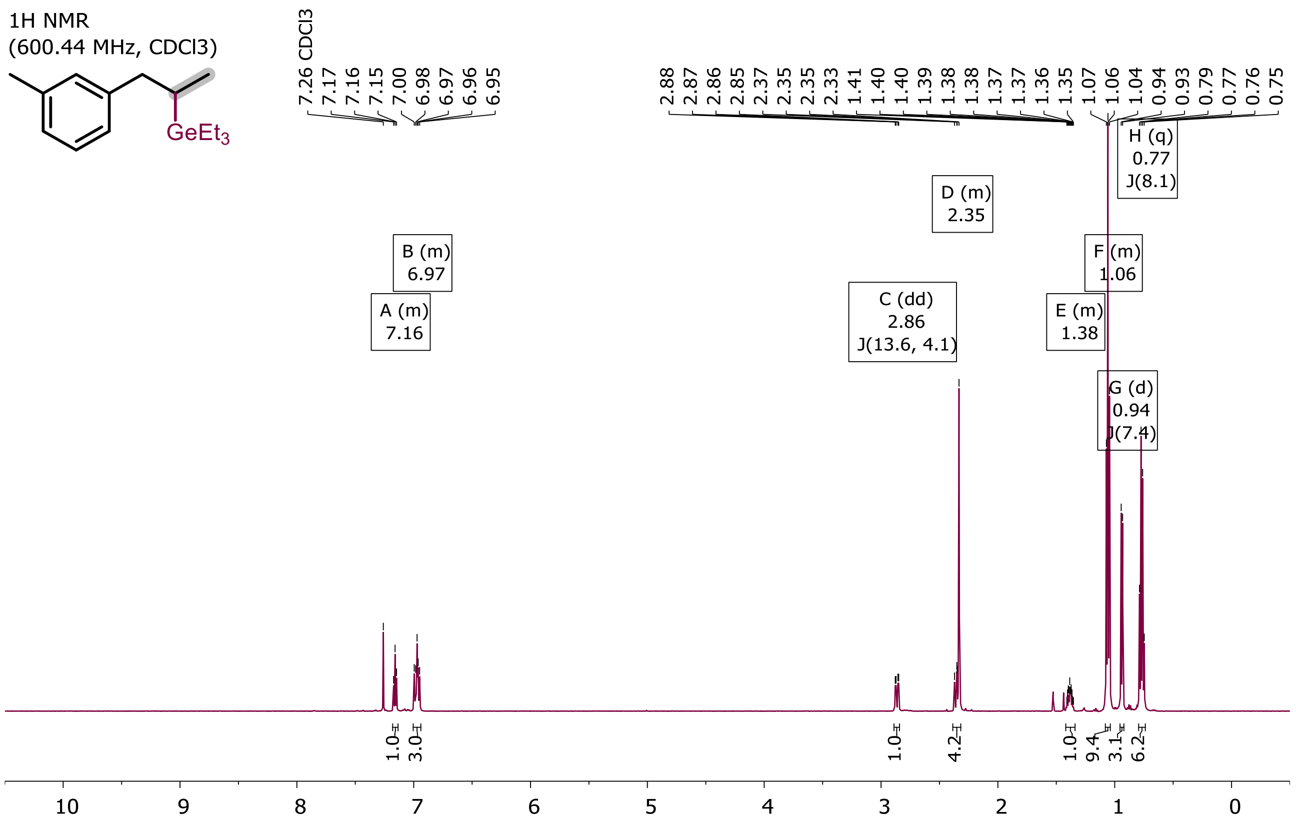

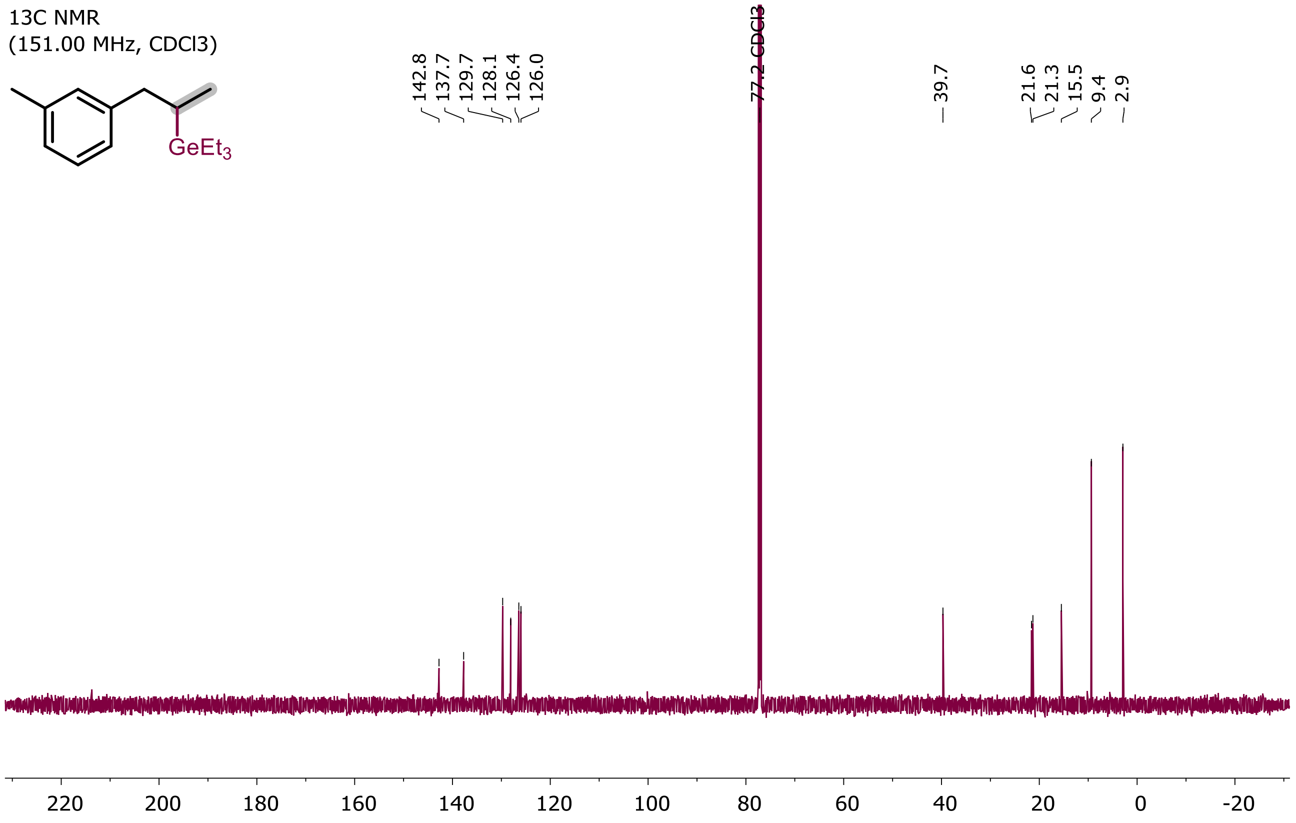

(1-(3-Chlorophenyl)propan-2-yl)triethylgermane (11)

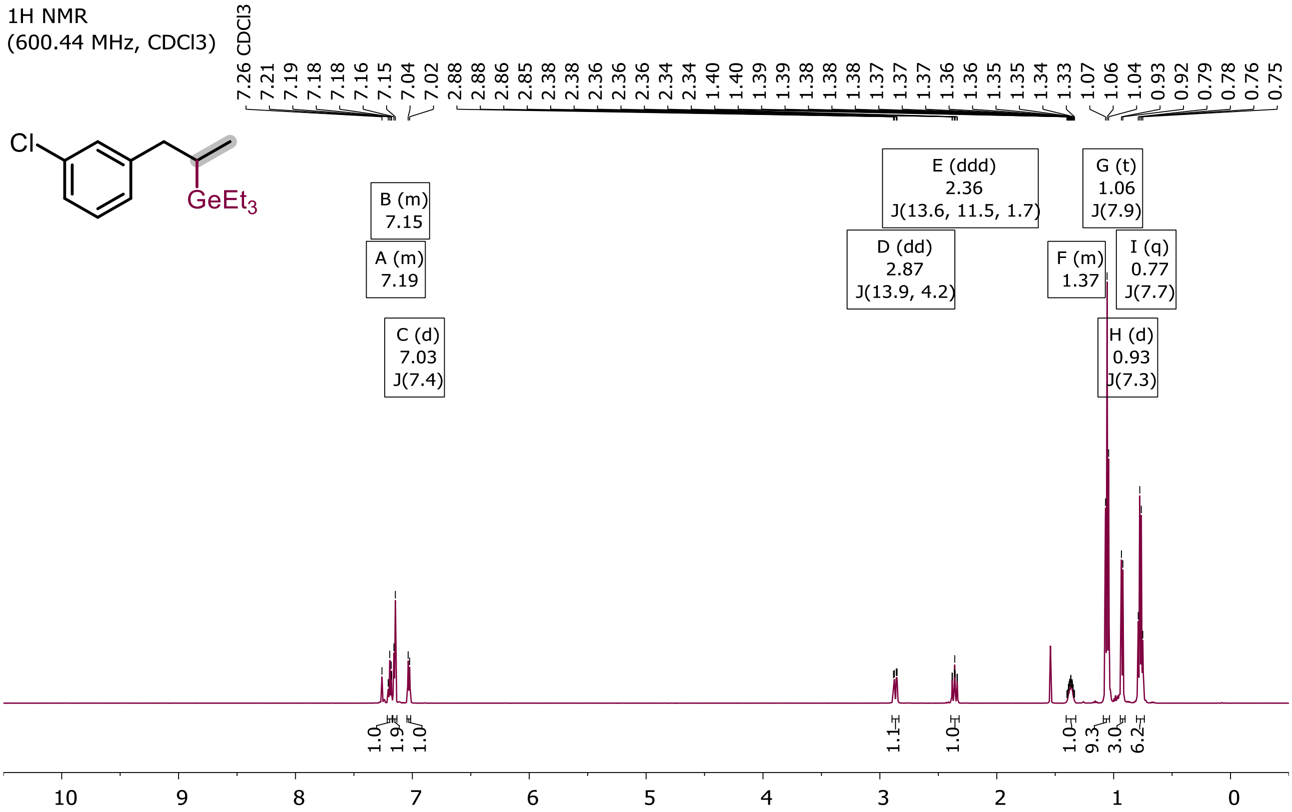

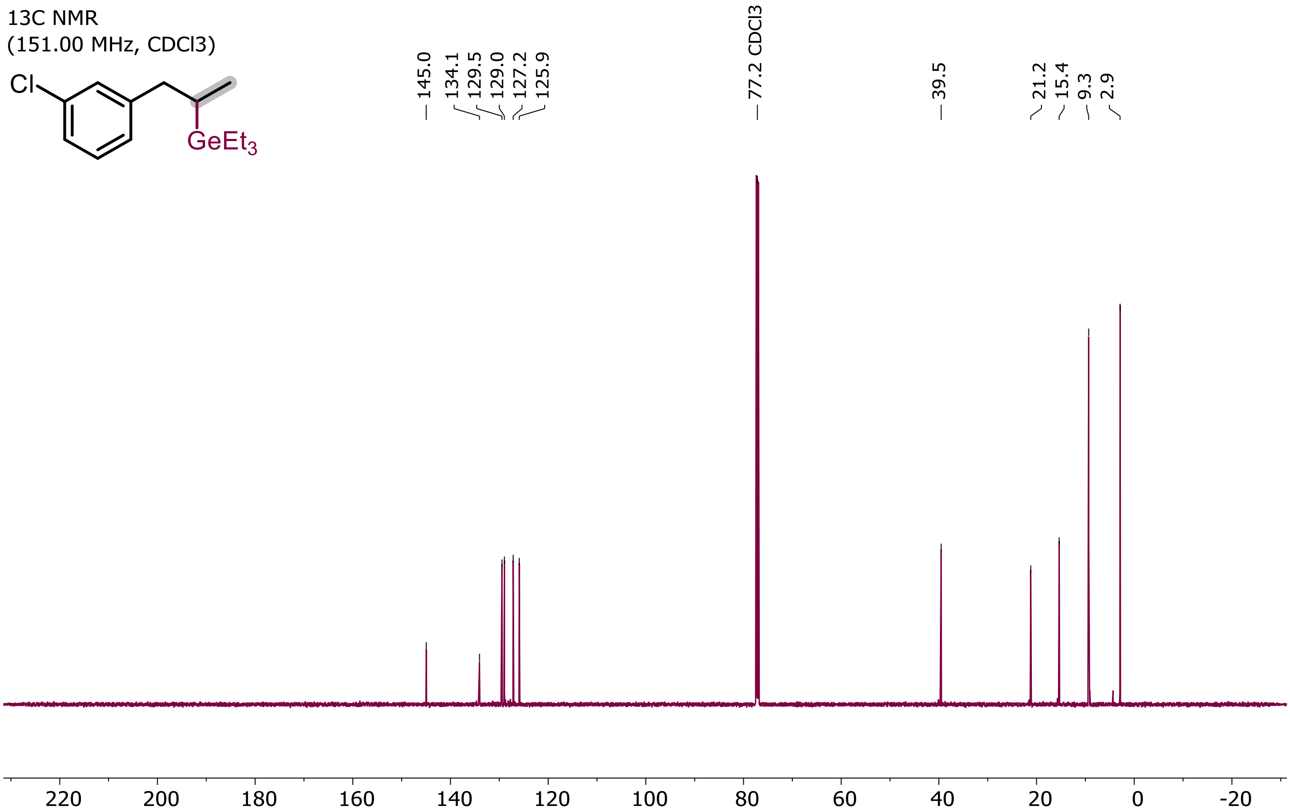

Triethyl(1-(4-fluorophenyl)propan-2-yl)germane (12)

1H NMR
(600.44 MHz, CDCl3)

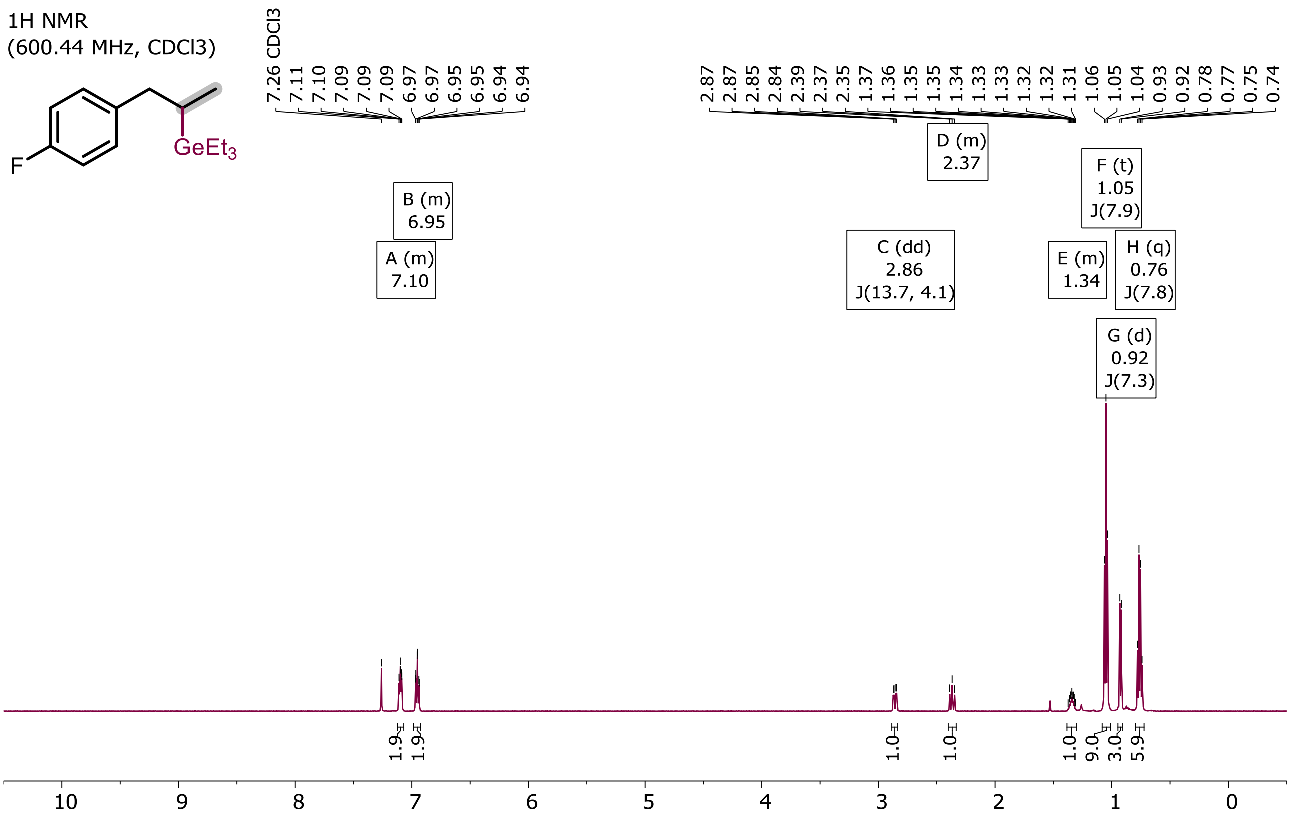

13C NMR
(151.00 MHz, CDCl3)

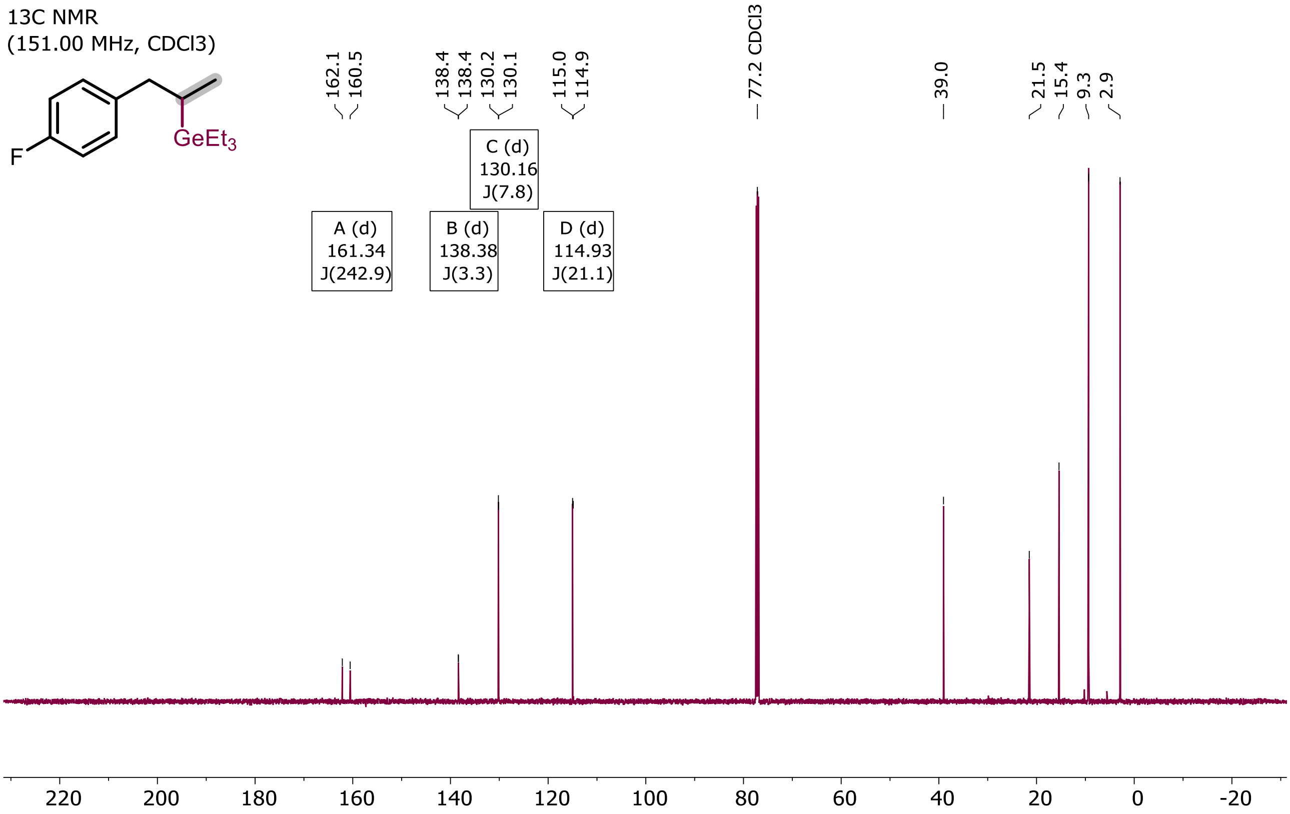

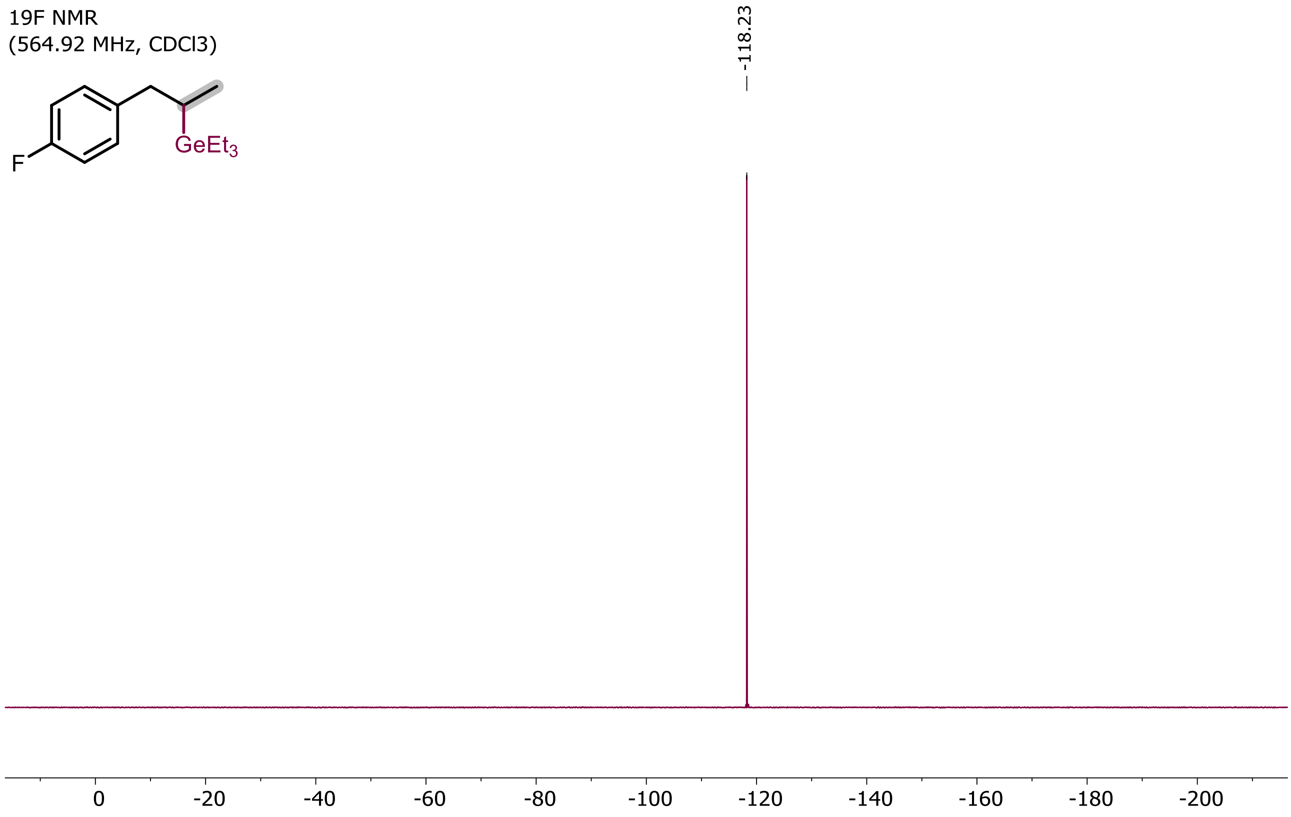

Triethyl(1-(3-(trifluoromethyl)phenyl)propan-2-yl)germane (13)

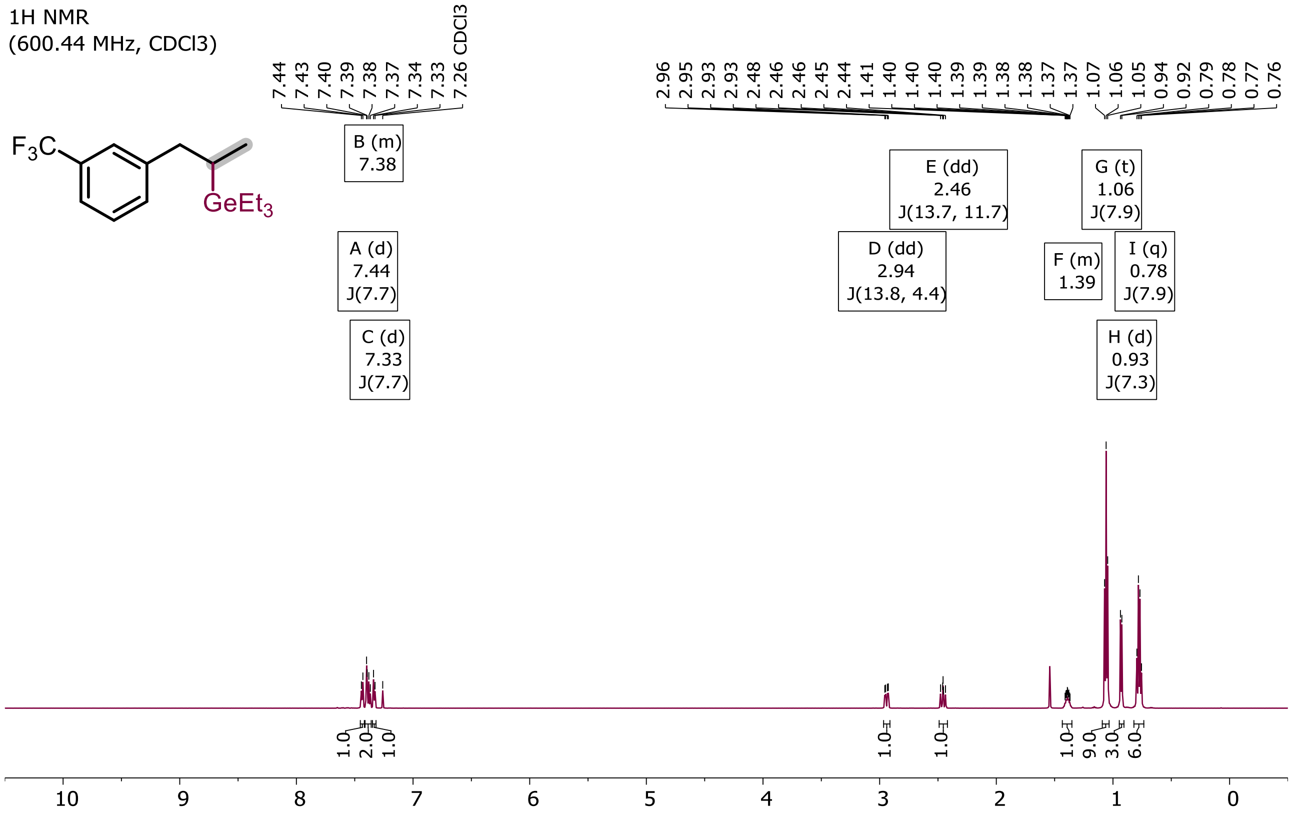

13C NMR
(151.00 MHz, CDCl3)

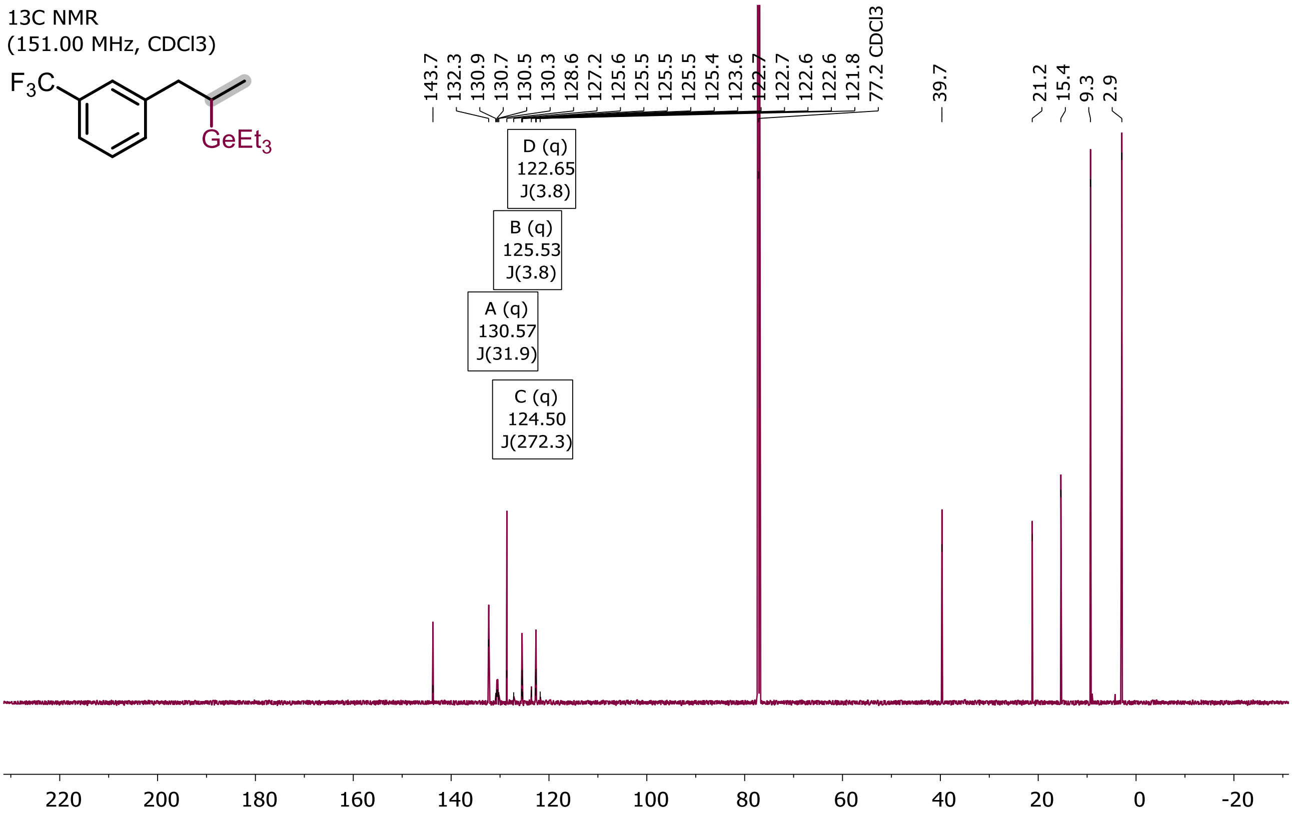

19F NMR
(564.92 MHz, CDCl3)

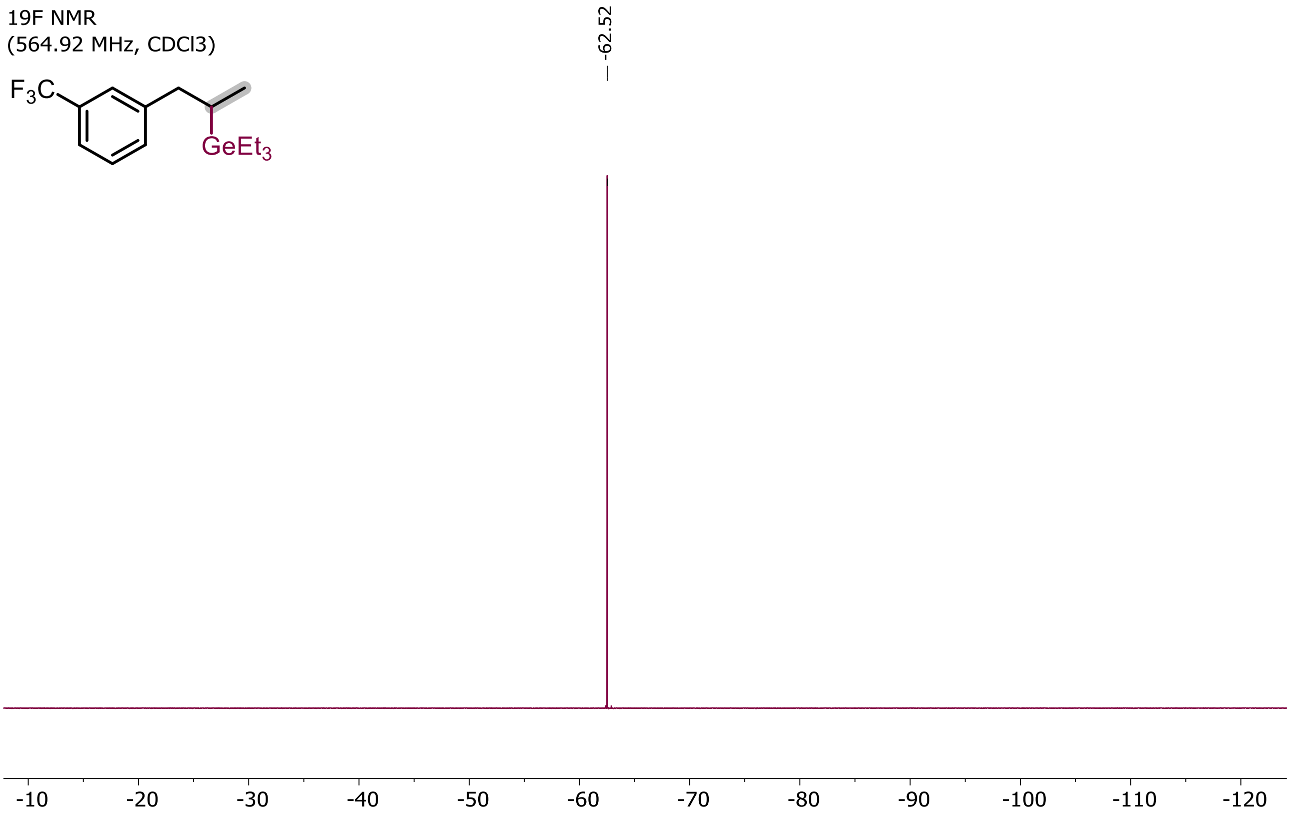

N,N-Diphenyl-4-(2-(triethylgermyl)propyl)aniline (14)

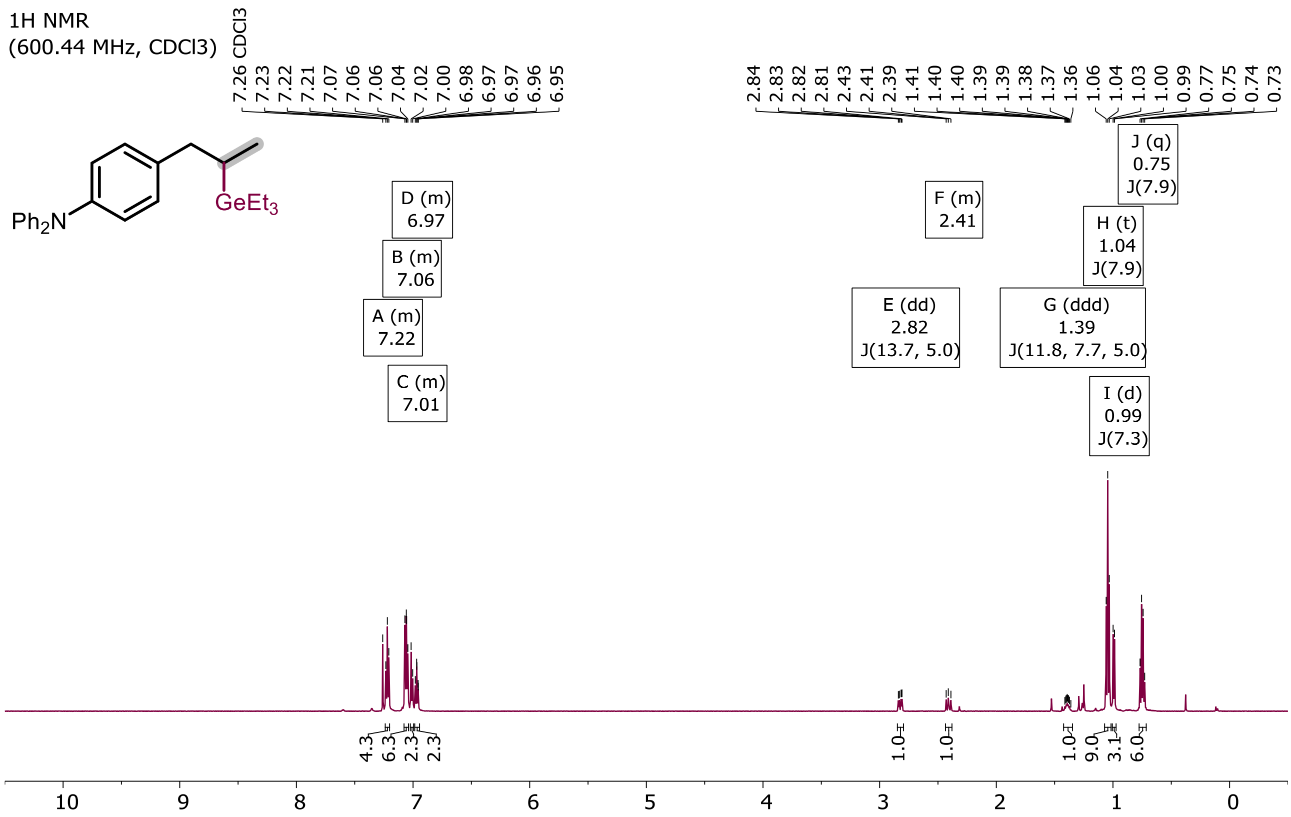

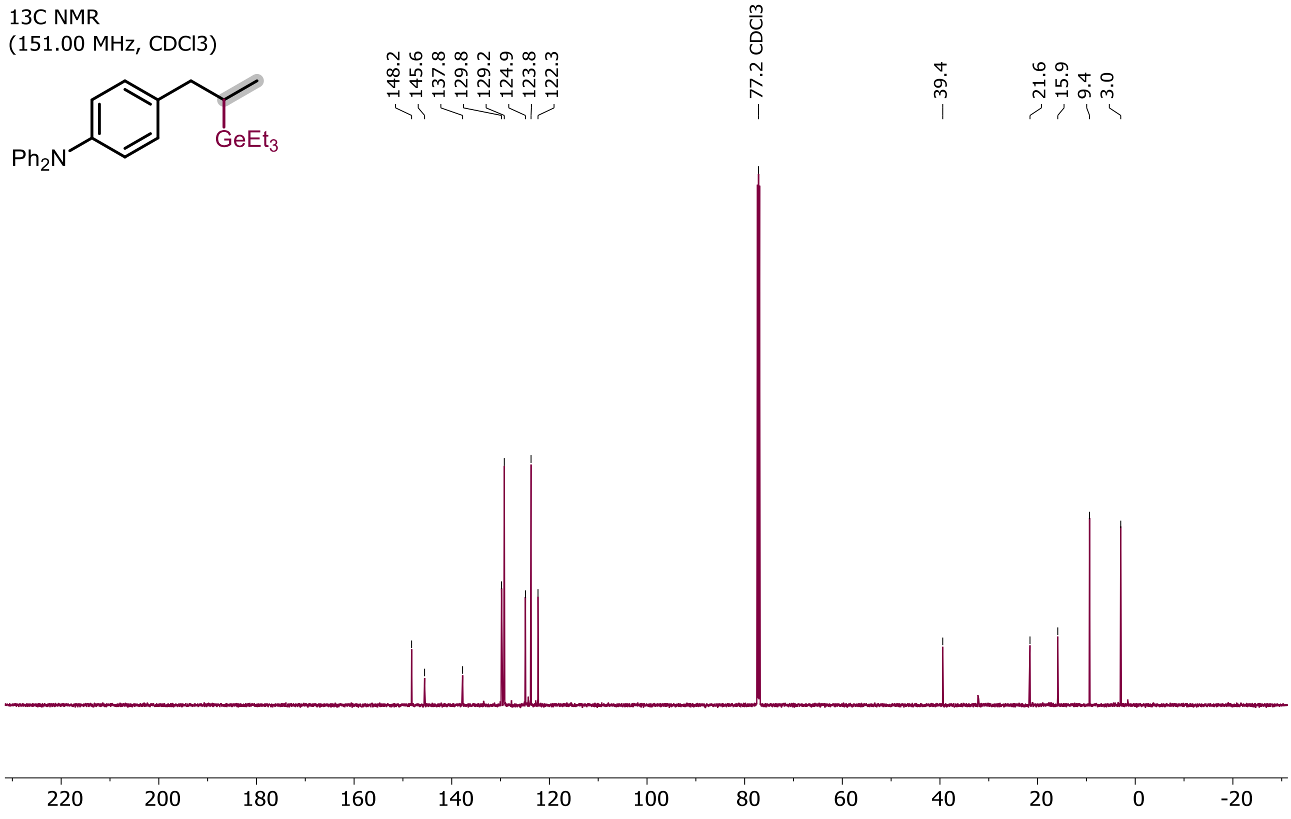

(Z)-triethyl(1-(2-(prop-1-en-1-yloxy)phenyl)propan-2-yl)germane (15)

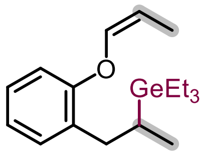

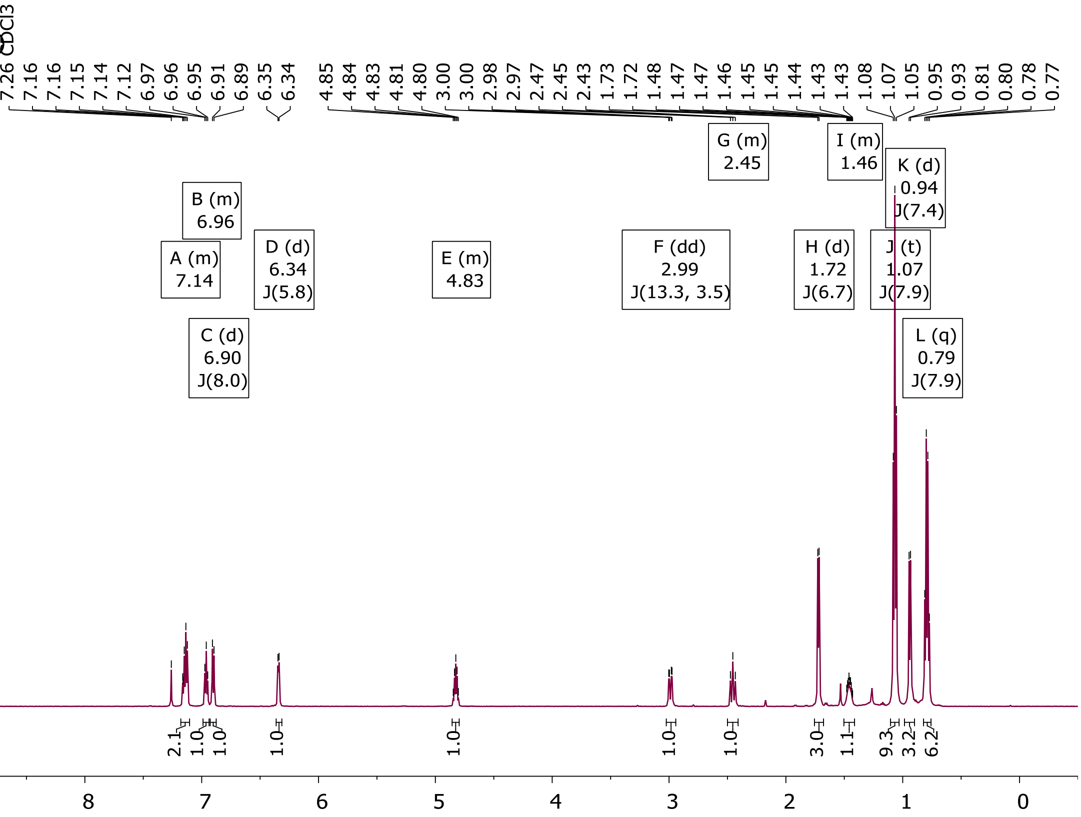

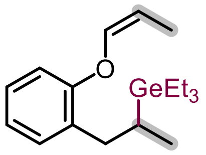

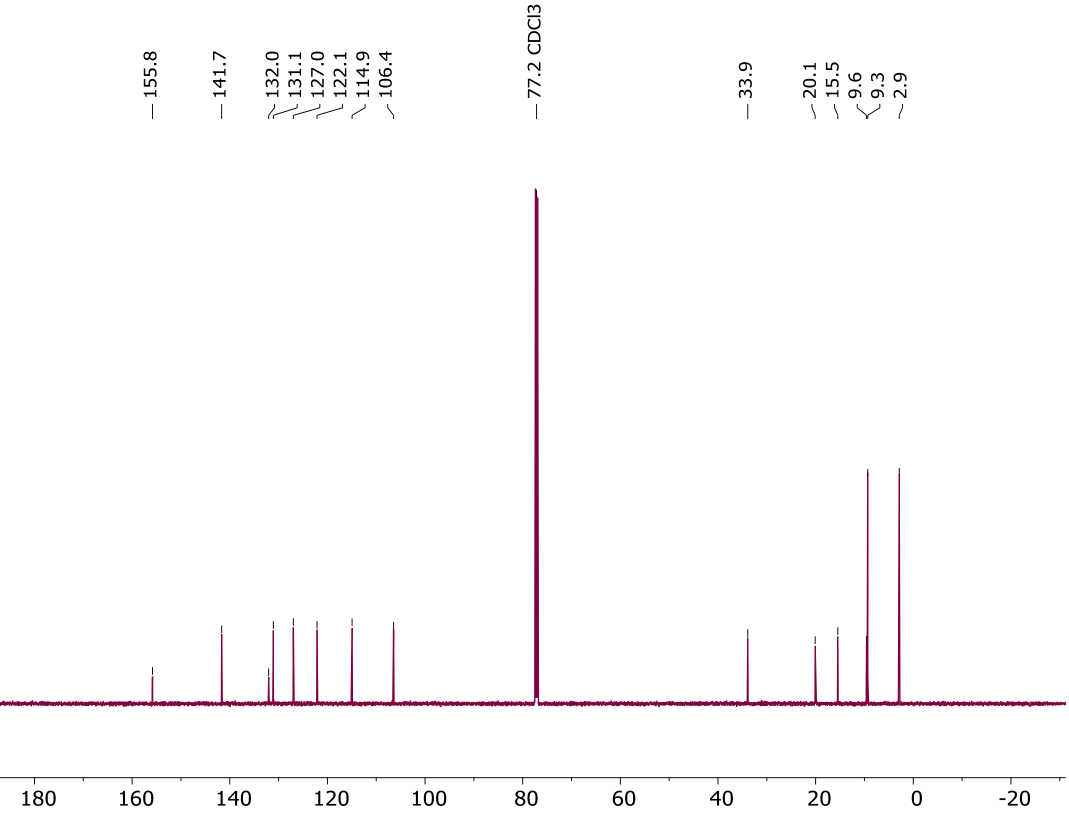

(1-(Benzo[*b*]thiophen-2-yl)propan-2-yl)triethylgermane (16)

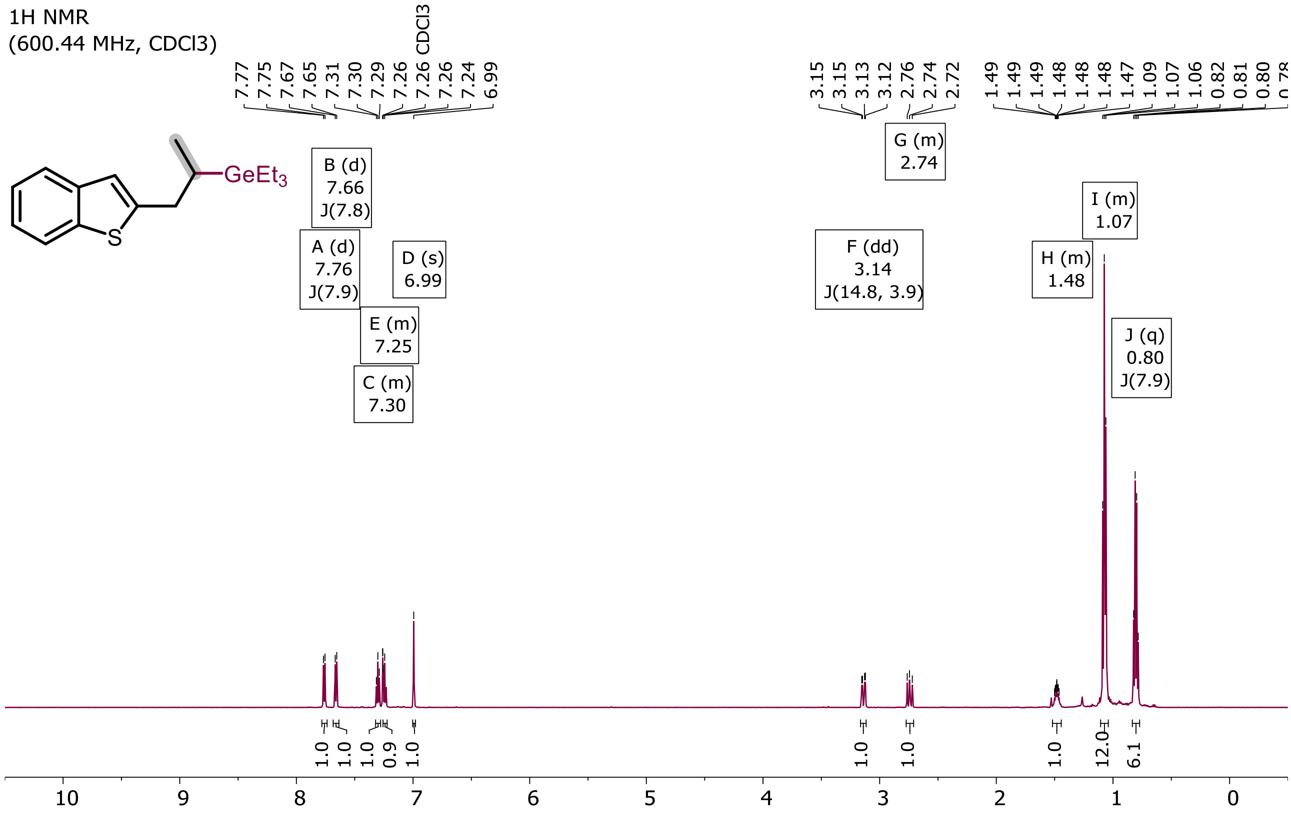

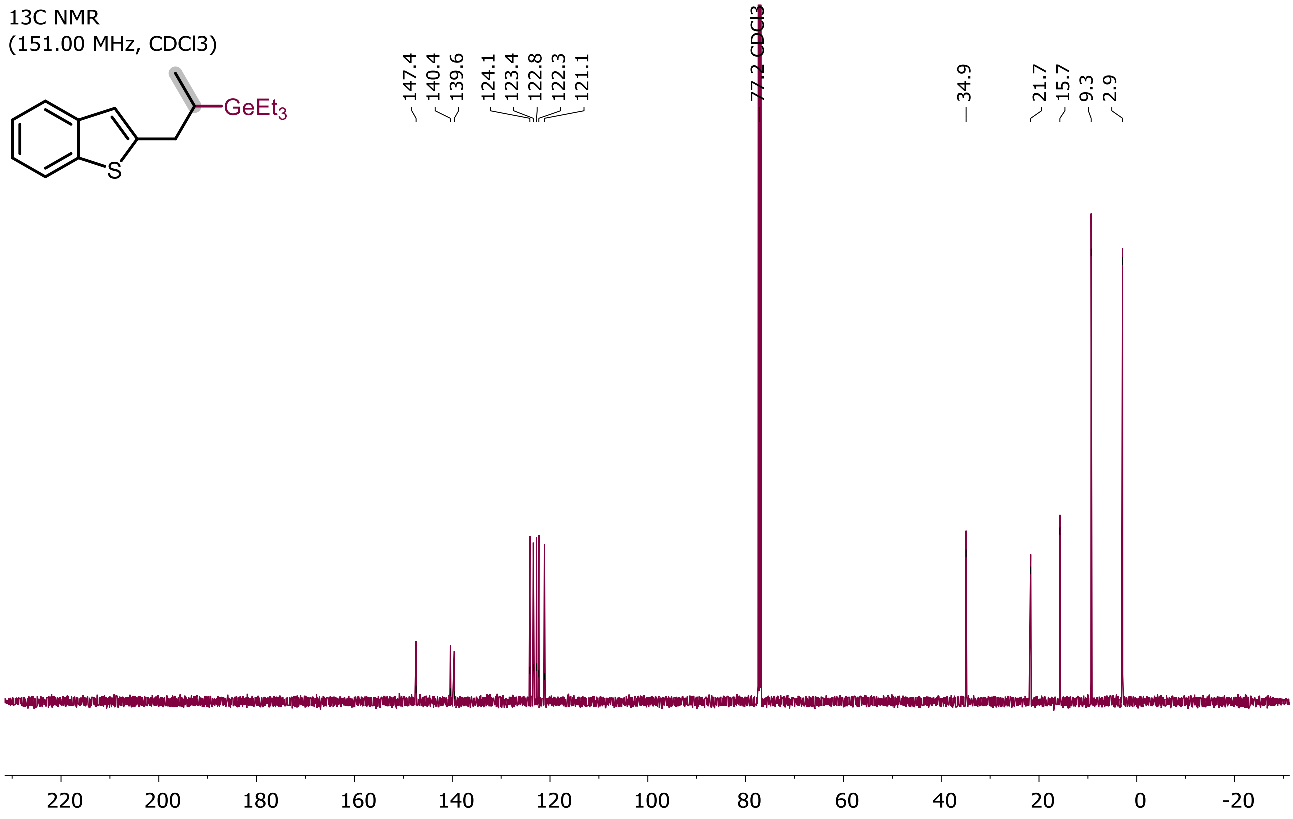

Triethyl(1-(thiophen-2-yl)propan-2-yl)germane (17)

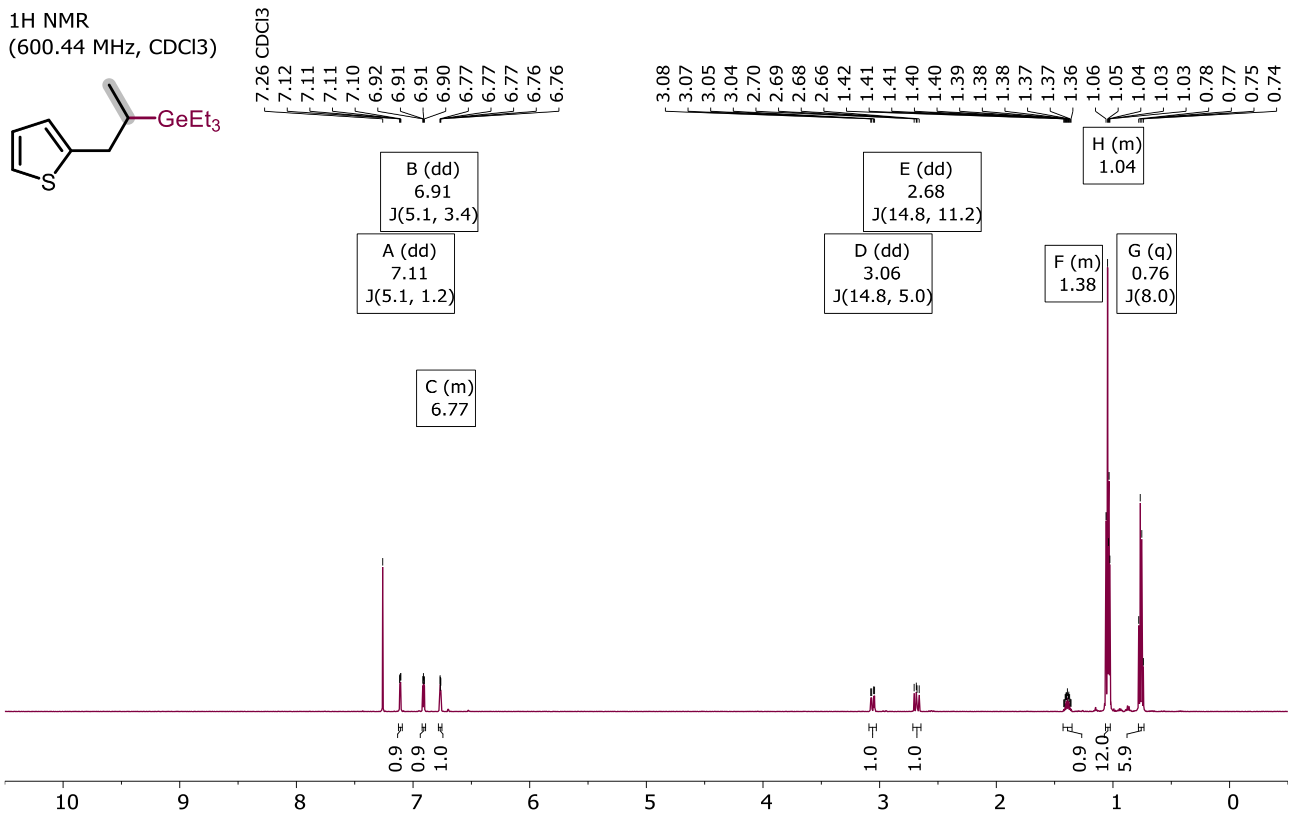

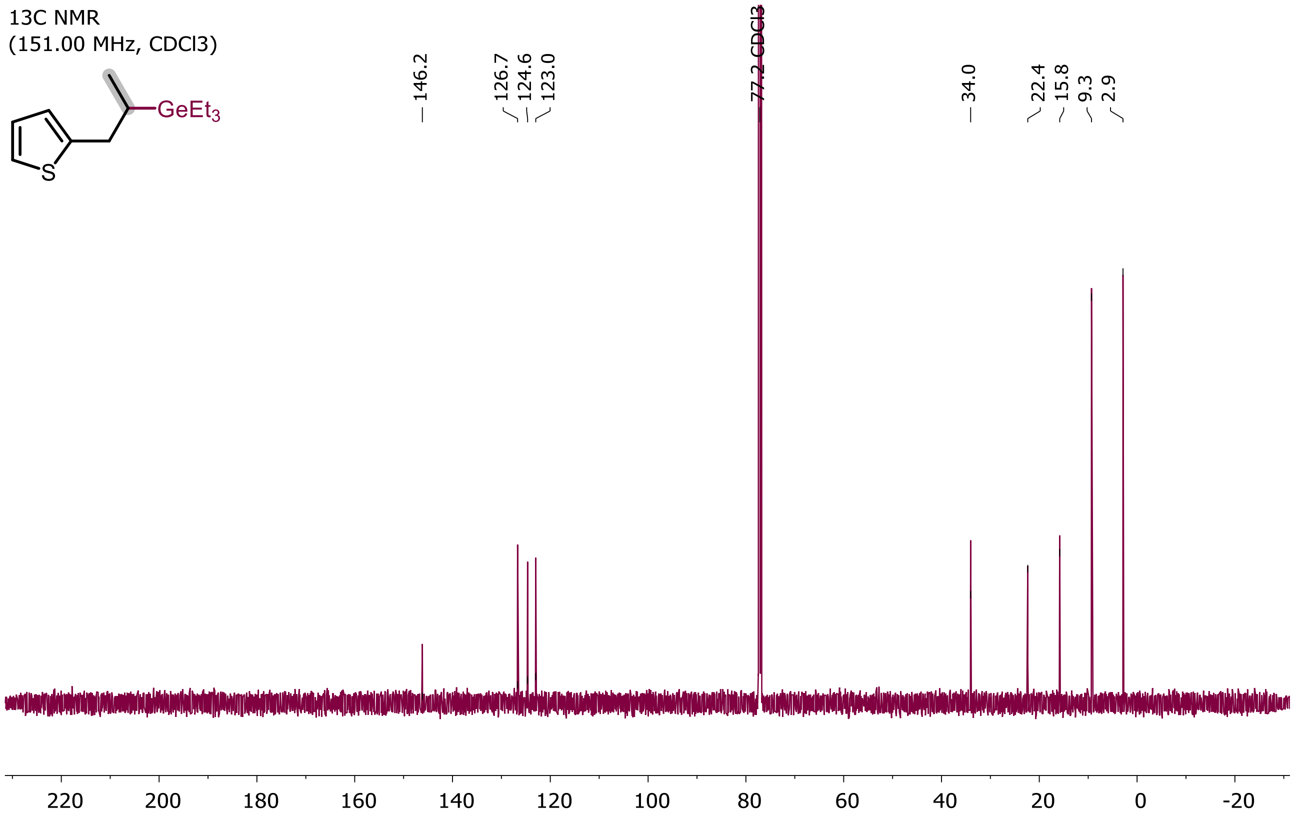

Triphenyl(2-(triethylgermyl)propyl)silane (18)

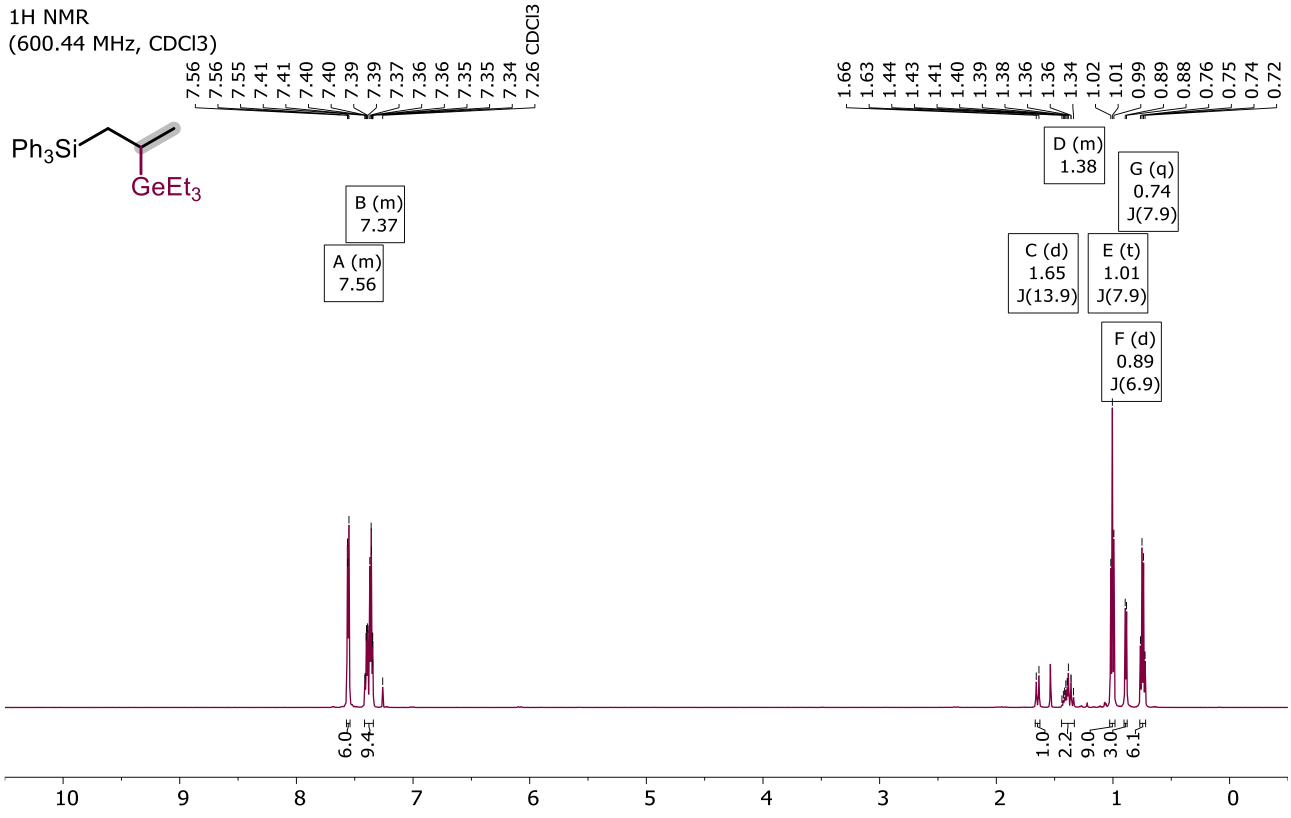

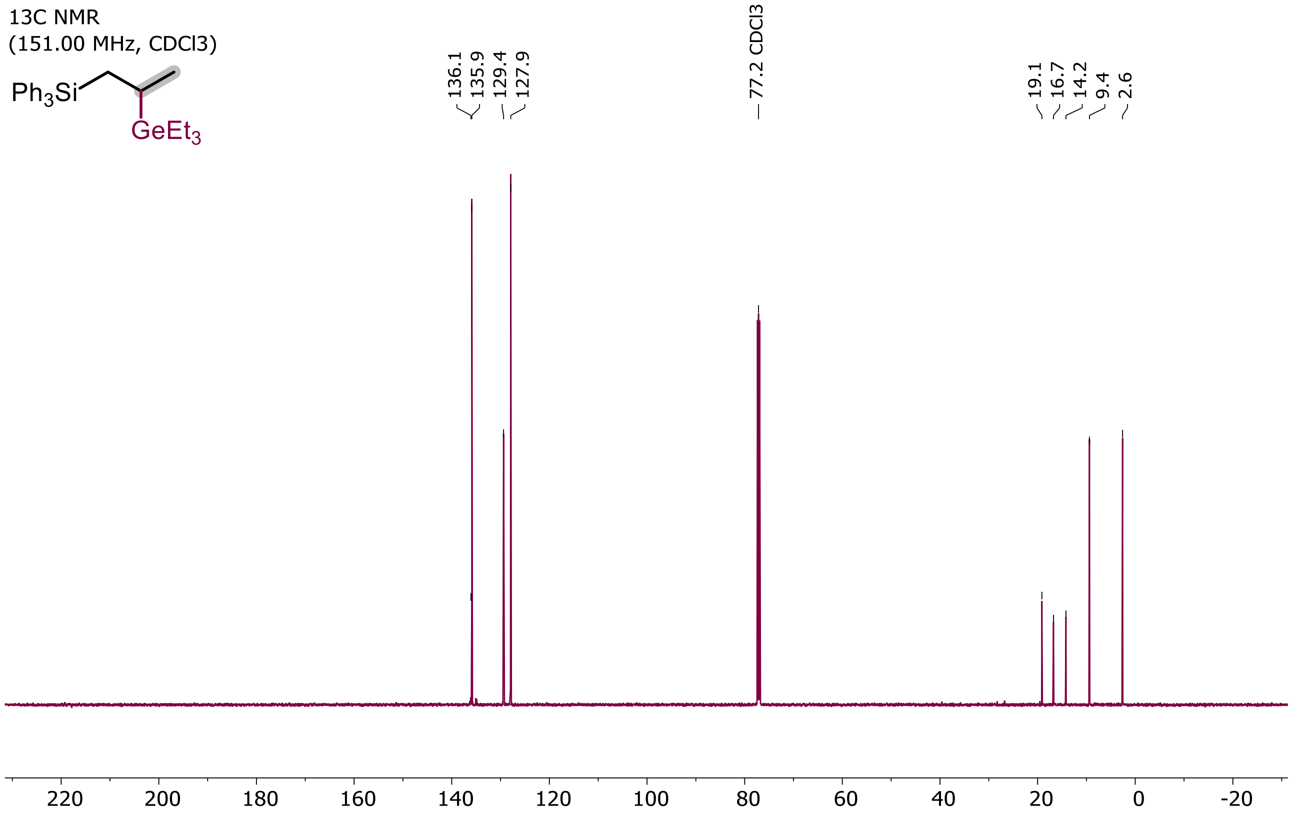

Triethyl(1-(4,4,5,5-tetramethyl-1,3,2-dioxaborolan-2-yl)propan-2-yl)germane (19)

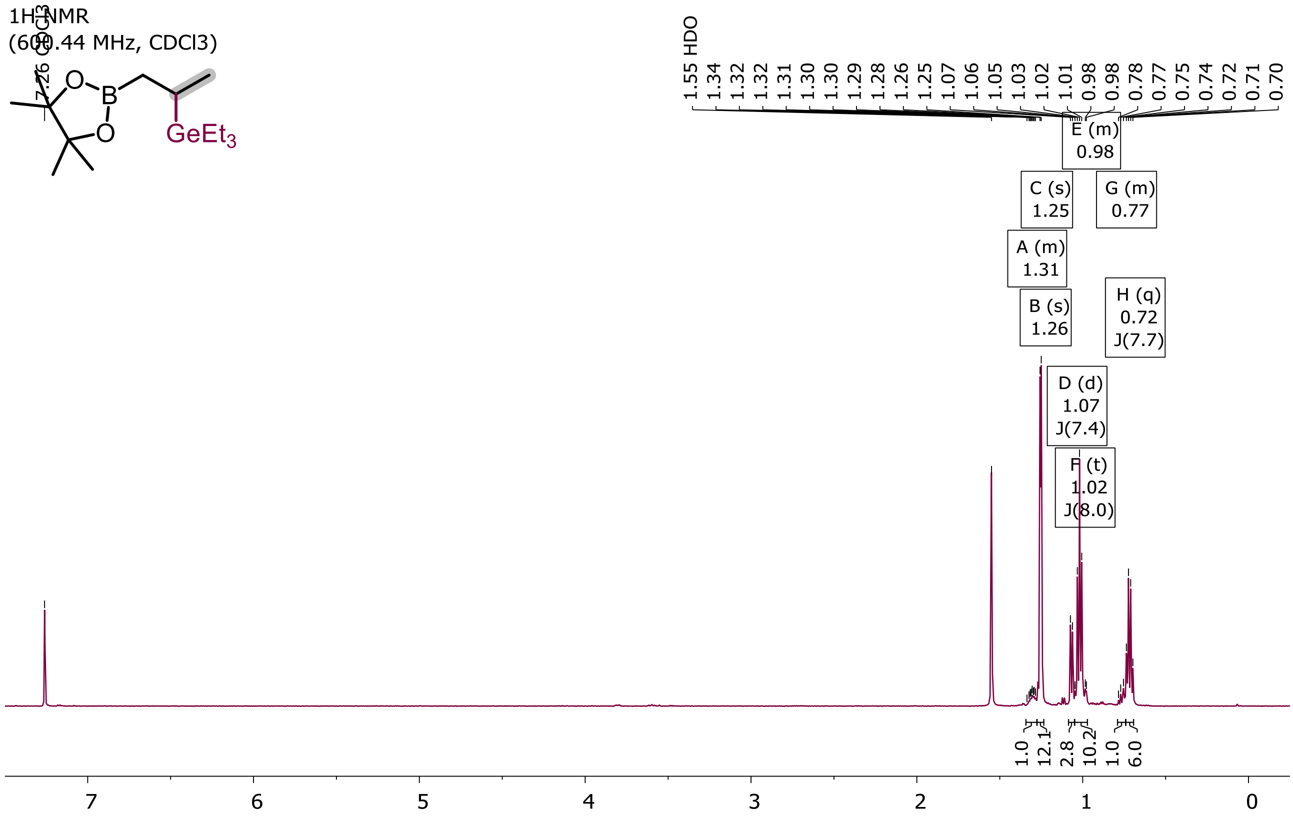

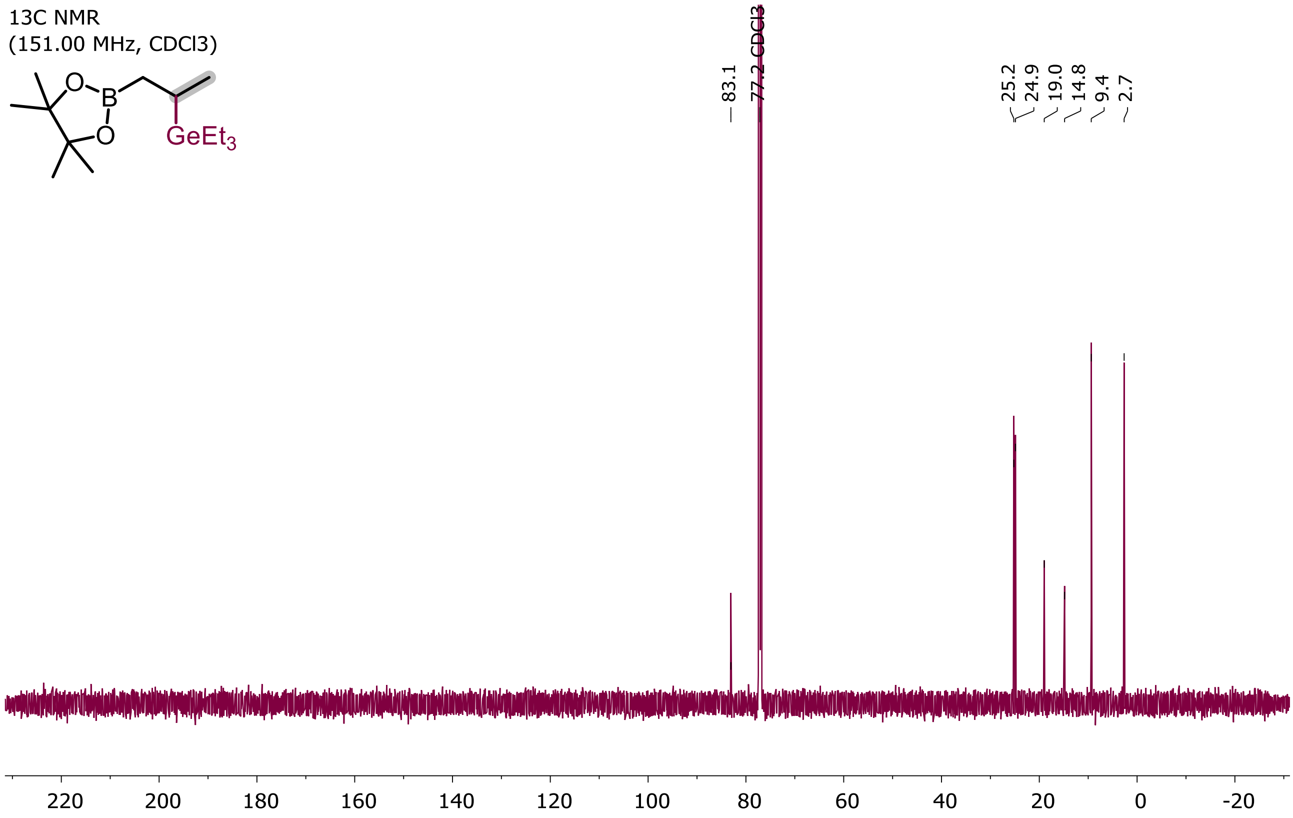

Diphenyl(2-(triethylgermyl)propyl)phosphine oxide (20)

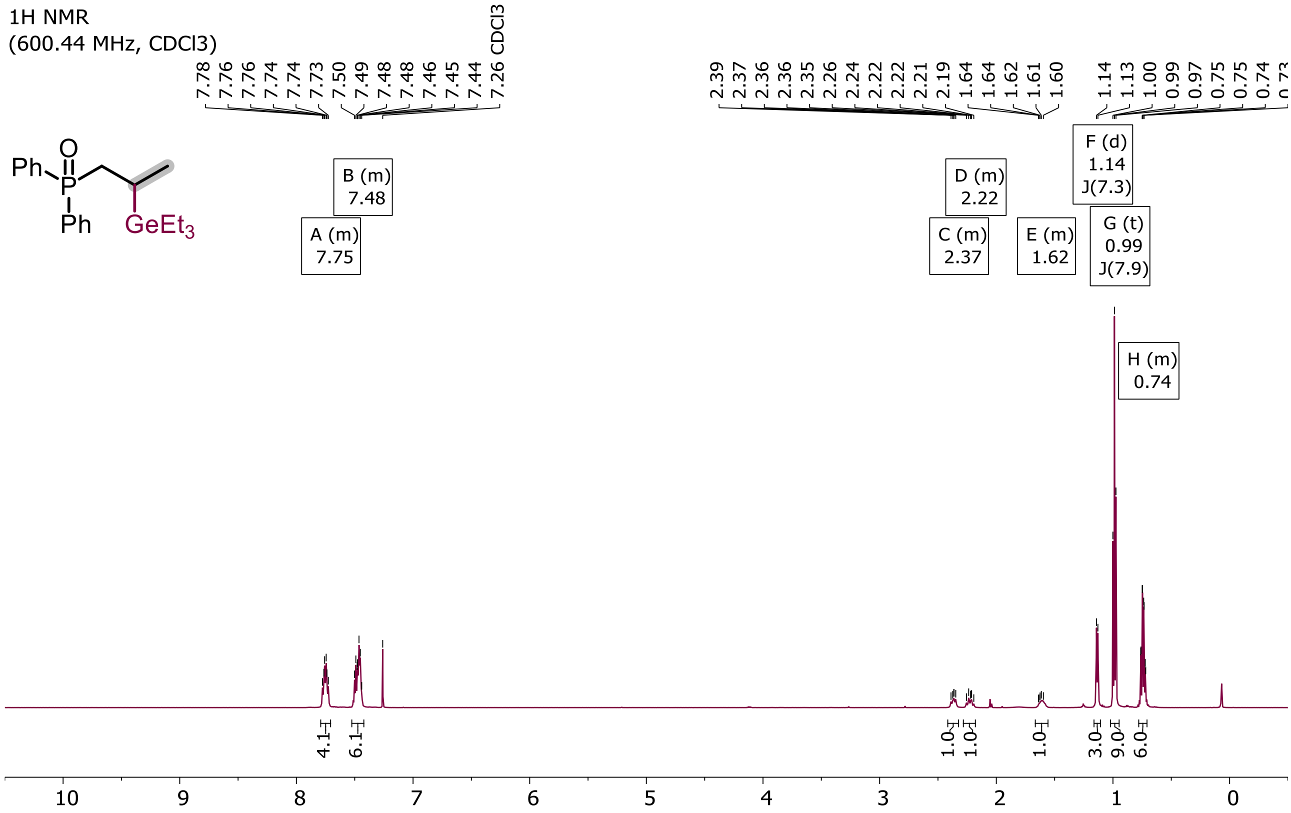

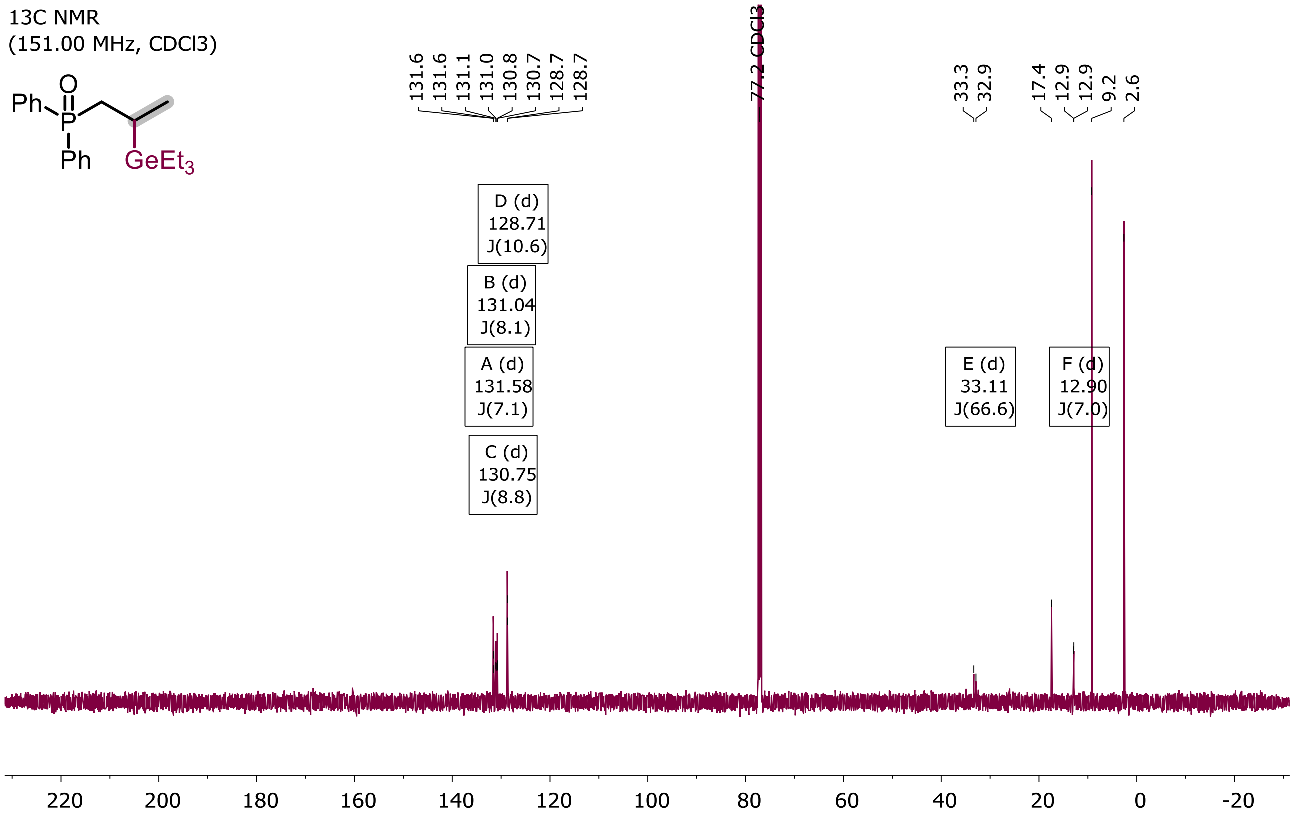

31P NMR
(243.05 MHz, CDCl3)

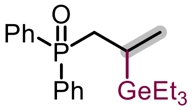

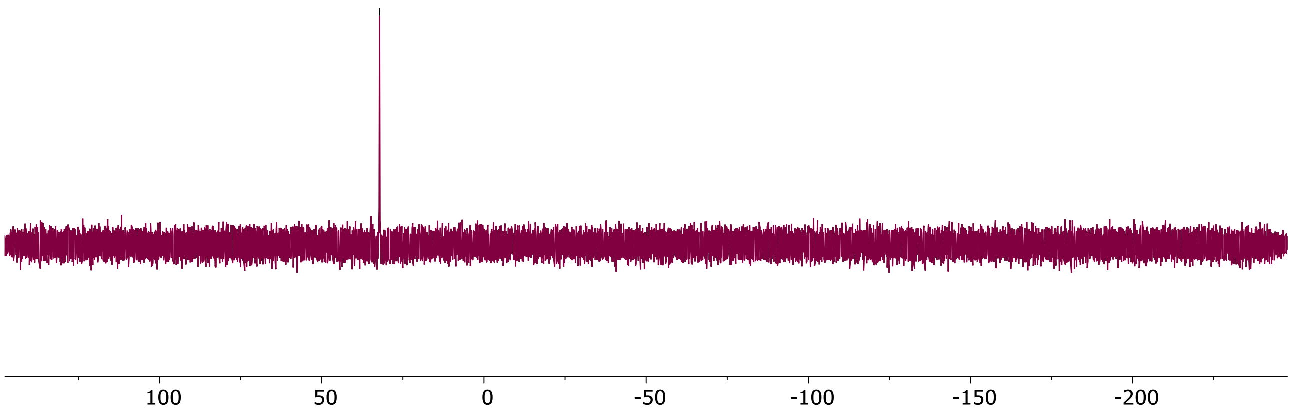

Triethyl(phenethyl)germane (21)

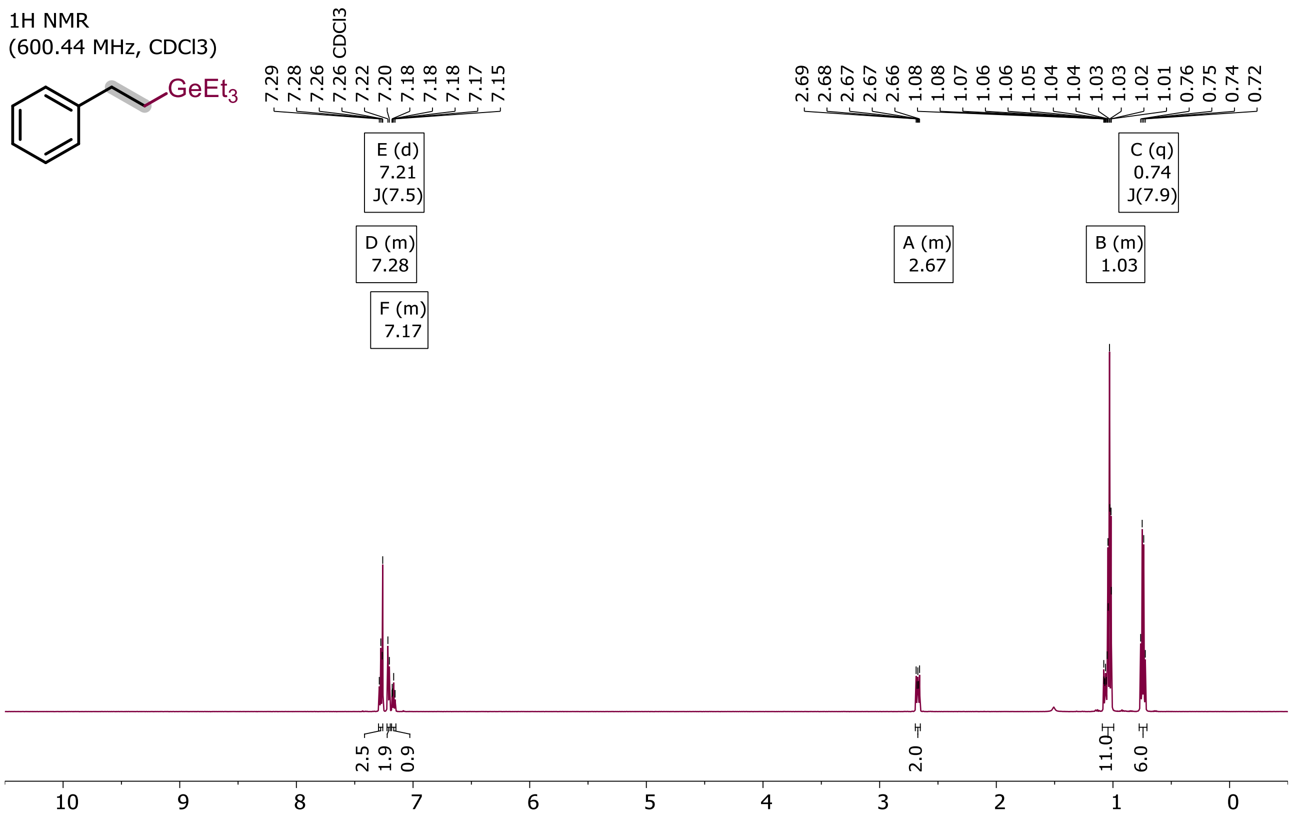

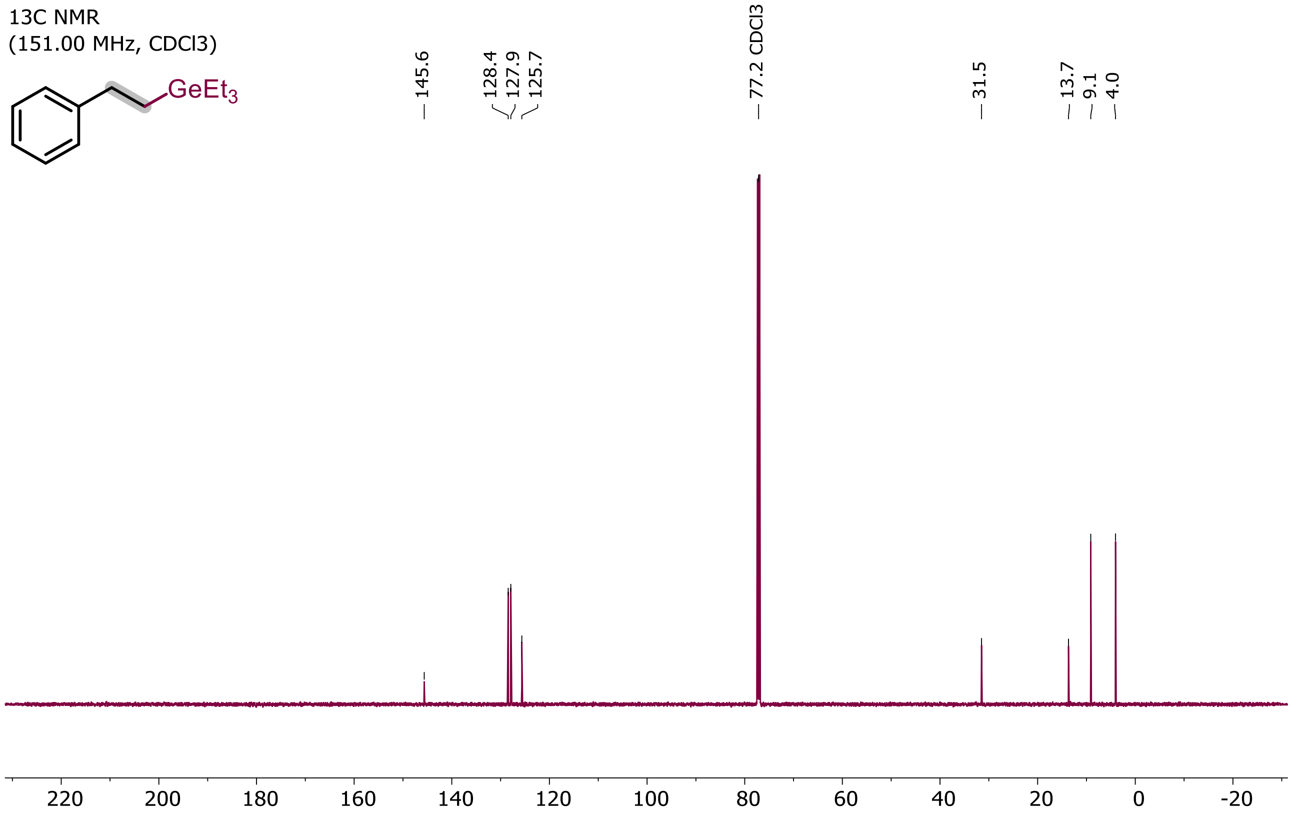

Triethyl(2-(phenyl-d5)ethyl-1,1,2,2-d4)germane (21-d_9)

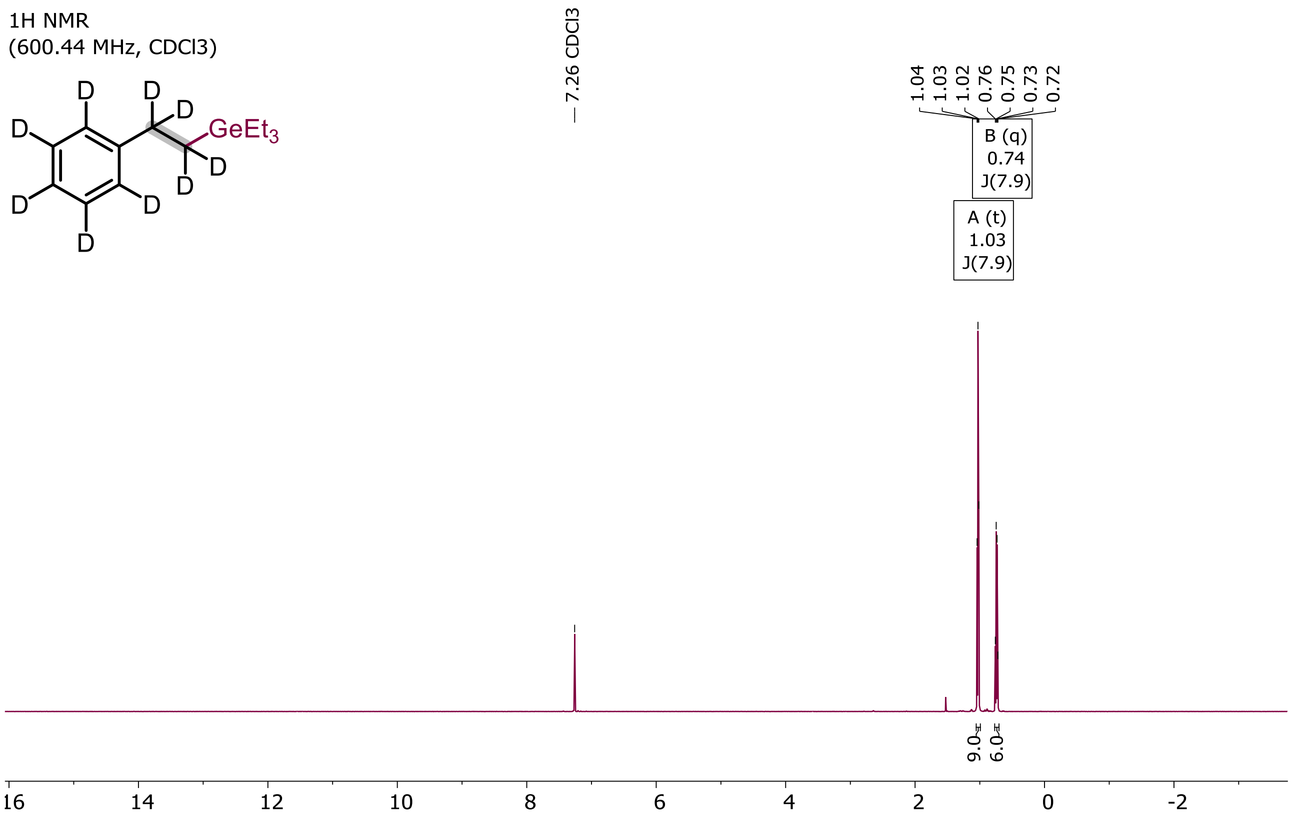

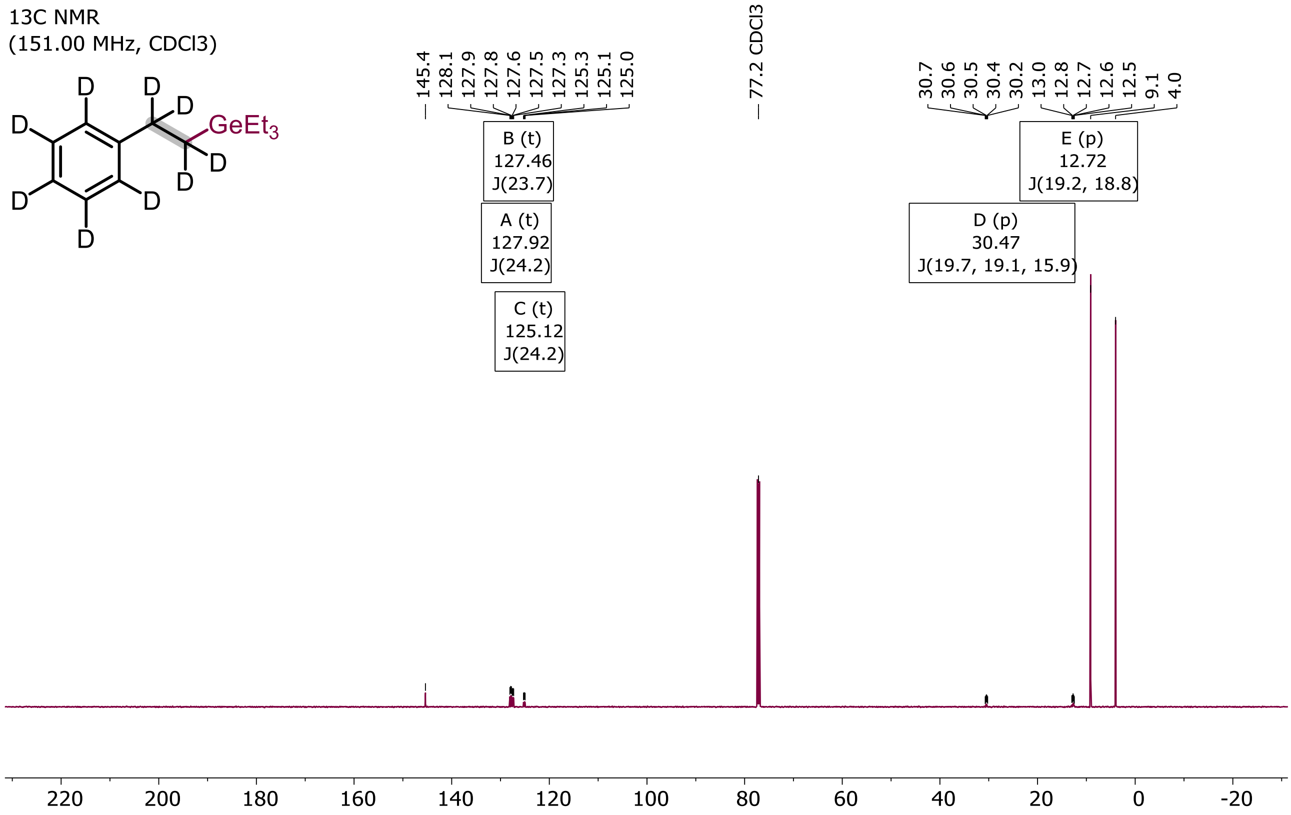

Triethyl(2-(phenyl-d_5)ethyl-1,1-d_2)germane (21-d_7)

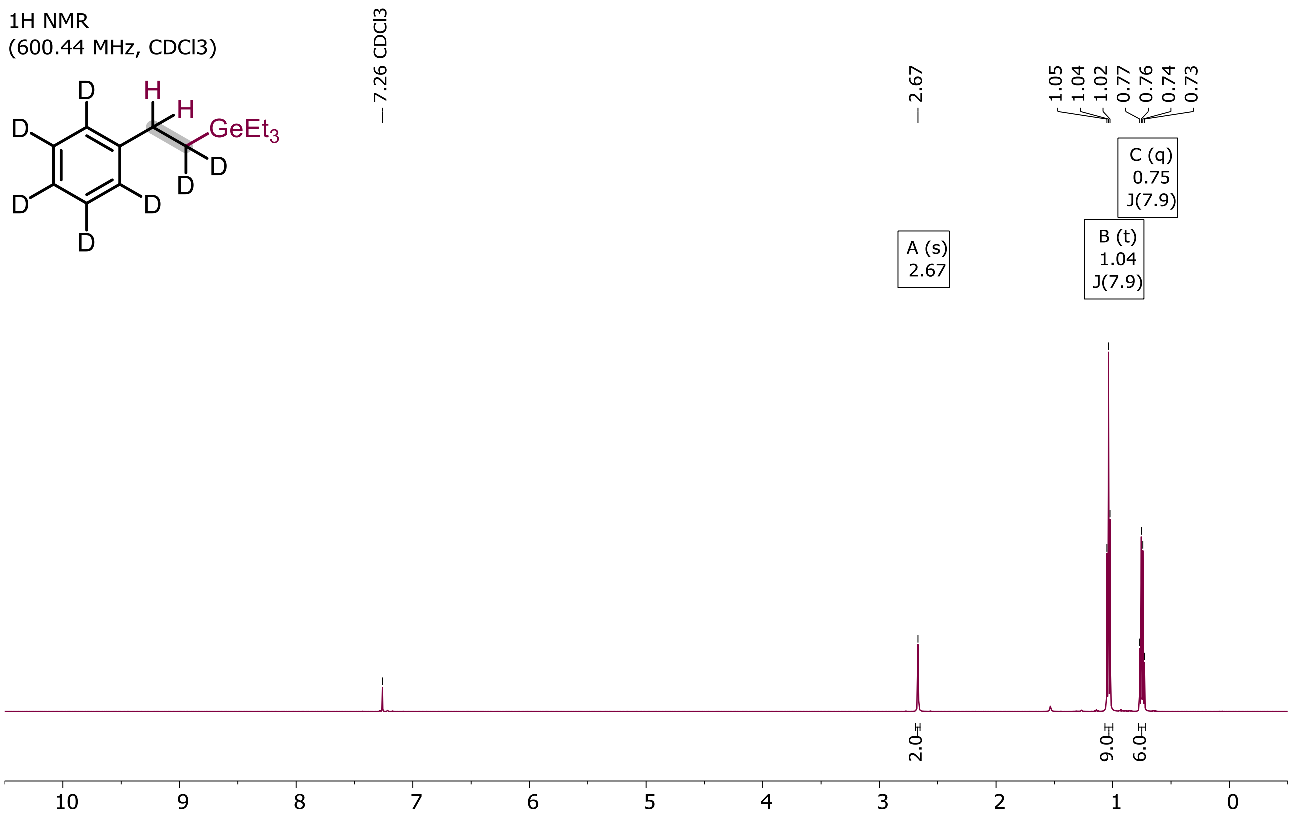

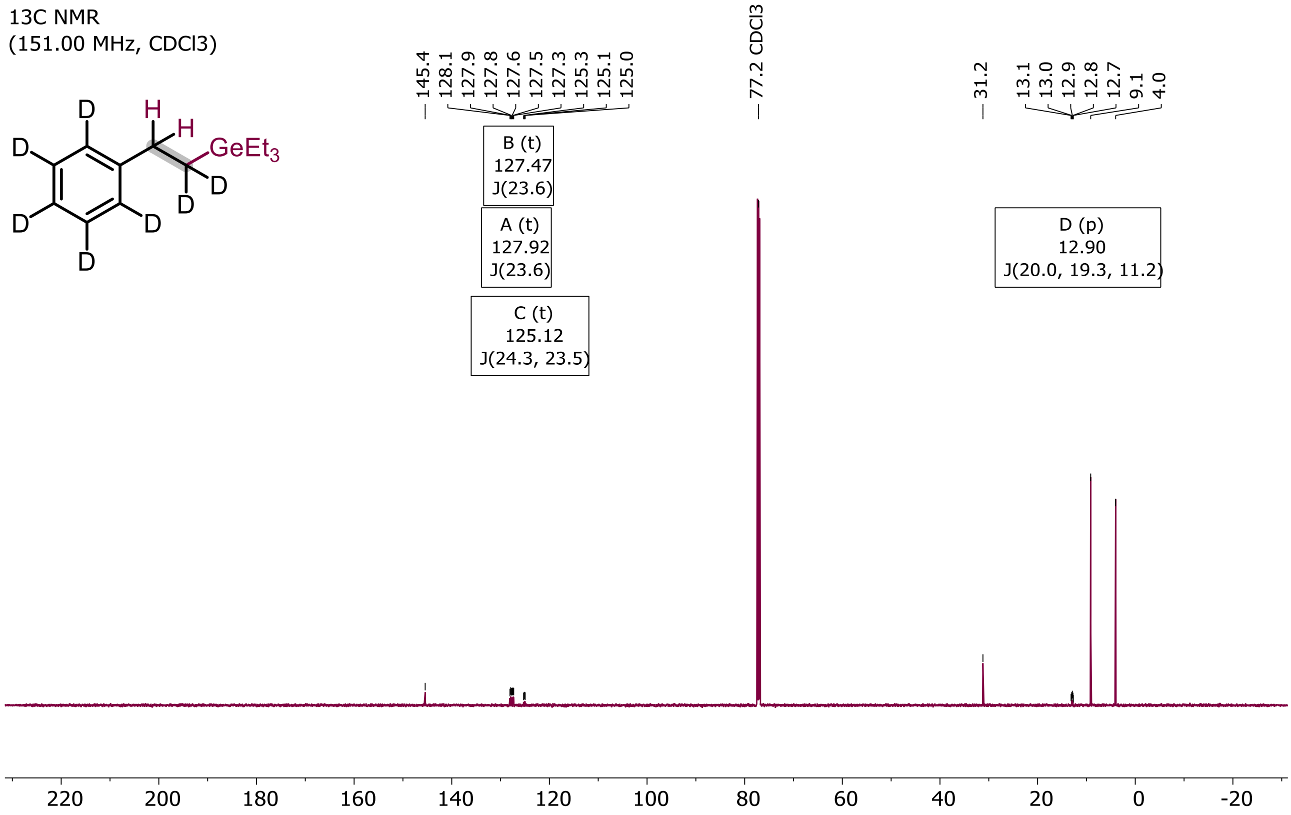

(4-(*tert*-Butyl)phenethyl)triethylgermane (22)

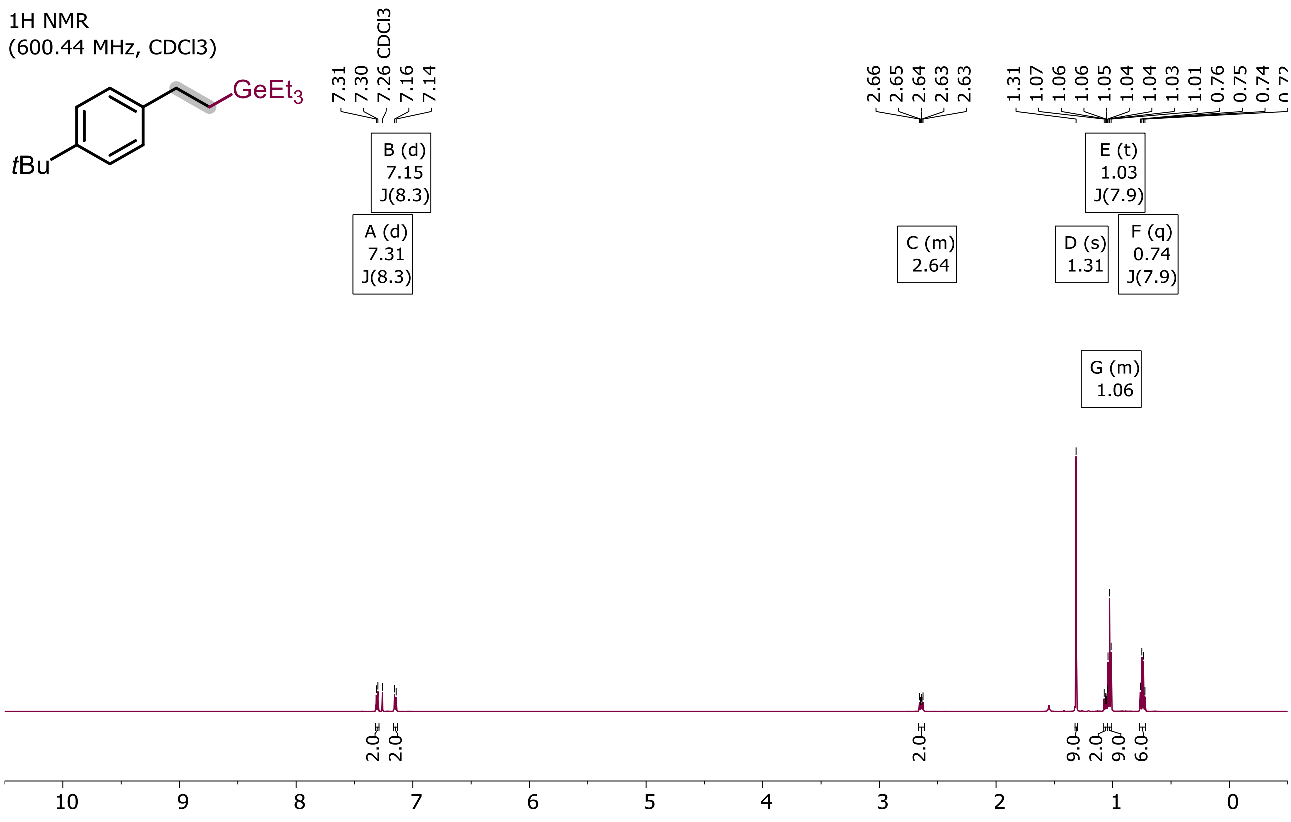

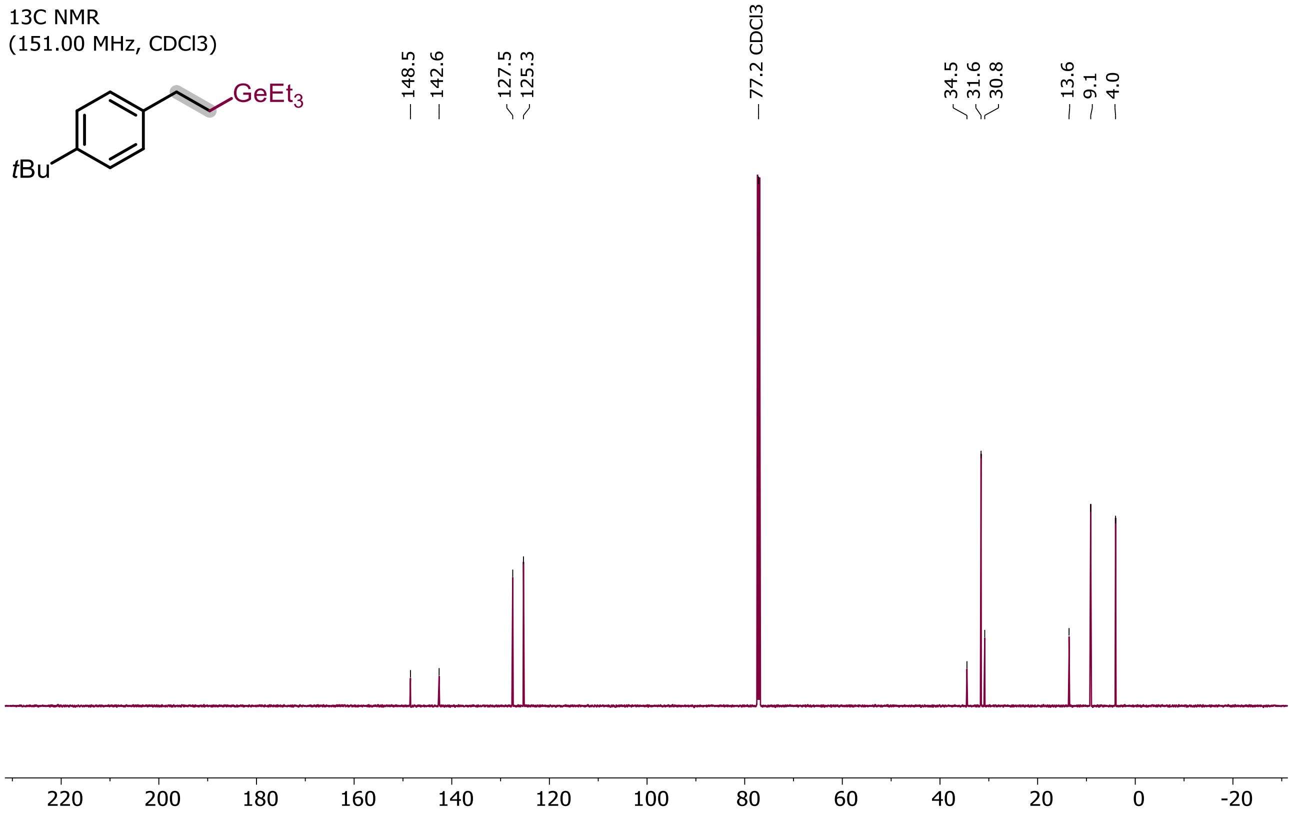

(2,2-Diphenylethyl)triethylgermane (23)

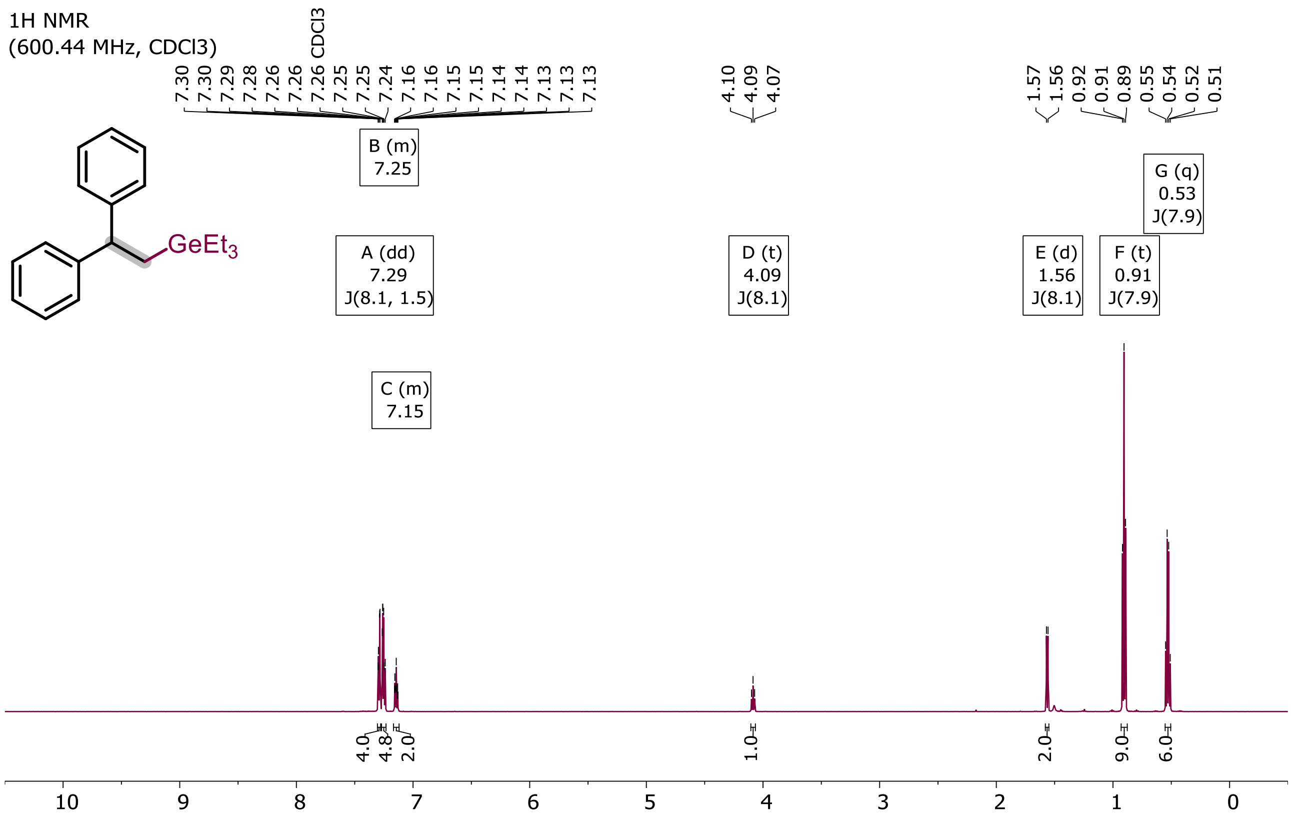

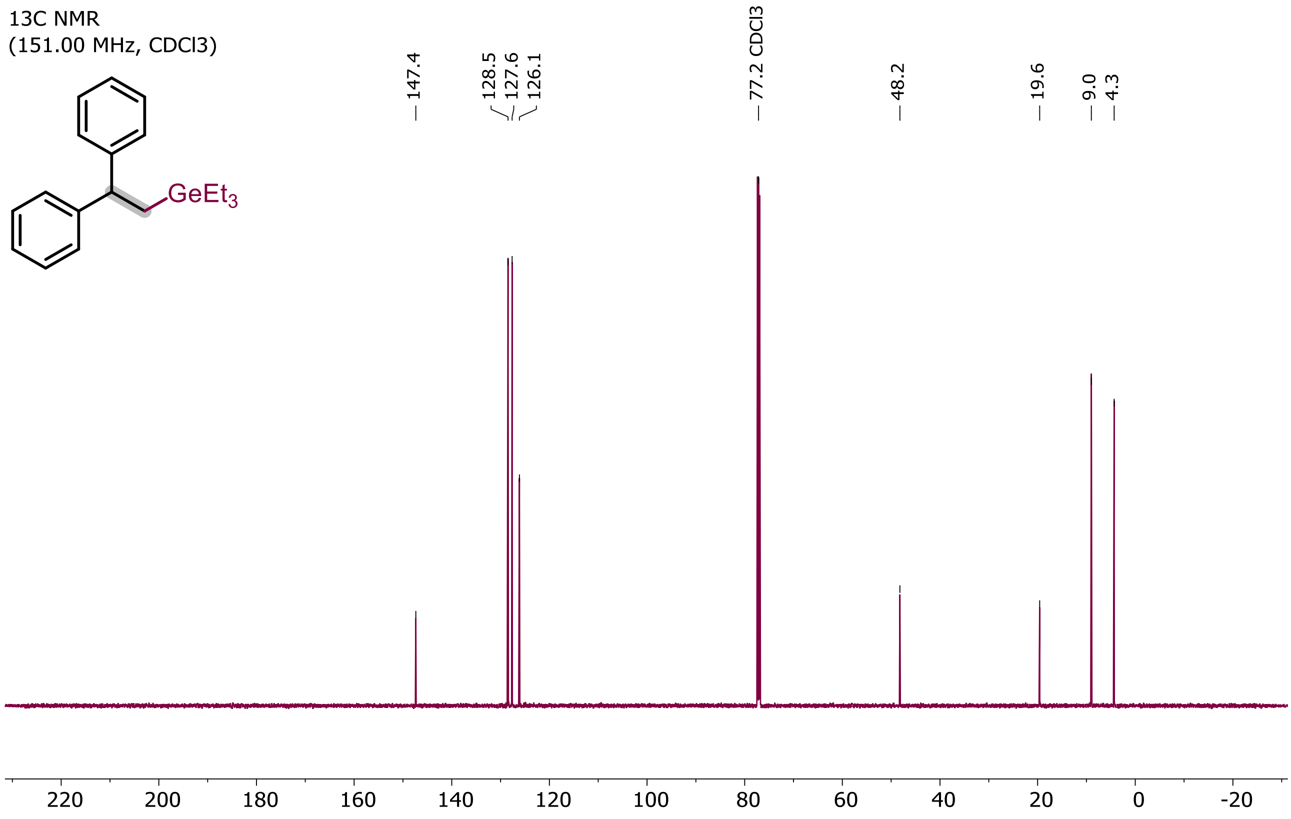

Triethyl(4-fluorophenethyl)germane (24)

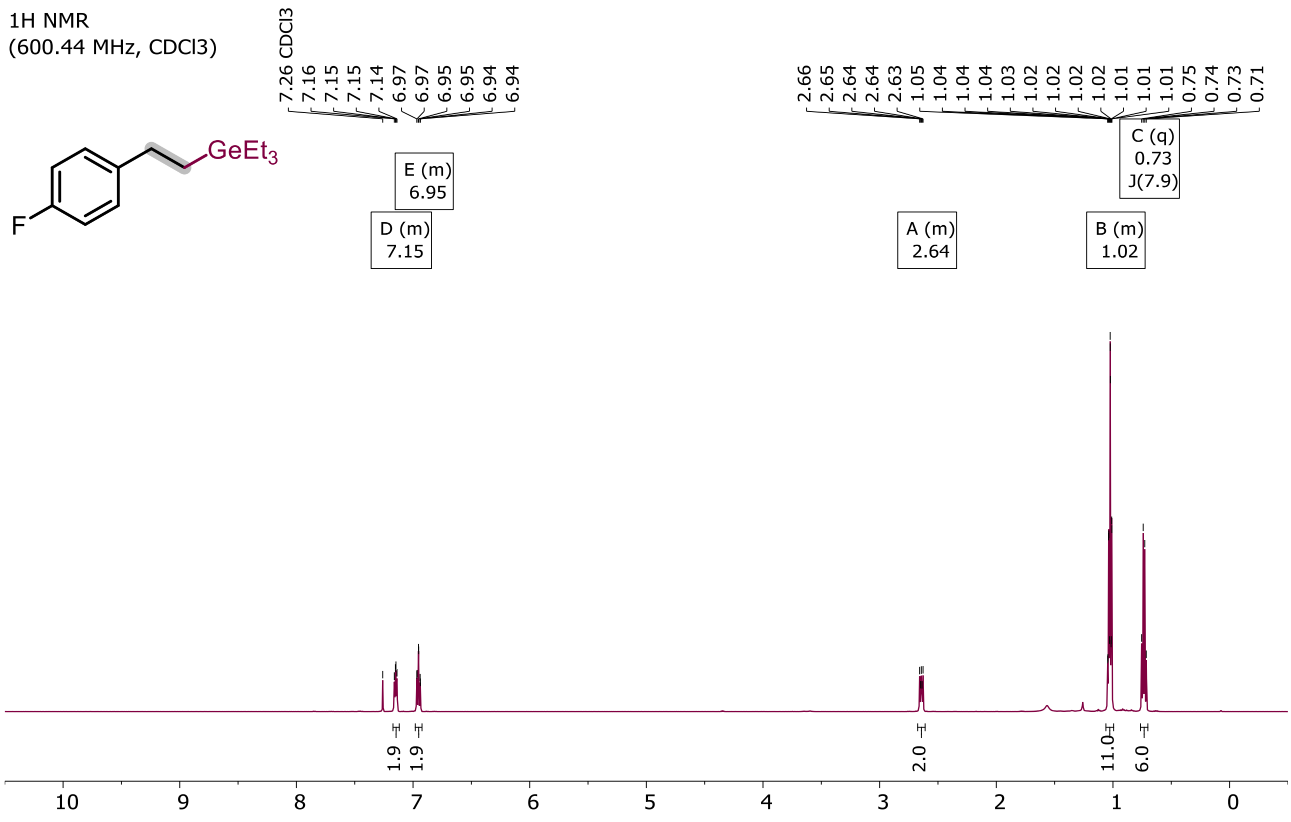

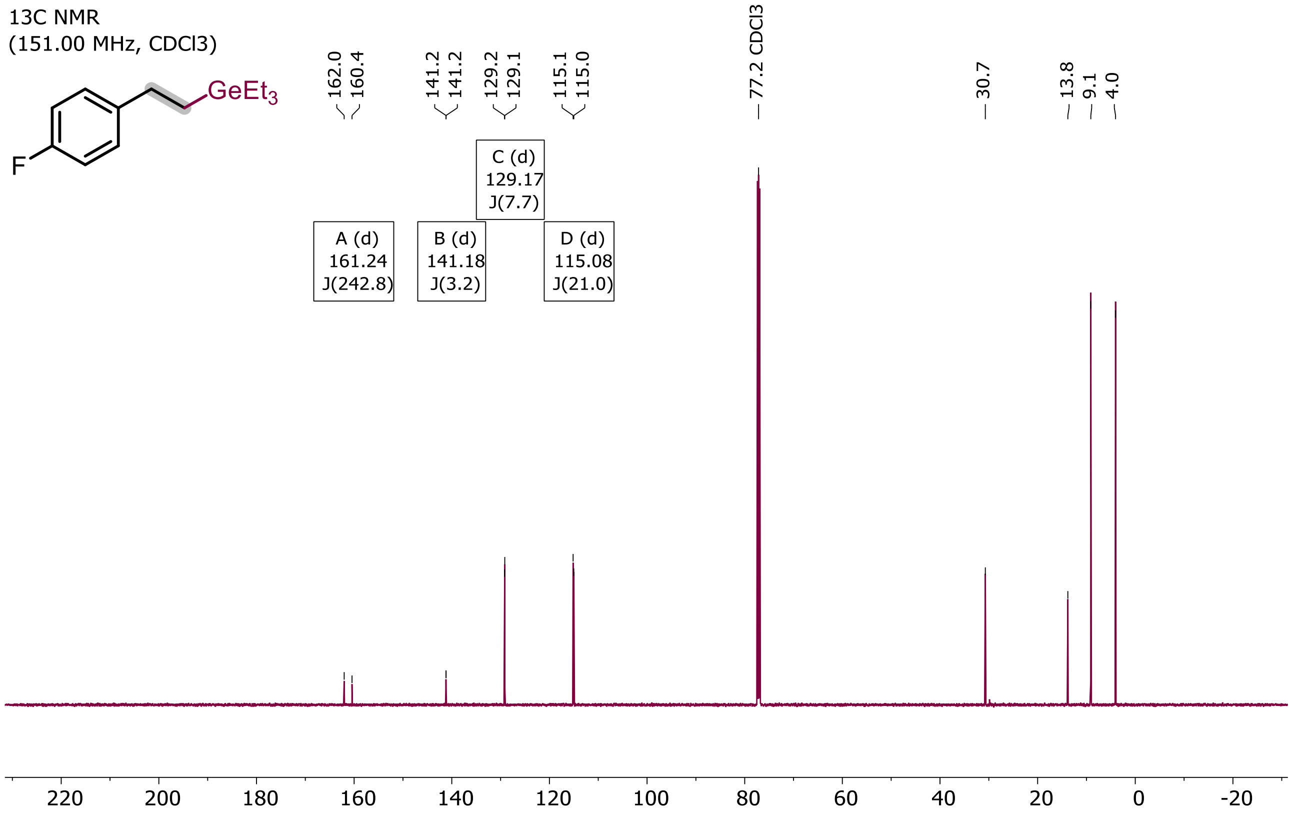

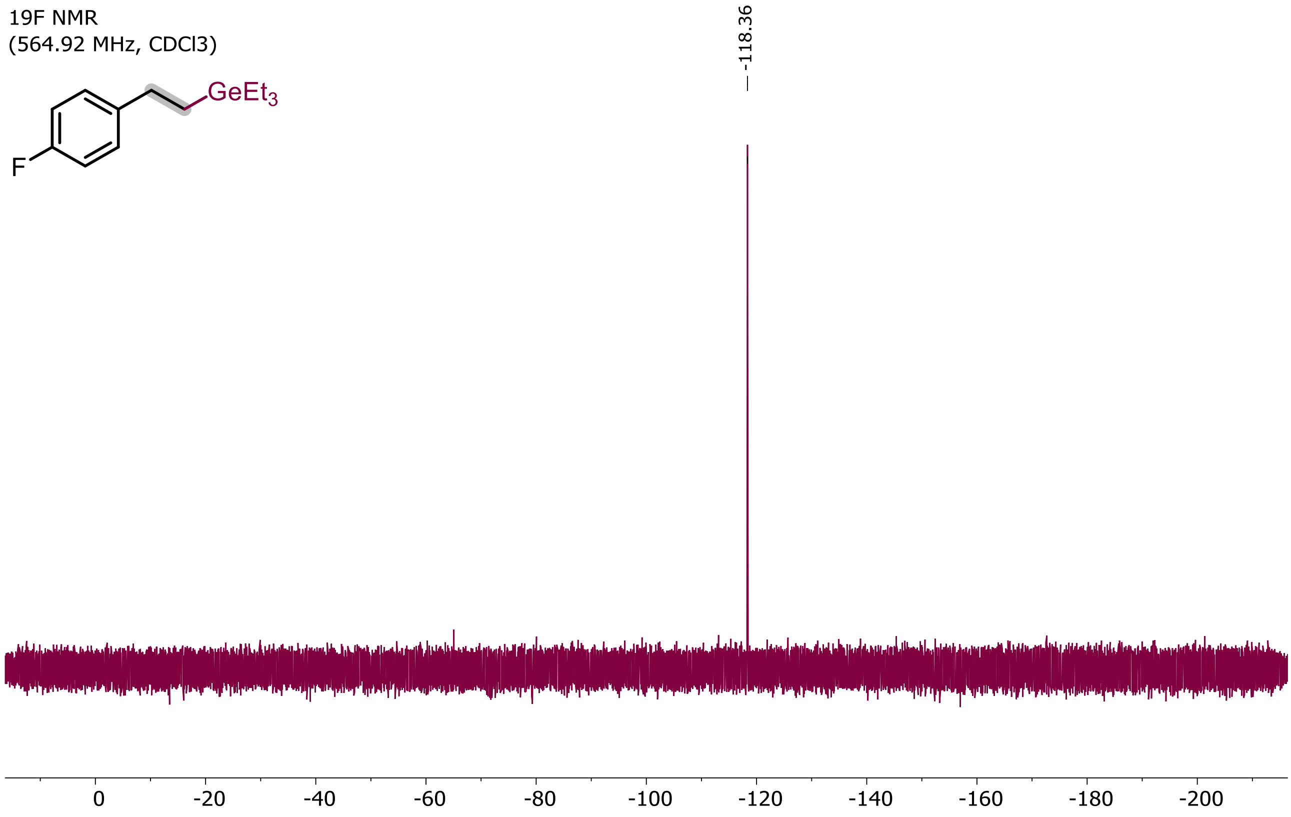

Triethyl(4-(trifluoromethyl)phenethyl)germane (25)

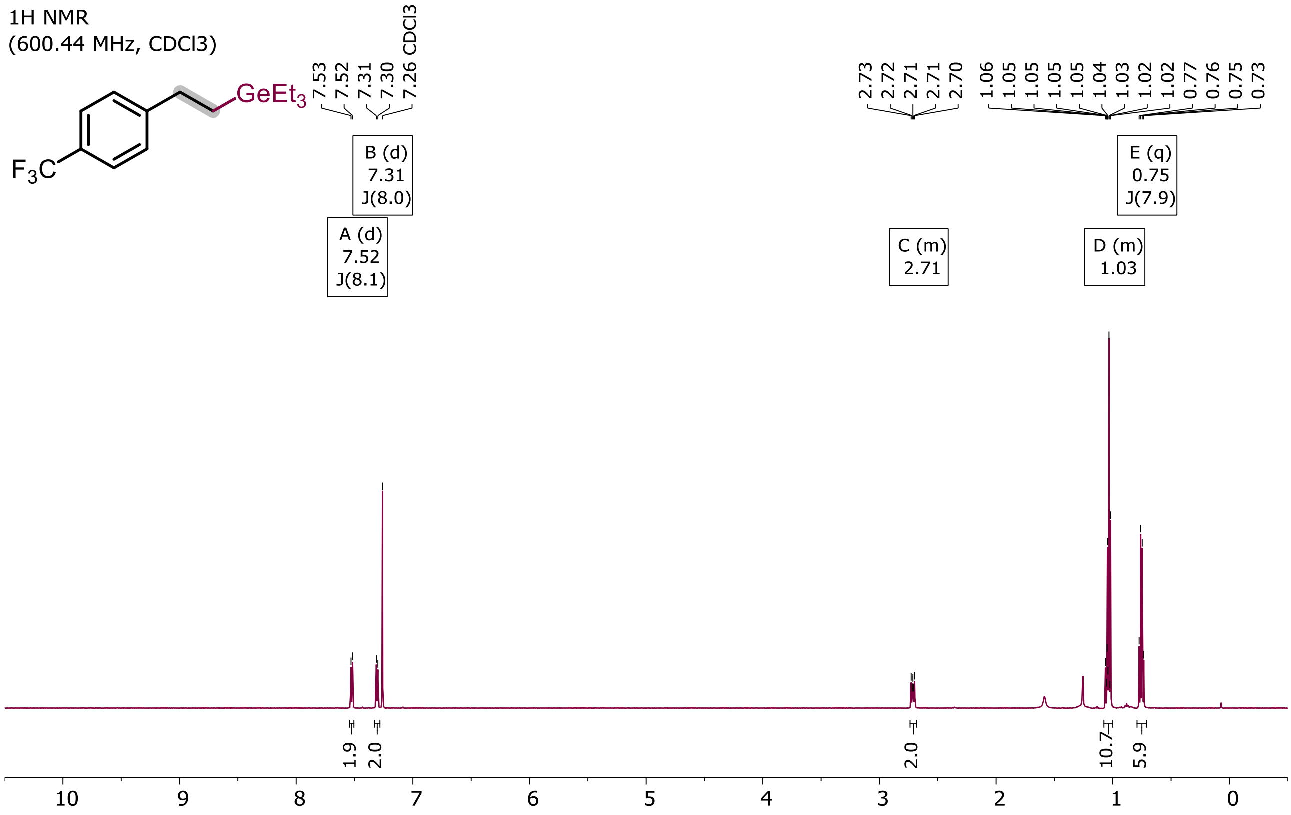

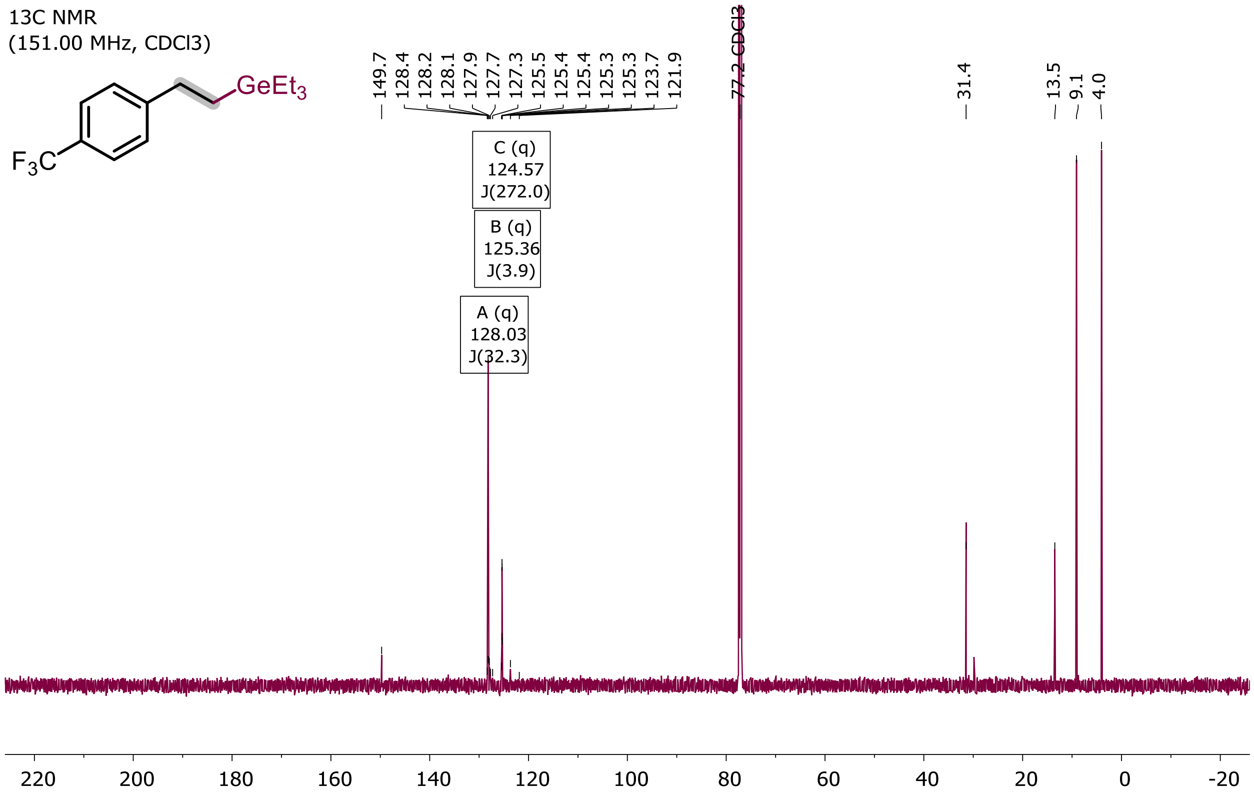
13C NMR
(151.00 MHz, CDCl3)
GeEt3
F3C
149.7
128.4
128.2
128.1
127.9
127.7
127.3
125.5
125.4
125.4
125.3
125.3
123.7
121.9
77.2 CDCl3
31.4
13.5
9.1
4.0
C (q)
124.57
J(272.0)
B (q)
125.36
J(3.9)
A (q)
128.03
J(32.3)
220
200
180
160
140
120
100
80
60
40
20
0
-20

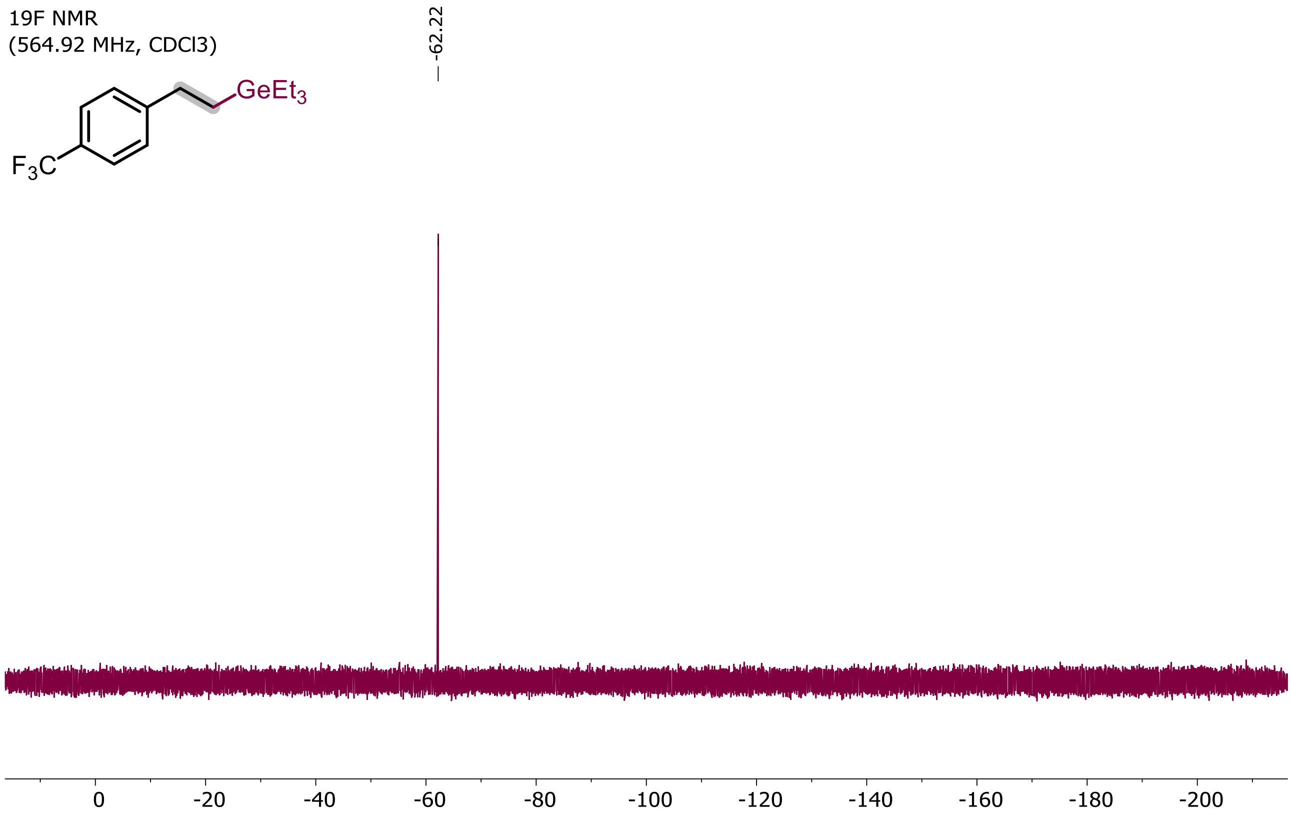
19F NMR
(564.92 MHz, CDCl3)
GeEt3
F3C
-62.22
0
-20
-40
-60
-80
-100
-120
-140
-160
-180
-200

Triethyl(1-(3-tolyl)butan-2-yl)germane (26)

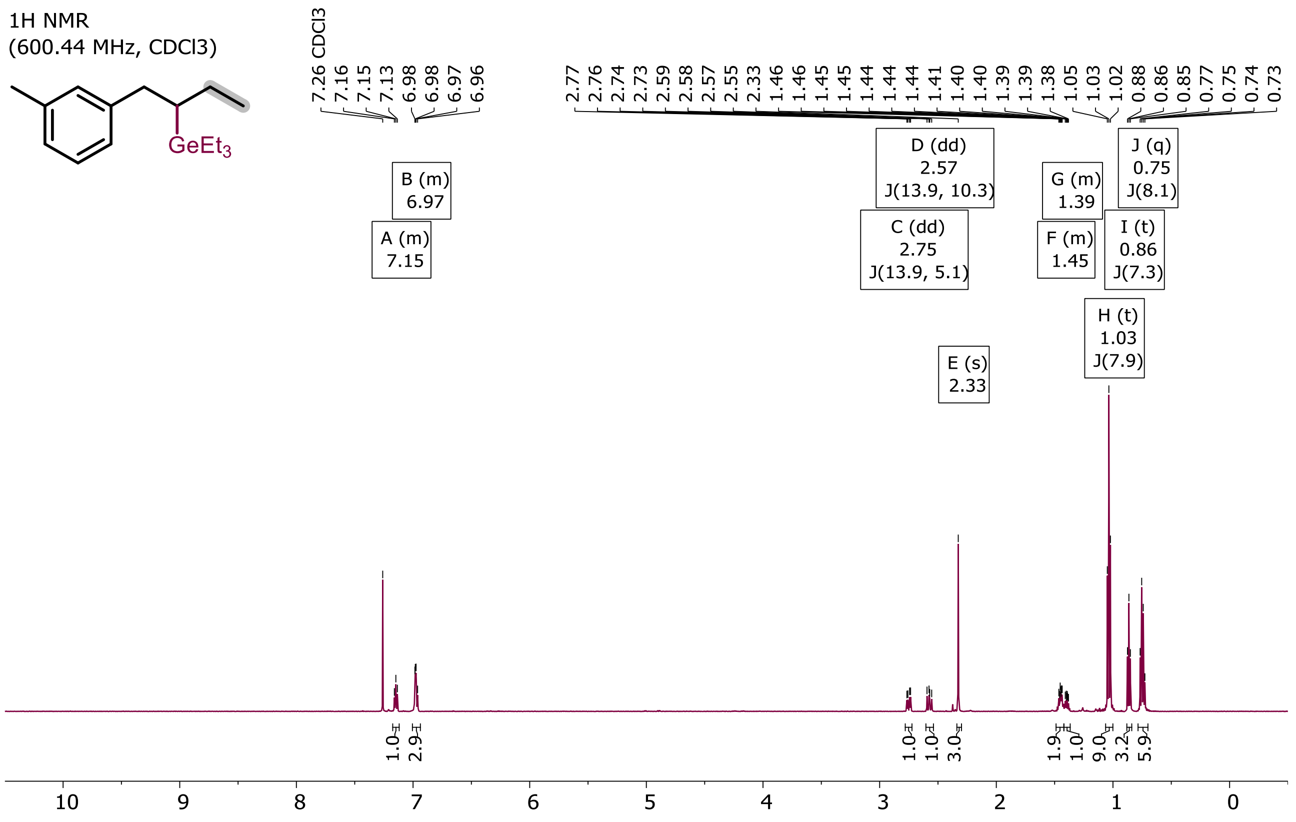

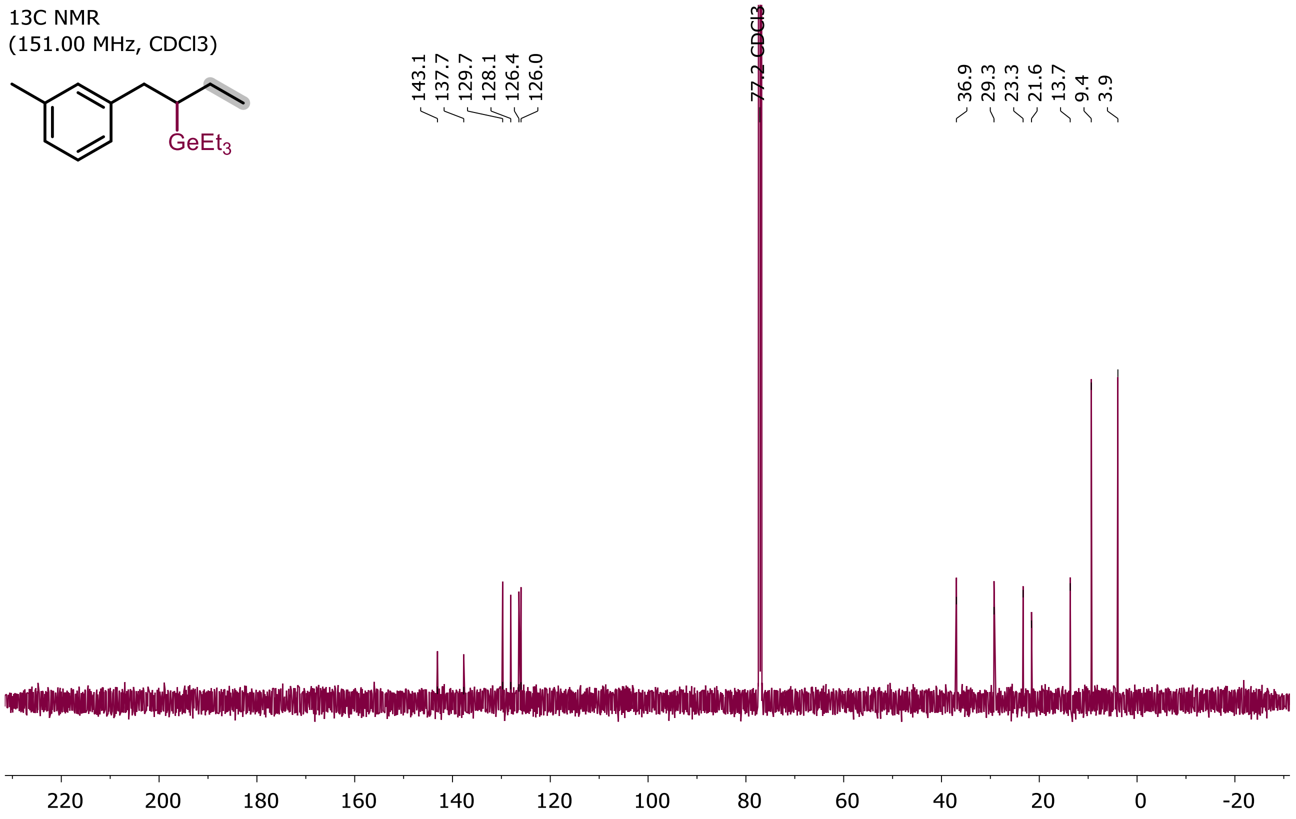

(1-(4-(*tert*-Butyl)phenyl)butan-2-yl)triethylgermane (27)

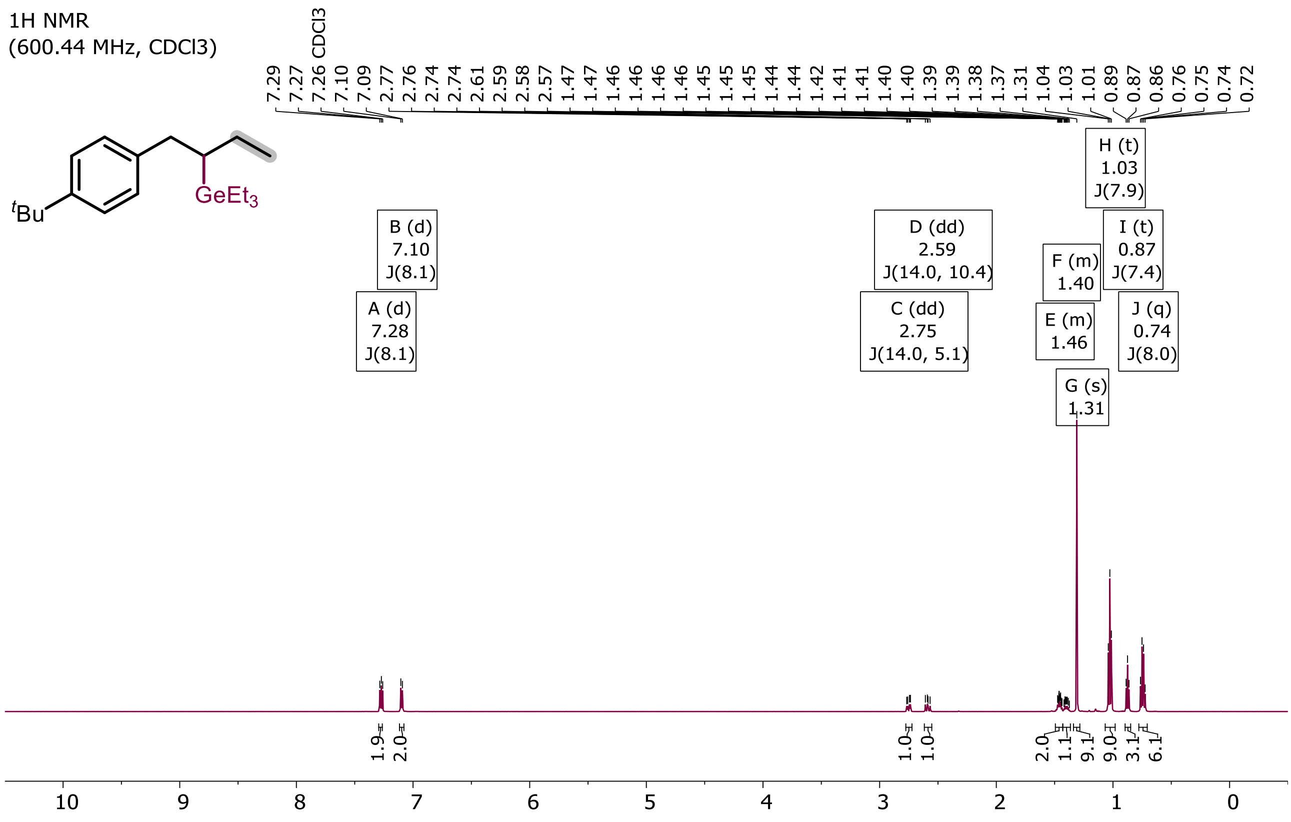

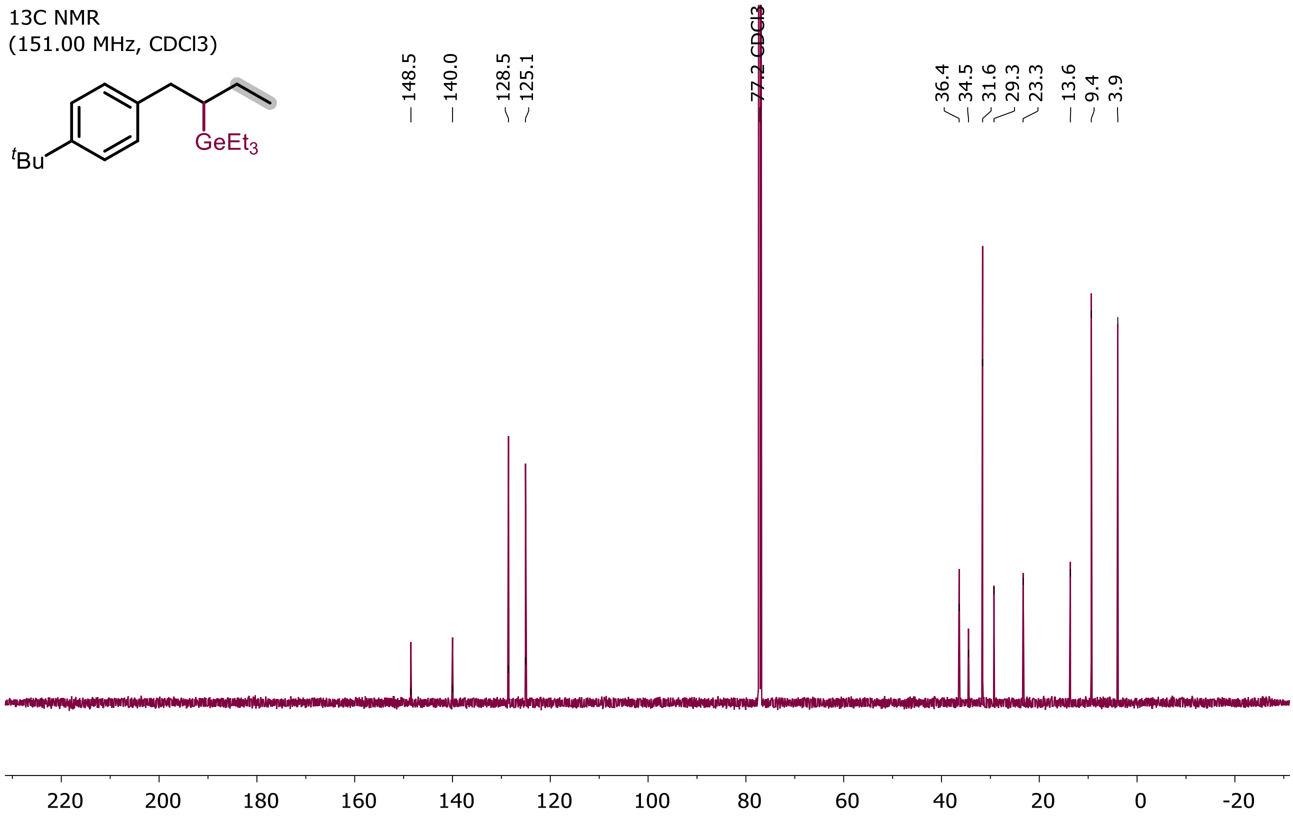

(1-([1,1'-Biphenyl]-3-yl)butan-2-yl)triethylgermane (28)

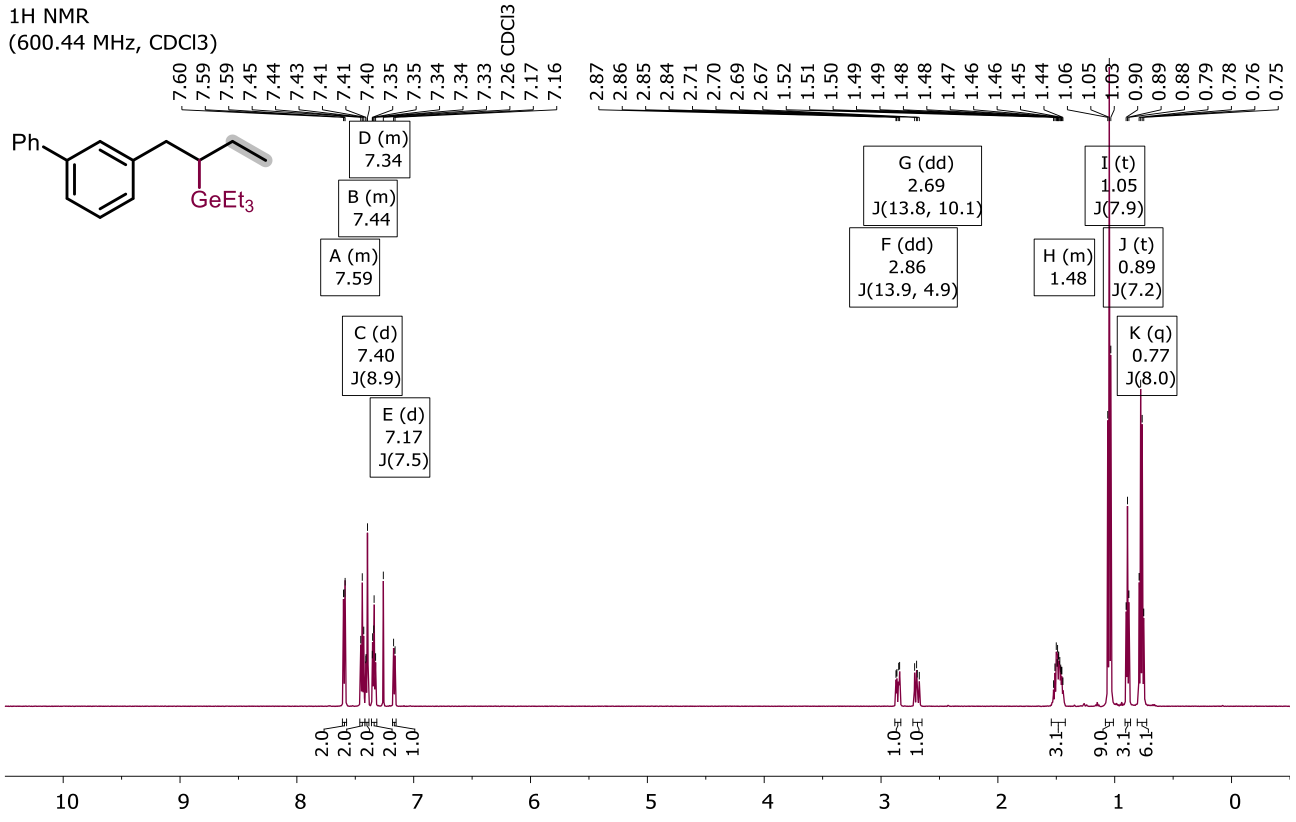

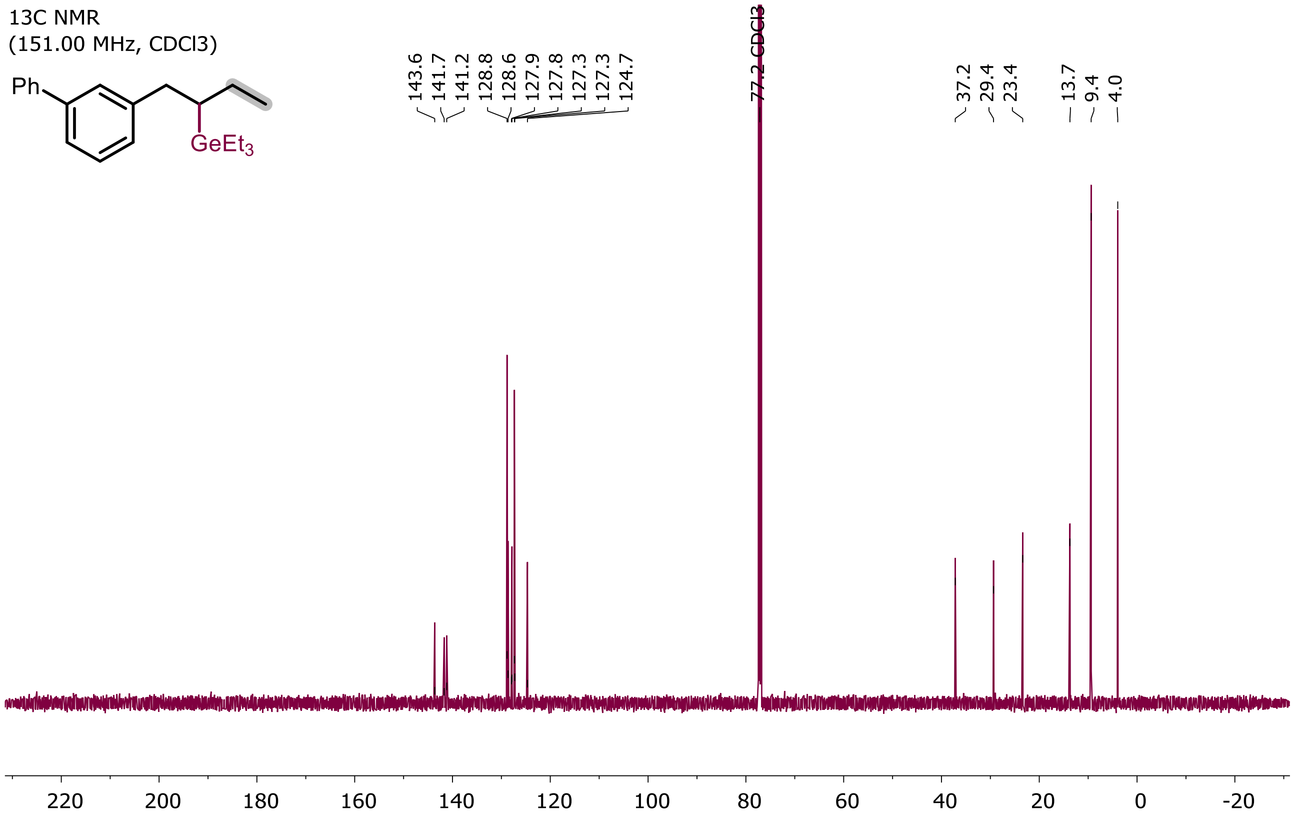

Triethyl(1-(naphthalen-2-yl)butan-2-yl)germane (29)

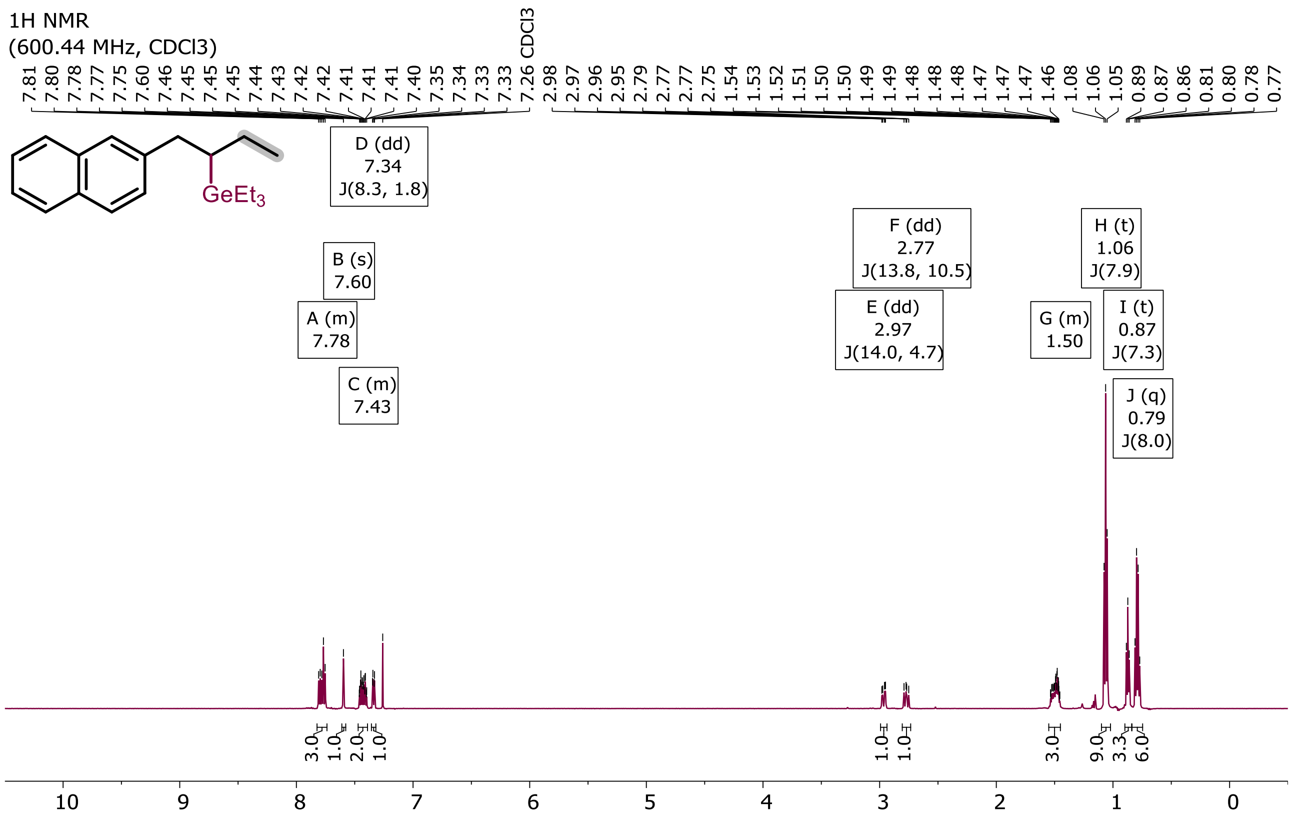

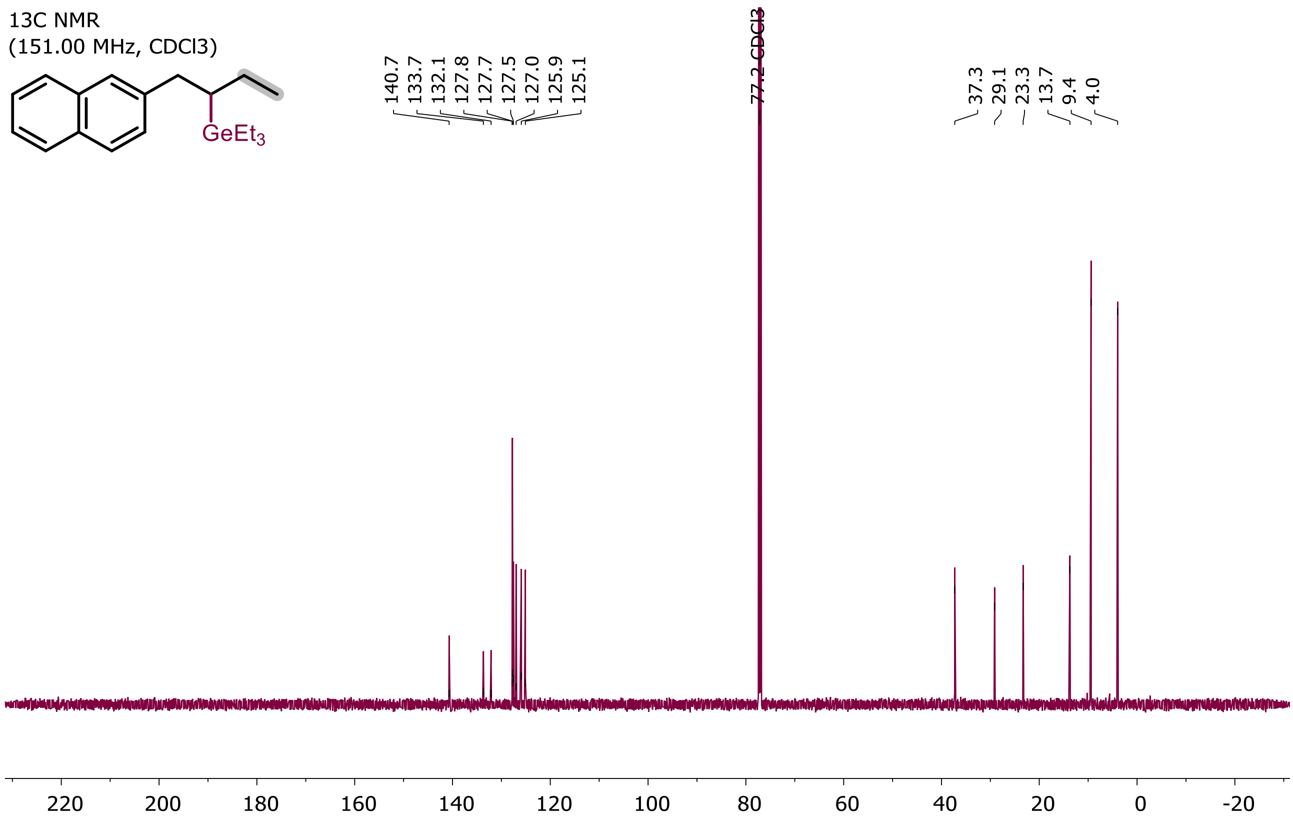

Triethyl(1-(4-fluorophenyl)butan-2-yl)germane (30)

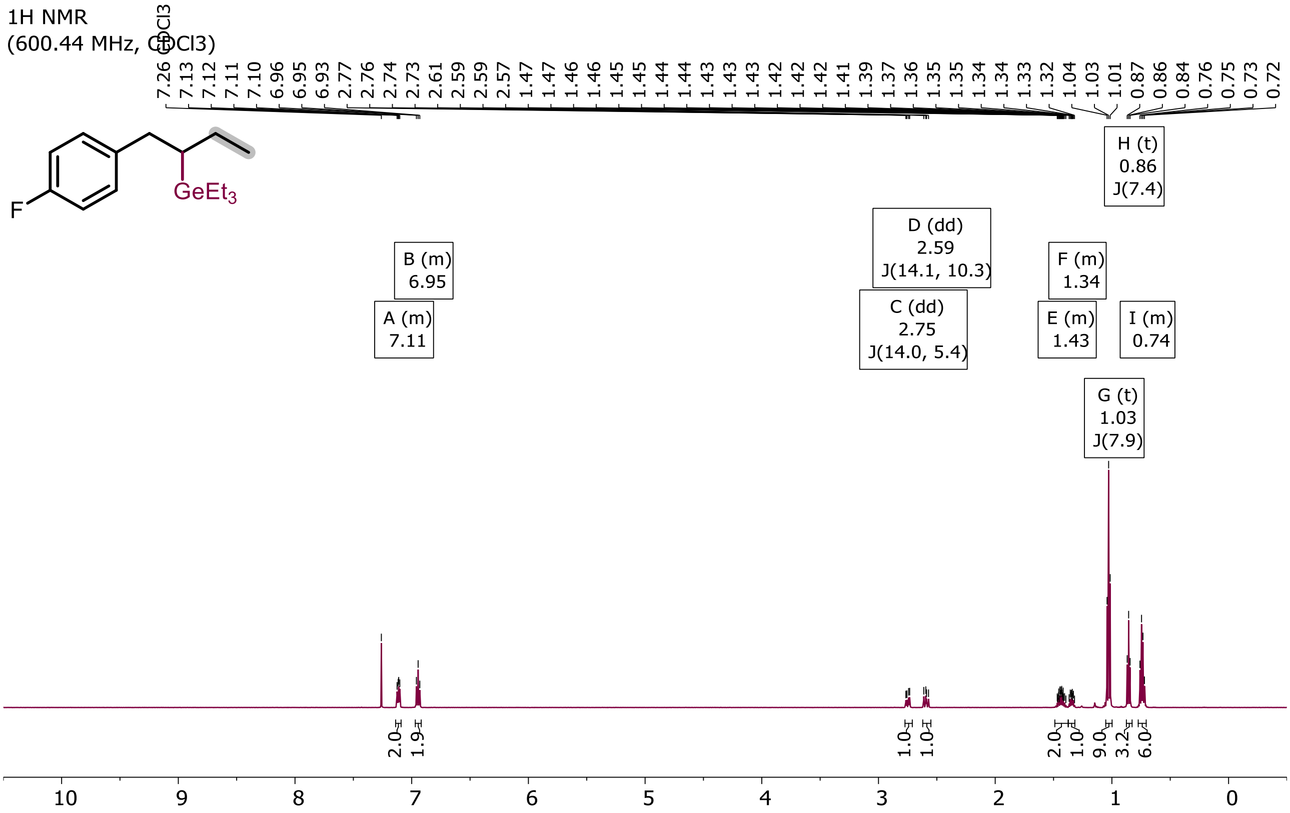

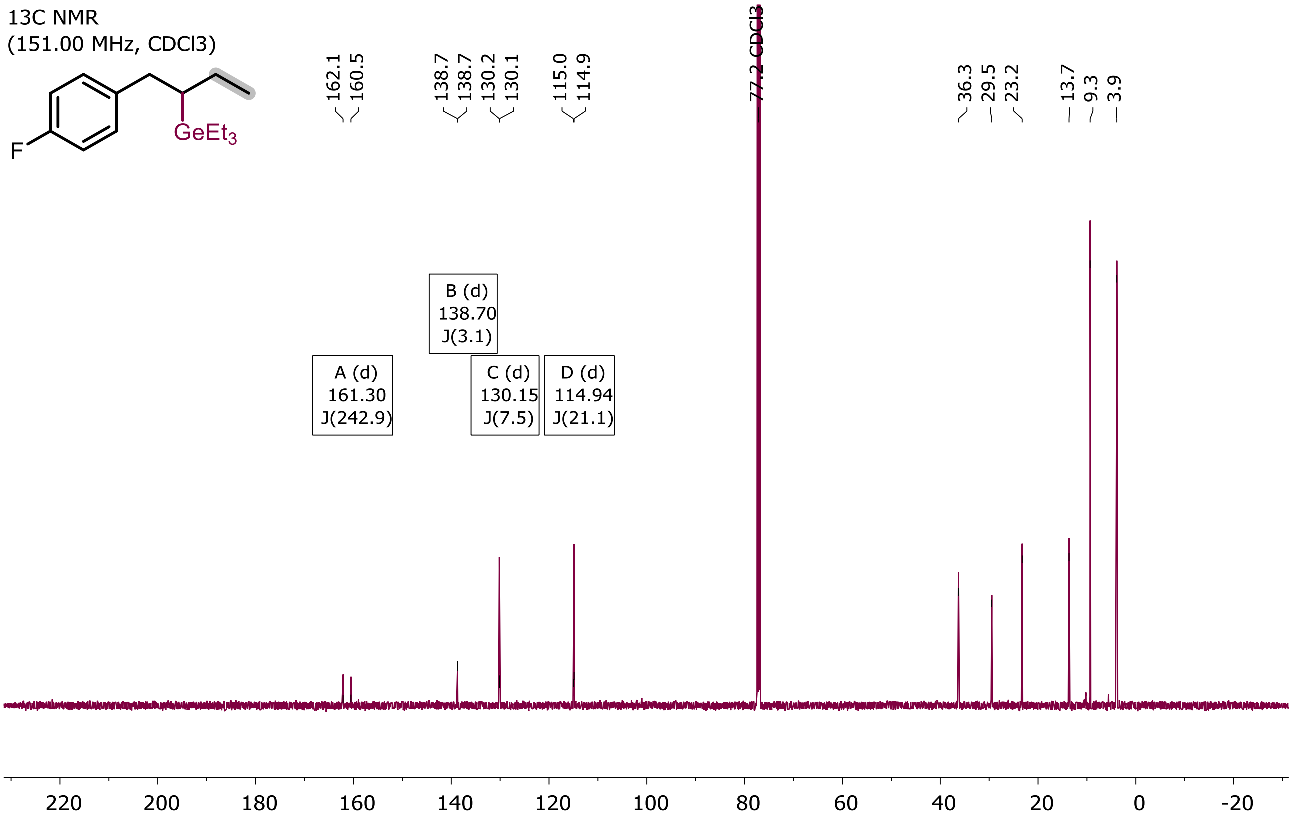

19F NMR
(564.92 MHz, CDCl3)

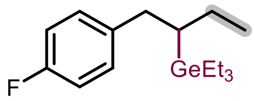

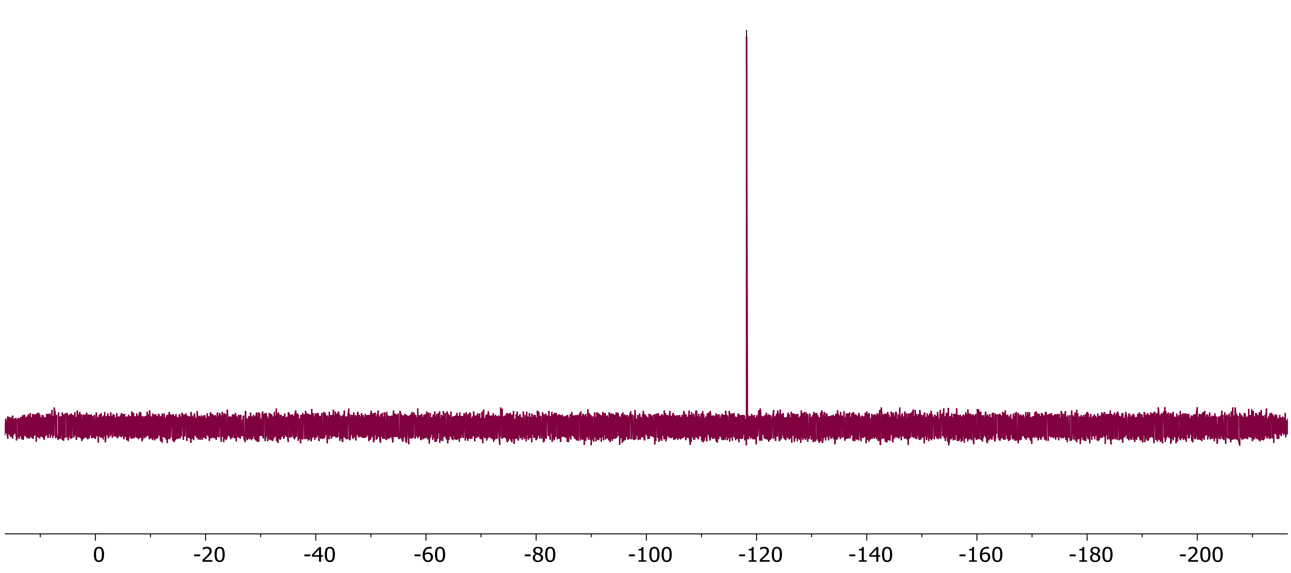

Triethyl(1-(3-methoxyphenyl)butan-2-yl)germane (31)

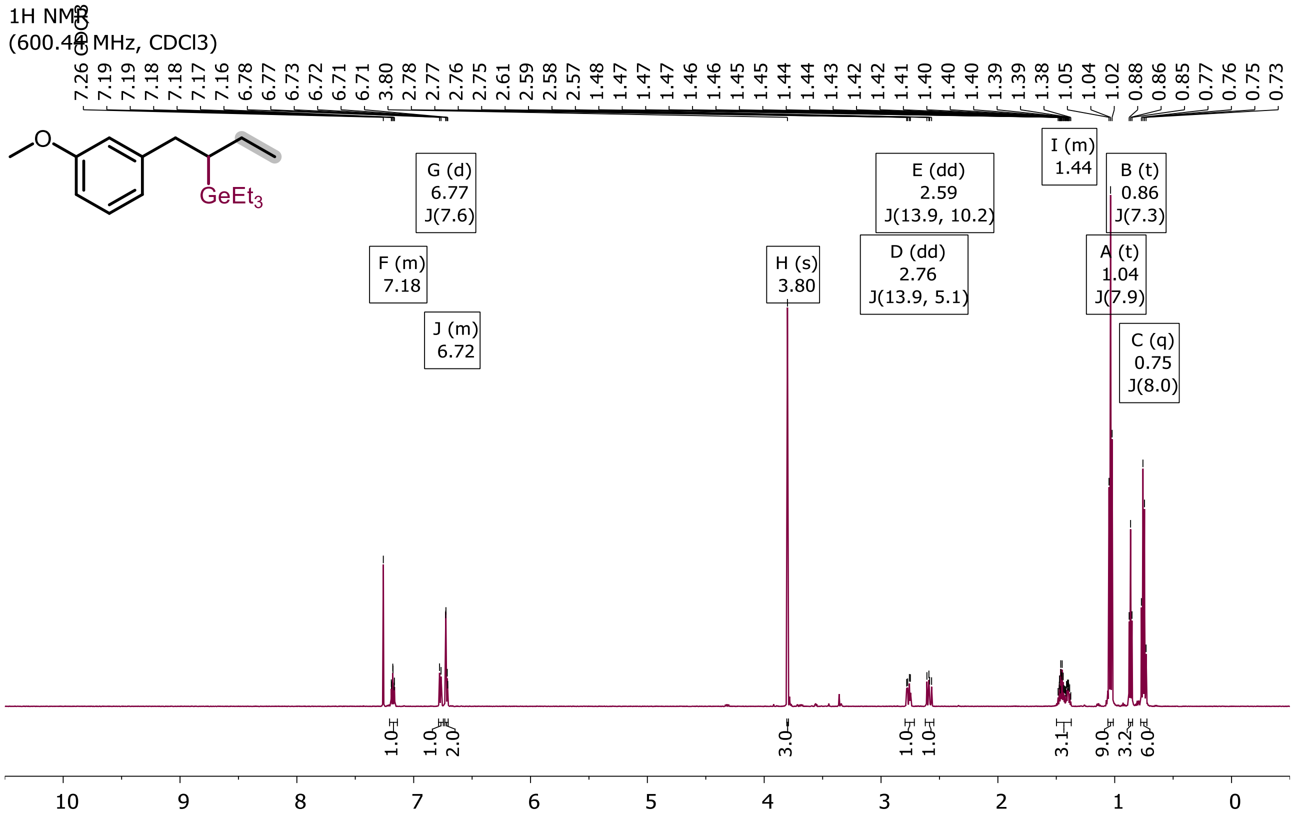

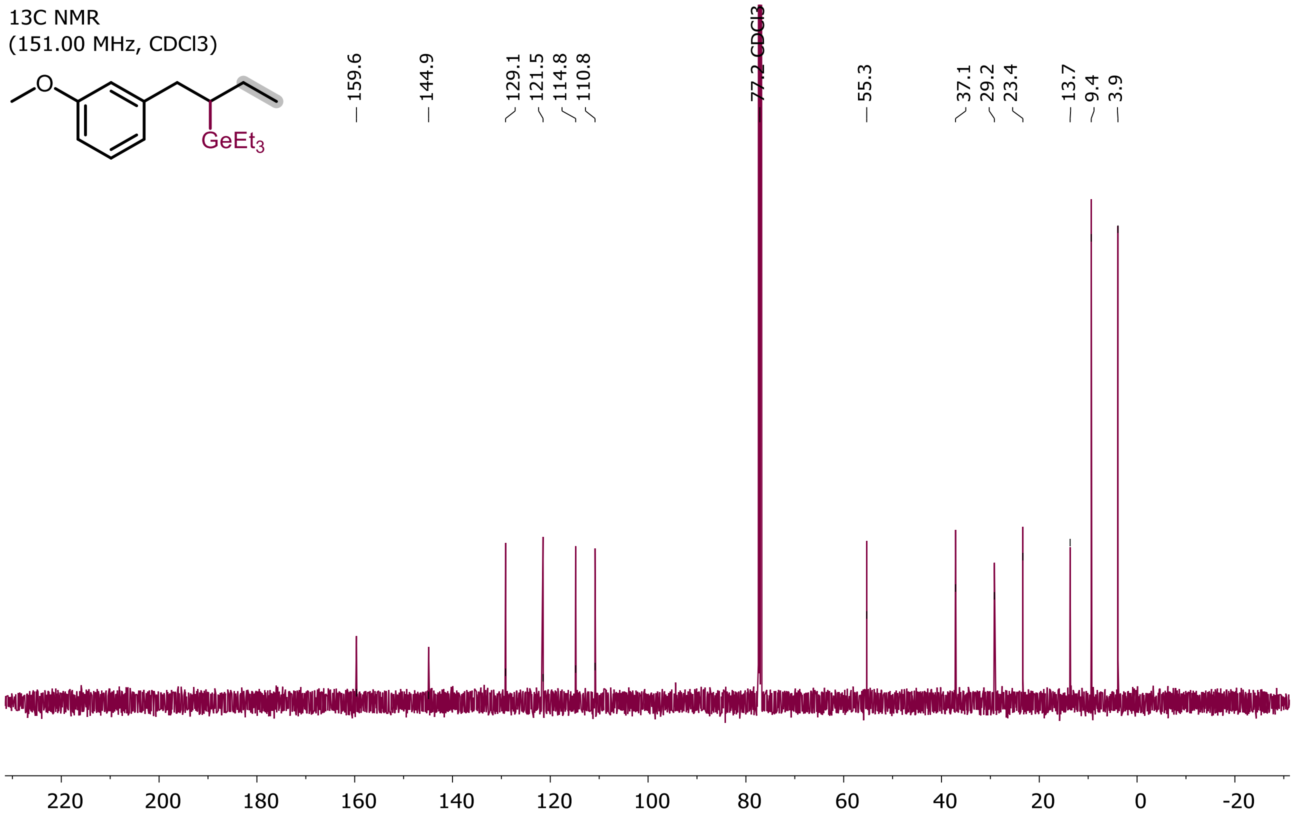

(1-(Benzo[*d*][1,3]dioxol-5-yl)butan-2-yl)triethylgermane (32)

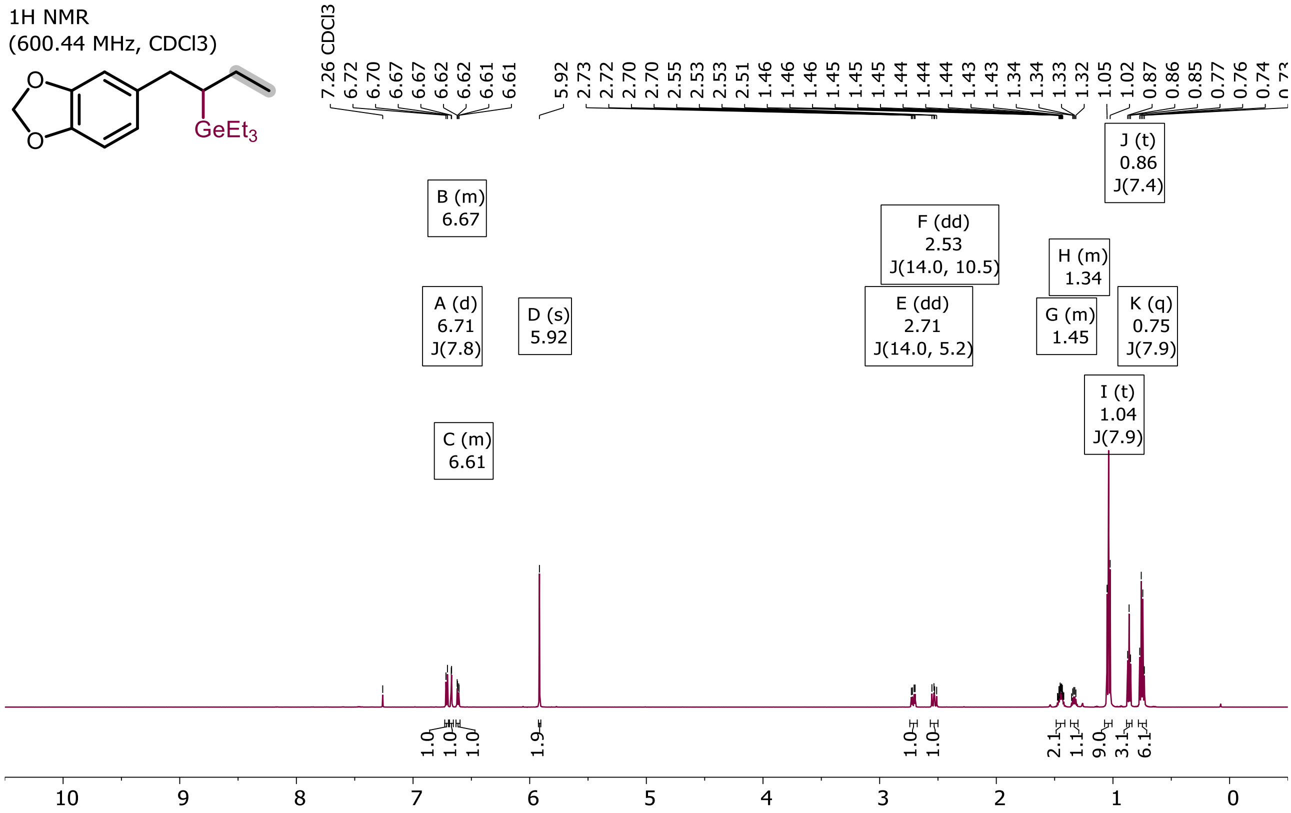

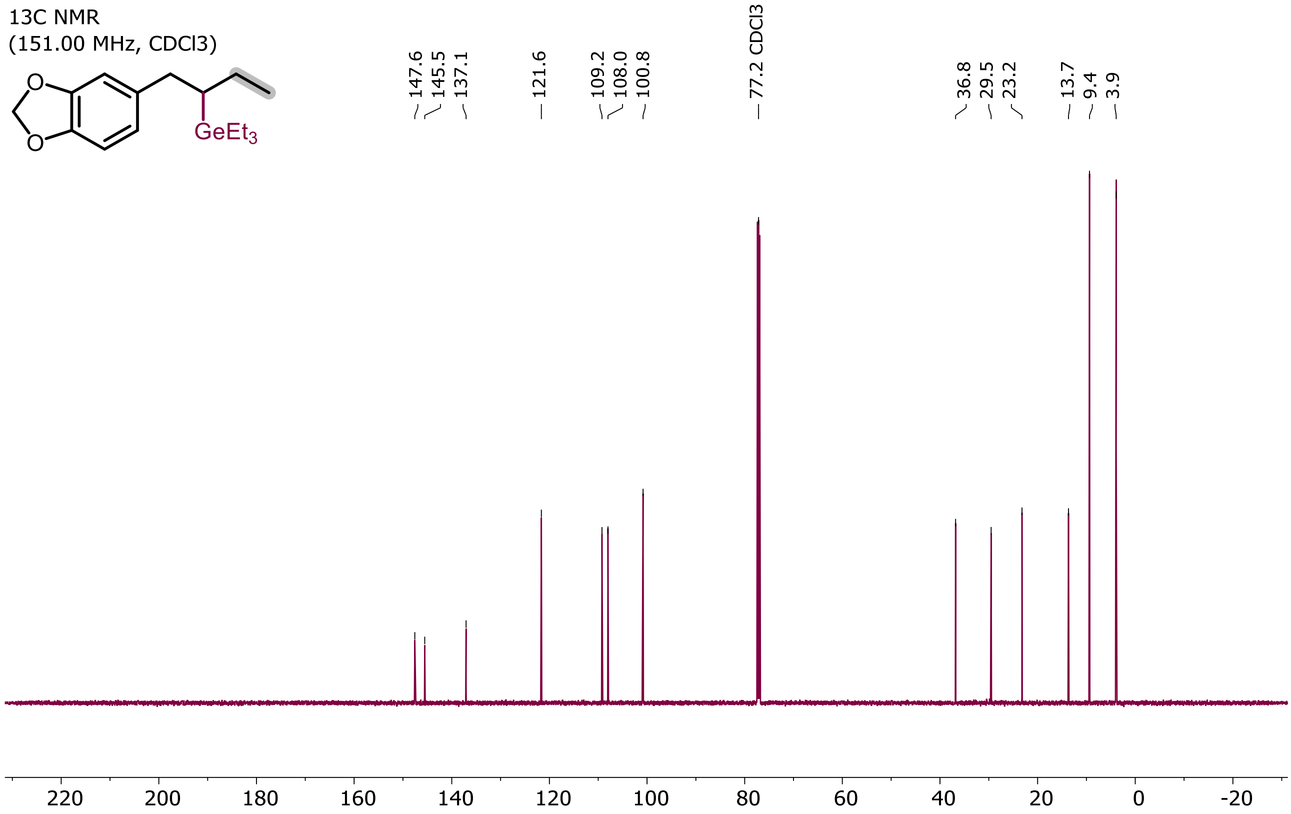

Triethyl(1-(4-(methylthio)phenyl)butan-2-yl)germane (33)

1H NMR
(600.44 MHz, CDCl3)

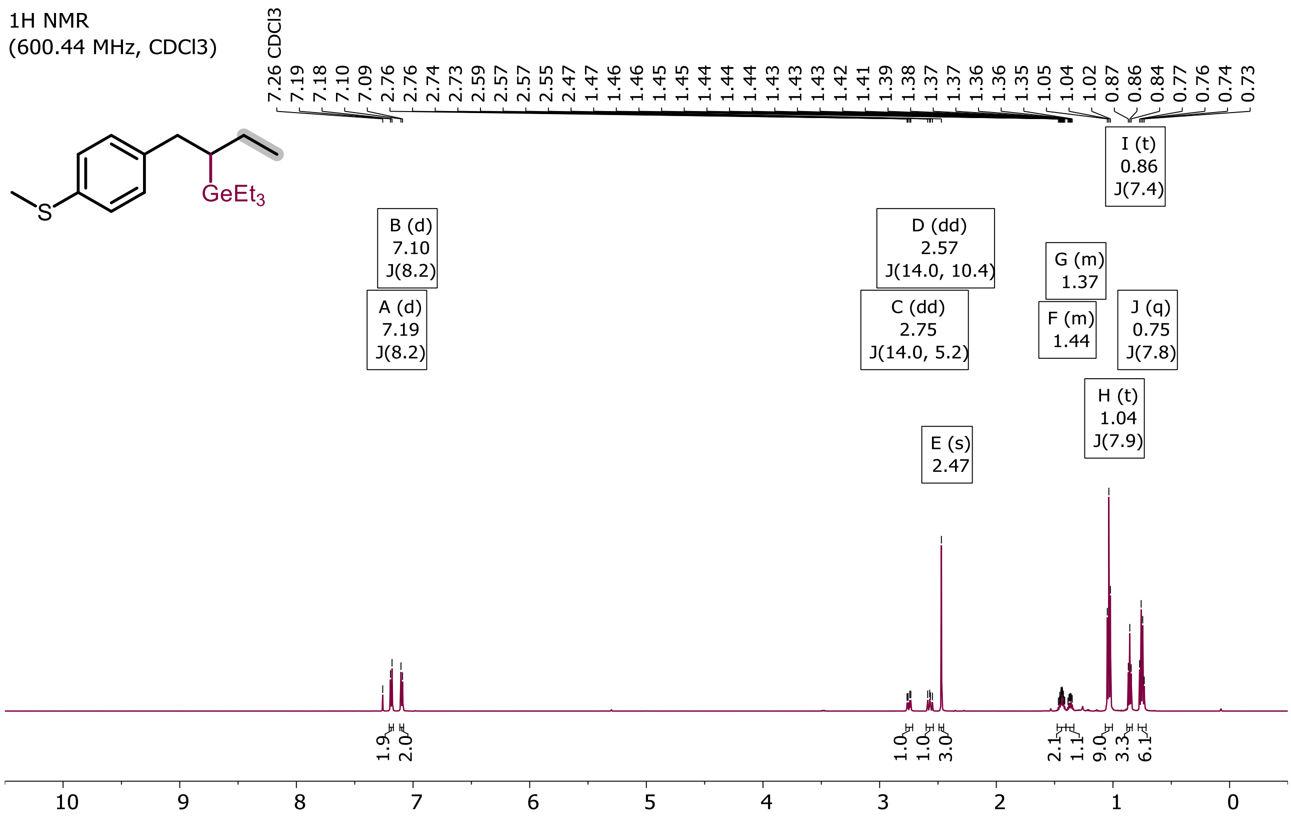

13C NMR
(151.00 MHz, CDCl3)

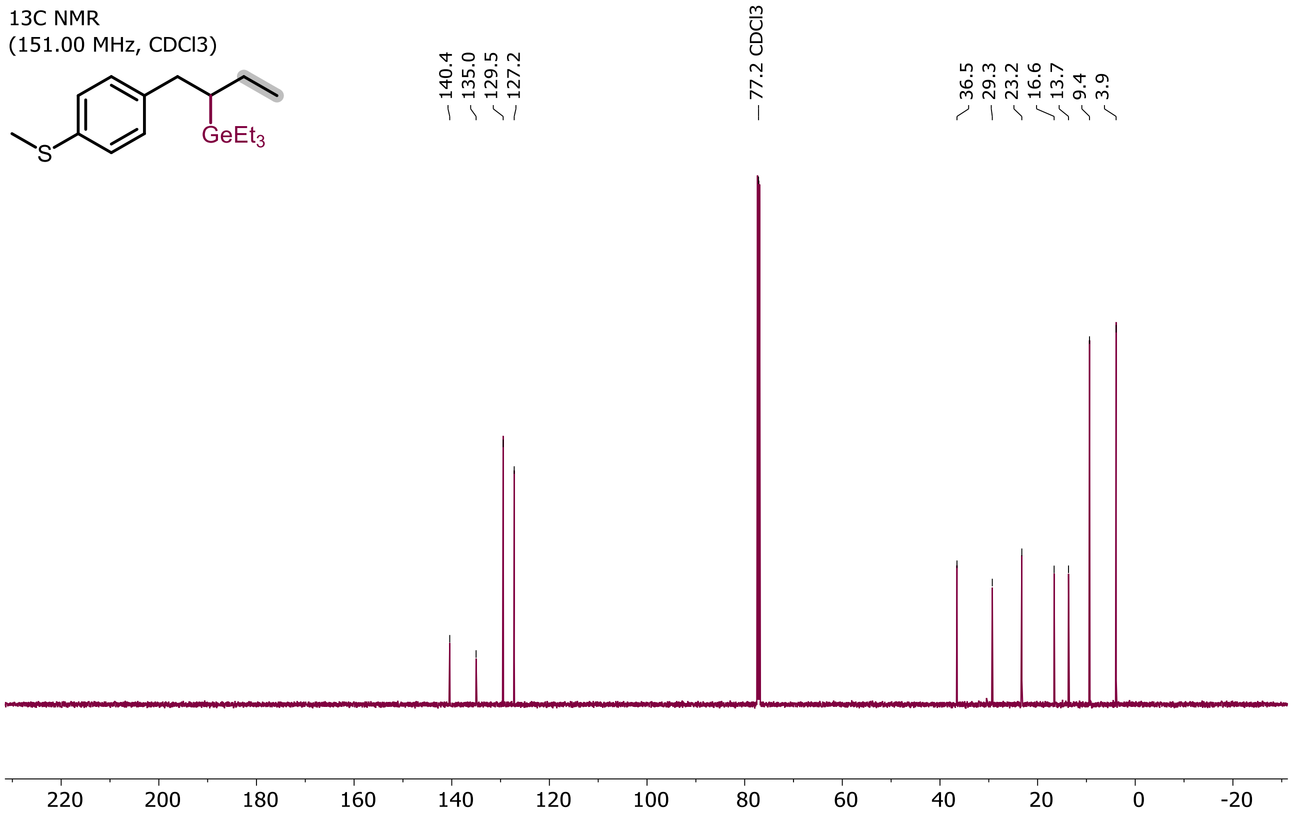

Triethyl(3-methyl-1-phenylbutan-2-yl)germane (34)

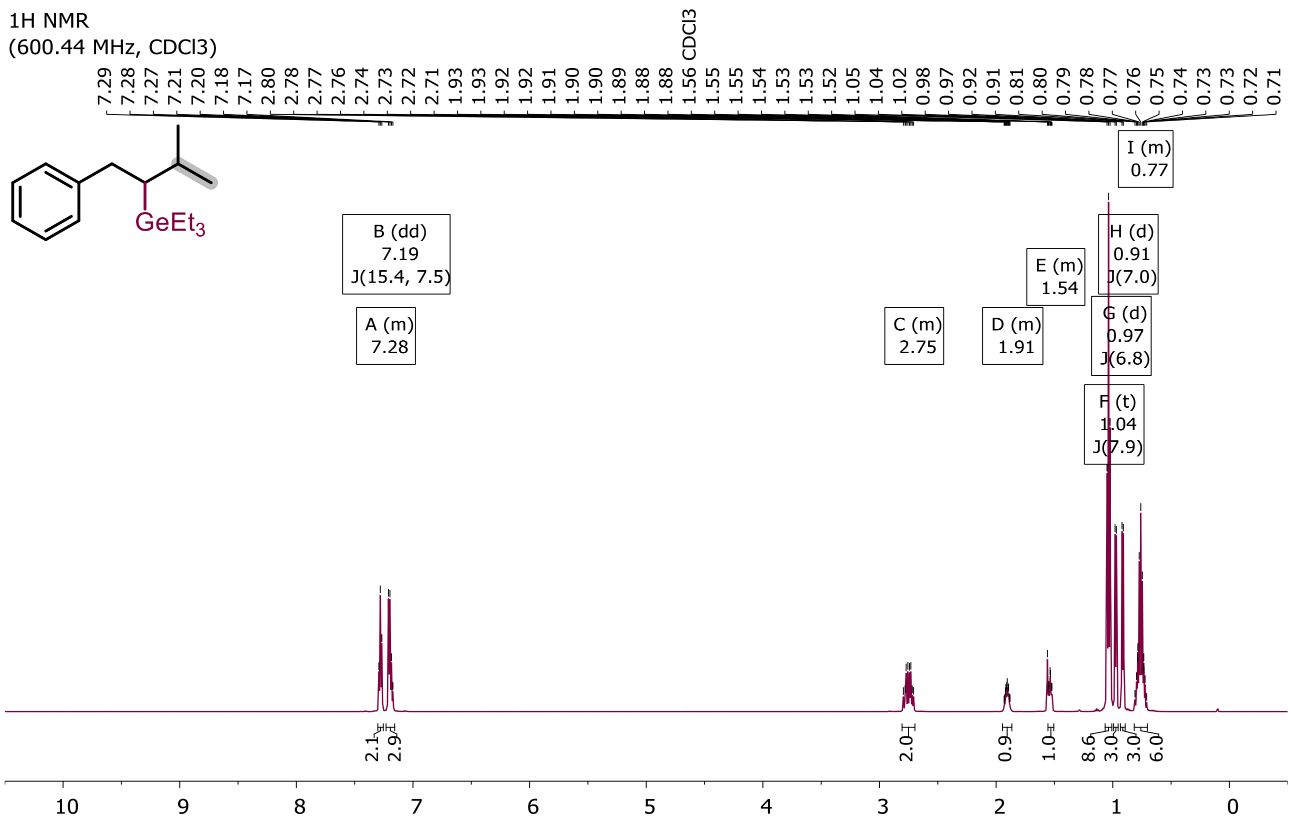

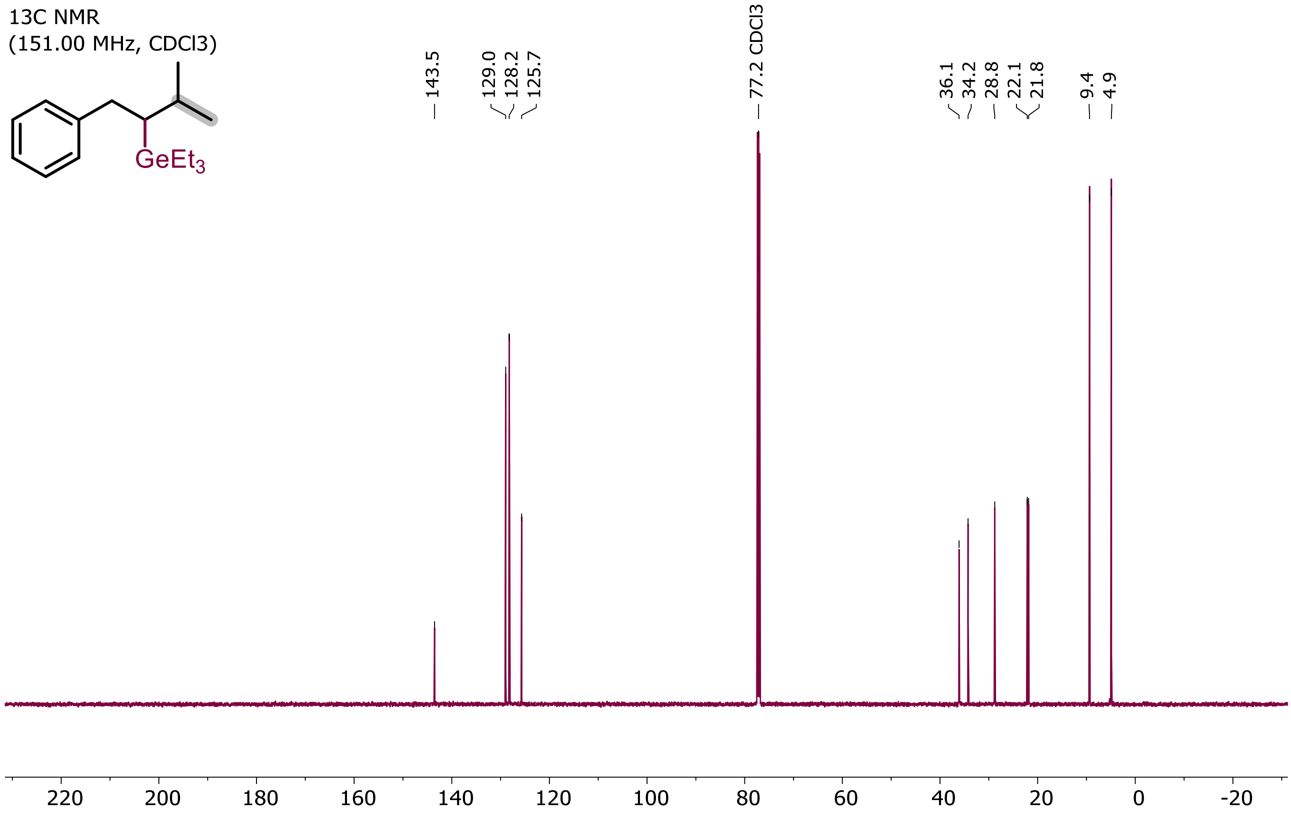

Triethyl(1-(6-methoxynaphthalen-2-yl)-3-methylbutan-2-yl)germane (35)

1H NMR
(600.44 MHz, CDCl3)

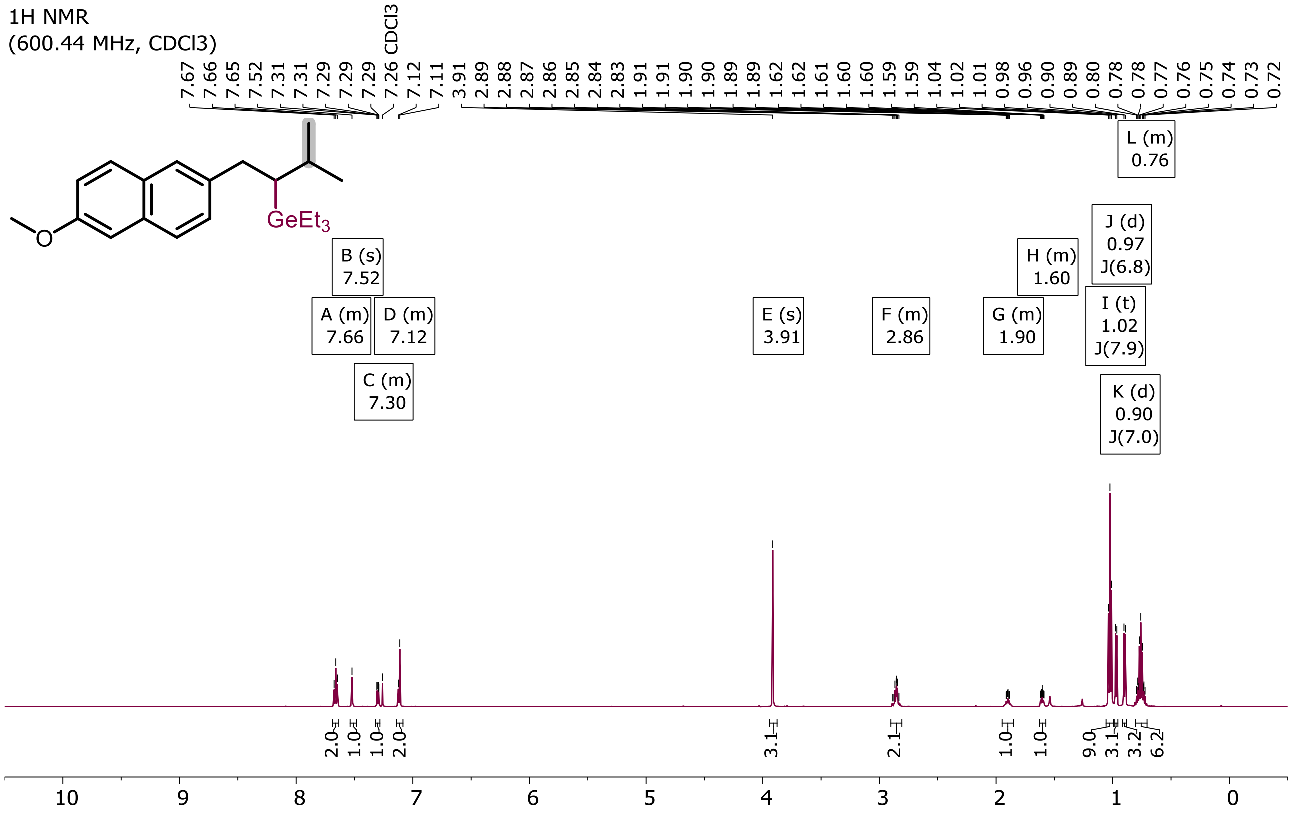

13C NMR
(151.00 MHz, CDCl3)

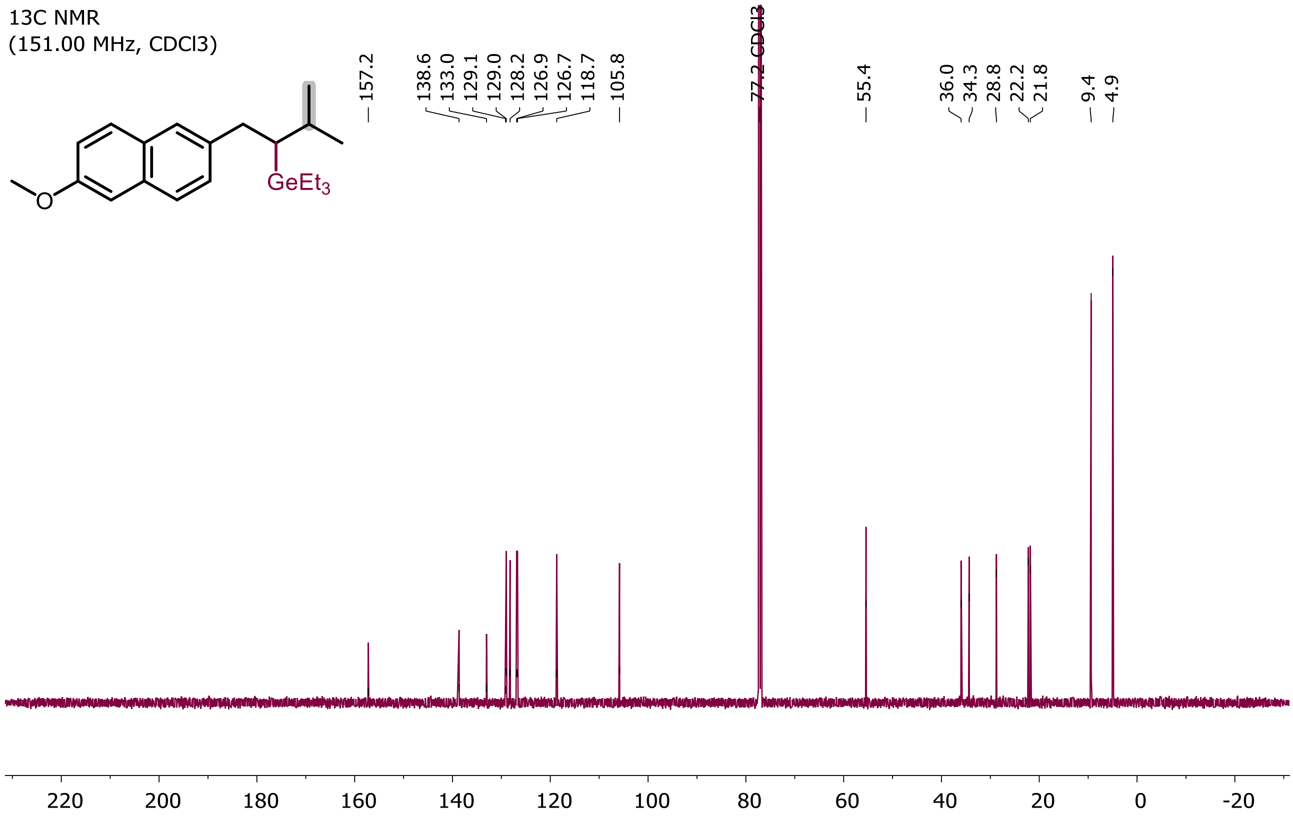

Triethyl(2-phenylpentyl)germane (37)

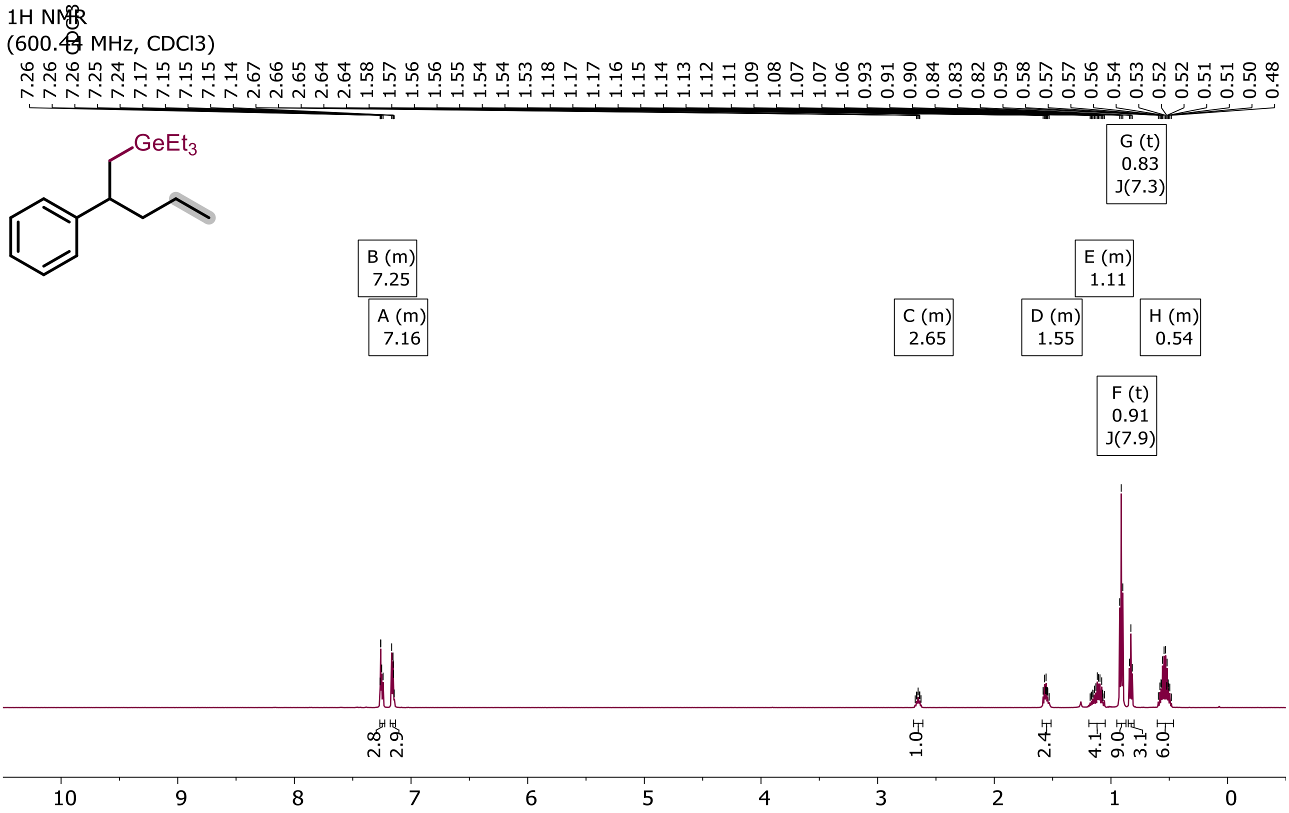

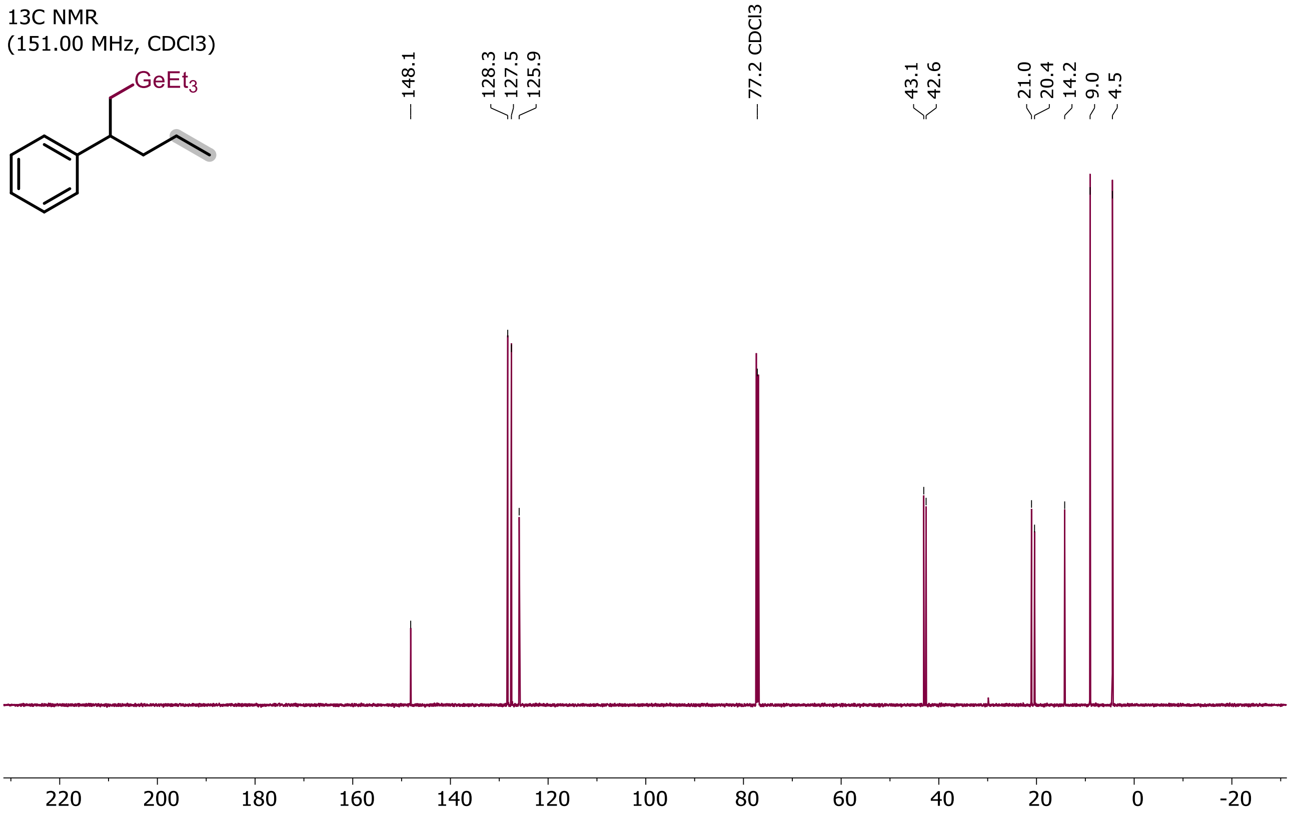

2-(2-(Triethylgermyl)butyl)pyridine (38)

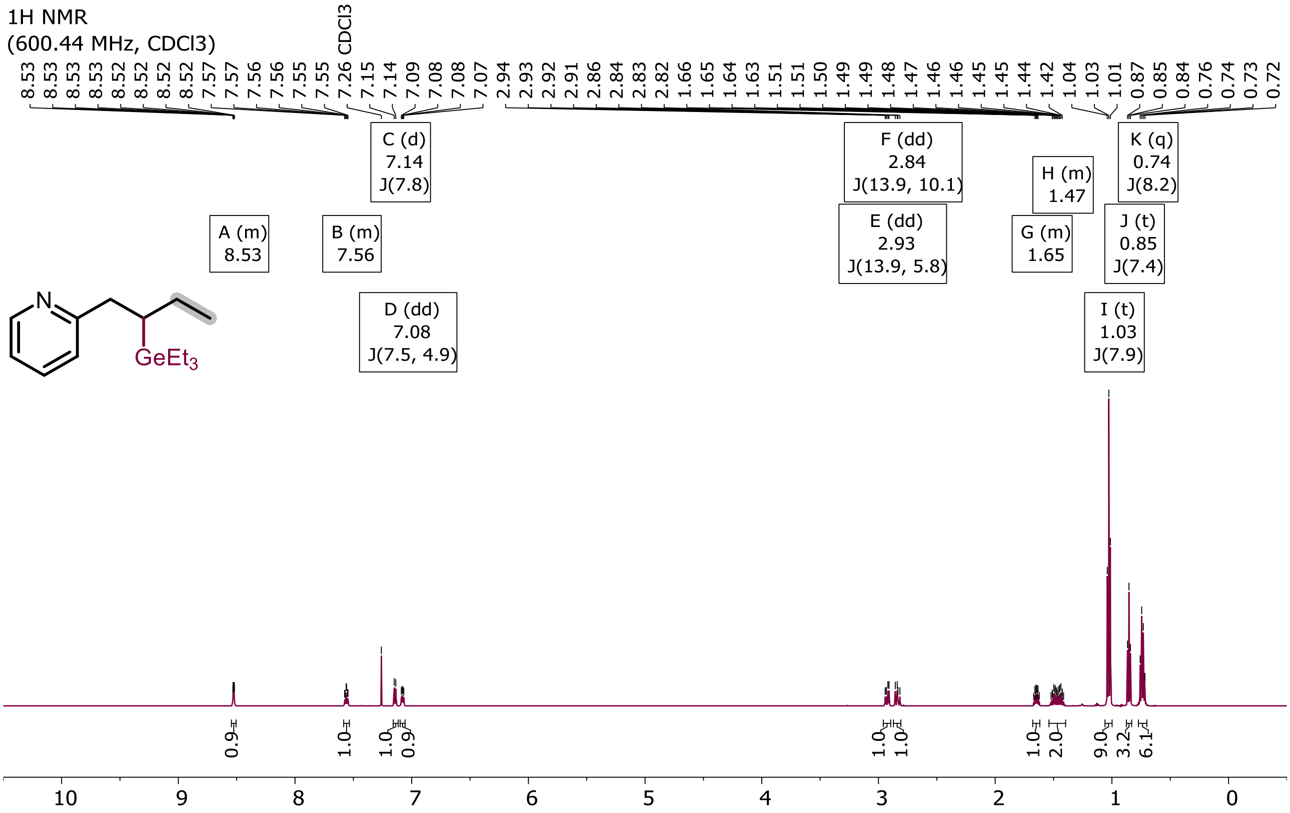

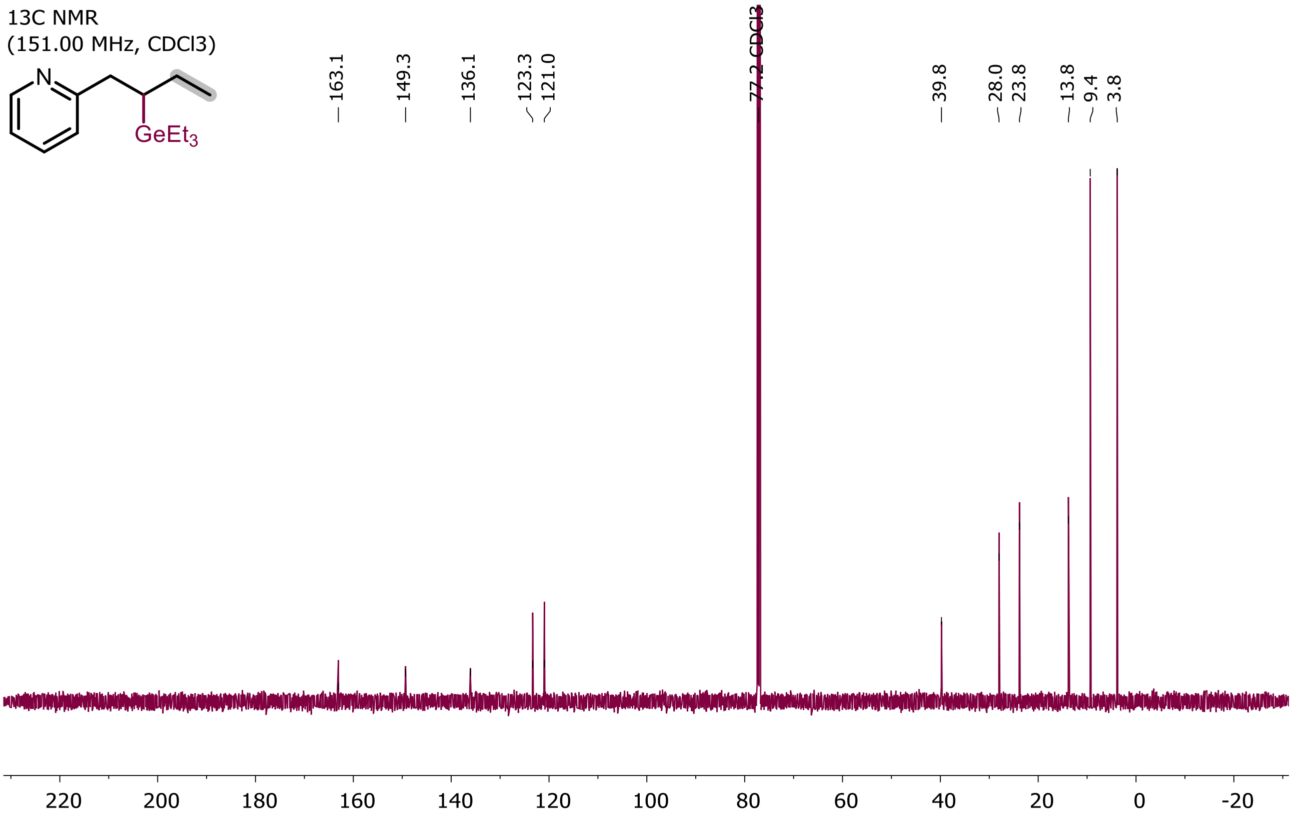

8-(2-(Triethylgermyl)butyl)quinoline (39)

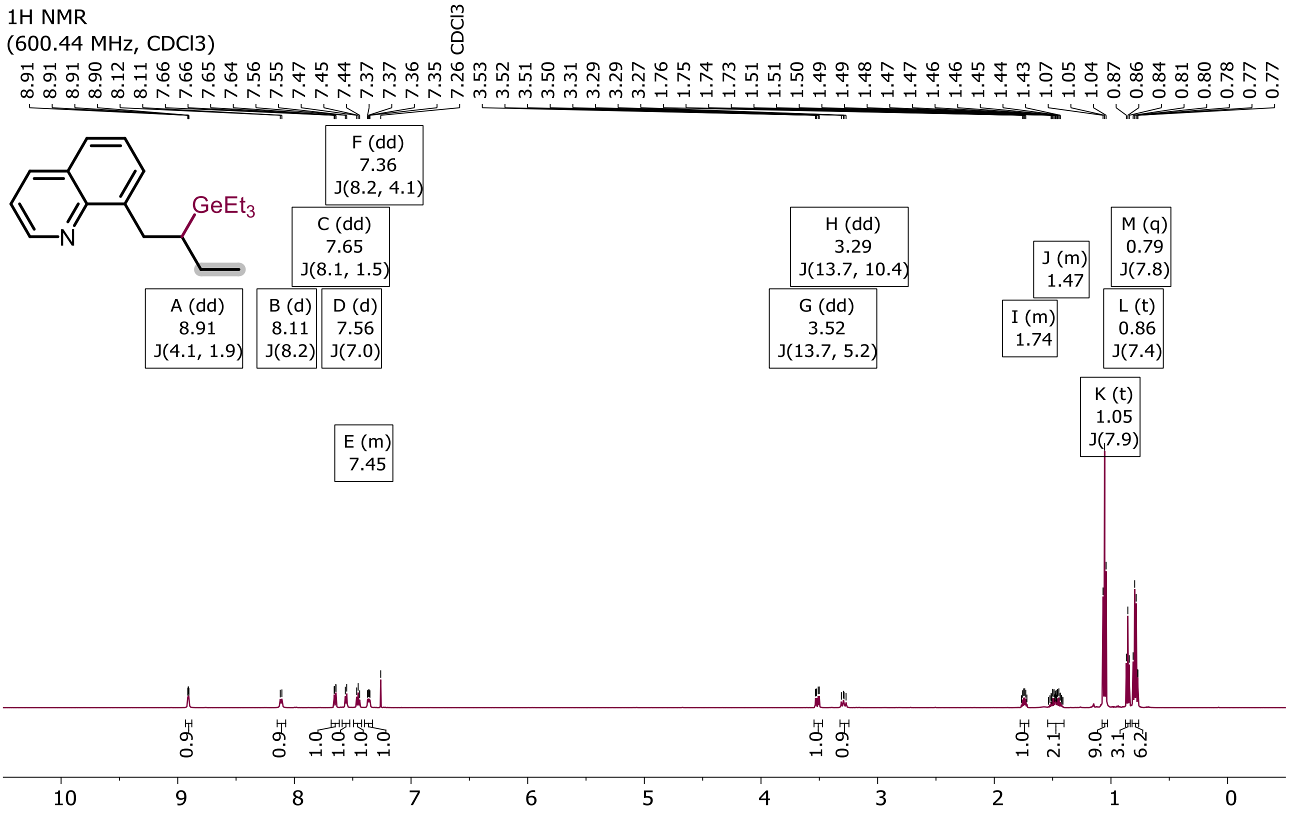

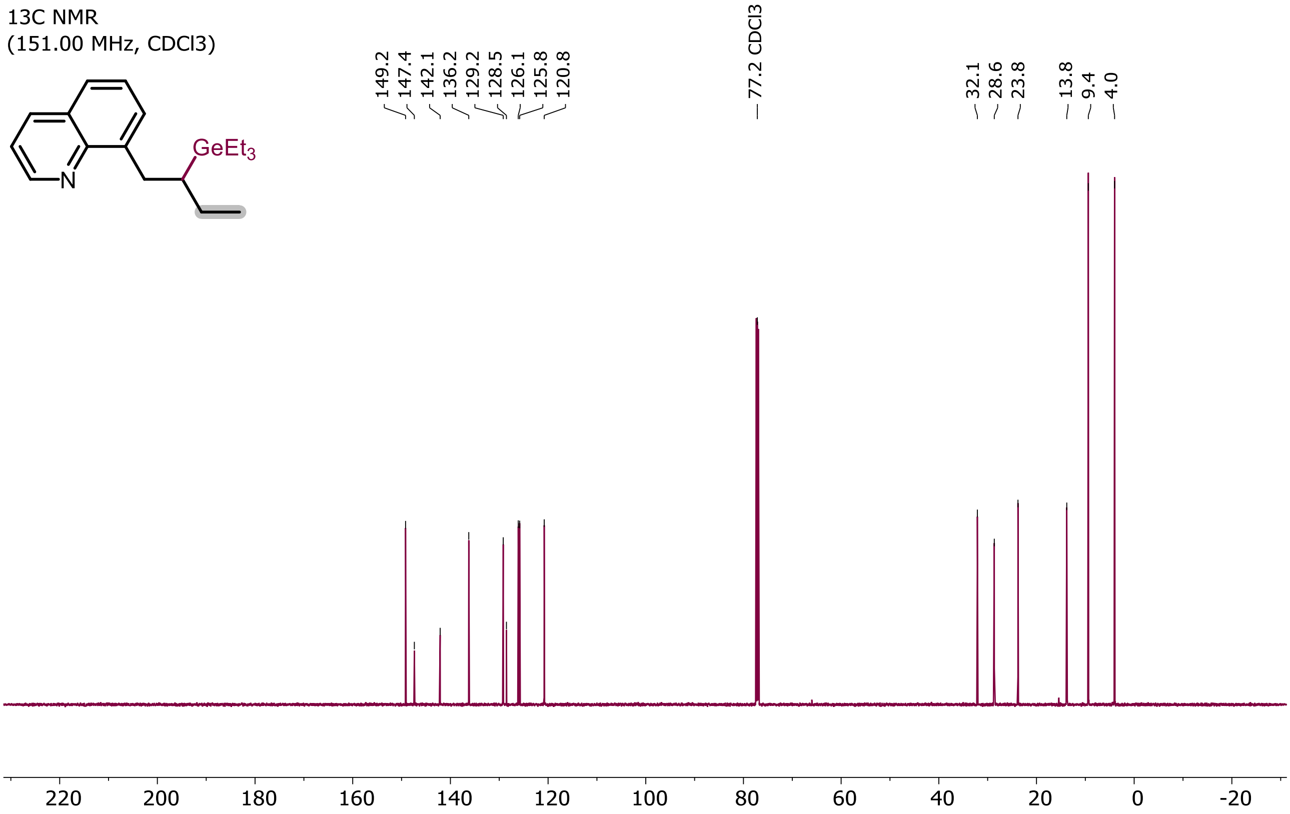

Triethyl(1-phenylpentan-2-yl)germane (40)

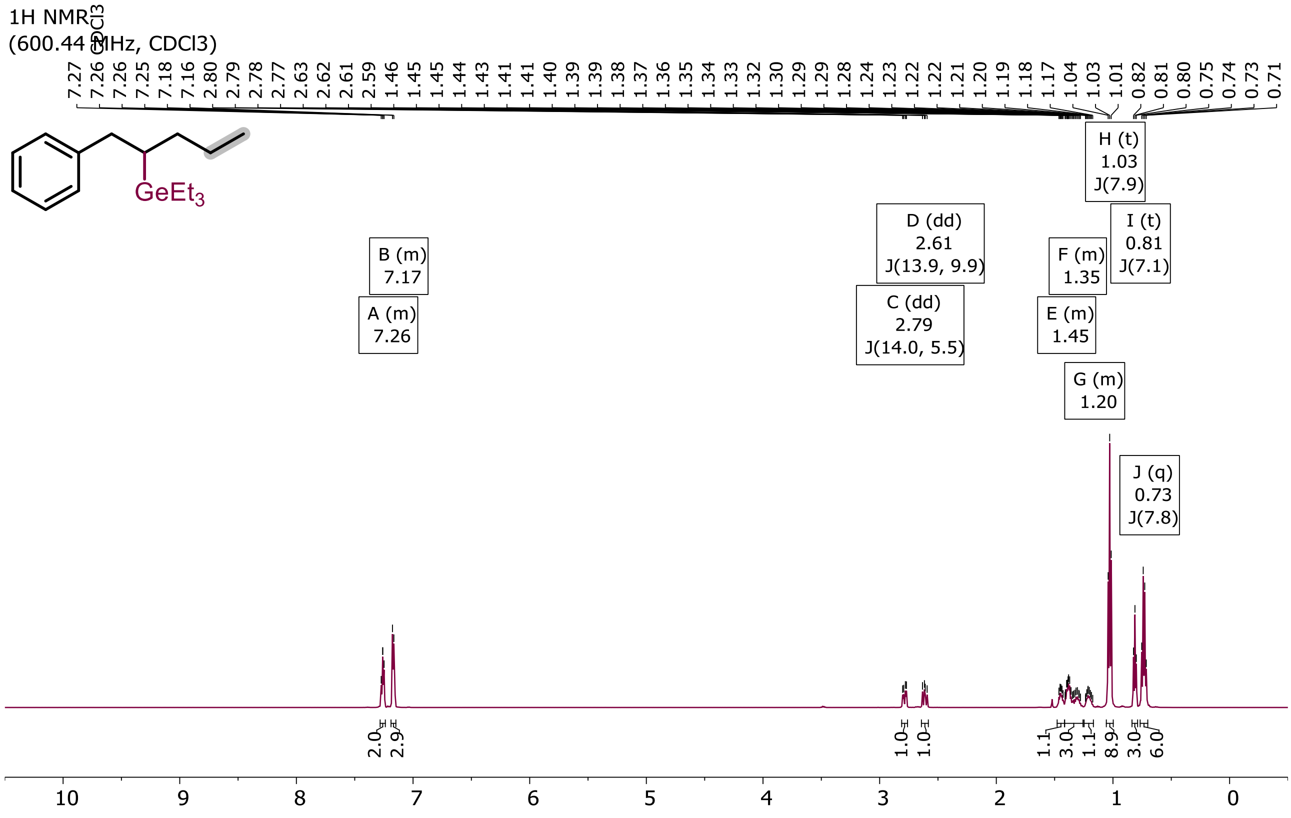

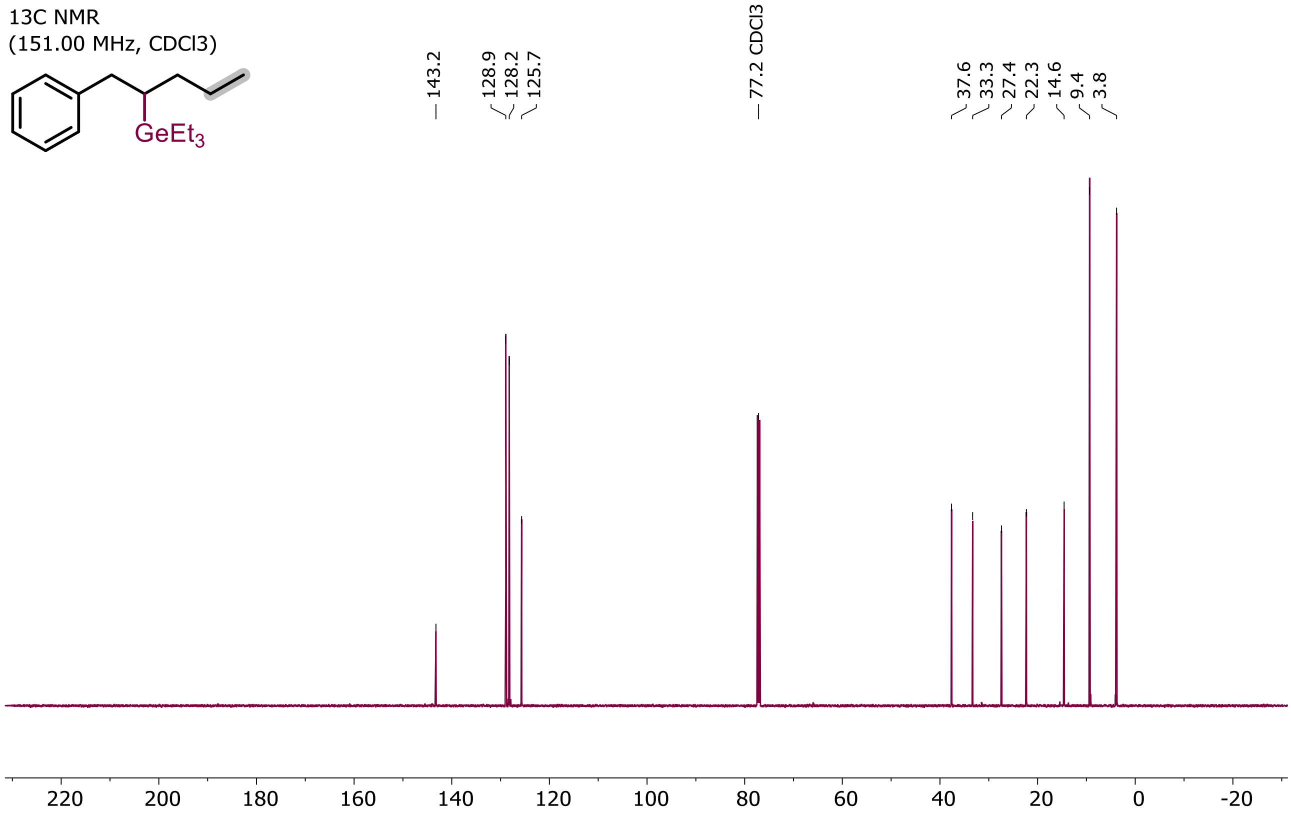

Triethyl(1-phenylnonan-2-yl)germane (41)

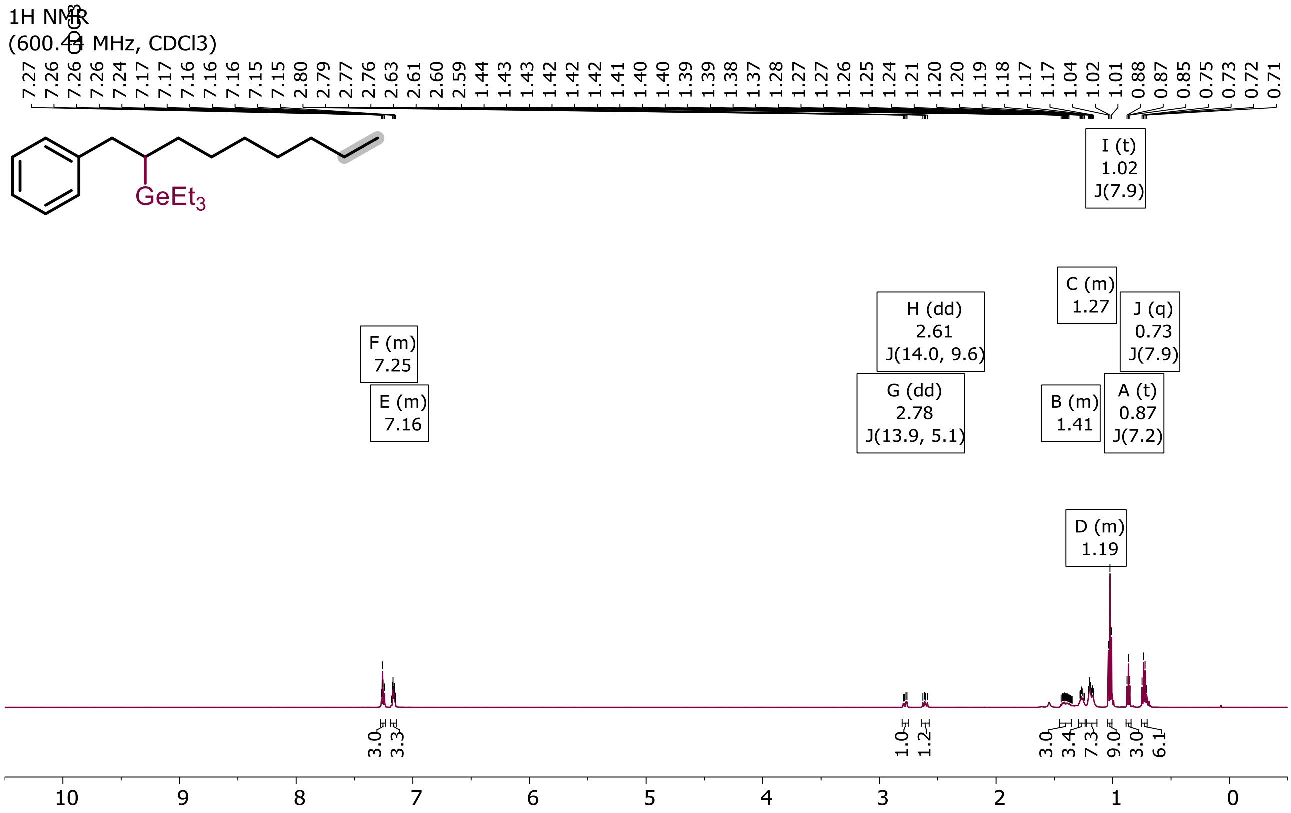

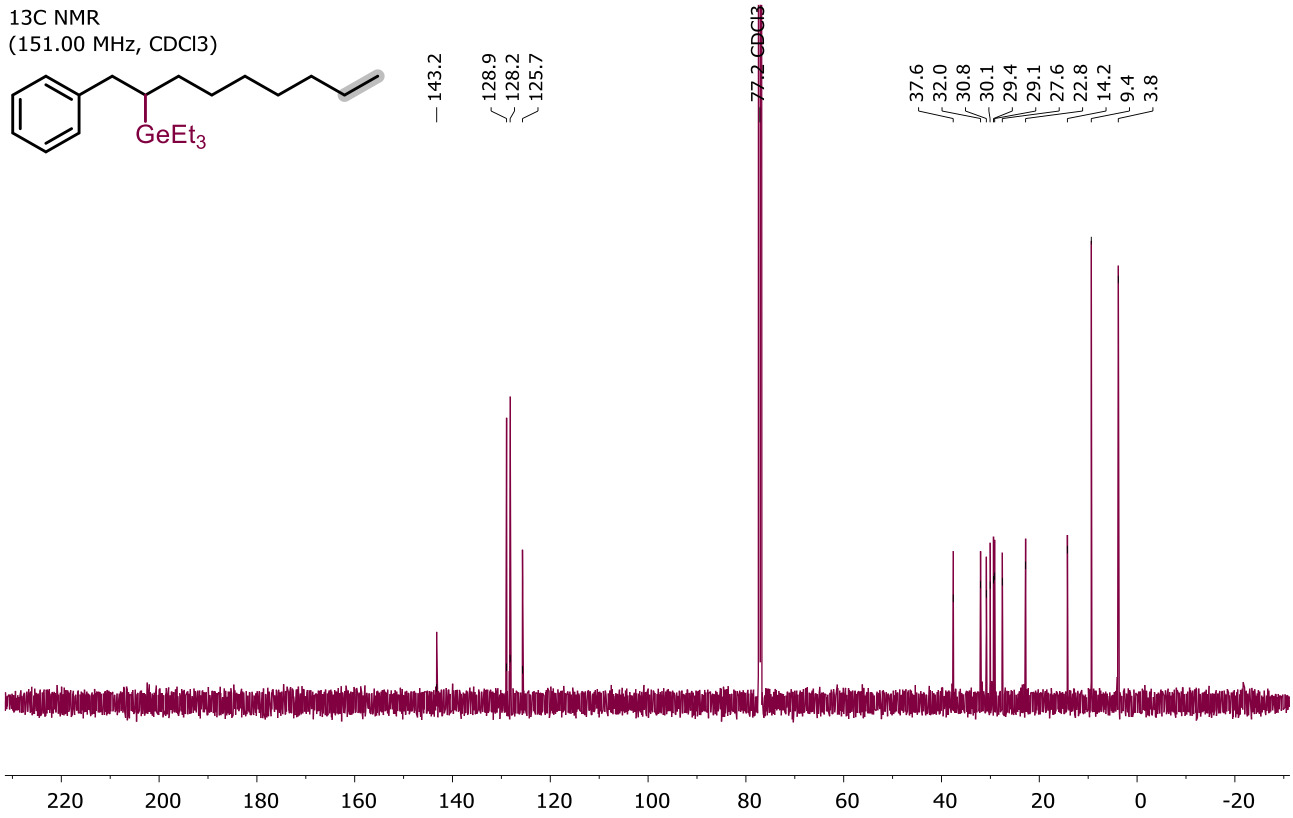

Triethyl(1-phenyldecan-2-yl)germane (42)

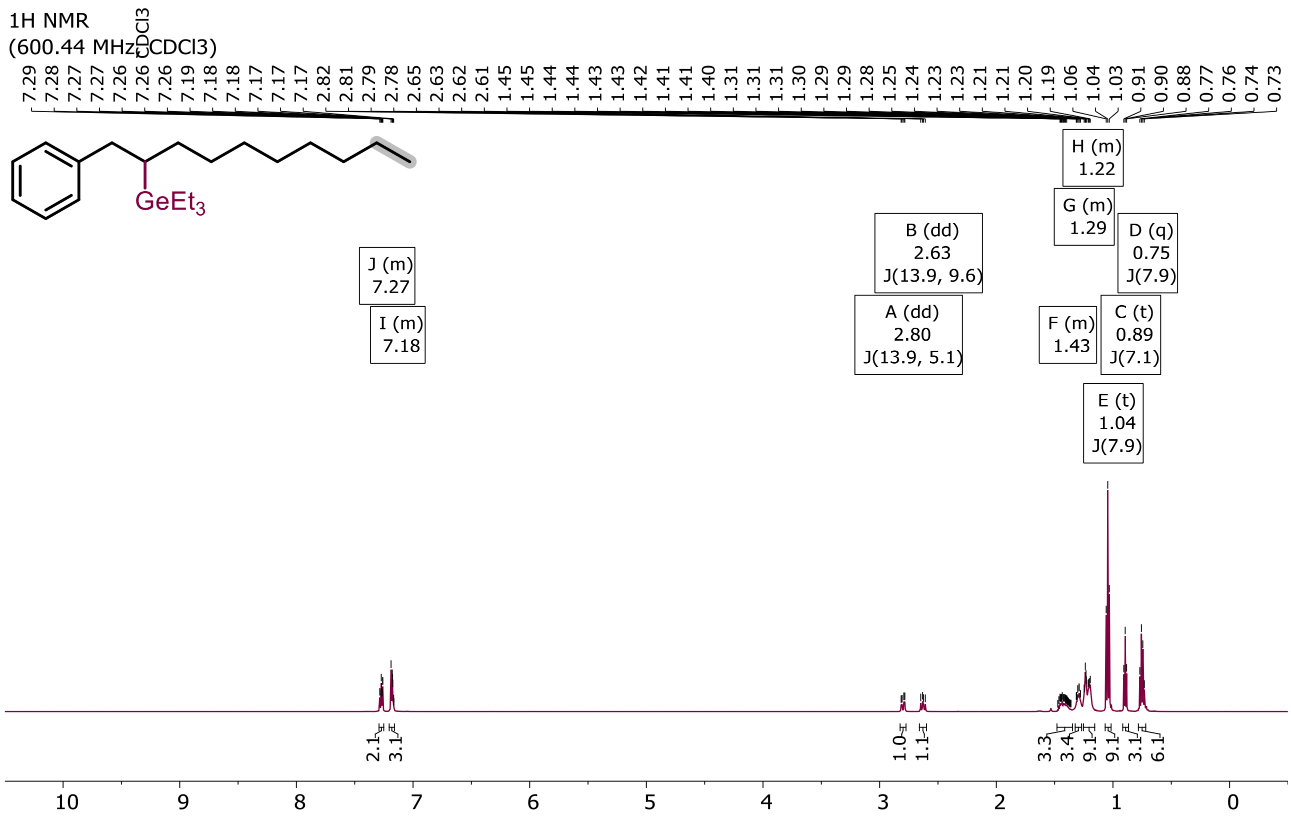

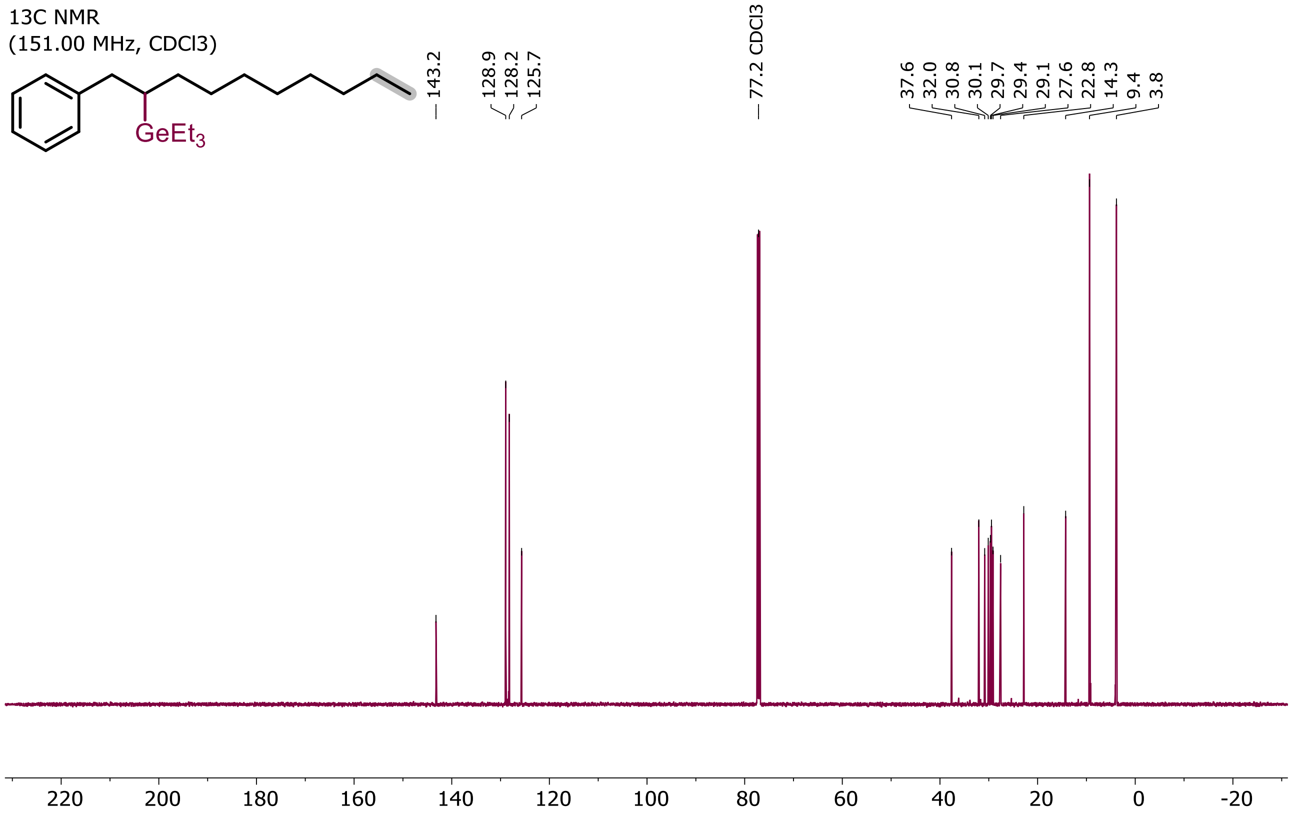

(*E*)-but-1-en-1-ylbenzene (43)

1H NMR
(600.44 MHz, CDCl3)

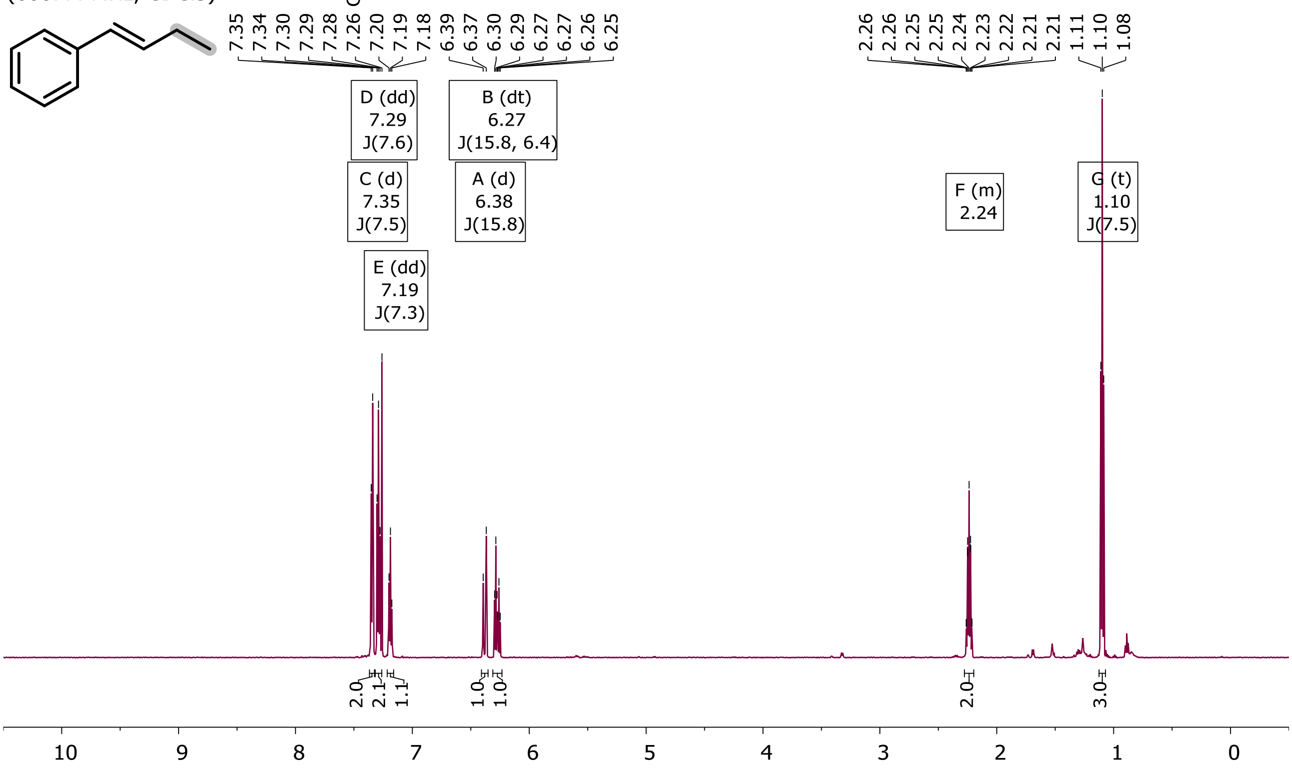

13C NMR
(151.00 MHz, CDCl3)

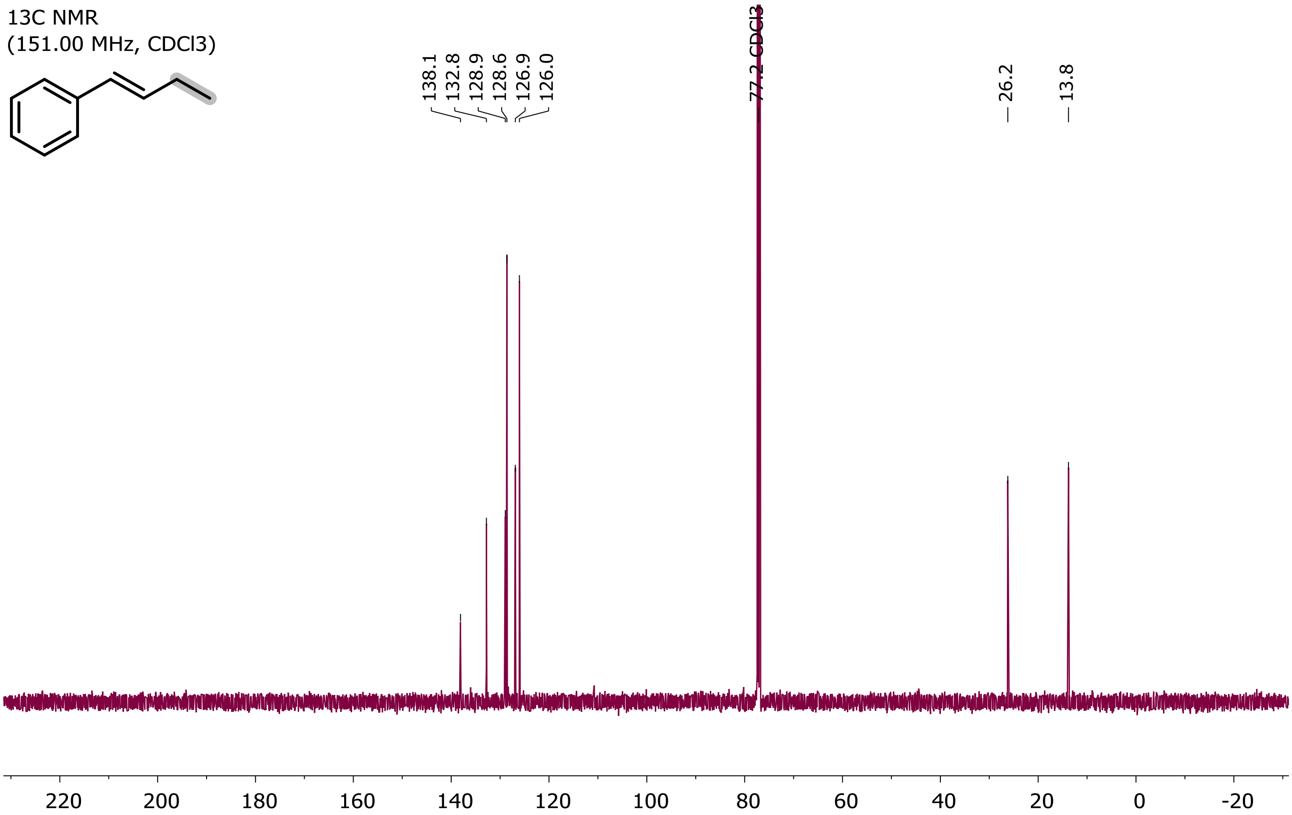

(2-Cyclopropyl-2-phenylethyl)triethylgermane (45)

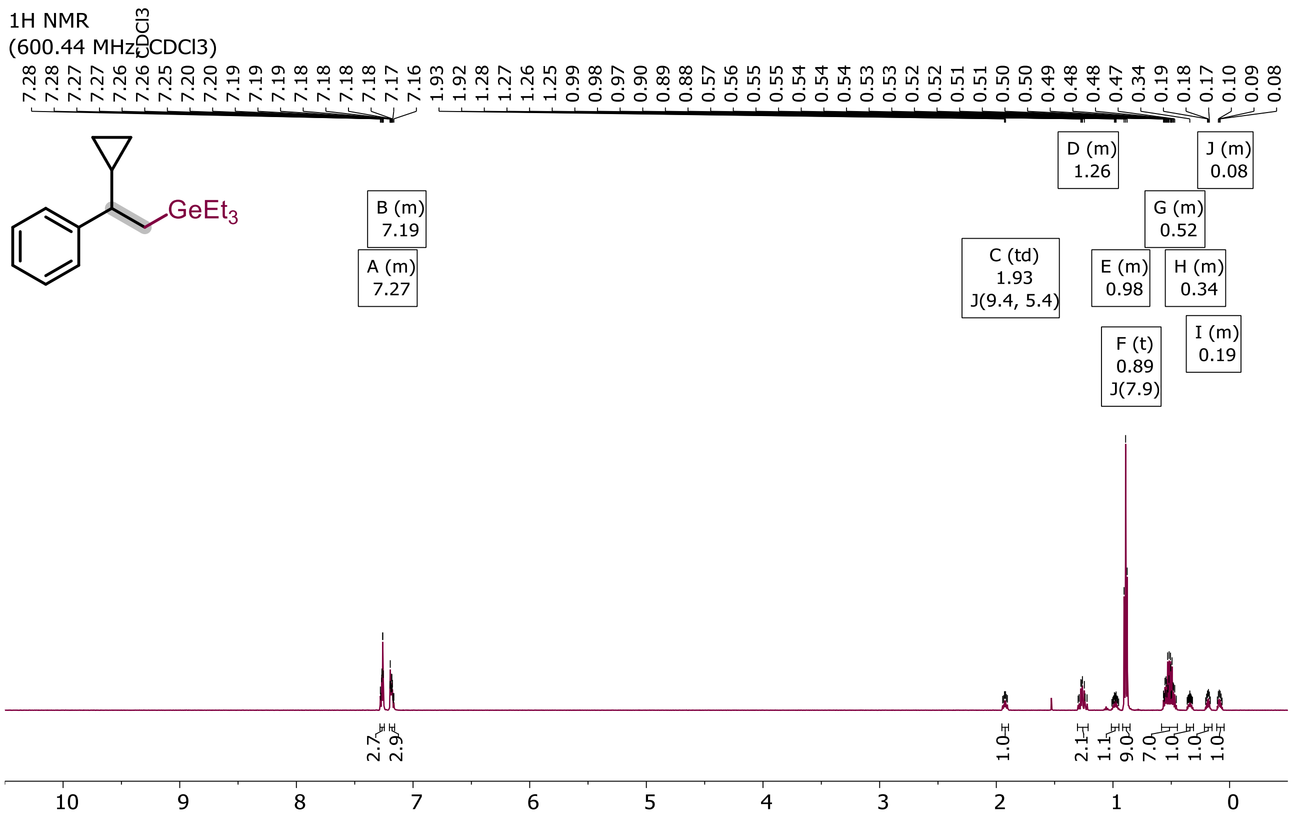

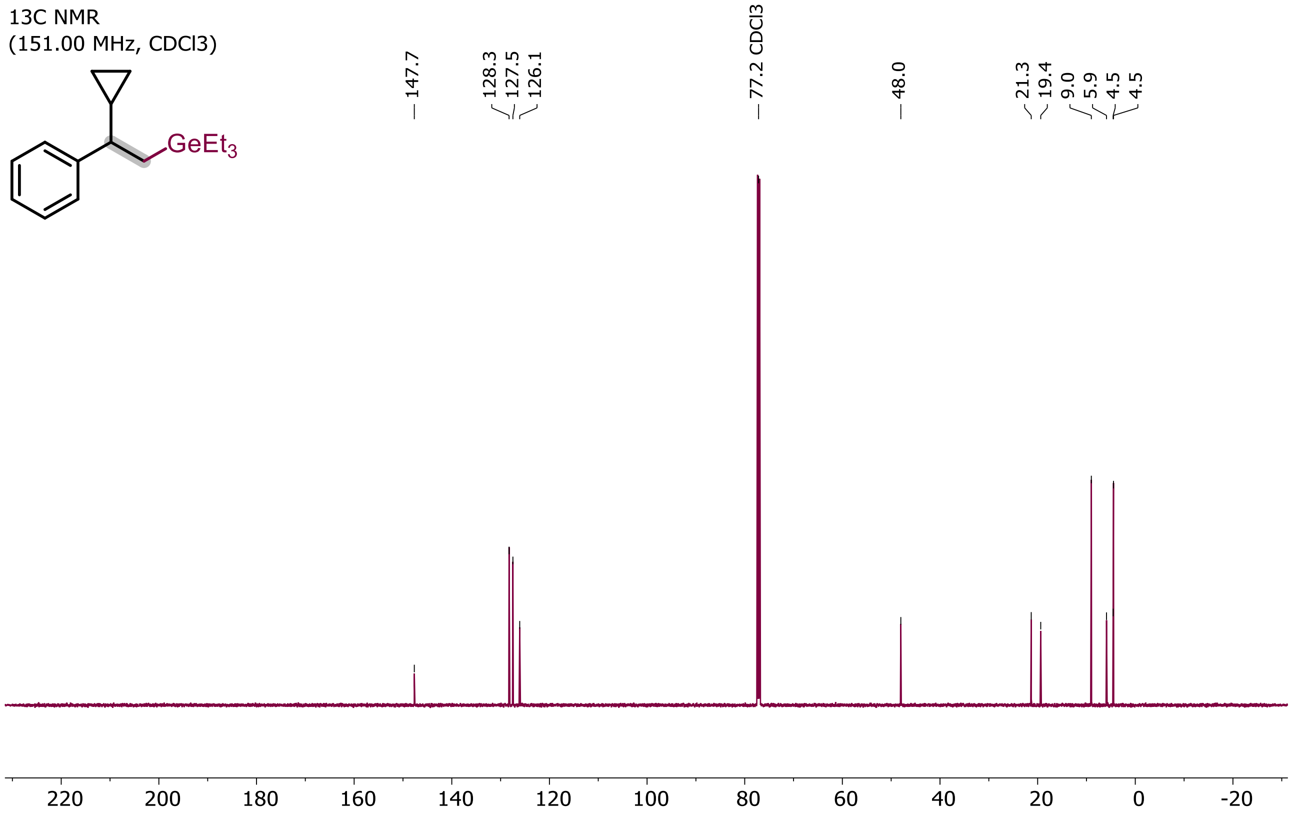

7.2 Starting materials

2-Allylbenzo[b]thiophene (S16)

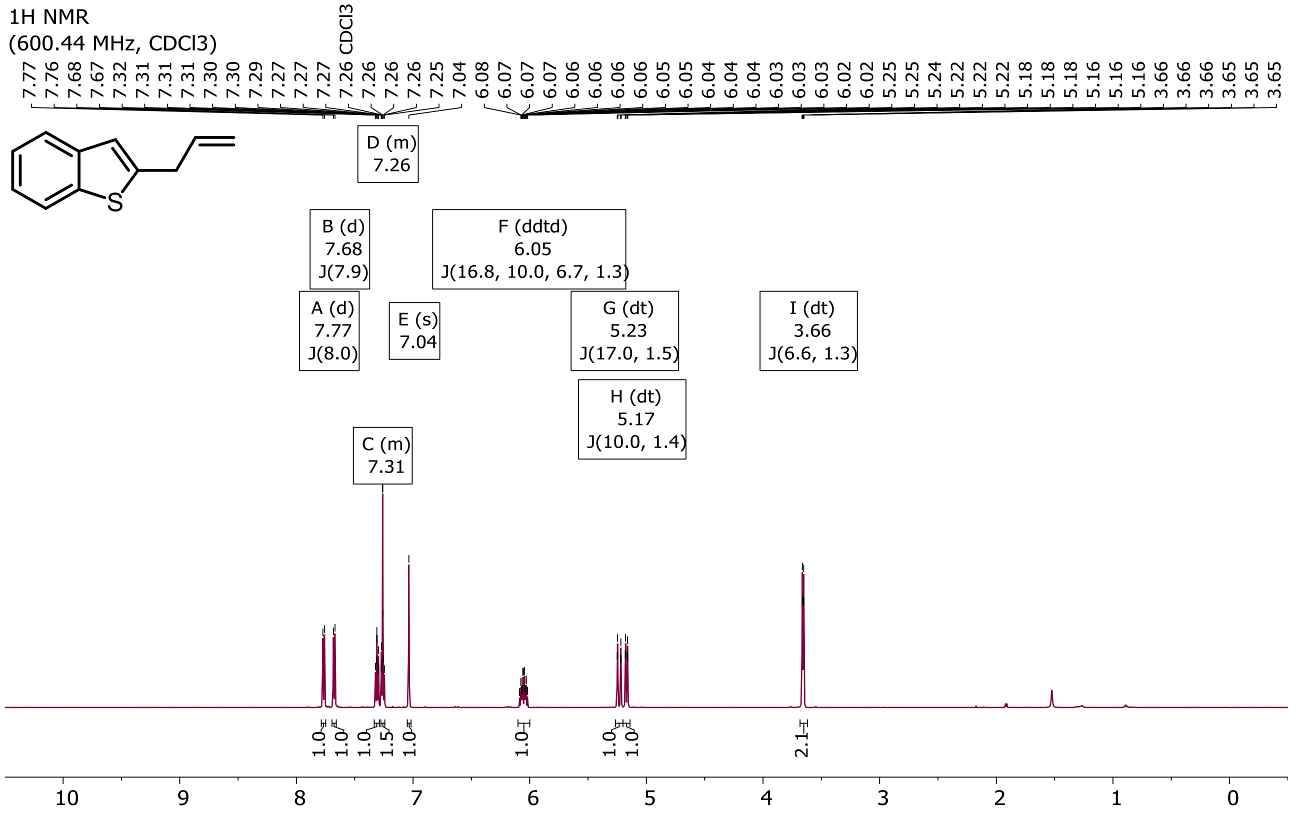

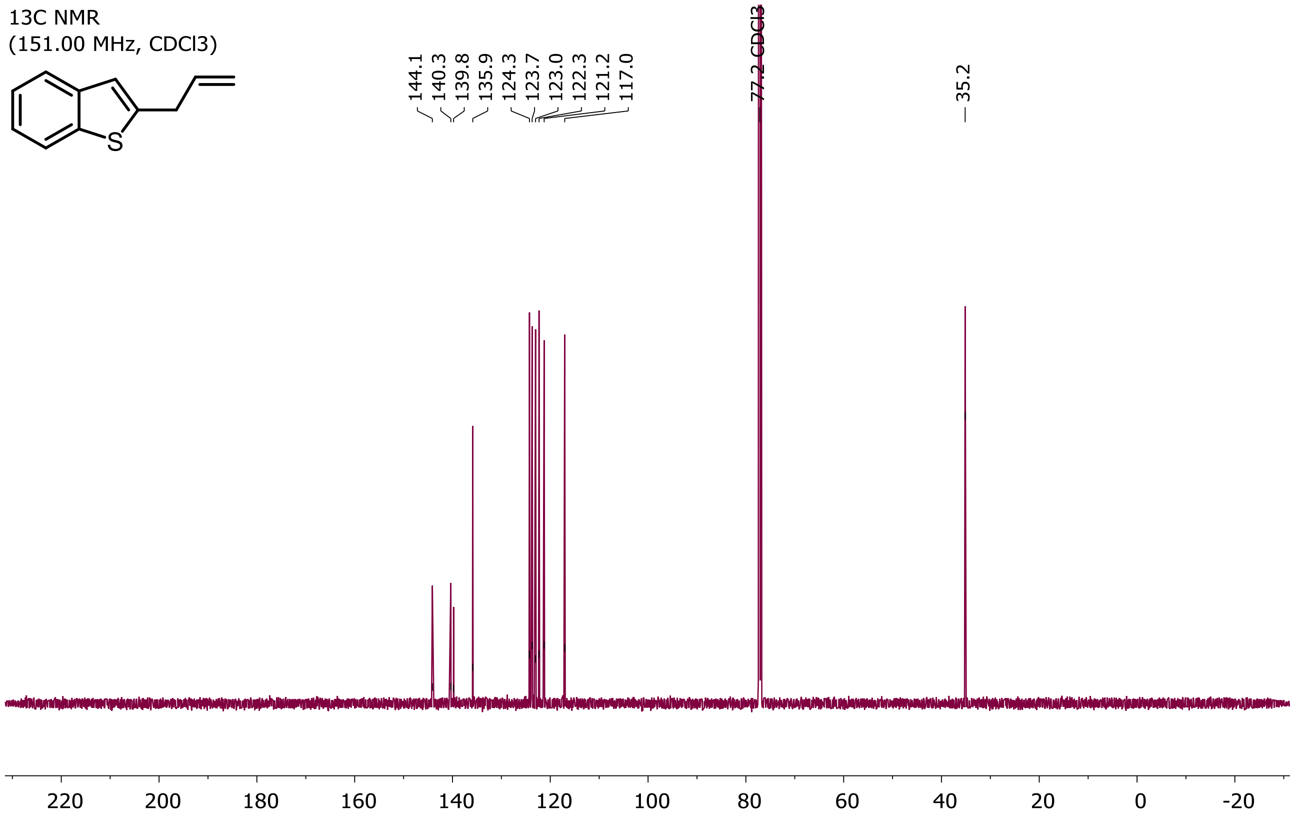

3-(But-3-en-1-yl)-1,1'-biphenyl (S28)

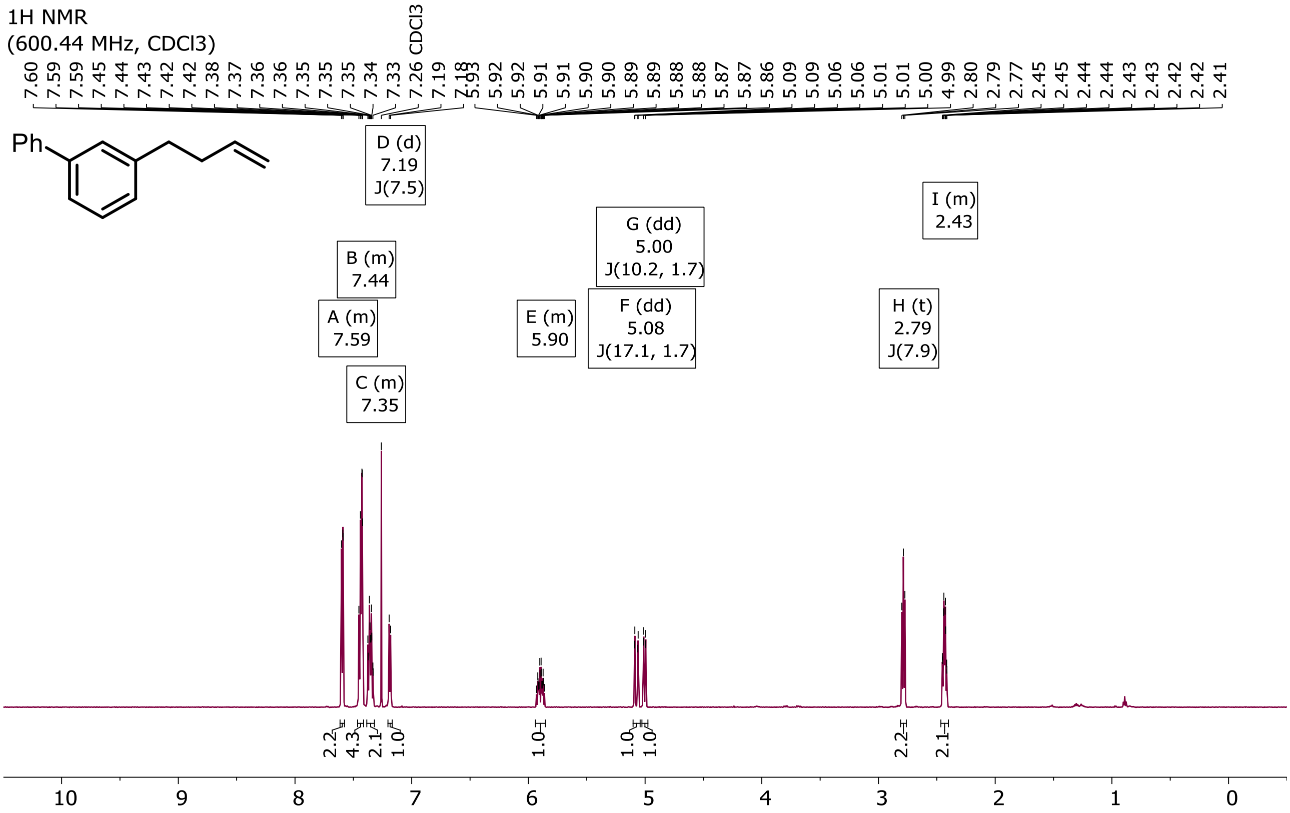

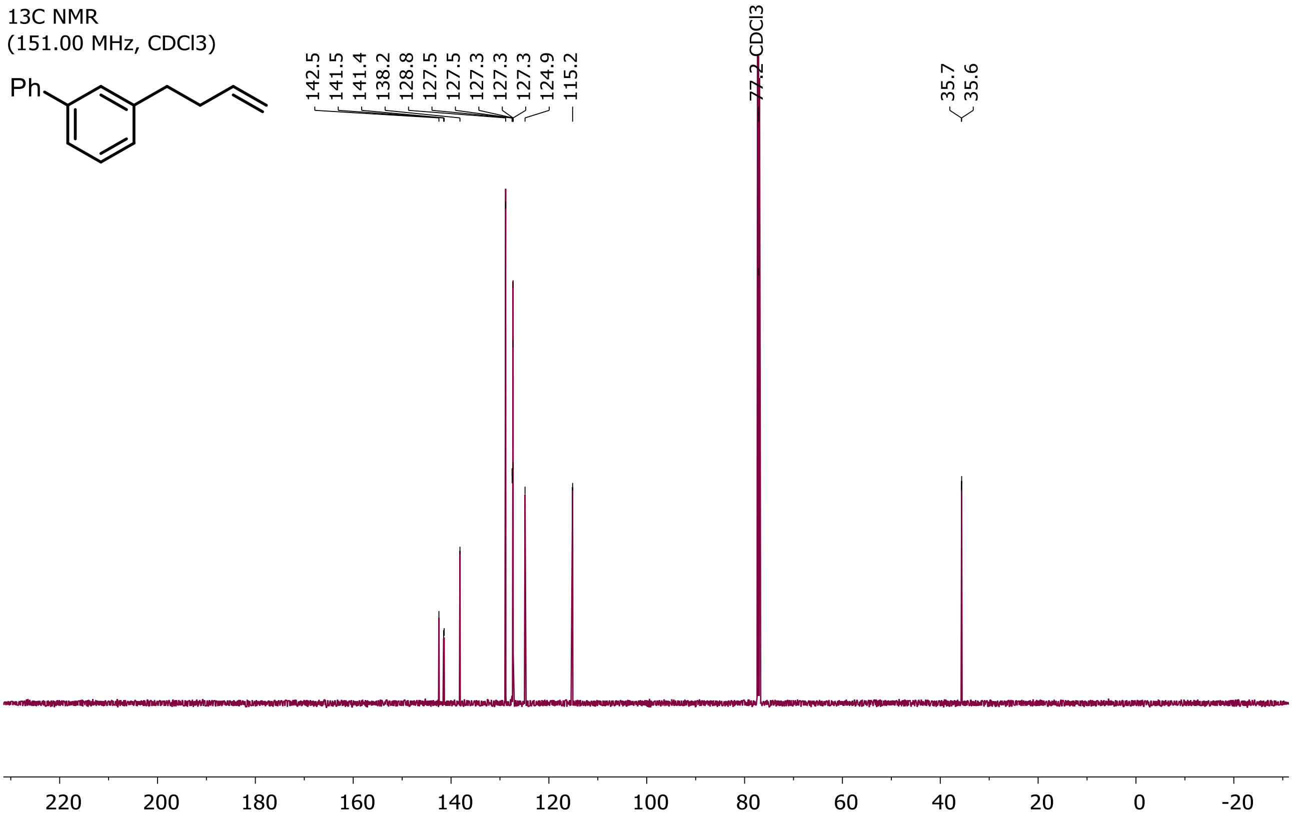

5-(But-3-en-1-yl)benzo[*d*][1,3]dioxole (*S*32)

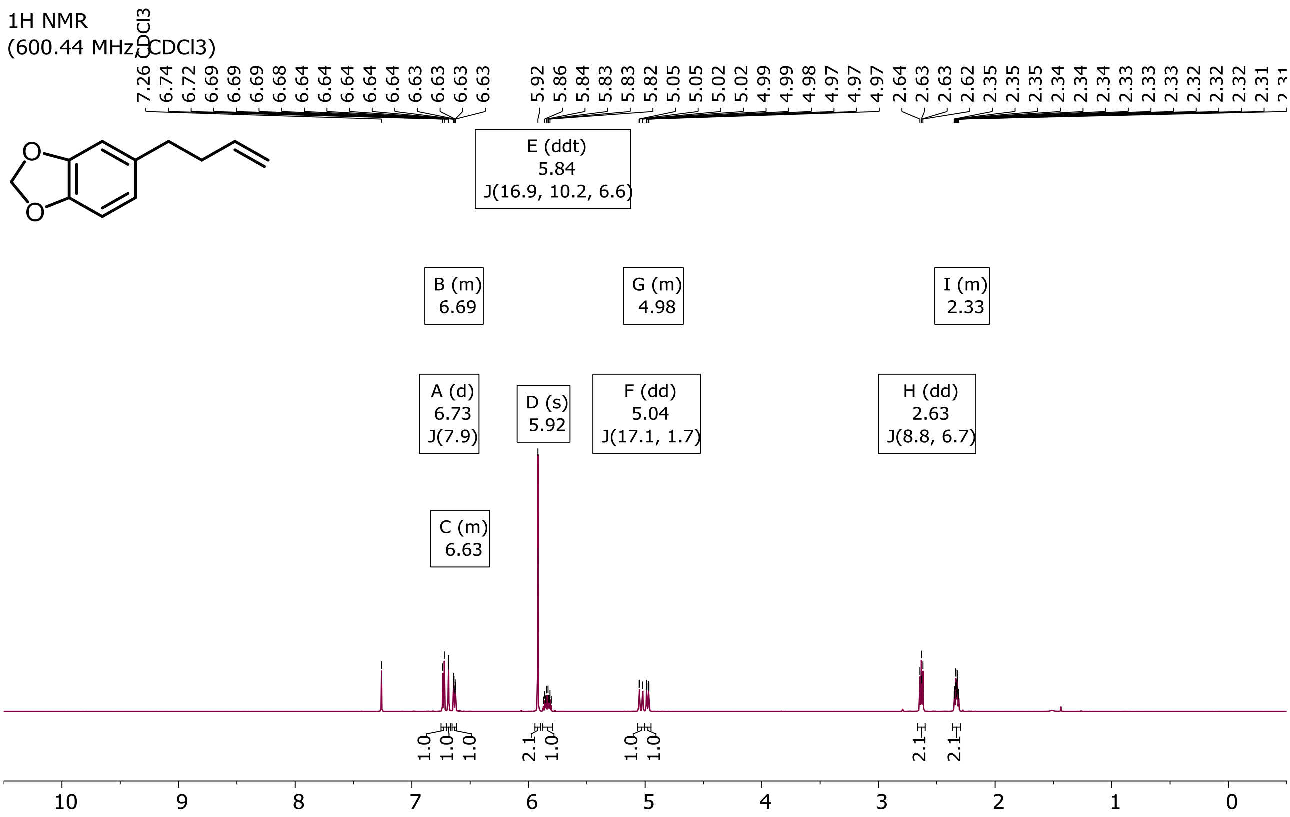

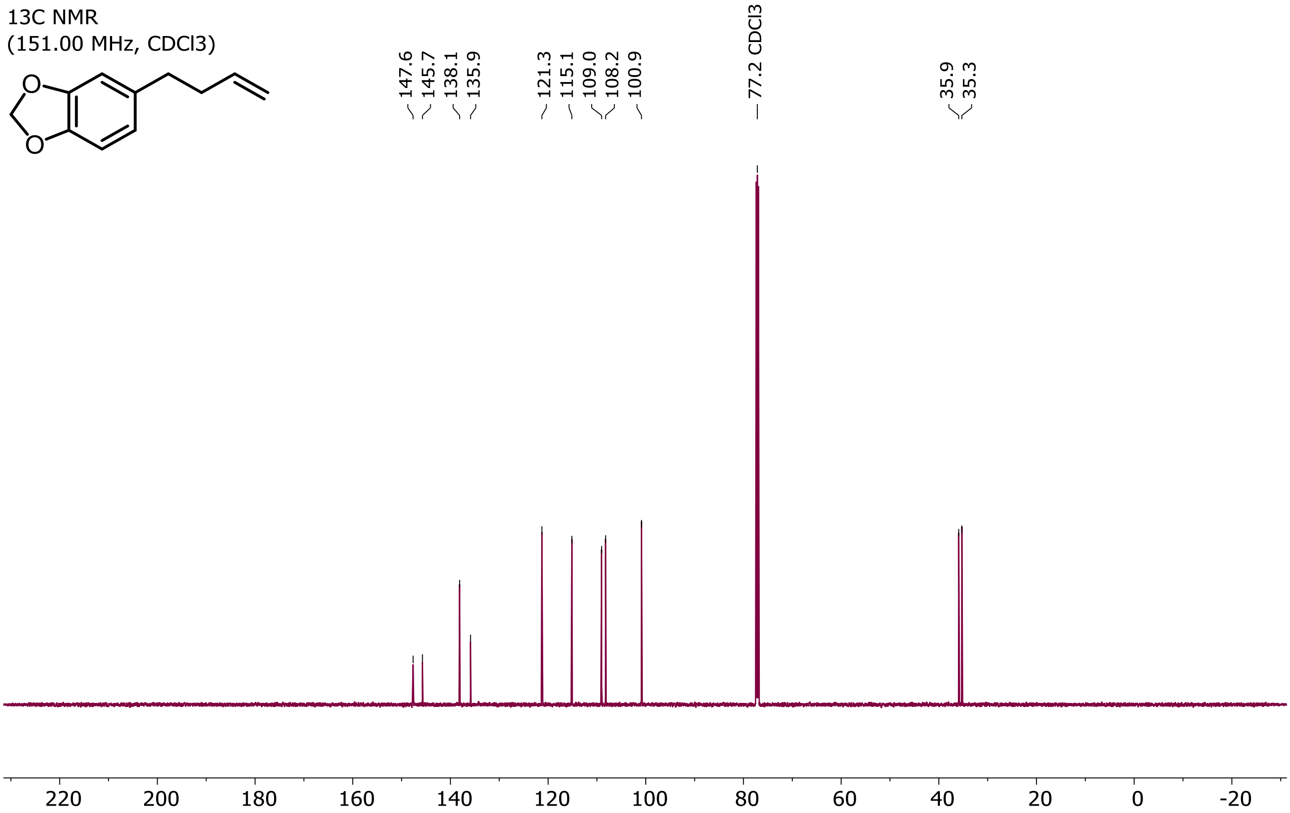

(4-(But-3-en-1-yl)phenyl)(methyl)sulfane (S33)

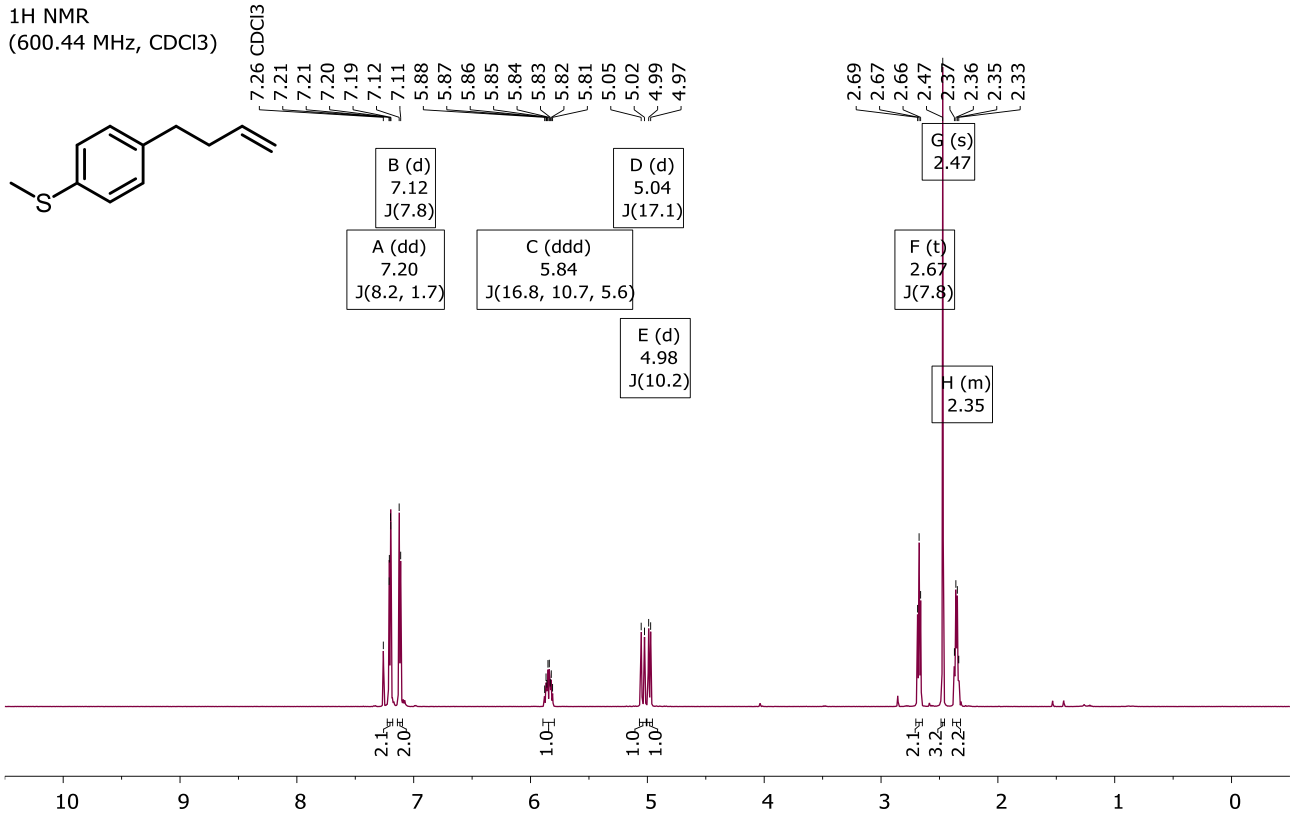

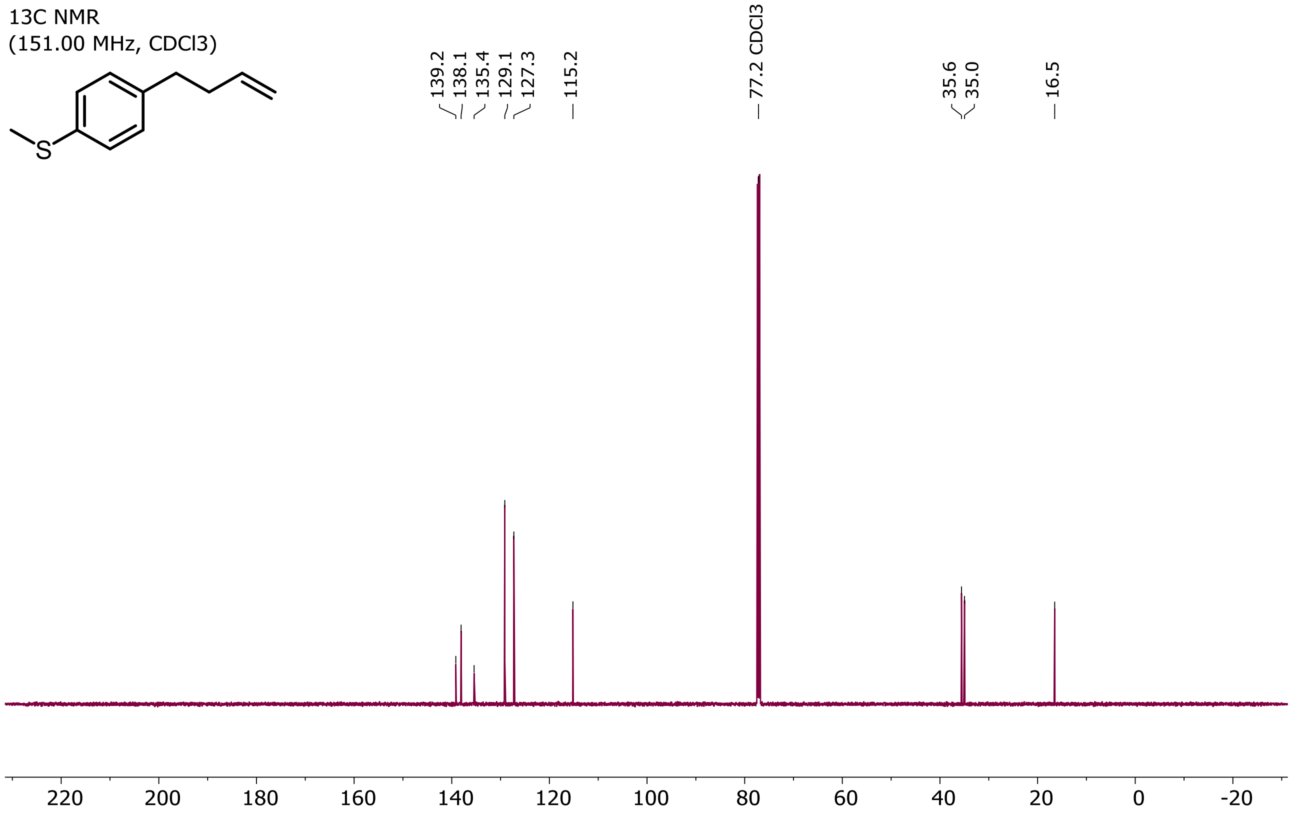

(3-Methylbut-3-en-1-yl)benzene (S34)

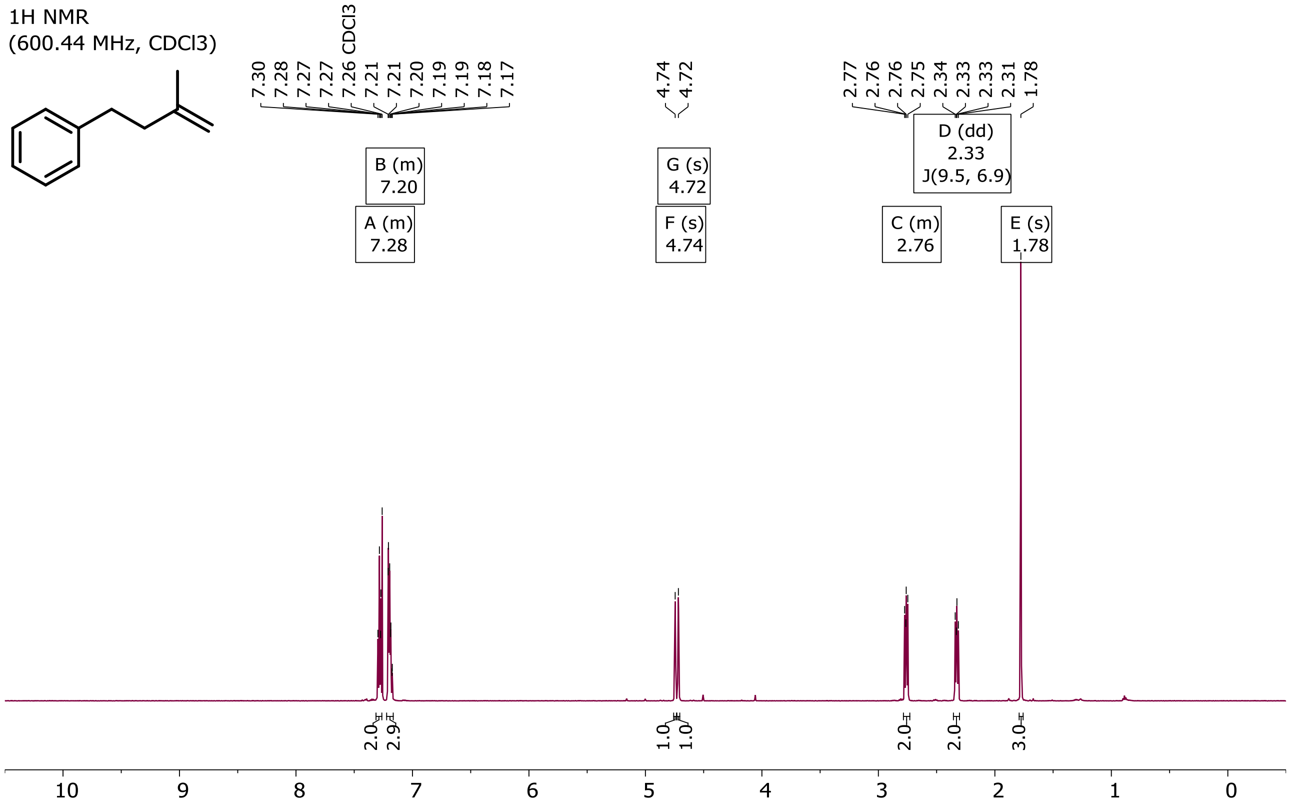

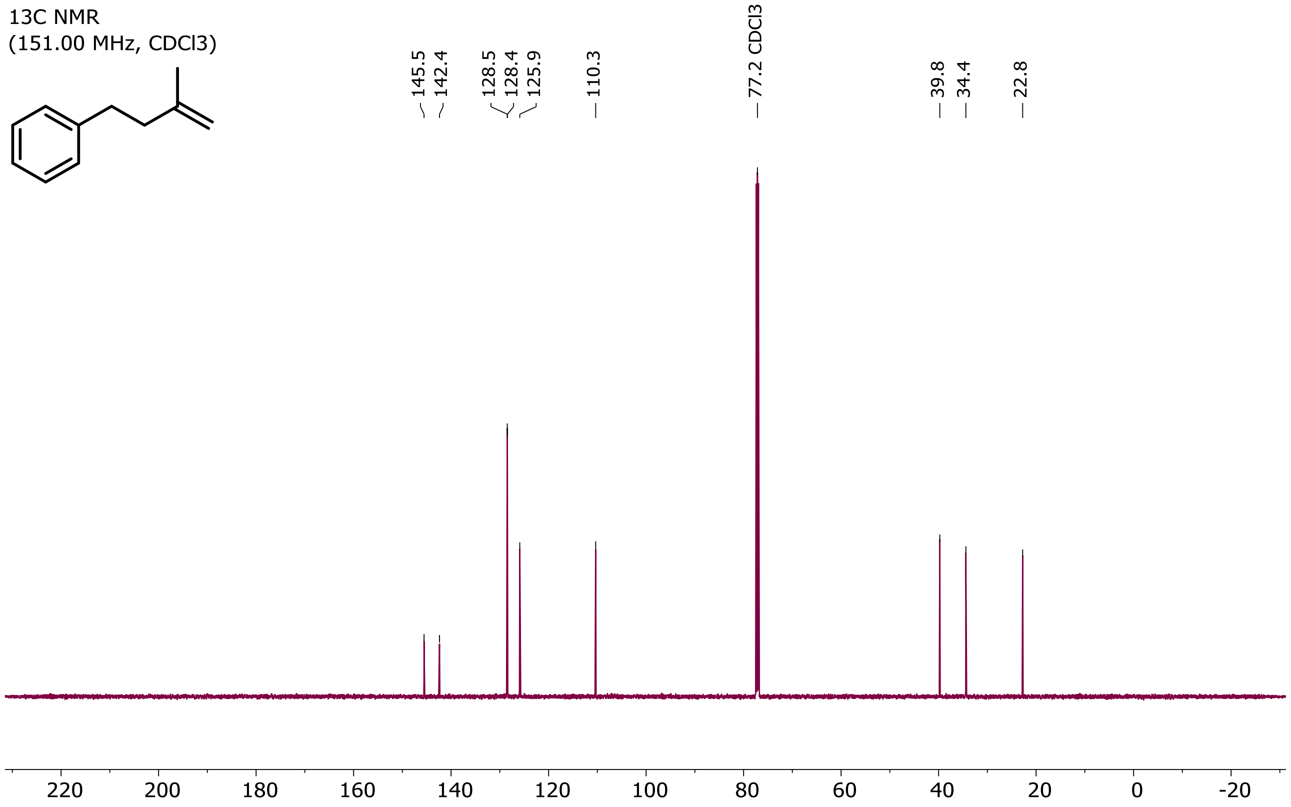

8-(But-3-en-1-yl)quinoline (*S39*)

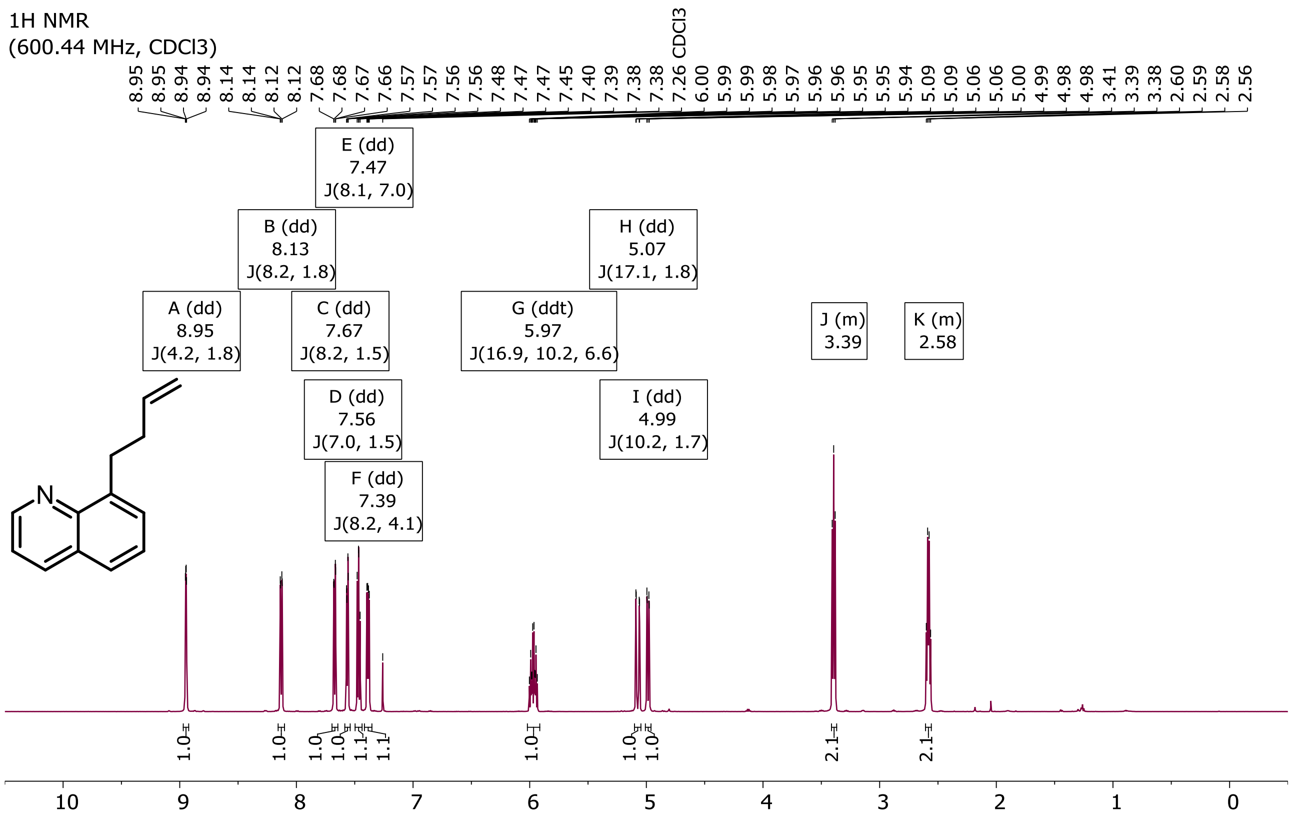

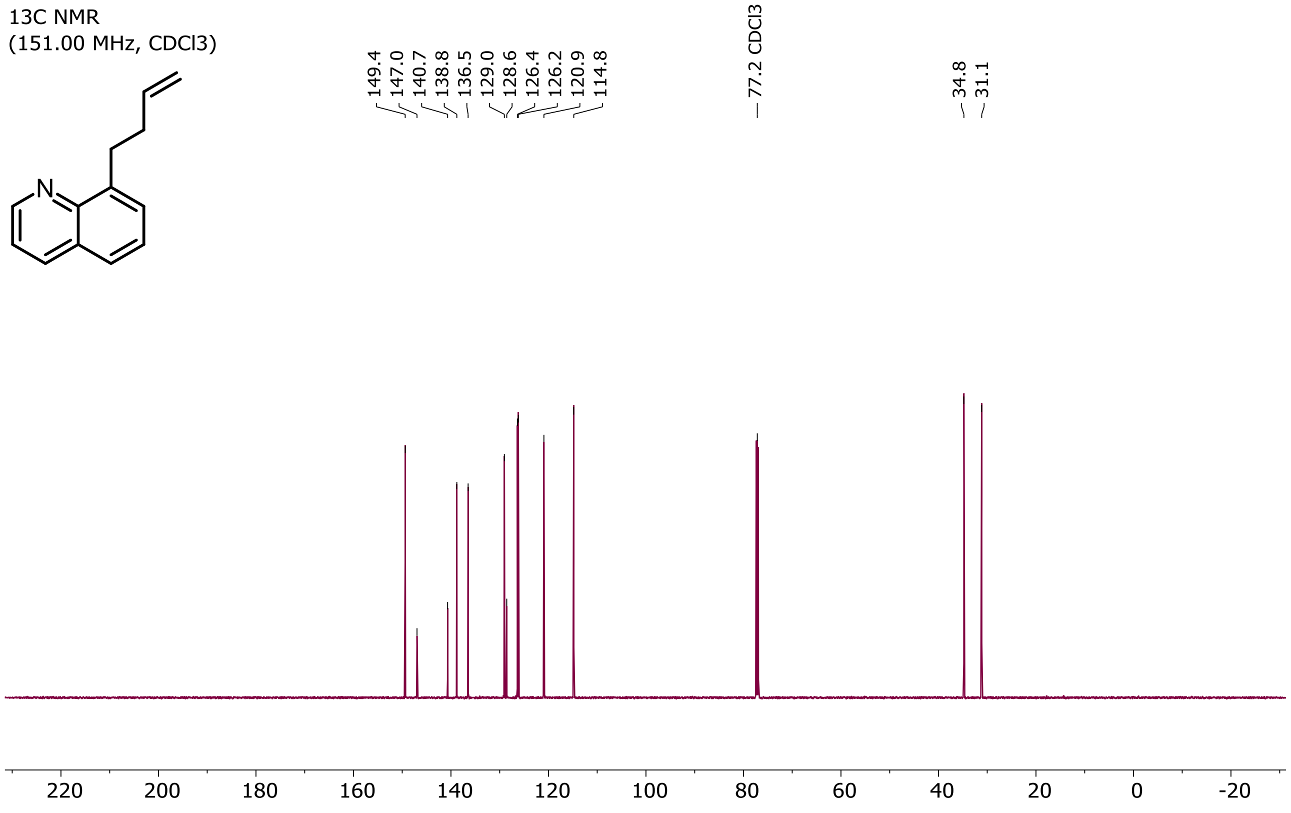

8. References

[1] S. Keess, M. Oestreich, *Org. Lett.* **2017**, *19*, 1898-1901.

[2] K. Yoshizawa, B.-X. Li, T. Matsuyama, C. Wang, M. Uchiyama, *Chem. Eur. J.* **2024**, *30*, e202401546.

[3] S. Mandal, J. Banerjee, S. Maity, S. K. Chattopadhyay, *Helv. Chim. Acta* **2021**, *104*, e2000216.

[4] R. Liu, Z.-H. Lu, X.-H. Hu, J.-L. Li, X.-J. Yang, *Org. Lett.* **2015**, *17*, 1489-1492.

[5] Q.-Y. Meng, T. E. Schirmer, K. Katou, B. König, *Angew. Chem. Int. Ed.* **2019**, *58*, 5723-5728.

[6] H. Deguchi, K. Hanaya, T. Sugai, S. Higashibayashi, *Chem. Commun.* **2023**, *59*, 748-751.

[7] A. Selmani, F. Schoenebeck, *Synthesis* **2023**, *55*, 1792-1798.

[8] W.-L. Xu, H. Zhang, Y.-L. Hu, H. Yang, J. Chen, L. Zhou, *Org. Lett.* **2018**, *20*, 5774-5778.

[9] E. Bergamaschi, F. Beltran, C. J. Teskey, *Chem. Eur. J.* **2020**, *26*, 5180-5184.

[10] J. J. Gladfelder, S. Ghosh, M. Podunavac, A. W. Cook, Y. Ma, R. A. Woltornist, I. Keresztes, T. W. Hayton, D. B. Collum, A. Zakarian, *J. Am. Chem. Soc.* **2019**, *141*, 15024-15028.

[11] Q. Ge, J. Meng, H. Liu, Z. Yang, Z. Wu, W. Zhang, *Chin. J. Chem.* **2022**, *40*, 2269-2275.

[12] A. J. Kennedy, T. P. Mathews, Y. Kharel, S. D. Field, M. L. Moyer, J. E. East, J. D. Houck, K. R. Lynch, T. L. Macdonald, *J. Med. Chem.* **2011**, *54*, 3524-3548.

[13] S. K. Kristensen, S. L. R. Laursen, E. Taarning, T. Skrydstrup, *Angew. Chem. Int. Ed.* **2018**, *57*, 13887-13891.

[14] R. Matsubara, A. C. Gutierrez, T. F. Jamison, *J. Am. Chem. Soc.* **2011**, *133*, 19020-19023.

[15] A. Shiozuka, K. Sekine, T. Toki, K. Kawashima, T. Mori, Y. Kuninobu, *Org. Lett.* **2022**, *24*, 4281-4285.

[16] S. Datta, C.-L. Chang, K.-L. Yeh, R.-S. Liu, *J. Am. Chem. Soc.* **2003**, *125*, 9294-9295.

[17] Y. Li, Y.-Y. Hu, S.-L. Zhang, *Chem. Commun.* **2013**, *49*, 10635-10637.

[18] J. C. Siu, J. B. Parry, S. Lin, *J. Am. Chem. Soc.* **2019**, *141*, 2825-2831.

[19] S. Xia, D. Cao, H. Zeng, L.-N. He, C.-J. Li, *JACS Au* **2022**, *2*, 1929-1934.

[20] H. Kondo, Y. Yamanoi, H. Nishihara, *Chem. Commun.* **2011**, *47*, 6671-6673.

[21] P. Pracht, F. Bohle, S. Grimme, *Phys. Chem. Chem. Phys.* **2020**, *22*, 7169-7192.

[22] a) C. Bannwarth, S. Ehlert, S. Grimme, *J. Chem. Theory Comput.* **2019**, *15*, 1652-1671; b) S. Grimme, *J. Chem. Theory Comput.* **2019**, *15*, 2847-2862.

[23] M. J. Frisch, G. W. Trucks, H. B. Schlegel, G. E. Scuseria, M. A. Robb, J. R. Cheeseman, G. Scalmani, V. Barone, G. A. Petersson, H. Nakatsuji, X. Li, M. Caricato, A. V. Marenich, J. Bloino, B. G. Janesko, R. Gomperts, B. Mennucci, H. P. Hratchian, J. V. Ortiz, A. F. Izmaylov, J. L. Sonnenberg, Williams, F. Ding, F. Lipparini, F. Egidi, J. Goings, B. Peng, A. Petrone, T. Henderson, D. Ranasinghe, V. G. Zakrzewski, J. Gao, N. Rega, G. Zheng, W. Liang, M. Hada, M. Ehara, K. Toyota, R. Fukuda, J. Hasegawa, M. Ishida, T. Nakajima, Y. Honda, O. Kitao, H. Nakai, T. Vreven, K. Throssell, J. A. Montgomery Jr., J. E. Peralta, F. Ogliaro, M. J. Bearpark, J. J. Heyd, E. N. Brothers, K. N. Kudin, V. N. Staroverov, T. A. Keith, R. Kobayashi, J. Normand, K. Raghavachari, A. P. Rendell, J. C. Burant, S. S. Iyengar, J. Tomasi, M. Cossi, J. M. Millam, M. Klene, C. Adamo, R. Cammi, J. W. Ochterski, R. L.

Martin, K. Morokuma, O. Farkas, J. B. Foresman, D. J. Fox, *Gaussian 16, Revision A.03,* **2016**, Gaussian, Inc., Wallingford, CT.

[24] F. Eckert, A. Klamt, *COSMOtherm,* Version C3.0, Release 17.01, **2016**, COSMOlogic GmbH & Co. KG, Leverkusen (Germany), http://www.cosmologic.de.

[25] TURBOMOLE V7.5.1 **2021**, a development of University of Karlsruhe and Forschungszentrum Karlsruhe GmbH (1989-2007), TURBOMOLE GmbH (since 2007); available from https://www.turbomole.org.

[26] C. Y. Legault, *CYLview,* Version 1.0b, **2009**, Université de Sherbrooke, http://www.cylview.org.
